# Bioinsecticide-Predator Interactions: Azadirachtin Behavioral and Reproductive Impairment of the Coconut Mite Predator *Neoseiulus baraki*

**DOI:** 10.1371/journal.pone.0118343

**Published:** 2015-02-13

**Authors:** Debora B. Lima, José Wagner S. Melo, Nelsa Maria P. Guedes, Lessando M. Gontijo, Raul Narciso C. Guedes, Manoel Guedes C. Gondim

**Affiliations:** 1 Departamento de Agronomia, Área de Fitossanidade, Universidade Federal Rural de Pernambuco, Recife, Pernambuco, 52.171-900, Brazil; 2 Departamento de Entomologia, Universidade Federal de Viçosa, Viçosa, Minas Gerais, 36570-900, Brazil; 3 Universidade Federal de Viçosa–Campus Florestal, Florestal, Minas Gerais, 35690-000, Brazil; New Mexico State University, UNITED STATES

## Abstract

Synthetic pesticide use has been the dominant form of pest control since the 1940s. However, biopesticides are emerging as sustainable pest control alternatives, with prevailing use in organic agricultural production systems. Foremost among botanical biopesticides is the limonoid azadirachtin, whose perceived environmental safety has come under debate and scrutiny in recent years. Coconut production, particularly organic coconut production, is one of the agricultural systems in which azadirachtin is used as a primary method of pest control for the management of the invasive coconut mite, *Aceria guerreronis* Keifer (Acari: Eriophyidae). The management of this mite species also greatly benefits from predation by *Neoseiulus baraki* (Athias-Henriot) (Acari: Phytoseiidae). Here, we assessed the potential behavioral impacts of azadirachtin on the coconut mite predator, *N. baraki*. We explored the effects of this biopesticide on overall predator activity, female searching time, and mating behavior and fecundity. Azadirachtin impairs the overall activity of the predator, reducing it to nearly half; however, female searching was not affected. In contrast, mating behavior was compromised by azadirachtin exposure particularly when male predators were exposed to the biopesticide. Consequently, predator fecundity was also compromised by azadirachtin, furthering doubts about its environmental safety and selectivity towards biological control agents.

## Introduction

The use of synthetic pesticides has been the dominant method of agricultural pest control since the early 1940s [1–2]. However, the continuing shift in society’s attitudes and behaviors towards crop protection products has led to drastic changes in the development of new pesticides, where emphasis is placed on improved human and environmental safety profiles [3–6]. The science behind the negative perception of synthetic pesticides, which is deeply ingrained among the general public, is debatable as it is largely based on insecticides such as organochlorines that have been banned for over 40 years [1, 3, 6–7]. Curiously, more than 70% of the current groups of synthetic insecticides have natural analogs [8]. This fact, together with the perceived general (and invalid) notion that natural compounds are safer than their synthetic counterparts [9–10], explains the allure of natural pesticides, or biopesticides, and the drastic reemergence of interest in these compounds, particularly compounds that are plant-derived, also referred to as botanical pesticides [11–13].

The current burgeoning of scientific interest in biopesticides in general, and in botanical pesticides in particular, has only led to a limited amount of credible information and to a small increase in their practical use as crop protection agents [13–14]. Slow action, brief persistence, relatively high cost for large-scale production, and legislative limitations are the main reasons for the limited expansion of biopesticide use in agriculture [11, 14–15]. The 1960s Western discovery of the insecticidal activity of the limonoid triterpene azadirachtin, extracted from the seeds of the Indian neem tree (*Azadirachta indica* A. Juss (Meliaceae)), is one of the likely catalysts of the latest growth in interest and spurt in academic research on botanical insecticides, as well as the subsequent commercialization of plant essential oils as insecticides [12–13]. It is also interesting that azadirachtin remains the most successful botanical pesticide in agricultural use worldwide [13, 16].

Azadirachtin arguably stands out as the most widely used botanical pesticide since the onset of synthetic pesticides for pest control, which is well established in organic agriculture, public health, home and garden, and selected agricultural settings [16–17]. This biopesticide has unique features and can act as an arthropod anti-feedant, growth regulator and sterilant, while its safety to vertebrates is broadly recognized [11, 16]. However, the earlier perception of azadirachtin’s safety towards non-target arthropods has been questioned [18–20]. Such a change in perception is the likely consequence of a shifting in focus, from reliance on acute lethal effects, to sublethal effects of insecticidal compounds [21–23].

Phytophagous mites and their predators are a focus of attention not only regarding the sublethal impact of crop protection compounds, but also regarding the effect that azadirachtin has on these species [24–26]. The coconut production system, particularly organic production, represents one of the agricultural systems where azadirachtin use is important for controlling the coconut mite, *Aceria guerreronis* Keifer (Acari: Eriophyidae). The management of *A*. *guerreronis* also benefits from the predatory mite species, *Neoseiulus baraki* (Athias-Henriot) (Acari: Phytoseiidae) [27–30]. The lethal effect of acaricides in the predatory mite *N*. *baraki* has been a subject of attention. Azadirachtin was recognized as exhibiting low acute toxicity to *N*. *baraki*, but was shown to spark behavioral avoidance on this predator, potentially limiting its foraging behavior [30–31]. Here, we assessed the potential sublethal behavioral effects of azadirachtin, at its label rate for controlling the coconut mite, and the potential consequences in the overall activity, mating and fecundity of the coconut mite predator *N*. *baraki*.

## Materials and Methods

### Ethics Statement

This study did not involve any endangered or protected species. The species studied is a species of predatory mite from a colony maintained in laboratory, where the experiments were performed and no specific permission was required.

### Predatory mites and azadirachtin

Specimens of the mite predator *N*. *baraki* were field-collected from coconut fruits infested with the coconut mite, *A*. *guerreronis*, on Itamaracá Island (07°46’S, 34°52’W; Pernambuco, Brazil). Predator colonies were established from 100 females, which were obtained from, and maintained on, coconut perianth. *Aceria guerreronis* was provided as prey every other day. The mites were maintained under laboratory conditions at 27.5 ± 0.5°C, 70 ± 10% RH, and 12:12 (LD) photoperiod.

Azadirachtin was the insecticide/acaricide used in the experiments. The compound was used in its commercial formulation (AzaMax, 1.2 g a.i./L, emulsifiable concentrate, DAV Agro, Ituverava, SP, Brazil) at the label rate registered and recommended for the coconut mite, *A*. *guerreronis* in Brazil (i.e., 30 mg a.i./L) [17]. No predatory mite mortality takes place at this insecticide concentration, which is sublethal to *N*. *baraki* based on previous determinations [30], preventing any confounding effect of mortality on the sublethal experiments performed. Indeed no azadirachtin mortality was observed in the experiments here performed, as expected.

### Overall mite group activity

Rather than assessing individual mite activity, bioassays of the overall group activity were performed with unsexed adult predatory mites in congruence with the aggregate distribution of the species observed on coconut fruits [32–34]. The methods used in this study were adapted from Lima et al. [30] as follows: individual discs of black polyvinyl chloride (PVC; 1.2 cm diameter) were immersed for 5 s in azadirachtin solution (30 mg a.i./L) and allowed to air-dry for 2 h before being glued to a piece of wood (1 cm thick) and placed in the center of a Petri dish (6 cm diameter) containing water (0.5 cm deep). This set up allowed the PVC disc to float on the surface of the water, preventing mite escape. Each disc received 10 adult couples of the predatory mite (8 days old) and eight disc arenas were used for each treatment (i.e., azadirachtin-treated discs as well as untreated control discs, were only water was used). The overall mite group activity in each disc arena was digitally recorded for 10 min by an automated video tracking system per unit of time (ViewPoint LifeSciences, Montreal, Quebec, Canada). The overall activity was digitally determined by the change in captured pixels per fraction of time (Δ pixels/s x 10^–2^) corresponding to summation of any change in position and posture of the individuals within the arena. The length of time that the mites spent inactive (variation lower than 4 pixels/s x 10^–2^), under slow (variation between 4 and 8 pixels/s x 10^–2^) or fast activity (variation over 8 pixels/s x 10^–2^) was also recorded, as was the rate of change in activity within each of these three categories. The bioassays were performed under 27 ± 2°C.

### Male mate-searching behavior

Pieces of coconut perianth (0.5 cm^3^) were placed in individual wells of bioassay trays with an adhesive cover (128 cells; Bio-Serv, Frenchtown, NJ, USA) and subsequently immersed for 5 s in either azadirachtin solution (30 mg a.i./L) or water (control), and allowed to dry for 2 h. Individual virgin male and female mites (8 days old) were released in each well containing a treated piece of coconut perianth (0.5 cm^2^) and were confined for 16 hours of exposure. Approximately 200 coconut mites (*A*. *guerreronis*) were also transferred to each well to serve as a food source for the predators. After insecticide exposure, each virgin male mite was released at the edge of a PVC disc arena (1.9 cm diameter), which was surrounded by a layer of glycerin to prevent escape. The opposite margin of each PVC disc contained an opening (0.5 cm diameter) covered with voile, under which 10 virgin females were contained within the cut bottom of an Eppendorf tube (1 cm diameter). The mite walking pattern when in search of the virgin females was recorded using the ViewPoint video tracking system. This system recorded the length of time it took each male to find the contained (virgin) females, and lasted for up to 10 min. The following treatments were used, each with 20 replicates: untreated male with untreated females, azadirachtin-treated male with untreated females, untreated male with azadirachtin-treated females, and azadirachtin-treated male with azadirachtin-treated females.

### Mating behavior and associated fecundity

The mating behavior of the predatory mite, *N*. *baraki*, was recorded and assessed by building ethograms and analyzing the first order sequential behavioral transitions and time budgets observed, as well as the lifetime fecundity of each couple. Each virgin mite couple was exposed to azadirachtin, or not, as previously described (subsection “*Male mate-searching behavior”*). Male and female were placed at opposite sides on the surface of a PVC disc arena (0.25 cm). The treatments employed were the same described in the subsection “*Male mate-searching behavior”*, namely: untreated male with untreated females, azadirachtin-treated male with untreated females, untreated male with azadirachtin-treated females, and azadirachtin-treated male with azadirachtin-treated females. Twenty replicates (i.e., couples) were used for each treatment. The mating behavior of each couple was recorded following the protocol of Pappas et al. [35]. Briefly, the initial approach between male and female were characterized by contact using their anterior (gnathosoma to gnathosoma), lateral (male’s gnathosoma to female’s lateral part of idiosoma) or posterior portions (male’s gnathosoma to female’s posterior part of idiosoma). The male subsequently climbed on the female, moved into the mating position (venter-to-venter) and finally copulated [35]. The recording continued until the end of the first mating, when the couple separates and the experiment was interrupted. The behavioral traits assessed included: walking, male and female meeting, mounting, and copulating. At the end of mating, the females were retrieved and individualized in untreated pieces of perianth (0.5 cm^3^) within bioassay trays (128 cells) and provided with *A*. *guerreronis* as a food source. The piece of perianth was replaced every other day, and egg-laying was recorded daily until female death. This bioassay was performed under the same controlled environmental conditions of mite rearing.

### Statistical analyses

The assumptions of normality and homoscedasticity were checked (PROC UNIVARIATE; SAS v. 9) [36], and log_10_x transformation was necessary to stabilize the variance for male mate-searching time, male walking time until female mounting, and duration of mounting. Data from overall group activity (Δ pixels/s x 10^–2^) and total female fecundity (no. eggs laid/female) were subjected to analysis of variance (PROC GLM; SAS v. 9) [36], as were the data from male mate-searching behavior (min), where treatment differences were subsequently subjected to Tukey’s HSD test (*P* < 0.05; SAS v.9) [36]. Ethograms depicting the sequence and frequency of events were manually constructed for each mating treatment based on first order behavioral transitions. The sequence of behavioral transitions was tested for consistency across treatments using Cochran-Mantel-Haenszel statistics (CMH; *P* < 0.05) (PROF FREQ; SAS v. 9) [36], and eventual differences in the proportion of behavioral transitions between treatments were compared using the χ^2^ test (*P* < 0.05). The eventual differences in the recorded time budgets were also subjected to individual analysis of variance and Tukey’s HSD test (*P* < 0.05), when appropriate (PROC GLM; SAS v. 9) [36].

Daily mite fecundity (no. eggs daily laid/female) was subjected to linear regression analysis against female lifetime using the curve-fitting procedure of TableCurve 2D (Systat, San Jose, CA, USA). The significant regression models (*P* < 0.05) were tested from the simplest (linear and quadratic) to more complex peak models and the model selection was based on parsimony, high F-values (and mean squares), and steep increases in R^2^ with model complexity. Residual distribution was also checked for each analysis to validate parametric assumptions.

## Results

### Overall mite group activity

**Fig 1:**
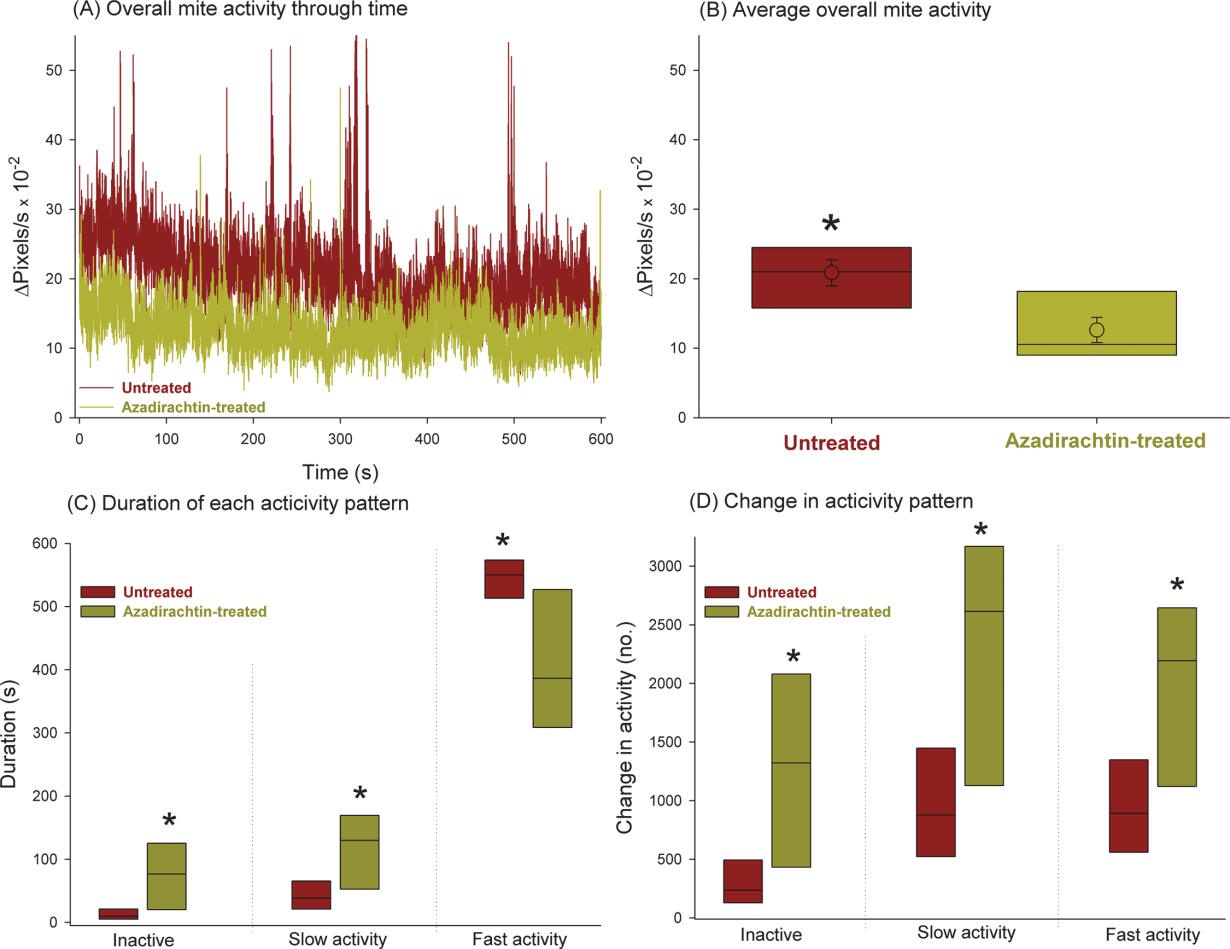
Overall activity of groups of the coconut mite predator *Neoseiulus baraki* exposed to azadirachtin represented as: (A) activity profile through time: (B) average overall activity; (C) duration of each activity pattern; (D) changes in activity pattern. Individual group profiles are represented (A), while box plots with median (and mean ± SE for (B)) and lower and upper quartiles are exhibited in the remaining plots (B, C, D). Box plots with an asterisk indicate significant differences between azadirachtin-treated and untreated mites (Fisher’s F test at *P* < 0.05).

The profile of overall mite group activity through time, exhibited in Fig. 1A, is suggestive of higher activity levels among untreated predatory mites, which was confirmed with subsequent analysis of variance for the average overall activity during the assessment period (F_1,14_ = 10.09, *P* = 0.007) (Fig. 1B). The duration spent in each level of activity, either inactive, or under slow or fast activity, also varied significantly between azadirachtin-treated and untreated predatory mites (F_1,14_ ≥ 7.97, *P* ≤ 0.01). Fast activity prevailed in untreated mites, in contrast with azadirachtin-treated predatory mites, which remained inactive and under slow activity for longer lengths of time (Fig. 1C). Furthermore, there were significant differences in the change of overall activity patterns in groups of mites either azadirachtin-treated or untreated, with the former experiencing significantly higher changes in activity (F_1,14_ ≥ 9.30, *P* ≤ 0.009) (Fig. 1D).

### Male mate-searching behavior

The length of time it took virgin male predatory mites to first find virgin females was subjected to analysis of variance (after data transformation); however, no significant difference was found between treatments (i.e., untreated mites of both sexes, azadirachtin-treated mites of either sex, and azadirachtin-treated mites of both sexes) (overall mean: 2.41 ± 0.30 min to first find the females) (F_3,76_ = 1.54, *P* = 0.21).

### Mating behavior and associated fecundity

**Fig 2:**
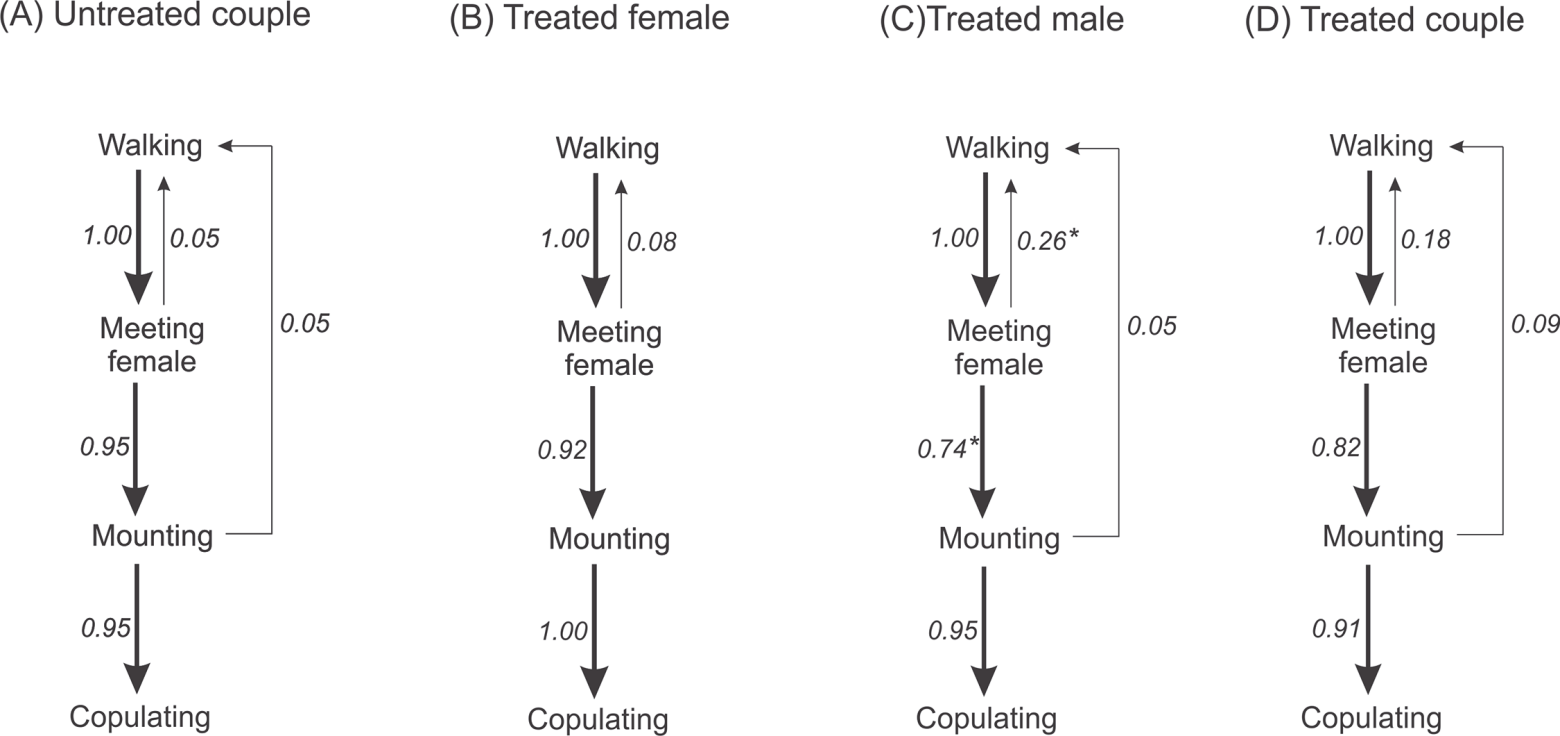
Ethogram of the mating behavior of the coconut mite predator *Neoseiulus baraki* with and without exposure to azadirachtin represented as first order transition diagrams. The solid arrows indicate each behavioral transition. The relative thickness of each arrow represents the frequency of each behavioral transition (*n* = 20). Asterisk indicates significant difference in behavioral transition by the χ^2^ test (*P* < 0.05).

The sequential analysis of the first order of behavioral transitions for each treatment involving azadirachtin-treated and untreated mites was significant and consistent across treatments (CMH non-zero correlation = 30.53, df = 1, *P* < 0.001). Regarding the individual behavioral transitions, a significant difference was detected for the transition between the male meeting the female and either mounting or returning to walk, with significantly larger failure to mount when azadirachtin-treated males were attempting to mate (χ^2^ = 4.1, df = 1; *P* = 0.04) (Fig. 2).

**Fig 3:**
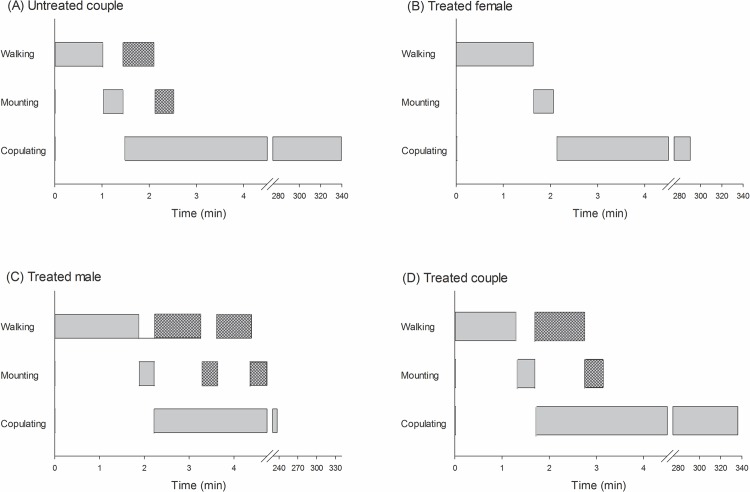
Schematic representation of time budgets of the mating behavior of the coconut mite predator *Neoseiulus baraki* with and without exposure to azadirachtin (*n* = 20). The horizontal histogram bars indicate the average duration of each behavior. The dashed bars indicate events that were repeated before copulation eventually occurred, as indicated in the transition diagrams of Fig. 2.

**Fig 4:**
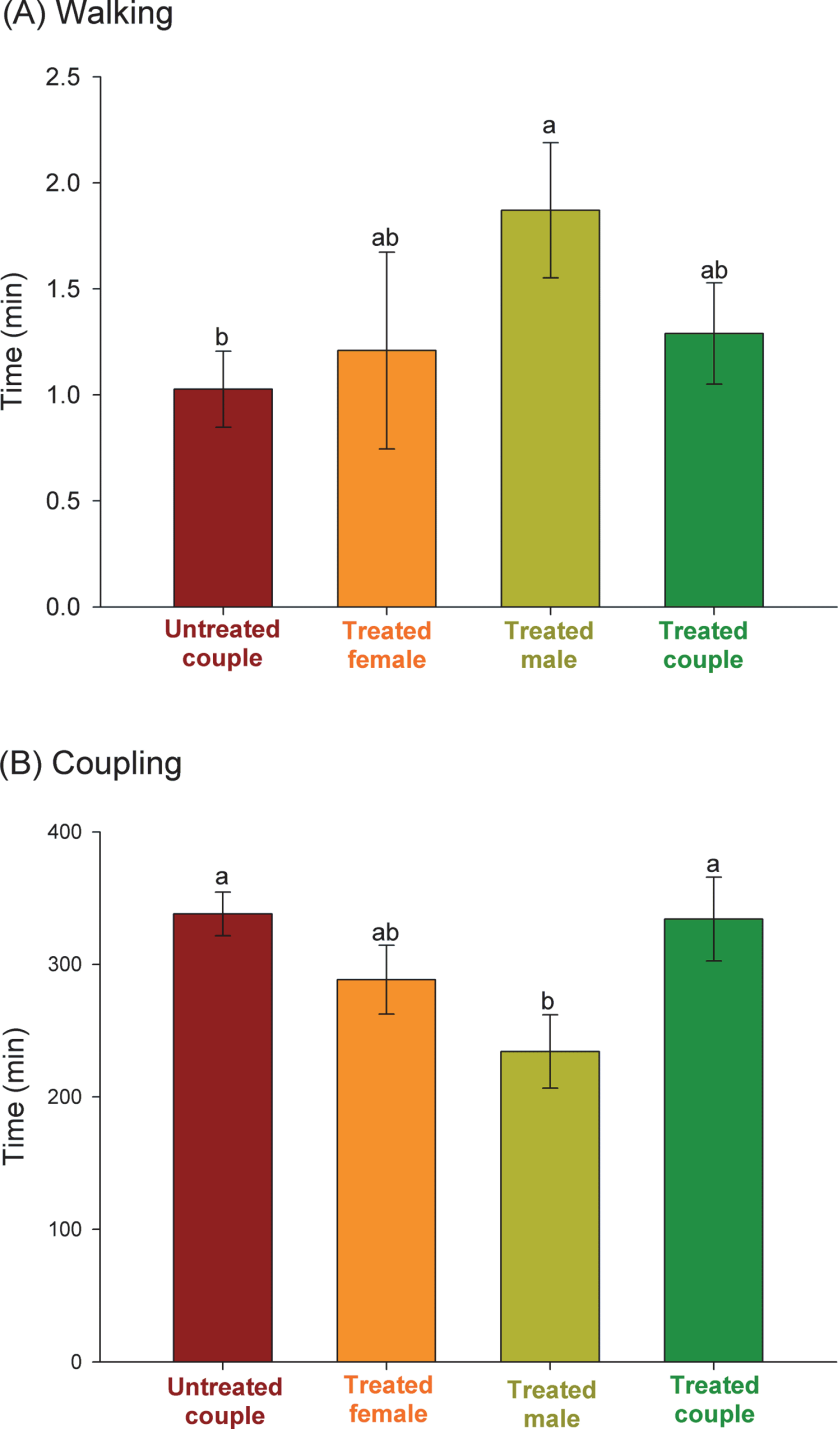
Duration of walking (± SE) of male mites (A) and duration of copulation (± SE) in pairs of the coconut mite predator *Neoseiulus baraki* with and without exposure to azadirachtin (*n* = 20). Different letters at the top of the histogram bars indicate significant differences by Tukey’s HSD test (*P* < 0.05).

The time budgets were also recorded for each mating treatment and are exhibited in Fig. 3. The length of time spent walking and in mounting attempts when male mites were exposed to azadirachtin is notable (i.e., when only males were exposed and when both male and females were exposed), with mites incurring up to three attempts of mounting the female before copulating (Fig. 3CD). Among the three recorded durations of the behaviors leading to mating, walking and copulating were significantly different among treatments (F_3,76_ ≥ 2.65, *P* ≤ 0.05), in contrast with mounting, which was similar among treatments (overall mean = 0.41 ± 0.04 min; F_3,76_ = 0.68, *P* = 0.56). Azadirachtin-treated males spent a significantly longer amount of time walking than did untreated males before mounting untreated females. The time spent by treated males walking before mating with azadirachtin-treated females, however, led to intermediate results (Fig. 4A). A distinct trend was apparent for the time spent in copulation. Untreated couples and azadirachtin-treated couples copulated for longer periods of time, while copulation was quickest between azadirachtin-treated males and untreated females (Fig. 4B).

**Fig 5:**
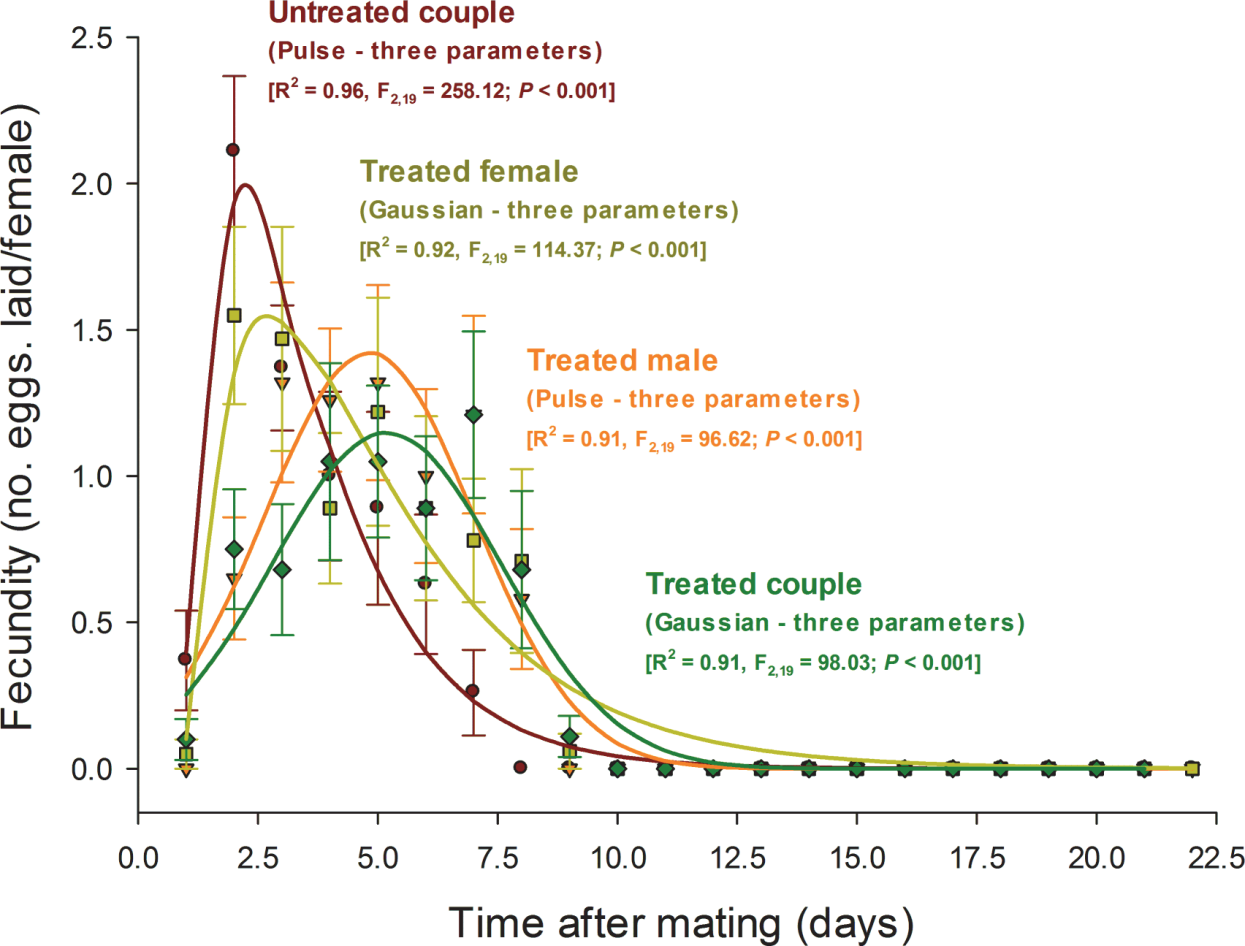
Daily fecundity of females of the coconut mite predator *Neoseiulus baraki* with and without exposure to azadirachtin (*n* = 20). Each symbol indicates the mean (± SE) observed values.

Total female fecundity (no. eggs laid/female) did not differ among treatments (F_3,76_ = 0.12, *P* = 0.95) probably due to the high variability among females within each treatment. However and more importantly, the observed differences in mating among azadirachtin-treated couples, azadirachtin-treated individuals of either sex (i.e, the male or the female of each pair), and untreated couples led to significant differences in daily fecundity (Table 1, Fig. 5). Females from untreated couples exhibited a higher and earlier peak of egg-laying, which was observed approximately 2 days after mating. Azadirachtin-treated females that mated with untreated males exhibited a 0.5 day delay in peak fecundity, with levels that were 25% lower than females from the untreated couples. Females mated with azadirachtin-treated males exhibited even longer delays in peak fecundity, which occurred 5.0 days after mating, and reached levels as low as half that of untreated couples (Fig. 5). Such differences in daily fecundity are more important than total fecundity due to their greater impact in the rate of population growth, as evidenced in life-table and population studies [21,24,25].

**Table 1:** Summary of the non-linear regression analyses of the daily fecundity curves (Fig. 5) of females of the coconut mite predator *Neoseiulus baraki* with and without exposure to azadirachtin (*n* = 20).

Model	Treatments	Parameter estimates (± SE)			df_error_	F	*P*	R^2^
		a	b	c				
Pulse (3-parameter) y = 4*a*n where n = exp(- (x—*b*)/*c*)	Untreated couple	1.94 ± 0.09	0.90 ± 0.03	1.75 ± 0.09	19	258.12	< 0.001	0.96
	Azadirachtin-treated male	1.53 ± 0.11	0.95 ± 0.08	2.64 ± 0.20	19	96.62	< 0.001	0.91
Gaussian (3-parameter) y = *a* exp(- 0.5((x—*b*)/*c*)^2^)	Azadirachtin-treated female	1.42 ± 0.09	4.81 ± 0.17	2.19 ± 0.18	19	114.37	< 0.001	0.92
	Azadirachtin-treated couple	1.15 ± 0.08	5.18 ± 0.20	2.40 ± 0.21	93	201.32	< 0.001	0.91

All parameter estimates were significant at *P* < 0.01 by Student’s *t*-test.

## Discussion

Azadirachtin is a chemical compound representative of the botanical biopesticides, whose safety to non-target arthropods has been a matter of debate, largely due to its reported deleterious effects on natural enemies of arthropod pest species [18–20]. The few studies that have been published investigated a rather small number of species and did not explore the potential impact of detected impairments on the reproductive output of the natural enemies studied [37–40]. Even for the coconut mite predator, to which azadirachtin exhibited low acute lethal effect, behavioral avoidance to this limonoid triterpene was detected, but its impact on the natural enemy longevity and reproduction is not known.

Here, we assessed the impact of azadirachtin in the overall group activity and mating behavior of the predatory mite *N*. *baraki* and assessed its impact on the predator’s fecundity. Azadirachtin is one of the pesticides used against the coconut mite in Brazil, and it is the sole pesticide allowed in organic coconut production systems, where the beneficial control provided by the predatory mite *N*. *baraki* is particularly important [17,28–29]. Our study has both environmental and agricultural relevance. Azadirachtin is reported to exhibit arthropod sterilant activity, in addition to anti-feedant and growth regulator activity. However, behavioral impairment may also compromise exposed arthropods when in low doses.

Azadirachtin reduced the overall activity in groups of the predatory mite. The group determination is consistent with the aggregate pattern of distribution associated with phytoseiid mites and *N*. *baraki* in particular [32–34]. The low activity level detected with sublethal levels of azadirachtin exposure was due to a reduced rate of activity, with mites remaining inactive or under low levels of activity for longer, and frequently changing the pattern of activity from higher to lower levels. Reduced activity may have diverse consequences for the predatory mites, ranging from reduced foraging, to lower dispersal, and possibly compromised mating. To address the later potential consequence, the reproductive behavior of the predatory mite species *N*. *baraki* was investigated after azadirachtin exposure.

Azadirachtin did not compromise female searching for the first meeting between males and females. However, azadirachtin exhibited significant effects on exposed males, extending their latent period before copulation, often requiring multiple mounting attempts before eventual copulation. Untreated males coupled for longer periods of time with females (both azadirachtin-treated and untreated) than did azadirachtin-treated males except when mated with treated females. Therefore, azadirachtin impairs copulation and the end result is reduced fecundity of treated couples, particularly when the males are exposed to this biopesticide.

Azadirachtin does not seem to affect sex pheromone communication between males and females of *N*. *baraki*, as no difference was observed in the time necessary for the males to first locate the females. The observed reproductive impairment likely has endocrine origin, which is consistent with the growth regulator and sterilant activity reported for azadirachtin [16]. The synthesis, transport, and release of morphogenic peptide hormones in the arthropod brain are major components of the azadirachtin mode of action [16, 41]. The detected reproductive effect of azadirachtin is stronger in male mites, impairing mating and compromising fecundity, in contrast with the more frequent reports on female fecundity reduction [16,40]. Here the impact of azadirachtin-treated females was smaller, unlike reports on spider mites [39,40]. The reduction in male fertility caused by azadirachtin has been reported in few instances and only for a few arthropod pest species to the best of our knowledge. These effects have been reported as either a consequence of reduced potency, spermatocyte degeneration, or blocked cell division in developing spermatocytes. The findings have differed depending on the model insect pest species studied [42–44], but there have not yet been any studies on male mites.

The low acute mortality of azadirachtin towards the predatory mite *N*. *baraki* previously reported [30–31] contrasts with its significant (sublethal) reproductive effects reported in the present study. This later finding has potential practical consequences since such reproductive effects may compromise the predator field performance against the coconut mite. The low daily fecundity can lead to changes in the numerical response of the predator, which is the change in predator density as a function of change in prey density [45], and consequently may result in a bigger time lag between prey and predator populations. Although azadirachtin exhibits a safer lethal profile to the predator *N*. *baraki* than alternative compounds used against the coconut mite, the range of choices available for organic coconut production is restricted to this botanical pesticide. Azadirachtin sparks behavioral avoidance in the coconut mite predator *N*. *baraki*, as also reported in lacewings and in contrast with earwigs [20, 46]. This avoidance may potentially favor predator survival while reducing exposure, but may lead the predators to leave the area, compromising the biological control of the coconut mite [30]. More importantly, azadirachtin reduces the predatory mite fecundity, compromising the population growth potential of exposed individuals. Therefore, this phenomenon should be a matter of concern when designing management programs for the coconut mite and gives credence to the recent concerns with the significant deleterious effects of the biopesticide azadirachtin on non-target arthropod species.

## Supporting Information

S1 Dataset Raw data of overall group activity of predatory mites (*Neoseiulus baraki)* exposed or not to azadirachtin. (PDF)

S2 Dataset Raw data of activity duration and frequency of change in activity levels of grouped predators of *Neoseiulus baraki* exposed or not to azadirachtin. (PDF)

S3 Dataset Raw data of the behavioral components of mating behavior of the predatory mite *Neoseiulus baraki* exposed or not to azadirachtin. (PDF)
