# Supplementary material for: Bioinsecticide-Predator Interactions: Azadirachtin Behavioral and Reproductive Impairment of the Coconut Mite Predator Neoseiulus baraki
Source: PLoS One. 2015 Feb 13;10(2):e0118343. doi: 10.1371/journal.pone.0118343 (PMC4334557; doi:10.1371/journal.pone.0118343)
Supplement: S1 Dataset — (PDF) [file pone.0118343.s001.pdf]

time (sec)	Azadirachtin Activity (pixels/sec * 10-2)	time (sec)	Control Activity (pixels/sec * 10-2)
0.0513	17.75	0.0725	27.25
0.0888	19.25	0.1063	36.25
0.1213	19	0.14	30.25
0.1525	18.25	0.1738	28
0.1863	20.25	0.2088	30.75
0.2213	19.5	0.2413	24
0.2525	16.5	0.2738	23.75
0.2863	20.5	0.3063	30.5
0.3163	22.25	0.3413	24
0.3538	17.25	0.3725	23.75
0.3888	17.25	0.4075	28
0.4225	23	0.4413	21.75
0.4538	15.5	0.4775	28
0.485	14.25	0.5075	22.25
0.515	18.5	0.5388	22.5
0.5513	15	0.5725	24
0.5875	15	0.6063	25.25
0.6213	16	0.64	29.75
0.6525	19.75	0.6738	26.75
0.6863	20.5	0.7088	29
0.7213	15.75	0.7413	27
0.7525	20.5	0.7738	28
0.7863	17.5	0.8063	26.75
0.8163	15.5	0.8413	25.5
0.8538	15.5	0.8725	27
0.8888	26.5	0.905	26.75
0.9225	17.5	0.9438	30.5
0.9538	16	0.9775	25.75
0.985	19.75	1.0075	24.5
1.015	15.5	1.0413	27.75
1.0513	15.75	1.0725	28.5
1.0875	14.5	1.1063	25
1.1213	16.25	1.1375	23.25
1.1525	15.5	1.1738	25.25
1.1863	14.25	1.21	20.25
1.2238	16.25	1.2413	22.5
1.2525	17.25	1.2738	21
1.2863	15	1.3063	25.25
1.3163	20	1.3413	23.25
1.3525	16.5	1.3725	23.25
1.3888	13.5	1.405	26.25
1.4225	15	1.4438	25
1.4538	17	1.4775	23.5
1.485	18.75	1.5075	25.5
1.515	16.75	1.5413	23.5
1.5538	16.5	1.5713	20.5
1.5875	13.25	1.6088	23.75

1.6225	16	1.6413	21.5
1.655	20.5	1.6738	21.5
1.69	22	1.7088	22.25
1.7238	18.75	1.7413	20
1.755	16.5	1.7738	18.75
1.7875	18.75	1.8063	21.75
1.8188	20.5	1.8413	25
1.8563	19	1.8725	24
1.8913	29.25	1.905	26
1.9213	19.25	1.9438	21.5
1.955	18.5	1.9775	23.5
1.9863	29	2.0075	24.25
2.02	25.75	2.0413	25
2.0538	18	2.0713	26.5
2.0875	14	2.1063	27
2.1225	13.75	2.1375	28
2.155	13.25	2.1713	22
2.1888	17.25	2.2088	32.75
2.2238	12.25	2.2413	25.5
2.255	18	2.275	29.25
2.2875	16.5	2.3063	24.75
2.3188	12.75	2.3413	26
2.3563	16.75	2.3725	27.25
2.3913	13.25	2.405	28
2.4213	15	2.4438	26.75
2.455	15.5	2.4775	22
2.4863	17.75	2.5075	23.5
2.52	15.75	2.5413	27.5
2.5538	16	2.5713	26
2.5875	15.25	2.6063	26.25
2.6238	15.5	2.6375	24
2.655	16	2.6725	24
2.6888	18.25	2.71	29
2.7238	14.5	2.7413	24.5
2.755	15	2.775	26.5
2.7875	16	2.8063	25
2.8188	14.25	2.8425	26.25
2.8538	16	2.8725	24
2.8913	15.5	2.905	21.25
2.9213	12	2.9425	31.75
2.955	14.25	2.9775	24
2.9863	15.5	3.0075	23.25
3.0213	15	3.0413	22.75
3.0538	17	3.0713	21.25
3.0875	20.25	3.1063	24
3.1225	17.25	3.1375	23.25
3.155	18.75	3.1725	20.25
3.1888	15	3.21	22.5
3.2238	11.75	3.2413	21
3.255	18.75	3.275	26.75

3.2875	13.25	3.3063	21.5
3.3188	13	3.3413	24
3.3513	17.25	3.3725	23
3.3913	17.75	3.405	20.25
3.4213	17	3.4425	23.5
3.455	16.25	3.4775	24.25
3.4863	17.75	3.51	23.75
3.52	17	3.5413	25
3.5538	18.5	3.5713	24.5
3.5875	18.25	3.6063	24.5
3.6225	18	3.6388	23.75
3.655	14.25	3.6725	25.25
3.6913	16	3.71	25.25
3.7238	11.75	3.7413	25.25
3.7563	15.5	3.775	23.5
3.7875	16.25	3.8063	26.5
3.8188	16.5	3.8388	25
3.8513	15.5	3.8738	25.75
3.8913	14	3.9038	27.25
3.9213	14.5	3.94	22.5
3.955	16.75	3.9775	23.5
3.9863	10.75	4.01	22.25
4.02	15.75	4.045	24.5
4.0538	16	4.0713	24.25
4.0875	16	4.1063	24.75
4.1225	13.25	4.1375	25.75
4.155	14.5	4.1725	27.25
4.1888	16.5	4.21	28.5
4.2238	17	4.2413	24.75
4.2563	23.75	4.275	25.25
4.2875	16.25	4.3063	23.5
4.3188	18.75	4.3388	24.75
4.3513	16.25	4.3725	27.5
4.3913	14.75	4.4038	21.25
4.4213	15.5	4.44	25.5
4.455	14.75	4.4788	24.25
4.4863	15.75	4.51	24.75
4.52	15.75	4.5413	27.5
4.5538	17.25	4.5713	24
4.5875	17	4.6038	23.75
4.6225	14.25	4.6375	22.75
4.655	16.75	4.6725	18
4.6888	17.75	4.71	23.5
4.7238	16.75	4.7413	26.5
4.7563	17.25	4.775	22.75
4.7875	17	4.8063	25
4.8188	15.5	4.8388	25.5
4.8513	16.5	4.8725	24.25
4.8913	16	4.9063	28.5
4.9213	15.75	4.94	27.75

4.955	16.25	4.9775	24.5
4.9863	19.25	5.01	22.5
5.02	15.75	5.0413	29.25
5.0538	15.25	5.0713	22.75
5.0875	15.5	5.1038	27.75
5.1213	16.75	5.1375	29
5.155	16.25	5.1725	27
5.1888	16.25	5.21	22.5
5.2238	17.5	5.2425	24.5
5.2563	15	5.275	25.75
5.2875	14.25	5.3063	24.75
5.3188	15	5.3388	23.5
5.3525	16.25	5.3725	23.25
5.3913	15.5	5.4063	26.75
5.4213	14.25	5.44	18.25
5.455	15.75	5.4775	24
5.4863	15	5.51	22.5
5.52	15.5	5.5413	26
5.5538	18.25	5.5713	23.5
5.5875	17.5	5.6038	23.75
5.6213	21.75	5.6375	24.25
5.655	13	5.6738	26
5.6888	21.75	5.71	21.75
5.7238	17.5	5.7425	25
5.7563	17	5.775	26.5
5.7875	15.75	5.8063	23
5.8188	16	5.8388	26.5
5.85	16.25	5.8725	26.5
5.8913	16.25	5.9063	22.5
5.9213	17.25	5.94	23.5
5.955	19	5.9775	26.5
5.9888	16	6.01	21.5
6.02	14.75	6.0413	29.75
6.0538	16.25	6.0713	23.75
6.0875	16.75	6.1013	26.5
6.1213	19.25	6.1375	26
6.155	14.75	6.17	27.25
6.1888	13.5	6.21	27
6.2238	18	6.2425	24.25
6.2563	15.25	6.275	24.25
6.2875	17.75	6.3063	22.25
6.3188	18.75	6.3388	27.25
6.35	18.75	6.3713	26
6.3913	16.75	6.4063	22.75
6.4213	17.5	6.44	25
6.455	16.75	6.4775	23.5
6.4888	13.25	6.51	24.25
6.52	16.5	6.5425	27.5
6.5538	13.75	6.5713	26.25
6.5875	16.25	6.6013	23.75

6.6225	14.5	6.6388	24.75
6.655	19.25	6.67	21
6.6888	14.25	6.71	24.25
6.7238	14.5	6.7425	24
6.7563	15	6.775	27
6.7875	13	6.8063	25.75
6.8188	15.5	6.8388	26.25
6.85	16.75	6.8713	22.75
6.8888	15.5	6.9063	30.25
6.9213	17	6.94	31.5
6.955	14.25	6.98	29.5
6.9888	17.25	7.01	26.25
7.02	16.5	7.0413	24.5
7.0538	13.5	7.0713	21.75
7.0888	14.75	7.1013	28
7.12	17.25	7.1388	29.5
7.155	15.5	7.17	25
7.1888	12.25	7.21	26.75
7.2238	16.25	7.2425	28
7.2563	15.5	7.275	25.25
7.2875	16.25	7.3063	26.5
7.3188	14.5	7.3388	28
7.35	13.75	7.3713	24
7.3888	14.25	7.4063	26.75
7.4213	12.5	7.4425	31.25
7.455	20.75	7.48	22.25
7.4913	14	7.51	27
7.52	12.25	7.5413	23
7.5538	14	7.5713	27.75
7.5875	12.25	7.6013	28.5
7.62	16.75	7.6388	24.25
7.655	15	7.67	24.75
7.6888	16.75	7.71	23
7.7238	17.25	7.7425	24
7.7563	17.5	7.775	28
7.7875	19	7.8063	22
7.8188	12.5	7.8388	26.25
7.85	16.25	7.87	26.5
7.8888	16.75	7.9063	25.5
7.9213	12.75	7.9425	30.25
7.955	16.5	7.98	24
7.9888	13	8.01	30.5
8.02	13.5	8.0413	25
8.0538	12.25	8.0713	30
8.0875	12.5	8.1013	25.25
8.12	13.75	8.1363	23.5
8.1525	11.75	8.17	27.25
8.1888	12	8.21	27.25
8.2263	14.5	8.2425	27
8.2563	12	8.275	27

8.2875	10	8.3063	29
8.3188	14	8.3388	26.5
8.35	12	8.37	29.5
8.3888	16	8.4063	25.5
8.4213	12	8.4425	26
8.455	12	8.48	24.5
8.4888	14.5	8.51	21.5
8.52	12.25	8.5413	26.75
8.5538	14.75	8.5713	24.75
8.5875	16.25	8.6013	24.5
8.62	16.75	8.6363	28
8.6525	13	8.67	26.5
8.6888	14.75	8.71	23.5
8.7263	16.25	8.7425	22.25
8.7563	20.25	8.775	23.75
8.7875	15.25	8.8063	27.75
8.8188	18	8.8388	27.5
8.85	13.75	8.87	24.25
8.8888	17.25	8.9063	22.5
8.9213	13	8.94	23.75
8.955	12.75	8.98	22
8.9888	14	9.01	24.75
9.02	15.25	9.0413	25.5
9.0538	13.25	9.0713	19.75
9.0875	11.5	9.1013	28.25
9.12	15.5	9.1363	22
9.1525	15.75	9.17	24.25
9.1888	9.5	9.2113	31.25
9.2263	12.75	9.2425	31
9.2563	11.75	9.275	27.25
9.2875	11	9.3063	23
9.3188	11.5	9.3388	22.25
9.35	15	9.37	24.75
9.3913	19	9.4063	23.75
9.42	17.75	9.4413	26.5
9.455	12.75	9.48	23.25
9.4913	14	9.51	23.75
9.52	15.5	9.5413	22.5
9.5538	15.25	9.5713	26
9.5875	14.5	9.6013	26.75
9.62	14.5	9.6363	28.5
9.6525	15	9.6725	28.5
9.6888	16.5	9.7113	35
9.7263	16	9.7438	23.5
9.7563	15.75	9.7775	21.5
9.7875	18.25	9.8088	24.25
9.8175	16.75	9.8413	22
9.85	18	9.8725	24.5
9.8888	15.75	9.9063	25.25
9.92	18.75	9.9438	22.25

9.955	15	9.9825	27.5
9.9888	14.25	10.0125	24.75
10.02	17	10.0425	24.25
10.0538	17.75	10.0725	19
10.0875	14.5	10.1025	21.5
10.12	14.25	10.135	25.75
10.1525	15.75	10.1738	21
10.1888	14.5	10.2113	24.25
10.2263	19	10.2438	23.5
10.2563	17.25	10.2775	22.75
10.2875	12.5	10.3088	26.5
10.3175	15.75	10.3413	26.5
10.35	16	10.375	24
10.3888	14.75	10.4063	26.25
10.42	14.5	10.4438	23.75
10.455	14.25	10.4825	25.25
10.4888	17.75	10.5125	30.5
10.52	15	10.5425	31
10.5538	14	10.5725	28.5
10.5875	13.25	10.6025	30.25
10.62	14.75	10.635	31.5
10.6525	12.75	10.675	23.5
10.6863	13	10.7113	24.5
10.7263	10.25	10.7438	28
10.7563	10.25	10.7775	25.75
10.7875	12.5	10.8088	24.25
10.8175	14	10.8413	23.5
10.85	12.5	10.875	27.5
10.8888	10.75	10.9063	25.5
10.92	15.25	10.9438	26
10.9563	9.5	10.9825	26
10.9888	13.5	11.0125	26.5
11.02	10.5	11.0425	27.5
11.0538	10.5	11.0725	26
11.0875	9.5	11.1025	26
11.12	11.25	11.135	27
11.1525	10	11.1738	26
11.1863	13.75	11.2125	28
11.2263	14.5	11.2438	26.5
11.2563	14.25	11.2775	29.25
11.2875	18.25	11.3088	24.75
11.3175	14.75	11.3413	23.5
11.35	17.75	11.375	26.5
11.3888	17	11.4063	27.25
11.42	15.75	11.4438	22
11.4563	17	11.4825	25.25
11.4888	15	11.5125	26.75
11.52	15.5	11.5425	22.5
11.5538	15	11.5725	28
11.5875	16.5	11.6025	29.75

11.62	12.75	11.635	26
11.6525	15.25	11.6738	30.25
11.6863	16.25	11.7125	26.5
11.7263	15.25	11.7463	29.25
11.7563	18	11.7775	25.5
11.7875	12.75	11.8088	23
11.8175	13	11.8413	23.75
11.85	10.75	11.875	20
11.8888	16	11.905	24
11.92	14.25	11.9438	22.5
11.9563	13.75	11.9825	26.75
11.9888	13.5	12.0125	27.5
12.02	13.25	12.0425	21
12.0538	18.25	12.0725	23.75
12.0875	15.75	12.1025	28.25
12.12	11.25	12.135	27.25
12.1525	13.5	12.1738	25.25
12.1863	9.75	12.2113	25.75
12.2263	14.5	12.2438	22.75
12.2563	14.75	12.2775	25.75
12.2875	13	12.3088	23
12.3175	11.5	12.3413	22.5
12.35	14.25	12.375	19.75
12.3888	11	12.405	23
12.42	8.75	12.4438	21.5
12.455	14.5	12.4825	21.75
12.4888	10.25	12.5125	27.75
12.52	10.75	12.5425	26.5
12.5538	10.75	12.5725	26.25
12.585	7.25	12.6025	22.25
12.62	9.5	12.635	22.75
12.6525	10.5	12.6738	27.5
12.6863	14	12.7113	28.75
12.7263	16.5	12.7463	28
12.7563	11.5	12.7775	23
12.7875	13.25	12.8088	19.5
12.8175	10	12.8413	21
12.85	11.75	12.875	26.5
12.8888	12.25	12.905	31.5
12.92	14.75	12.9413	25.5
12.955	13.75	12.9825	27.75
12.9888	15.25	13.0125	23
13.02	13.25	13.0425	27.5
13.0538	15.5	13.0725	21.5
13.085	18.25	13.1025	26.5
13.12	15.5	13.135	23.5
13.1525	12	13.1763	21
13.1863	16	13.2113	23
13.2263	12.5	13.2438	24
13.2563	11.75	13.2775	22.5

13.2875	10.75	13.31	25.5
13.3175	13	13.3413	19.25
13.35	10.5	13.375	25
13.3888	14.75	13.41	26.5
13.42	12.75	13.4438	20.25
13.455	10.25	13.4825	24.5
13.4888	13.25	13.5125	26.75
13.52	9.5	13.5425	23.5
13.5538	13.25	13.5725	19.75
13.585	10.5	13.6025	25
13.62	12	13.635	24
13.6525	14	13.6738	25.75
13.6863	12.75	13.7113	31.25
13.7238	13.75	13.7438	21.75
13.7563	10.25	13.7775	27
13.7875	10	13.8088	23
13.8175	14	13.8413	23.75
13.85	12	13.875	25
13.8888	12.75	13.91	30.5
13.92	15	13.9438	25.25
13.955	16	13.9825	26.25
13.9888	17	14.0125	24.75
14.02	14	14.0425	27.5
14.0538	16	14.0725	29.25
14.085	17.25	14.1025	26.75
14.12	14.75	14.135	33.75
14.155	13.75	14.1738	26.25
14.1863	16	14.21	27.5
14.2238	18.75	14.2438	28
14.2563	19	14.2775	23.25
14.2875	16.75	14.3088	23.5
14.3175	16.75	14.3413	25
14.35	19.25	14.3788	31
14.3888	15	14.415	22.75
14.4213	14.5	14.4475	21.5
14.455	12.5	14.4863	22.5
14.4888	16	14.5163	21.5
14.52	11.25	14.5488	24.25
14.5538	13.5	14.5763	25.25
14.585	16.25	14.6088	21.25
14.62	15.75	14.6388	26.5
14.6525	14	14.6763	24
14.6863	12.5	14.7188	25.75
14.7238	16	14.75	21
14.7563	14.5	14.7825	26.75
14.7875	14.25	14.8138	24
14.8175	16.25	14.8488	24
14.8475	18.5	14.88	22
14.89	19	14.9163	17.5
14.92	16.5	14.95	18.5

14.955	16.5	14.9875	21
14.9875	15.5	15.0188	23.25
15.02	14.75	15.0488	20.5
15.0538	12	15.0788	22.25
15.085	14	15.1113	23.5
15.12	14.25	15.1413	16.5
15.1525	17	15.1763	19.5
15.1888	19.75	15.2188	19
15.2238	16	15.25	21
15.2563	17.25	15.2825	26
15.2875	15.5	15.3138	21.75
15.3175	18.75	15.3488	23.5
15.3475	17	15.38	24.25
15.3888	18	15.4163	24.25
15.42	18.75	15.45	23.5
15.455	14.5	15.4875	25
15.4875	18	15.5188	22.5
15.52	19	15.5488	22.25
15.5538	20.5	15.5788	21.5
15.585	18	15.6138	23.25
15.62	17.25	15.6413	17.75
15.6525	19	15.6763	23.5
15.6888	17	15.7188	19.75
15.7238	18.25	15.75	23.25
15.7563	18.5	15.7825	24.5
15.7875	17.75	15.8138	24.75
15.82	20.75	15.8488	22.25
15.8513	17.25	15.88	21.25
15.8913	18.75	15.9163	19.5
15.9225	23.25	15.95	27
15.9588	17.75	15.9875	26
15.99	15.75	16.0188	23.5
16.0225	15.75	16.0488	19.75
16.055	17.5	16.0788	26.75
16.0863	15.75	16.1113	30
16.1213	17.75	16.1438	26.75
16.1538	17	16.1763	21.5
16.1913	15.75	16.215	26.75
16.225	17.5	16.25	23
16.255	17.75	16.2825	25
16.29	18.5	16.3138	30
16.32	20.25	16.3488	27.75
16.3513	18.25	16.38	25.25
16.3913	17.75	16.4163	25.75
16.4225	16.5	16.45	22.25
16.4588	17.25	16.485	21
16.49	16.25	16.5188	21.75
16.5225	13.75	16.5488	26.5
16.555	14.75	16.5788	24.75
16.5863	14.75	16.6113	21.75

16.6213	17	16.6438	24
16.6538	16	16.6763	29
16.6913	18.75	16.715	25.5
16.725	12.5	16.75	27.5
16.755	13.25	16.7825	27.5
16.79	15.25	16.8138	24.5
16.82	16.75	16.8488	23.5
16.8513	11.75	16.88	26
16.8913	13	16.9163	29
16.9225	11.5	16.9488	22.75
16.9588	12	16.985	25
16.99	18	17.0188	26.75
17.0225	14.75	17.0488	24
17.055	16.25	17.0788	25.75
17.0863	17	17.1113	19.75
17.12	19.25	17.1463	27
17.1538	16	17.1763	25.25
17.1913	17	17.215	24.75
17.225	17.25	17.25	24.75
17.255	16.5	17.2825	24
17.29	15.25	17.3138	21.25
17.32	15	17.3488	20
17.3513	15	17.38	22.5
17.3913	16.5	17.4163	18.5
17.4225	16.25	17.4488	21.5
17.4588	20	17.485	17.5
17.49	16.75	17.5188	17.25
17.5225	17.75	17.5488	18.75
17.555	15.5	17.5788	19.5
17.5863	16.25	17.6113	21.25
17.62	19	17.6463	20.75
17.6538	14.25	17.6763	25.25
17.6913	15.25	17.715	20.25
17.725	15	17.75	23
17.755	15.75	17.7825	23.5
17.79	18	17.8138	20
17.82	15.5	17.8513	20.5
17.8513	18.5	17.88	23.25
17.8913	12.5	17.9163	24.75
17.9225	13.25	17.95	23
17.9588	13.25	17.9838	27.75
17.99	17.5	18.02	24.75
18.02	18	18.0488	25.25
18.055	17.25	18.0788	28
18.0863	16.75	18.1113	22.25
18.12	17.5	18.1463	22.75
18.1538	19.25	18.1763	22.5
18.1913	20	18.215	26
18.225	18.75	18.25	25.75
18.255	19.5	18.2825	24.5

18.29	14.25	18.315	23.25
18.32	12.5	18.3488	27.25
18.3513	15.5	18.38	26.5
18.3913	16.25	18.4163	26.5
18.4225	14.25	18.4488	21.75
18.4588	15.25	18.4838	27.5
18.49	16.25	18.5188	21.25
18.52	14.5	18.5488	21.75
18.555	13.75	18.5788	24.5
18.5863	14.5	18.6113	26.75
18.62	15.5	18.6463	22.25
18.6538	18	18.6763	27
18.6913	15.25	18.715	25.25
18.725	12.25	18.75	28
18.755	13	18.7825	19.75
18.79	14.75	18.8138	27.25
18.82	18.75	18.8488	23.25
18.8513	20.25	18.88	24
18.8888	16.25	18.9163	24.75
18.9225	12.5	18.9488	25.75
18.9588	14	18.9838	23.5
18.99	13.25	19.0188	27
19.02	17	19.0488	24
19.055	13.5	19.0788	28
19.0888	10.75	19.1125	26.5
19.12	14.25	19.1463	24
19.1538	14.5	19.1763	24.5
19.1913	15.75	19.2175	25.75
19.225	13	19.2525	31.25
19.255	15.75	19.2838	25.75
19.2888	13.5	19.315	27
19.32	17.75	19.35	29.75
19.3513	12.25	19.3838	26.25
19.3888	16.5	19.4175	27.5
19.4238	15.5	19.45	27
19.4588	15	19.485	31
19.49	13	19.5225	28.25
19.52	17.75	19.5513	25
19.555	17.25	19.5813	28.75
19.5888	20.75	19.6138	29.75
19.62	17.5	19.6488	25.5
19.6538	18.75	19.6788	31.5
19.6913	14.25	19.715	33
19.725	16.75	19.75	30.5
19.755	14	19.7838	27.75
19.7888	15	19.815	33.5
19.82	14.25	19.85	29.5
19.8513	17	19.8838	36.5
19.8888	18.25	19.9175	33.75
19.9225	15.75	19.95	30.25

19.9588	14.75	19.985	38.5
19.99	12.25	20.0213	31
20.02	15.5	20.0513	35.25
20.055	10.75	20.0813	33.25
20.0888	9.5	20.1138	35.5
20.12	9.25	20.1488	31.5
20.1538	11.25	20.1788	32.25
20.1913	11	20.215	38.5
20.225	16.75	20.25	36.5
20.2563	17	20.2838	35.75
20.29	17.5	20.3163	36
20.3225	12.5	20.35	35
20.3538	17	20.3838	37
20.39	15.5	20.4175	33.5
20.4275	14.25	20.45	37.75
20.4613	17.5	20.485	33.75
20.4925	11.75	20.5213	32.25
20.5225	15.25	20.5513	30
20.555	13.75	20.5813	35.25
20.59	13.25	20.615	31.25
20.6213	12.5	20.6488	30.5
20.655	14	20.6788	31.5
20.6938	14.75	20.715	34.25
20.7263	14.25	20.75	33.25
20.7563	12.25	20.7838	29.25
20.79	15.25	20.815	34
20.8225	15	20.85	31.25
20.8538	11.5	20.8838	33.25
20.89	10.75	20.9175	30.5
20.9275	13.25	20.95	34.5
20.9613	13	20.985	29.5
20.9925	9.25	21.0213	32.5
21.0225	10.5	21.0513	33.5
21.055	8.25	21.0813	31
21.09	13	21.115	30
21.1213	13.25	21.1488	32.5
21.155	13.75	21.1788	24.5
21.1938	11.5	21.215	30.5
21.2263	12.5	21.25	29.75
21.2563	13.25	21.2863	29.25
21.29	11.5	21.315	29.5
21.3225	12.5	21.35	27.25
21.3538	10.25	21.385	29.75
21.39	12	21.4175	25
21.4275	14.75	21.45	26.75
21.4613	14.5	21.4825	26.75
21.4925	15.25	21.5188	29
21.5225	14.75	21.5513	27.25
21.555	13	21.5813	28.5
21.59	12.75	21.615	31.5

21.6213	13.5	21.6488	30.75
21.655	13	21.6813	32.75
21.6938	14.5	21.715	28.5
21.7263	14	21.75	32
21.7563	14.5	21.7838	30.25
21.79	16.75	21.815	30.25
21.8213	13	21.8475	33.5
21.8538	15.5	21.885	29.5
21.89	14.5	21.9175	28
21.9275	14.75	21.9525	28.5
21.9613	16.25	21.9825	31.75
21.9925	15.25	22.0188	27.75
22.0225	16.75	22.0513	25.75
22.055	14	22.0813	30
22.09	19	22.115	26
22.1213	14.25	22.1488	26.75
22.155	15.75	22.1813	27.5
22.1938	12	22.215	27.5
22.2263	12.25	22.25	24
22.2563	14.75	22.2838	23.75
22.29	12.75	22.315	24
22.3213	15	22.3475	24.5
22.3538	17.25	22.385	26.75
22.39	15.25	22.4175	24.75
22.4275	13.25	22.45	27.75
22.4613	19.25	22.4825	31
22.4925	16.75	22.5213	21.25
22.5225	13.25	22.5513	29.25
22.555	16.5	22.5813	29.25
22.59	18.5	22.615	27.75
22.6213	18	22.6488	30.5
22.655	15	22.6813	35.5
22.6938	21.5	22.715	30.75
22.7263	17.5	22.75	31.25
22.7563	14	22.7838	28.75
22.79	16.75	22.815	28
22.8213	16	22.8475	28.5
22.8538	16.25	22.885	24.75
22.89	14.25	22.9175	28.5
22.9275	17.25	22.95	29.75
22.9613	13.75	22.98	25.5
22.9925	16	23.0188	25.5
23.0225	17.75	23.0513	24.25
23.055	17.5	23.0813	29
23.0875	16	23.1175	30.5
23.1213	18.5	23.1488	32.5
23.155	18.75	23.1813	30.25
23.1938	19	23.215	30
23.2275	19.25	23.2488	28
23.2563	11.25	23.2825	26.75

23.29	15.5	23.315	28.25
23.3213	14.25	23.3475	29
23.3538	15.75	23.385	30.5
23.39	11.75	23.4175	32.25
23.4275	13.5	23.45	33
23.4613	15.5	23.48	29.5
23.4925	10.75	23.5188	35.75
23.5225	12.5	23.5525	28.5
23.555	17	23.5813	31
23.5875	18.5	23.615	31
23.6213	18	23.6488	35.25
23.655	22.25	23.6813	30.5
23.6938	20.75	23.7175	31
23.7263	18.5	23.7488	30.5
23.7563	20.25	23.7825	32
23.79	21.5	23.815	28.75
23.8225	19	23.8475	24.75
23.8538	20.75	23.885	24.25
23.89	16.5	23.9175	23.5
23.925	18.25	23.95	26
23.9588	19.75	23.98	23.25
23.9925	23.25	24.0188	31
24.0225	20.75	24.0513	28.75
24.055	17.5	24.0813	30.25
24.0875	20	24.115	26.5
24.1213	20	24.1488	32.75
24.155	20	24.1813	33
24.1938	19	24.215	23.75
24.2263	18.25	24.2513	29.25
24.2563	14.75	24.2825	28.25
24.29	19.5	24.315	28.25
24.3225	19.25	24.3475	28.5
24.3538	19.25	24.385	30.5
24.3888	20.75	24.4175	29.25
24.425	17	24.45	25.25
24.4588	18.25	24.48	28.75
24.4925	19.75	24.5188	26
24.5225	22	24.5513	26
24.555	17.25	24.5813	29.5
24.5875	18.75	24.615	27.75
24.6213	20.25	24.6488	28.75
24.655	19.25	24.6813	29.5
24.6938	16	24.715	23.25
24.7263	18.75	24.7475	28.5
24.7563	16.25	24.7825	27.5
24.79	17.5	24.815	28
24.8225	18.75	24.8475	29.5
24.8525	17	24.885	31.5
24.8888	19.25	24.9175	27
24.925	18	24.95	23.5

24.9588	16.25	24.98	21.5
24.9925	17.25	25.0188	24.5
25.0225	16	25.0488	25.25
25.055	17	25.0813	23.5
25.0875	17.5	25.115	26
25.1213	13.75	25.1488	28.5
25.155	14.25	25.1813	26
25.1938	17.5	25.215	29.75
25.2263	16.5	25.2475	23.5
25.2563	18.75	25.2825	30.25
25.29	18	25.315	25
25.3225	11.75	25.3475	28.75
25.3525	16.25	25.385	29
25.3888	15.25	25.4175	23.75
25.4263	15	25.45	26.25
25.4613	15.75	25.48	23.25
25.4925	10.5	25.5188	26.75
25.5225	14.5	25.5488	25.75
25.555	17.25	25.5813	26.5
25.5875	19.25	25.615	24.75
25.6188	19	25.6488	24.75
25.655	17.25	25.6838	22.25
25.6938	14.25	25.7163	26.5
25.7263	17.5	25.75	25.75
25.7563	14.75	25.785	23.5
25.79	14	25.8163	27.5
25.8225	19.5	25.8488	21.25
25.8525	17.5	25.8863	23.5
25.8888	13.75	25.9188	20.25
25.925	14.25	25.9525	28
25.9588	13.5	25.9825	23
25.9925	15.25	26.02	28
26.0225	14.25	26.0513	24.75
26.055	16.5	26.0838	27.5
26.0875	11.25	26.1175	23.5
26.1188	17	26.1513	24.75
26.155	14.75	26.1838	26.5
26.1925	15.75	26.2163	26.25
26.2263	12.75	26.25	29.75
26.2563	14	26.285	31.5
26.29	16.5	26.3163	29.25
26.3225	19.25	26.3513	35.25
26.3525	14.75	26.3863	28.75
26.3888	15.25	26.42	25.75
26.425	17.75	26.4525	24.5
26.4588	17.5	26.4825	21.75
26.4925	15.75	26.52	23.5
26.5225	13.5	26.5513	22.75
26.555	15	26.5838	19.75
26.5875	11	26.6163	24.75

26.62	13.75	26.6513	28
26.655	15.25	26.6838	24.75
26.6925	12	26.7163	26.25
26.7263	12.75	26.75	25.75
26.7563	16.25	26.785	25
26.79	16.25	26.8163	26
26.8225	15	26.8488	30.75
26.8525	16.25	26.8863	27.5
26.8875	16.25	26.92	25.5
26.925	22.25	26.9538	28.75
26.9588	18.75	26.9825	32
26.9925	18.75	27.02	26
27.0225	15.5	27.0513	26.5
27.055	20	27.0838	27.25
27.0875	17.5	27.1163	28
27.1188	20.25	27.1513	29.25
27.155	16.25	27.1838	29.75
27.1925	17.5	27.2163	28.5
27.2263	15	27.25	31.75
27.2563	19	27.285	29.75
27.29	21.25	27.3163	28.5
27.3225	15.5	27.3513	26
27.3525	17.75	27.3863	30.25
27.3875	15.25	27.42	25
27.425	16	27.4525	25.25
27.4588	19.5	27.4825	23.75
27.4925	16	27.52	27
27.5225	16.5	27.5513	24.5
27.555	15.75	27.5838	29.25
27.5875	19.25	27.6163	25.5
27.6188	15.5	27.6513	26.75
27.655	18	27.6838	28.25
27.6925	15.5	27.7163	26
27.7263	13.5	27.75	25.5
27.7563	20.75	27.785	24.25
27.79	18	27.815	23.5
27.8225	19.25	27.8488	26.75
27.8525	16	27.8863	25.75
27.8875	19.25	27.92	29
27.925	14.75	27.9525	28
27.9588	17	27.9825	23.75
27.9925	15.75	28.0175	27.75
28.0225	16.5	28.0513	24.5
28.055	17	28.0838	29
28.0875	12.75	28.1188	32.25
28.1188	17.5	28.1513	26.5
28.1525	14.75	28.1838	28
28.1925	16.25	28.2163	30.75
28.2263	19.75	28.25	31.25
28.2563	14.75	28.285	27.75

28.29	15.75	28.315	29.25
28.3225	18.25	28.3488	29.5
28.3525	18.25	28.3863	28.25
28.3875	18.25	28.42	25.25
28.425	18	28.4525	28
28.4588	18	28.4825	30.5
28.4925	14.75	28.5175	30
28.5225	16.5	28.5513	31
28.555	17.75	28.5838	28
28.5875	18	28.6188	29
28.6188	14	28.6513	27.5
28.65	14	28.6863	27.5
28.6925	11.25	28.7163	32.5
28.7263	19.75	28.75	26.25
28.7563	18.25	28.785	30.5
28.79	18.5	28.815	25.75
28.8225	18.25	28.8488	29
28.8525	17.75	28.8863	29
28.8875	15.75	28.92	30
28.925	18	28.9525	33
28.9588	17.25	28.9825	31.25
28.9925	21.5	29.0175	27.5
29.0225	17.5	29.0513	26
29.055	15	29.0838	30.75
29.0875	13.25	29.1188	25
29.1188	17.75	29.1513	34.25
29.15	15.25	29.1863	27.25
29.1925	17.25	29.2163	29.25
29.2263	18.75	29.2475	28.5
29.2563	18	29.285	27.5
29.29	15.25	29.315	31.5
29.3225	19	29.3488	28
29.3525	16.25	29.3863	30
29.3875	22	29.4225	35
29.425	17.75	29.4525	23.75
29.4588	17.25	29.4825	27.5
29.4925	17.25	29.5175	24
29.5225	12.5	29.5513	34
29.555	15.75	29.5875	31.25
29.5875	16.25	29.6188	31.75
29.6188	12	29.6513	28.75
29.65	11.75	29.6838	26.5
29.6925	16	29.7163	25.25
29.7263	16.5	29.7475	26.25
29.7563	14.75	29.785	25.25
29.79	18.25	29.8175	23.5
29.8225	19	29.8488	27.75
29.8525	16	29.8863	28.25
29.8875	20.5	29.92	28
29.9225	16.75	29.9525	26.25

29.9588	16.5	29.9825	34.25
29.9938	17.5	30.0175	25.75
30.0238	13.75	30.0513	31
30.055	14.75	30.0838	25.5
30.0875	15.75	30.1188	31.25
30.1188	17.75	30.1513	30.75
30.15	12.75	30.1838	29
30.1925	16.25	30.2163	32.75
30.2263	16	30.2475	26.25
30.2563	15.75	30.2838	34
30.29	15	30.315	30.75
30.3225	13.25	30.3488	32.25
30.3525	17	30.3863	26.25
30.3875	16.25	30.42	23.5
30.4225	19.75	30.4525	27.5
30.4563	17.25	30.4825	25
30.4925	16.5	30.5175	30.75
30.5225	16	30.5513	29
30.555	17	30.5838	31.25
30.5875	18	30.6188	28
30.6188	16.5	30.6513	27.25
30.65	18	30.6838	34.75
30.6925	20.25	30.7163	28
30.7263	17.75	30.7475	24
30.7563	14.25	30.7838	24.5
30.79	17	30.815	29
30.8225	18	30.8488	26.25
30.8525	16.5	30.8863	29.5
30.8875	15.75	30.92	29.25
30.9225	21.25	30.9525	25.75
30.9563	17.75	30.9825	26.75
30.9925	19.75	31.0175	31.75
31.0225	19.5	31.0513	25.25
31.055	18.5	31.0813	28.75
31.0875	22.5	31.1188	30
31.1188	21.25	31.1513	26.25
31.15	15.75	31.1838	28.25
31.1925	15	31.2163	25
31.2263	16.25	31.2475	32
31.2563	15	31.2838	25
31.29	17	31.315	27.5
31.3225	16.25	31.3488	28.75
31.3525	13.5	31.3863	24.75
31.3875	16	31.42	28.25
31.4225	14.5	31.4525	28.5
31.4563	11.75	31.4825	31.5
31.4925	17.75	31.5175	22.5
31.5225	16.25	31.5513	27.25
31.555	17	31.5813	30.75
31.5875	17.5	31.6188	28

31.6188	18.25	31.6513	27.75
31.65	20.75	31.6838	28.75
31.6925	20.5	31.7163	29
31.7263	19.75	31.7475	29.75
31.7563	19	31.7838	29.25
31.79	20.5	31.815	27.25
31.8225	17.5	31.8488	28.5
31.8525	12.75	31.8863	29.75
31.8875	15.75	31.92	25.75
31.9213	18.25	31.9525	28.25
31.9563	17	31.9825	26.5
31.9925	15	32.0175	24
32.0225	13.25	32.0513	21
32.055	16.5	32.0813	25.75
32.0875	18.25	32.1188	30.5
32.1188	17.5	32.1513	25.75
32.15	17.25	32.1838	24.75
32.1913	19.5	32.2163	23
32.2263	19.75	32.2475	23
32.2563	19	32.2838	27
32.29	18.75	32.315	21.75
32.3225	18.75	32.3488	19.25
32.3525	15	32.3863	19.5
32.3875	24.25	32.42	23.25
32.4213	20.25	32.4525	25.75
32.4563	16.5	32.4825	22
32.4925	21.5	32.5163	22
32.5225	20.5	32.5513	23.75
32.555	18.75	32.5813	26
32.5875	20.25	32.6188	26.5
32.6188	17.75	32.6513	25
32.65	20	32.6838	24
32.6913	17.75	32.7163	25.75
32.7263	16.5	32.7475	24
32.7563	18.5	32.785	24.5
32.79	13.75	32.815	25.75
32.8225	16.75	32.8488	25.5
32.8538	15.25	32.8863	22.75
32.8888	13.5	32.92	21.5
32.9238	15.75	32.9525	22.75
32.9575	15.25	32.9825	20.25
32.995	14.75	33.0163	25.5
33.025	16	33.0488	26
33.0575	16.75	33.0813	28.75
33.09	12.75	33.1188	26.25
33.1213	13.75	33.1513	22.5
33.1525	14.25	33.1838	24
33.195	16	33.2163	26.5
33.2288	14.25	33.2488	28
33.2588	14.5	33.2838	29

33.2913	15.5	33.315	28.5
33.3238	15.5	33.3488	29.25
33.3538	15.5	33.3863	27.5
33.3888	17	33.42	29.25
33.4238	17.25	33.4525	27.75
33.4575	14	33.4825	26
33.4938	20.5	33.5163	27.25
33.525	13.5	33.5488	31.5
33.5575	15.75	33.5813	29.5
33.59	17	33.6188	24.75
33.6213	17	33.6538	24
33.6525	17	33.6838	23
33.695	14.5	33.7163	25
33.7288	16.75	33.7488	27.5
33.7588	17.75	33.7838	28
33.7913	17.75	33.815	17.75
33.8238	16.25	33.8475	21.25
33.8538	15	33.8863	27
33.8888	13.75	33.92	30.5
33.9238	15.5	33.9525	22.5
33.9575	18.5	33.9825	24.25
33.9938	17	34.0163	25.75
34.025	16.5	34.0488	25.5
34.0575	17.75	34.0813	24.5
34.09	21	34.1188	24.75
34.1213	19.5	34.1513	28.25
34.1525	15.25	34.1838	24.75
34.195	20.75	34.2163	24
34.2288	18.5	34.2488	24.25
34.2588	22	34.2838	23.25
34.2913	19.25	34.315	24.5
34.3238	21.75	34.3475	24.25
34.3538	19.75	34.3863	23
34.3888	22	34.42	28.25
34.4238	19	34.4525	25.25
34.4575	20.5	34.4825	28.25
34.4938	14.5	34.5163	30.5
34.525	22.5	34.5488	27
34.5575	18	34.5813	24.25
34.59	22	34.6188	29.5
34.6213	20.5	34.6513	30.75
34.655	23.5	34.6838	28.5
34.695	21.5	34.7163	27
34.7288	18.5	34.7488	26.75
34.7588	19.75	34.7813	26.75
34.7913	21.25	34.815	25.25
34.8238	18.75	34.8525	31.25
34.8538	20.25	34.8888	22.75
34.8888	18.25	34.9225	23.5
34.9238	20.5	34.955	24.25

34.9575	20	34.985	23.25
34.9938	20.5	35.0175	20.5
35.025	18	35.05	24
35.0575	23	35.0825	22.75
35.09	22.25	35.1213	24.5
35.1213	19.75	35.1525	24.5
35.155	19.25	35.185	20.5
35.1925	19.25	35.2175	26.5
35.2288	19.75	35.2513	24.25
35.2588	19.25	35.2838	25.5
35.2913	16.25	35.3175	23
35.3238	19.75	35.3525	24
35.3538	15	35.3913	21.25
35.3888	17	35.4225	31.75
35.4238	16.5	35.455	28.25
35.4575	14.25	35.485	27
35.4938	15	35.5175	26.5
35.525	17.25	35.55	29.25
35.5575	19.5	35.5825	27.75
35.59	13.75	35.6188	27
35.6213	18	35.6525	24.75
35.655	19.5	35.685	24.25
35.6925	18.75	35.7175	27
35.7288	18.75	35.7513	28.75
35.7588	20.75	35.7838	21.75
35.7913	20.5	35.8175	26.75
35.8238	18	35.8525	26.5
35.8538	18.75	35.8888	27.75
35.89	19.75	35.9225	25
35.925	18.25	35.955	24
35.9588	19	35.985	22.5
35.995	19	36.0175	17.75
36.0263	15.75	36.05	21.75
36.06	18.25	36.0825	23.25
36.0925	21.75	36.1188	18.75
36.1238	16.75	36.1525	20.5
36.1575	17.75	36.1875	24.25
36.1975	18.25	36.2175	21.25
36.2313	16.25	36.2513	25.25
36.2613	17.75	36.285	24.25
36.2938	14.25	36.3163	25.25
36.325	19	36.3525	24
36.355	17.5	36.3888	19.25
36.39	16	36.4238	22.5
36.425	16.75	36.455	27.75
36.4588	17.5	36.4875	25
36.495	13.5	36.5175	23.5
36.5263	17.75	36.55	24.25
36.56	14.25	36.585	26.5
36.5925	16	36.6188	25

36.6238	14.5	36.6525	22.25
36.6575	13.25	36.685	17
36.6975	15.5	36.7175	23
36.7313	16	36.7513	23.25
36.7613	14	36.7838	25
36.7938	13	36.8163	24.5
36.825	15.5	36.8525	24
36.855	18.25	36.8888	28.25
36.89	17.5	36.9225	23.5
36.925	16	36.955	23.5
36.9588	20	36.9875	29.25
36.995	19.5	37.0175	34.25
37.0263	18.5	37.0475	28.5
37.06	19.25	37.0825	30.5
37.0925	17	37.1188	28
37.1238	15.25	37.1525	25
37.1575	15.5	37.1875	34
37.1975	15.25	37.2175	24.5
37.2313	14.5	37.2513	25.75
37.2613	14.5	37.2838	19.5
37.2938	18.5	37.3163	30
37.3238	15	37.3525	28.75
37.355	12.25	37.3875	27.75
37.39	15.5	37.4225	29.5
37.425	16.25	37.455	26
37.4588	14	37.4875	25.5
37.4963	14.75	37.5175	30.25
37.5263	17	37.5475	25.25
37.56	15.25	37.585	28
37.5925	18	37.6213	31.75
37.6238	17.75	37.655	30.5
37.6575	22.75	37.685	31.25
37.6975	15.25	37.7175	27.5
37.7313	18	37.7513	28.75
37.7613	20.75	37.7838	36.25
37.7938	20.5	37.8163	30.75
37.8238	17.75	37.8525	34.25
37.855	17.5	37.8875	32.5
37.89	20	37.9238	32
37.925	17.5	37.955	36.75
37.9588	18	37.9875	31.5
37.995	17.25	38.0175	31.25
38.0263	20.5	38.0475	27.25
38.06	18.75	38.085	27.5
38.0925	14	38.1188	32
38.1238	19	38.155	28
38.1575	16.5	38.185	30.25
38.1975	14.25	38.2175	26
38.2313	21	38.2513	28
38.2613	17.25	38.2838	36.75

38.2938	17.75	38.3163	30.25
38.3238	18.5	38.3525	32.75
38.355	16.25	38.39	32.5
38.39	15.5	38.4225	25
38.425	14.5	38.455	28.5
38.4588	14.75	38.4875	33.75
38.495	13.5	38.5175	29.5
38.5288	11.5	38.5475	30.25
38.56	16	38.5875	33.25
38.5925	17.25	38.6188	32.25
38.6238	14.5	38.6525	29.25
38.6575	17.5	38.685	31.25
38.6975	13.75	38.7175	32.75
38.73	17	38.7513	31.25
38.7613	19.25	38.7838	29.5
38.7938	15.5	38.815	31
38.8238	16.75	38.8525	29
38.855	21	38.8875	29.75
38.89	19	38.9225	29.75
38.925	21	38.955	27.5
38.9588	19.5	38.9875	32.75
38.995	14	39.0175	26.25
39.0263	18.25	39.0475	32
39.06	17	39.0875	28.5
39.0925	22.5	39.1188	34.75
39.1238	18.25	39.1525	31.75
39.1575	27	39.185	35.75
39.1975	16	39.2188	34.75
39.23	21	39.2513	34.75
39.2613	16	39.2838	28.25
39.2938	21	39.315	28
39.3238	18.25	39.3525	28.5
39.355	17	39.3875	28
39.39	18.75	39.4238	27.25
39.425	19.75	39.455	33
39.4588	13	39.4875	28
39.4963	14.25	39.5175	33
39.5263	16.25	39.5475	29.75
39.56	17	39.5875	30.5
39.5925	16.5	39.6188	33.75
39.6238	14	39.6525	32.25
39.6575	18.25	39.685	36
39.6975	17	39.7188	29.25
39.73	15.5	39.7513	32.25
39.7613	16	39.7838	31.5
39.7938	16.25	39.815	29.25
39.8238	15.5	39.8525	31.5
39.855	15.25	39.8875	44.75
39.8913	14.5	39.9225	35.75
39.925	14.5	39.955	31

39.9588	12.25	39.9875	34.25
39.995	10.5	40.0175	31
40.0263	12.25	40.0475	29.25
40.06	13.75	40.085	29
40.0925	11.75	40.12	26.25
40.1238	12.25	40.1525	29.5
40.1575	16.25	40.185	29.25
40.1975	12.75	40.2188	30.25
40.23	14	40.2513	29.25
40.2613	19.5	40.2838	24.25
40.2938	13.25	40.315	29
40.3238	18.75	40.3525	25.75
40.355	17	40.3875	32.25
40.3913	15	40.42	31.75
40.425	19	40.4563	33.25
40.4588	15	40.4875	32.25
40.4963	18	40.5175	30
40.5263	18.25	40.5475	27.75
40.56	21	40.5825	33.25
40.59	19.25	40.6188	32.75
40.6238	17.5	40.6525	28.5
40.6588	20.5	40.685	31.25
40.6975	17.25	40.7188	29.75
40.73	19.75	40.7513	31
40.7613	20.5	40.7838	29.5
40.7938	17.25	40.815	29.75
40.8238	16.5	40.8525	28
40.855	19.25	40.8863	28
40.8913	20	40.92	31.75
40.925	16.5	40.955	29
40.9588	19	40.9888	25
40.995	21	41.02	25.25
41.0263	15.5	41.05	20.25
41.06	18.75	41.0838	24.5
41.09	17.25	41.12	22
41.1238	18.5	41.155	24
41.1588	16.25	41.1875	25.5
41.1975	13.25	41.2238	23.5
41.23	15.75	41.2538	29.5
41.2613	17	41.285	26
41.2938	17	41.3188	27
41.3238	12.75	41.355	25.5
41.355	13.25	41.3913	28.25
41.3913	16.75	41.4213	24.75
41.425	13.75	41.4563	26
41.4588	16.75	41.4888	24.5
41.495	15.25	41.52	22.75
41.5263	16	41.5513	20.25
41.56	14.75	41.5838	23.25
41.59	13.25	41.62	24.25

41.6238	14.5	41.655	25.5
41.6588	13	41.6875	24
41.695	15	41.7213	28.25
41.73	14	41.7538	24
41.7613	13	41.785	23.75
41.7938	15	41.8188	27.75
41.8238	14	41.855	27.5
41.855	13	41.8888	28.25
41.8913	15	41.9213	26.75
41.925	15	41.9563	29
41.9588	16	41.9888	27.75
41.9925	15.25	42.02	29.75
42.0263	17.75	42.0513	29
42.06	19	42.0838	29.25
42.09	17.75	42.12	27.75
42.1238	18.25	42.155	30
42.1588	20.5	42.1863	25.75
42.195	14	42.2213	30.75
42.23	15.5	42.2538	25.5
42.2613	11.75	42.285	26
42.2938	13.75	42.3188	29.25
42.3238	12.25	42.355	26.25
42.355	13.25	42.3888	26
42.3913	17.25	42.4213	24.5
42.425	15.25	42.4588	29.75
42.4588	13.25	42.4888	24.25
42.4925	16.5	42.52	23
42.5263	13	42.5513	26.5
42.56	15.5	42.5838	26
42.59	16.75	42.62	27
42.6238	18.25	42.655	20.5
42.6588	14.25	42.6863	23.75
42.695	16.25	42.7213	24.25
42.73	12.25	42.7538	28.75
42.7613	13.5	42.785	26.25
42.7938	13	42.8188	23.75
42.8238	15.75	42.855	28.25
42.855	15.5	42.8888	22.5
42.8913	12.75	42.9213	27.5
42.925	15.25	42.9588	23
42.9613	14.25	42.9888	23.75
42.9925	14.5	43.02	28
43.0263	10.75	43.0538	28.5
43.06	15.25	43.0838	25.25
43.09	17	43.12	27.75
43.1238	15	43.155	28.75
43.1588	13.75	43.1863	24.5
43.195	11.25	43.2213	22.5
43.23	16.75	43.2563	31.75
43.2613	13.25	43.2875	27.75

43.2938	12.5	43.3213	30.25
43.3238	20.5	43.3538	26
43.355	19.5	43.3913	29.25
43.3913	17.5	43.4238	31.25
43.425	19.5	43.4575	29.25
43.4613	17.75	43.49	35
43.4925	17	43.5213	30
43.5263	16.75	43.5525	30.25
43.56	17.75	43.5863	27
43.59	16.25	43.6238	22
43.625	21	43.6563	26.5
43.6588	22	43.6888	26.75
43.695	17.5	43.7238	32.5
43.73	17.75	43.7563	27.5
43.7613	17.75	43.7875	27.75
43.7938	20	43.8213	29.25
43.8238	20	43.8563	32
43.8538	22.5	43.8913	29
43.8913	17.5	43.9238	30.5
43.925	20.5	43.9575	30.75
43.9613	18	43.99	28
43.9925	19.75	44.0213	26.75
44.0263	21	44.0525	25.75
44.0625	21.25	44.0863	32.25
44.09	18.5	44.1238	28
44.1275	17.75	44.155	28.5
44.1588	18.5	44.1888	26.25
44.195	19.5	44.2238	25.25
44.23	19.25	44.2588	24.5
44.2613	18.75	44.29	21
44.2938	19.5	44.3213	29.25
44.3238	20.5	44.3563	26.5
44.3538	18.75	44.3913	30
44.3913	18.75	44.4238	30.25
44.425	19.25	44.4575	27.25
44.4613	17.5	44.49	32.25
44.4925	23.25	44.5213	29.25
44.5263	17.25	44.5525	28
44.56	18	44.5863	28.25
44.59	19.25	44.6238	28.25
44.6275	19.75	44.655	28.25
44.6588	20.75	44.6888	22.25
44.695	18	44.7263	27.75
44.73	17	44.7563	29.5
44.76	17.5	44.79	25
44.7938	16.75	44.8213	31.25
44.8238	14.25	44.8563	27.5
44.8538	20	44.8913	22.5
44.8938	20.75	44.9238	27
44.925	20.5	44.9575	26.75

44.9613	19.75	44.99	25.75
44.9925	17.25	45.0213	28.75
45.0263	17.5	45.0525	25.5
45.06	21.25	45.0863	27
45.09	18.75	45.1238	30.5
45.1275	17.75	45.155	22.5
45.1588	18.5	45.1888	28
45.1963	15.25	45.2225	27
45.2325	18.5	45.2563	27.5
45.26	20.25	45.29	27.5
45.2938	17.75	45.3213	28.25
45.3238	18.25	45.3563	27
45.3538	17.5	45.3913	30.75
45.3913	18	45.4238	23
45.425	20	45.4575	27.5
45.4613	19	45.49	27
45.4925	18.5	45.5213	25
45.5263	12.25	45.5525	28
45.56	14	45.5863	25.75
45.59	15.75	45.6238	28.25
45.6275	14.25	45.6563	31.25
45.6588	16.75	45.6888	29.25
45.6963	17.75	45.7225	25.5
45.73	17.25	45.7563	33.25
45.76	15	45.79	32
45.7938	13.5	45.8213	29.75
45.8238	14.25	45.8563	29.25
45.8538	15.5	45.8913	30.25
45.8913	13.25	45.9238	30
45.925	14	45.9575	31.25
45.9613	11.5	45.9913	31.25
45.9925	16.75	46.0213	30.25
46.0263	14.5	46.0525	24.5
46.06	18.25	46.0875	27.25
46.09	20.25	46.1188	22
46.1275	21	46.155	32.5
46.1588	18.75	46.1888	26.75
46.1963	20.25	46.2225	24
46.2313	22.75	46.2563	30.25
46.26	20.5	46.29	29.75
46.2938	17.25	46.3213	27
46.3238	18	46.3563	29.25
46.3538	20.5	46.3938	24.75
46.3938	21.5	46.4213	27.25
46.425	15.75	46.4575	27.25
46.4613	18.25	46.4875	28
46.4925	20.5	46.5225	31
46.5238	16.75	46.5525	30.75
46.56	15.5	46.5875	28.25
46.59	16	46.6188	31

46.6275	17.75	46.6563	27
46.6588	17.5	46.6888	29.25
46.6963	19.5	46.7225	29.5
46.73	15	46.7563	24.75
46.76	17	46.79	32.25
46.7938	20.5	46.8213	35
46.8238	19.5	46.8563	52.75
46.8538	16	46.8913	33.75
46.8938	16.5		
46.9263	19.75		
46.9613	20.25	46.9875	36
46.9925	20.75		
47.0238	18.75	47.0525	33.25
47.06	18.5	47.0875	38.5
47.09	16.5	47.1188	32.5
47.125	16.5	47.155	32.75
47.1588	13	47.1888	50.25
47.1963	18.25	47.2225	47.25
47.23	17	47.2588	28.5
47.26	14.75	47.29	51
47.2938	18.5	47.3213	39.25
47.3238	15.5	47.3563	29.25
47.3538	15.25	47.3913	29.5
47.3938	13.5	47.4213	23
47.4288	12.75	47.4575	23.75
47.4613	14.25	47.4875	30.75
47.4925	14.25	47.5225	22.75
47.5238	14	47.5525	32.25
47.56	15.75	47.5875	33.75
47.59	15.5	47.6188	28.75
47.625	13.25	47.655	33.25
47.6588	16.75	47.6888	29
47.6988	13	47.7225	31.75
47.73	12.75	47.7563	34.75
47.76	12.5	47.79	28.75
47.7938	15.5	47.8213	28.25
47.8238	15.75	47.8575	28
47.8563	11.5	47.8913	37.5
47.8938	12.5	47.9213	32.5
47.9288	14.25	47.9575	35.5
47.9613	16.75	47.9875	32.75
47.9925	15.5	48.0225	28.5
48.0238	19	48.0525	33.75
48.06	16.25	48.0875	27.75
48.09	13.25	48.1188	25.25
48.125	13.25	48.155	27.25
48.1588	13.5	48.1888	28.5
48.1963	13.75	48.2225	23.25
48.23	11.75	48.255	24
48.26	11.75	48.29	30

48.2913	13.5	48.325	27
48.3238	11.5	48.3563	30.75
48.3563	16.75	48.3913	29.25
48.3938	11	48.4213	24.5
48.4288	13.25	48.4575	30.5
48.4613	14.5	48.4875	26
48.4925	18	48.5225	28.5
48.5238	17.25	48.5525	27.75
48.56	15.75	48.5875	29.75
48.59	17.25	48.6188	28
48.625	17.25	48.6563	25.75
48.6613	16	48.6888	31
48.6963	16.25	48.7225	30
48.7325	18.5	48.755	26.5
48.7625	18.5	48.79	31
48.7925	21.25	48.8238	30
48.825	14	48.8563	23.75
48.8575	14	48.8913	27.75
48.895	19.75	48.9213	27.75
48.93	13.75	48.9575	25.75
48.9625	18.25	48.9875	25
48.9938	16.5	49.0225	25
49.0263	16.75	49.0525	26.25
49.0625	15.5	49.0875	22.25
49.0925	15	49.1213	29.75
49.13	14	49.155	26
49.1613	17.5	49.1875	27
49.1988	16.5	49.2225	24.75
49.2325	18.25	49.255	23.75
49.2625	20.5	49.29	24.5
49.2925	17.5	49.3238	23.5
49.325	17.25	49.3563	20.5
49.3575	15	49.3913	22.75
49.395	17.25	49.4213	21
49.43	17.75	49.4575	24.5
49.4625	19.75	49.4875	24
49.4938	17.5	49.5225	25
49.5263	15.25	49.5525	23
49.5625	19	49.5875	23.5
49.5925	16.5	49.6188	26
49.63	17.5	49.655	24
49.6613	18.75	49.6875	25
49.6988	19.25	49.7225	27.25
49.7325	21.75	49.7575	27.75
49.7625	18.5	49.79	28.25
49.7925	20.25	49.8238	26
49.825	17.5	49.8575	23.75
49.8575	18	49.8925	26.5
49.895	16	49.9225	26
49.93	14.5	49.9588	27.25

49.9625	16	49.99	29.5
49.9938	14.75	50.025	22.25
50.0263	14.75	50.055	20.75
50.0613	16.75	50.09	24.25
50.0925	15.5	50.1213	24.5
50.13	16	50.1575	22.5
50.1613	16.5	50.19	28.5
50.1988	17.25	50.225	27.75
50.2325	16	50.2588	24.25
50.2625	20	50.2913	23.75
50.2925	15.25	50.325	28.25
50.325	18.5	50.3575	24.75
50.3575	17.25	50.3925	23
50.395	16.5	50.4225	19
50.43	17.25	50.4588	23.75
50.4625	17.75	50.49	22
50.4938	14.75	50.5225	26.5
50.5263	17.75	50.5575	24.25
50.5613	21.5	50.59	24.5
50.5938	20	50.6213	25.5
50.6325	21.25	50.6588	22.5
50.6613	18.5	50.69	21.25
50.7013	17.5	50.725	25.75
50.7325	17.5	50.7588	22
50.7625	14.75	50.7913	22.5
50.7925	13	50.825	26.25
50.8263	15.5	50.8575	27.25
50.8575	14.75	50.8925	25
50.895	13.5	50.9225	27
50.93	11.5	50.9588	22.75
50.9625	14.5	50.99	25.5
50.9938	15.5	51.025	24
51.0263	15.75	51.06	21.75
51.0613	19	51.0925	23.75
51.0938	19.5	51.1238	26.25
51.13	17.25	51.16	24
51.1613	17.25	51.1913	21
51.1988	17.25	51.2263	22.5
51.2325	20	51.26	24.25
51.2625	18	51.295	22.25
51.2925	17.75	51.3263	24
51.325	18.25	51.3588	20.25
51.3575	16.5	51.3938	22.5
51.395	17.5	51.425	19.75
51.43	14.5	51.4613	23.25
51.4625	14.75	51.4925	23.5
51.4938	18.25	51.525	21.5
51.5263	14.5	51.56	21.5
51.5613	18	51.5925	22.5
51.5938	16.25	51.6238	21.75

51.63	19	51.66	25.5
51.6613	14.75	51.6913	21.25
51.6988	19.25	51.7263	25.75
51.7325	17.25	51.76	29.25
51.7625	15.75	51.795	23.25
51.7925	16.5	51.8275	22.25
51.8225	18.75	51.8588	24.25
51.8575	17.75	51.8938	24.75
51.895	18	51.925	24
51.93	18	51.9613	27
51.9625	17.25	51.995	30.5
51.9938	16	52.0263	28.25
52.0288	18.25	52.06	26
52.0613	18.5	52.0925	34.75
52.0938	16.25	52.1238	33
52.13	17	52.16	29.75
52.1613	18.5	52.1913	31.75
52.2013	15	52.2263	30
52.2325	16.75	52.2625	30
52.2625	16.5	52.295	27.5
52.2925	17.75	52.3288	29
52.3225	18	52.3588	32.25
52.3575	16.75	52.3938	35.5
52.395	16.25	52.425	28
52.43	16	52.465	26.25
52.4625	19.75	52.4925	29.25
52.4938	16.75	52.5263	29.5
52.5263	17.75	52.56	28.5
52.5625	13.75	52.5925	25.5
52.5938	14.5	52.6238	26.25
52.63	16.25	52.66	28.75
52.6613	16.25	52.6913	28.5
52.6988	16.75	52.7263	28.75
52.7325	14.5	52.76	24.25
52.7625	15.5	52.7925	23.75
52.7925	15.25	52.8275	21.25
52.8225	17.75	52.8588	26.25
52.8575	15.5	52.8938	23.5
52.895	12.75	52.925	25
52.93	19.75	52.9638	24.5
52.9625	18.75	52.9925	25.75
52.9938	11.75	53.0263	24.5
53.0275	14.75	53.0625	26.5
53.0613	17	53.0925	21
53.0925	17.5	53.1238	21.5
53.13	15.75	53.16	22
53.1613	17	53.1913	25
53.1988	16	53.2263	18.5
53.2325	17.5	53.26	21.75
53.2625	18.5	53.2925	17.75

53.2925	18.25	53.3275	21.5
53.325	20.75	53.3588	19
53.3575	19.25	53.3938	18
53.3963	14.75	53.425	19
53.43	15	53.4613	24
53.4625	18.5	53.4925	23
53.4938	17.75	53.5263	20
53.5263	14.5	53.56	20.5
53.5613	15.75	53.595	20.75
53.5925	15.5	53.6238	22.5
53.63	13.5	53.66	21.5
53.6613	19	53.6913	20.75
53.6988	17.5	53.7263	18.25
53.7325	12.5	53.7625	22.5
53.7625	15.75	53.7925	24
53.7925	14.5	53.8275	21.5
53.825	13.5	53.8588	25.75
53.8575	16.75	53.8925	26
53.895	10.75	53.925	25.75
53.93	15.5	53.9625	26.5
53.9625	14.5	53.9925	28
53.9938	17.25	54.0263	28.5
54.0263	17.5	54.06	25.5
54.0613	20.25	54.0925	27.5
54.0925	15.25	54.1238	29
54.13	15	54.1613	28.5
54.1613	14.25	54.1913	30
54.1988	14	54.2263	25
54.2325	18.25	54.2625	25.25
54.2625	17.25	54.2925	23.75
54.2925	17.75	54.3275	25.25
54.325	16.75	54.3588	24.5
54.3575	18.5	54.3925	27
54.395	17	54.425	30.25
54.43	13.75	54.4613	27
54.4625	14.75	54.4925	25
54.4938	14.25	54.5263	24.25
54.5263	15.5	54.56	26.5
54.5613	15	54.5925	26.5
54.5925	14.5	54.6238	25.25
54.6313	14.75	54.66	26.75
54.6613	12	54.6913	26.5
54.6988	16.75	54.7263	29.75
54.7325	15.75	54.7625	26.75
54.7625	17.25	54.7925	33
54.7925	15	54.8275	31.25
54.825	14	54.8588	30.75
54.8575	12.75	54.8913	26.25
54.895	13.25	54.925	24.5
54.93	15.75	54.9613	29

54.9625	19.5	54.9925	28
54.9938	13	55.0263	26.5
55.0288	14.5	55.06	26
55.0613	14.5	55.0925	28
55.0925	14.5	55.1238	27
55.13	13.75	55.16	28.75
55.1613	18	55.1913	31.25
55.1988	17.25	55.2263	26.25
55.2325	17.5	55.2625	27.5
55.2625	14.25	55.2925	27
55.2925	19.5	55.3275	29.5
55.325	20.75	55.3588	30.75
55.355	17.75	55.3913	34.25
55.395	16.5	55.425	30.5
55.4275	18.5	55.4613	27.5
55.4625	17.75	55.4925	26.5
55.4938	15.75	55.5263	30.5
55.5263	20.25	55.56	29.75
55.5613	17	55.5925	28.75
55.5925	17	55.6238	28.25
55.63	14.75	55.66	30.5
55.6613	14.5	55.6913	26
55.6988	16.25	55.7263	25.25
55.7325	14.25	55.7625	28.25
55.7625	16.5	55.7925	29
55.7925	18.5	55.8275	28.25
55.8275	15.5	55.8588	30.75
55.855	17.75	55.8913	26.25
55.895	16.5	55.925	30.5
55.9275	18	55.9613	26.75
55.9625	14.75	55.9938	23.5
55.9938	15.75	56.0263	28
56.0263	11.75	56.0588	31.5
56.0613	9	56.0925	24
56.0925	10.5	56.1238	31.25
56.13	10	56.16	31
56.1613	12.5	56.1913	31.75
56.1988	13.5	56.2263	30.25
56.2325	12.75	56.2625	30
56.2625	6.5	56.2925	36
56.2925	14.75	56.3275	29.5
56.325	11.75	56.3588	30
56.355	15.5	56.3938	35.25
56.395	16.5	56.425	31.25
56.4275	17.75	56.4625	29.75
56.4625	15.5	56.4938	28.25
56.4938	16.25	56.5263	32
56.5263	18.25	56.56	31
56.5625	21	56.5925	30.5
56.5925	16.75	56.6238	28.5

56.63	12.75	56.66	38.5
56.6625	20.75	56.6913	31
56.7	17.5	56.7263	29.5
56.735	17.75	56.7625	25.5
56.765	18.25	56.7925	26.75
56.7963	14.25	56.8275	30.5
56.8263	15	56.8588	29.5
56.8575	15.25	56.8913	28
56.8975	13.25	56.925	28.25
56.93	14	56.9588	33.25
56.965	15.25	56.9938	29.25
56.995	14	57.0263	32.75
57.0275	16	57.0638	28.5
57.065	15	57.0925	30.25
57.095	15.5	57.1238	29.75
57.13	17	57.1613	22
57.1625	15	57.1913	25.5
57.2	16.25	57.2263	30.25
57.235	15.5	57.2625	28.5
57.265	17.5	57.2925	25
57.2963	15.25	57.3275	24.25
57.3263	19.75	57.3588	21.25
57.36	15.25	57.3913	25.75
57.3975	16.5	57.425	22
57.43	12	57.4563	26.75
57.465	15.75	57.4938	28
57.495	16.25	57.5263	25.75
57.5275	12.25	57.5613	21.75
57.565	12.5	57.5925	21
57.595	15	57.6238	25.25
57.63	15.25	57.66	24.25
57.6625	12.25	57.6913	23
57.6988	13.75	57.7288	21.25
57.735	13.75	57.765	18
57.765	14.75	57.7925	23.75
57.7963	13.75	57.8275	24.5
57.8263	16.25	57.8588	23.5
57.86	13.75	57.8913	20.75
57.8975	13	57.925	29
57.93	16.25	57.9563	27
57.965	15.25	57.9938	30.75
57.995	13.25	58.0263	29.25
58.0275	15.5	58.0625	30.5
58.065	16.25	58.0925	30.75
58.095	16	58.1238	26.5
58.13	15	58.16	24.75
58.1625	15	58.1913	24.5
58.1988	15.5	58.2288	31
58.235	13.25	58.2625	23.75
58.265	15.25	58.2925	23.25

58.2963	14.25	58.3275	24.25
58.3263	14.5	58.3613	25.5
58.36	16	58.3913	25.25
58.395	11.5	58.425	29
58.43	13.5	58.4575	28.5
58.465	12	58.4938	28.75
58.495	14.75	58.5263	29.75
58.5275	15.25	58.5613	28.25
58.565	16.5	58.595	28
58.595	16	58.6238	30.25
58.63	17.5	58.66	26.75
58.6625	18	58.6913	26.25
58.6988	17.5	58.7288	28
58.735	17.5	58.7625	25.75
58.765	12	58.7925	22.5
58.7963	18.5	58.8275	30.25
58.8263	14.25	58.8588	30.25
58.86	14.75	58.8913	31.25
58.895	12.25	58.925	25.5
58.93	11.5	58.9613	26.25
58.965	11.25	58.9938	29.75
58.995	16.25	59.0263	25.75
59.0275	14.5	59.0613	27
59.065	13.25	59.0925	24
59.095	14.25	59.1238	27
59.13	11.75	59.1575	25.5
59.1625	13	59.1913	22.75
59.1988	18	59.2288	21.5
59.235	15.5	59.2625	20
59.265	13	59.2925	20.25
59.2963	13.75	59.3275	23.25
59.3263	14	59.3588	23
59.36	12.75	59.3913	21.5
59.395	13.75	59.425	23.75
59.43	14.75	59.46	22.75
59.4625	10.75	59.4938	21.25
59.495	14.5	59.5263	24.25
59.5275	18	59.5625	27
59.565	16.75	59.595	27.5
59.595	15.5	59.625	26
59.63	17.25	59.6588	30.25
59.6625	16	59.695	28.5
59.6988	14.25	59.73	28
59.735	16.75	59.7638	28.5
59.7663	12	59.7938	25.75
59.7963	14.25	59.8288	32.25
59.8263	8.5	59.8613	26.75
59.86	13.25	59.8938	29.75
59.895	11.25	59.9275	29.75
59.93	11	59.9625	32.25

59.9625	11
59.995	10.75
60.03	12.75
60.065	12.5
60.095	11
60.13	12.5
60.1613	12.75
60.1988	9.5
60.235	11.25
60.265	10.75
60.2988	14.25
60.3263	12
60.36	10.25
60.395	11.5
60.4288	12
60.4625	10.5
60.495	12.5
60.53	15.75
60.565	13.5
60.595	10
60.63	11.75
60.6613	14.25
60.6988	13.75
60.735	11.75
60.765	12.25
60.7963	10.75
60.8263	15.5
60.86	13.75
60.895	11
60.9288	14.25
60.9625	11
60.995	12.25
61.03	10.5
61.065	10.75
61.0963	12
61.13	13.5
61.1613	10.75
61.1988	10.75
61.2325	15
61.265	14.25
61.2963	11
61.3263	12.5
61.36	11.75
61.395	13.5
61.4275	12.75
61.4625	10.5
61.495	10.25
61.53	12.25
61.565	11.25
61.5963	7.25

59.9988	29.75
60.0288	32.25
60.0638	29.5
60.095	28.75
60.125	31.5
60.1588	36.25
60.195	39
60.23	34.75
60.2638	40.75
60.2938	36.75
60.3288	29.5
60.3613	30.5
60.3938	27.25
60.4275	24
60.4625	35.25
60.4988	27.75
60.5288	24.5
60.5638	24.5
60.595	24
60.625	29
60.6588	29.5
60.6975	32
60.73	30.25
60.7638	30.5
60.795	32.25
60.8288	33
60.8625	26.5
60.8938	31.75
60.9275	28
60.9625	31.25
60.9988	32.5
61.0288	28
61.0638	30.5
61.095	26.25
61.125	31.75
61.1588	26.75
61.1975	34.25
61.23	26.75
61.2638	27
61.295	25.75
61.3288	32
61.3613	33
61.3938	32.25
61.4275	25
61.4625	28
61.4988	31.25
61.5288	29.25
61.5638	28.5
61.595	34

61.63	9.5	61.6588	29.75
61.6613	12.75		
61.6988	11.75		
61.7325	8.25	61.7638	46.5
61.765	10.5	61.795	52.25
61.7963	10		
61.8263	12.25	61.8613	39.75
61.86	8.5	61.8938	34.75
61.895	12.75	61.9275	36
61.9263	9.75	61.9625	26.75
61.9625	11.75	61.9988	48
61.995	12.5	62.0288	31
62.03	11.75	62.065	32.25
62.065	7.75	62.095	37.5
62.0963	9.75	62.125	25
62.13	8.5	62.1588	25.25
62.1613	11.75	62.1975	29
62.1988	9.5	62.2288	31
62.2325	10	62.265	24.75
62.265	13.5	62.295	35
62.2963	9.75	62.3288	28.25
62.3263	9.5	62.3613	34.25
62.36	12.75	62.3938	27.75
62.395	11.25	62.4313	41
62.4263	9.25		
62.4625	12.25	62.4988	43
62.495	9.75	62.5288	37.5
62.53	13.75	62.565	43.5
62.565	9.25	62.5975	29.25
62.5963	13.5	62.625	35.25
62.63	13	62.6588	36.25
62.6613	12	62.6975	38.25
62.6988	11.25		
62.7325	13.75	62.7638	34.25
62.765	9.5	62.795	37.75
62.7963	12	62.8288	48.25
62.8263	10.75	62.8613	34.75
62.86	10.5	62.8938	34.5
62.895	12	62.9288	34
62.9263	11.75	62.9625	30
62.9625	12	62.9988	39.25
62.9938	9.5	63.0288	27.25
63.03	12.25	63.065	37.25
63.065	12	63.095	41
63.0988	8.75	63.125	31
63.13	11.5	63.1588	31.5
63.1613	11.25	63.1975	27
63.1988	12.25	63.2288	30.5
63.2325	8.25	63.2638	26
63.265	12.75	63.295	32.75

63.2963	7.5	63.3288	28.75
63.3263	8.75	63.3613	27.75
63.36	8.75	63.3938	25
63.395	7.5	63.4288	30.5
63.4263	8	63.4625	23.5
63.4613	7.75	63.4988	32.5
63.4938	6.5	63.5313	32.25
63.53	6.75	63.565	30.25
63.565	8.5	63.595	30.25
63.5963	9	63.625	29.75
63.63	7.75	63.6613	31.25
63.6613	7	63.6975	29.5
63.6963	11.25	63.7288	31.75
63.7325	7	63.7638	25.5
63.7663	10	63.795	28
63.7963	10	63.8288	30
63.8288	12	63.8613	29.75
63.86	13.25	63.8938	24.75
63.8925	10	63.9275	22.75
63.9263	13	63.9625	23.75
63.9613	13.5	63.9988	25
63.9938	13.75	64.0313	21.5
64.03	11.25	64.065	25.5
64.065	15.5	64.095	22.5
64.0963	13.5	64.125	23.25
64.13	13.75	64.1613	20.75
64.1613	10.75	64.1975	28.75
64.1963	20.5	64.23	25
64.2325	11.75	64.2638	32.25
64.2663	14.75	64.295	29.75
64.2963	12	64.3313	28.25
64.3288	14.75	64.3613	29.5
64.36	13.25	64.3938	29.25
64.3925	12	64.4275	26
64.4263	12	64.4625	27.25
64.4613	11.25	64.4988	28.5
64.4938	12.25	64.5313	29.25
64.53	11.5	64.565	26
64.565	12.25	64.595	27.5
64.5963	14	64.625	25.5
64.63	14.25	64.6613	27.25
64.6613	10.75	64.6975	22.5
64.6963	14.5	64.73	28.5
64.7325	15.25	64.7638	22.75
64.7638	11.25	64.795	29.25
64.7963	12.25	64.8288	24
64.8288	12	64.8613	31.25
64.86	14.25	64.9	31.25
64.8925	8.5	64.9275	28
64.9263	11.75	64.9625	26.5

64.9613	11.25	64.9988	30
64.9938	12.5	65.0313	32
65.03	13.5	65.065	30
65.065	12.75	65.095	25
65.0963	12	65.125	26.5
65.13	15	65.1613	28
65.1613	13.25	65.1975	22.5
65.1963	12	65.23	24.75
65.2325	12.5	65.2638	25.5
65.2638	11.25	65.295	24.25
65.2963	15	65.3288	23.25
65.3288	16.75	65.3613	25
65.36	10.25	65.3963	26.75
65.3925	11.5	65.4275	26.5
65.4263	12	65.4625	28.5
65.4613	13.5	65.4963	23.25
65.4938	13.75	65.5325	24.5
65.53	11.5	65.5675	31
65.5663	12.75	65.5963	26
65.5963	13.75	65.6275	24
65.63	11.5	65.6625	26.5
65.6613	12.25	65.6988	26.25
65.6963	14	65.7325	26.5
65.7325	12	65.7663	24.25
65.7638	12.5	65.7975	27
65.7963	13.25	65.8313	24.5
65.8288	13.5	65.8638	22.25
65.86	16.5	65.9	21.75
65.8925	14.5	65.93	27.25
65.9263	12.25	65.965	26.5
65.96	18	65.9975	21.25
65.9938	12	66.0325	25.75
66.03	10.25	66.0688	25.25
66.0663	10.75	66.0963	26.25
66.0963	12.75	66.1288	26
66.13	10.75	66.1625	29.5
66.1613	11	66.1988	27.25
66.1963	12	66.2325	27
66.2325	10.5	66.2675	28.25
66.2638	9	66.2975	29.25
66.2963	9.25	66.3313	26.75
66.3288	8.75	66.3638	20.5
66.36	11	66.4	27.75
66.3925	11	66.43	27.75
66.4263	12	66.4675	24.75
66.46	17.5	66.4975	20.75
66.4938	11.75	66.5325	25.5
66.53	12.25	66.5663	23
66.5663	11.25	66.5963	23
66.5963	13.25	66.6288	23

66.63	13.5	66.6625	25.5
66.6613	10.75	66.6988	21.25
66.6963	10	66.7325	23.5
66.7325	12.75	66.7675	22.5
66.7638	13.75	66.8	21.75
66.7963	12.5	66.8313	20.5
66.8288	13.5	66.865	20.5
66.86	14	66.9	22.25
66.8925	14.25	66.93	25.25
66.9263	12.75	66.9675	23
66.96	12.5	66.9975	23.5
66.9938	10.25	67.0325	20.25
67.0288	11.5	67.0663	23
67.0663	15.75	67.0963	20.5
67.0963	12.25	67.1288	18.5
67.1288	13.5	67.1625	20.25
67.1613	12.25	67.1963	20
67.1963	11.75	67.2325	23.5
67.2325	10.5	67.2675	20.75
67.2638	10.5	67.2975	19.25
67.2963	10	67.3313	20.25
67.3288	12.75	67.3663	20.75
67.36	13	67.4	20.75
67.3925	10.75	67.43	20.75
67.4263	14	67.4675	21.5
67.46	15	67.4975	19.25
67.4963	12.5	67.5325	21.25
67.5288	16.5	67.5663	17.5
67.5663	16	67.5963	22
67.5963	16.75	67.6288	21.75
67.63	18.75	67.6638	21.75
67.6625	15	67.6963	19.75
67.6975	16	67.7325	22.5
67.735	17.25	67.7675	21.5
67.7663	18.75	67.7975	22.75
67.7975	16.75	67.8313	26
67.83	13	67.8663	26
67.8625	13.75	67.9	26.25
67.895	14	67.9313	25.5
67.9288	14.75	67.9675	22
67.9625	13.25	67.9975	26
67.9975	14.25	68.0325	23.25
68.0325	12.75	68.0663	26.75
68.0688	11.75	68.0963	25.75
68.0988	13.75	68.1288	23
68.13	11.5	68.1625	28
68.1625	12.75	68.1963	27
68.1975	15	68.2325	25.75
68.235	16	68.27	21.75
68.2663	14.25	68.2975	23.25

68.3	15	68.3313	23.25
68.33	19.75	68.3663	23
68.3625	17.5	68.4	24.5
68.395	14.5	68.4313	22.25
68.4288	12.25	68.4675	20.75
68.4625	14.25	68.4975	22.75
68.4975	12.5	68.5325	26.5
68.5325	11.5	68.5663	24.75
68.5688	14.25	68.5975	25.25
68.5988	16.5	68.6288	26.75
68.63	13	68.6625	23.75
68.6625	10.5	68.6975	22.5
68.6975	13.75	68.7325	24.25
68.735	12.5	68.7675	26.75
68.7663	10.25	68.8	27.25
68.8	9.75	68.8313	21.75
68.83	10.25	68.8663	24.75
68.8625	12.75	68.9	26.75
68.895	10	68.9313	26.25
68.9288	12	68.9675	21.5
68.9625	10.75	68.9975	25.75
68.9975	8.25	69.0325	21.5
69.0325	11.75	69.0663	21.75
69.0688	12	69.0975	21.75
69.0988	10.5	69.1288	20.5
69.13	9.5	69.1625	20.75
69.1625	7.5	69.1975	24.5
69.1975	12.25	69.2325	23.75
69.235	12.25	69.2675	26.75
69.2663	10.25	69.2975	19.5
69.3	12.25	69.3313	23.5
69.33	12.75	69.3663	23.25
69.365	12.75	69.4013	23.5
69.395	11.5	69.4313	18.25
69.4313	11.5	69.4675	21.25
69.4625	12.5	69.5	17.75
69.4975	11.75	69.5325	25.5
69.53	10.5	69.5663	22.25
69.5688	14	69.5975	24.25
69.5988	12	69.6288	16.5
69.63	14.5	69.6625	24
69.6625	11.5	69.6975	23
69.6975	12	69.7325	25.25
69.735	12	69.7663	23.5
69.7663	13.25	69.7975	21.75
69.8	11.5	69.8338	24.75
69.83	11	69.8663	21.5
69.8625	15	69.9	23.5
69.895	10.5	69.935	23.5
69.9288	12.25	69.965	20

69.9625	13	70	21
69.9975	12.25	70.0325	20.5
70.0313	12	70.0663	20.5
70.0688	13.5	70.0975	19.25
70.0988	11.75	70.1288	19
70.13	12.25	70.1625	22
70.1625	16	70.1975	22
70.1975	11.75	70.2325	23.25
70.2325	14.75	70.2663	27.25
70.2663	17	70.2975	30.25
70.3	19.75	70.335	22.75
70.33	13	70.3663	27
70.3625	16.25	70.4	23.5
70.395	13.25	70.4338	23
70.4288	12.75	70.465	26
70.4625	14.5	70.5	29.25
70.4975	10.75	70.5325	25.25
70.5313	13.5	70.5663	28.75
70.5688	14.5	70.5975	23.25
70.5988	16	70.6288	22
70.63	15.25	70.6625	25.5
70.6625	13.25	70.6975	22.75
70.6975	11.25	70.7325	24.25
70.7338	18	70.7663	28
70.7663	12.25	70.7975	22
70.8	11	70.8338	26.75
70.83	13.75	70.8663	23.75
70.8625	12	70.9013	22.25
70.895	13.5	70.9338	20.25
70.9288	12.75	70.965	25
70.9625	12.75	71	24.5
70.9975	11.75	71.0325	21
71.0313	10.25	71.0675	20.75
71.0688	13	71.0975	25.75
71.0988	11.75	71.1288	22.25
71.13	11.75	71.1625	23.25
71.1613	11.75	71.1975	23.5
71.1975	13.25	71.2325	22.5
71.2338	15	71.2663	24.25
71.2663	9.5	71.2975	24.75
71.3	13.5	71.3338	25.75
71.33	12.25	71.3663	24.75
71.3625	9.5	71.4	24.5
71.3925	14.75	71.4338	22.75
71.4288	13.25	71.465	27.75
71.4625	17.25	71.5025	24.75
71.4975	11.5	71.5325	25.5
71.5313	12.5	71.5675	19.5
71.5688	13.75	71.5975	28.75
71.5988	13.75	71.6288	25.25

71.63	12
71.6613	12.5
71.6975	15.25
71.7338	14
71.7663	16.75
71.8	15.25
71.83	12.75
71.8625	12.5
71.8925	13.75
71.93	14.75
71.9625	13.5
71.9975	18.75
72.0313	16
72.0688	16
72.0988	14.75
72.13	19.25
72.1638	18.25
72.1975	16.5
72.235	14.75
72.2663	16.75
72.3	14.5
72.33	15.25
72.3625	16.75
72.3925	15.25
72.4288	11.75
72.4625	15.75
72.4975	15.75
72.5313	16.25
72.5688	15
72.5988	16
72.63	13.25
72.6613	14.25
72.6975	14
72.7338	12.5
72.7688	10.5
72.8	15.5
72.83	13
72.8625	14.25
72.8925	16
72.9288	13.25
72.9625	13.75
72.9975	14.75
73.0313	13.25
73.0688	12.75
73.0988	12.75
73.13	14
73.1638	15.5
73.1975	16
73.2338	14.75
73.2688	17.25

71.6625	24.75
71.6975	25.75
71.7325	26
71.7663	25.25
71.7975	25
71.8338	26.75
71.8663	25
71.9	26.25
71.9338	27.5
71.965	28
72	27.75
72.0325	29.5
72.0675	25.25
72.0975	26
72.1288	22.5
72.1688	25
72.1975	24.75
72.2313	23
72.2675	34
72.3013	23.5
72.335	33.25
72.3675	27.75
72.405	29.75
72.435	29.25
72.4663	33
72.5025	28.25
72.535	29
72.57	29.75
72.6	30.5
72.6313	25
72.6688	25.75
72.7	25.25
72.7363	27.75
72.7675	28.5
72.8013	28.75
72.835	27.25
72.8675	26
72.9038	34.75
72.935	28.25
72.9663	28.5
73.0025	33.5
73.035	33
73.07	33.75
73.1	28.5
73.1313	36.75
73.1688	29
73.2	32
73.2363	28.25
73.2675	29.25
73.3013	31

73.2988	16.25	73.335	34.25
73.33	14.5	73.3675	32
73.3625	15.5	73.4038	28.25
73.3925	14.75	73.435	28.75
73.4288	14.75	73.4663	22.5
73.4625	16.5	73.5025	24.25
73.4975	13.5	73.535	23.75
73.5313	11.25	73.57	24.25
73.5688	14	73.6	26
73.5988	11.25	73.6313	24
73.63	14	73.6688	29.5
73.66	14.75	73.7	26.25
73.6988	13	73.7363	23.75
73.7338	15.25	73.7675	23
73.7688	14.25	73.8013	27.75
73.7988	19	73.835	26
73.83	13.5	73.8675	28.75
73.8625	11.25	73.9038	30.25
73.8925	14.5	73.935	27.5
73.9288	14.25	73.9663	28.75
73.965	13	74.0025	28.25
73.9975	13	74.0325	30
74.0313	14.5	74.07	28.75
74.0688	12.25	74.1	24.5
74.0988	13.5	74.1338	26.75
74.13	12.5	74.1688	26.5
74.16	13.25	74.2	21.5
74.1975	11.5	74.2363	24.25
74.2338	13.25	74.2675	24
74.2688	12	74.3038	26.25
74.2988	12.5	74.335	21
74.33	13	74.3675	24.25
74.3625	15.75	74.4038	21.25
74.3925	10.5	74.435	20.75
74.4288	10.5	74.4663	28
74.465	14.75	74.5025	23.5
74.4963	16	74.5375	21
74.5313	14	74.5725	22
74.5688	8.25	74.6025	21.75
74.5988	13	74.6363	27.75
74.63	11.75	74.67	25.25
74.66	13.75	74.7013	24
74.6975	10.75	74.7375	28.25
74.7338	10.25	74.7713	23.25
74.7688	11	74.805	30.5
74.7988	13	74.8363	26.25
74.83	15.5	74.8688	23
74.8625	17.75	74.905	27.5
74.8925	13.5	74.94	20.5
74.9263	14.5	74.9725	23.5

74.965	16.25	75.0025	25.25
74.9963	20	75.0375	21
75.0313	13.75	75.0725	27
75.0688	12.75	75.1025	23.25
75.0988	11.75	75.1363	30.25
75.13	12	75.17	25.25
75.16	14.75	75.2013	23.25
75.1975	15.75	75.2375	22
75.2338	16.25	75.2713	21.75
75.2688	15.5	75.305	23.75
75.3013	14.5	75.3363	18.75
75.3325	14.75	75.3688	26.5
75.365	11.75	75.405	21.75
75.395	11.75	75.4375	24.5
75.4313	11.5	75.4688	25
75.4675	11.5	75.5025	21.25
75.5013	11	75.5375	22
75.5338	13	75.5725	19.25
75.57	8.75	75.6025	23.25
75.6	12.5	75.6363	29.75
75.6313	11.75	75.67	24.75
75.6613	9	75.7013	21.5
75.6988	9	75.7375	26.5
75.735	10.25	75.7713	24.5
75.77	12.75	75.805	21.5
75.8013	12	75.8363	21.75
75.8325	11.25	75.8688	26.25
75.865	10.5	75.905	26.25
75.895	12.25	75.9375	24.25
75.9288	10	75.9688	20.25
75.9675	8	76.0025	26
76.0013	12.75	76.0375	27.25
76.0338	11.75	76.075	28.25
76.07	15.75	76.1025	24.5
76.1	10.75	76.1363	24
76.1313	11.25	76.17	23.25
76.1613	12	76.2013	22.25
76.1988	10.5	76.2375	26.5
76.235	10	76.2713	24.75
76.27	12.25	76.305	25.25
76.3013	12	76.3363	27.25
76.3325	12.5	76.3713	25
76.365	12	76.405	31.25
76.395	14	76.4375	27
76.4288	13	76.4688	25.75
76.4675	10.5	76.5025	35.5
76.5013	12	76.5375	26.5
76.5338	15.25	76.5738	27.25
76.57	12.5	76.6025	21
76.6	11.5	76.6363	18

76.6313	12.25	76.6725	23.25
76.6613	9.25	76.7013	14.5
76.6988	14.75	76.7375	17.5
76.735	11	76.77	21.75
76.77	11	76.8063	21.5
76.8013	13	76.8363	19.25
76.8325	11.25	76.8713	25.25
76.865	11.75	76.905	24.75
76.895	14.25	76.9375	24.25
76.9288	13.75	76.9688	27.25
76.9675	12.75	77.0025	23.75
77.0013	12.75	77.0375	24.5
77.0338	12.25	77.0725	23.5
77.07	10.5	77.1025	24.25
77.1	12.75	77.1363	22
77.1313	13	77.17	25
77.1613	9.75	77.2013	26
77.1988	11.25	77.2375	25.5
77.235	10.75	77.27	19
77.27	10.5	77.3038	22.5
77.3013	8.25	77.3363	26
77.3325	11.5	77.3713	27
77.365	12.75	77.405	30.5
77.395	11.25	77.4375	25.25
77.4288	7.75	77.4688	27.5
77.4688	9.5	77.5038	22.25
77.5013	10.25	77.5375	27.25
77.5338	11	77.5725	22.25
77.5713	10	77.6025	24.5
77.6	8.5	77.6388	25
77.6313	15	77.67	25.75
77.6613	12	77.7013	22.75
77.7013	11.75	77.7375	29.25
77.735	15	77.7713	24.5
77.77	13	77.8038	25.75
77.8013	14	77.8363	24
77.8325	13	77.8713	24.5
77.865	15.75	77.905	22.5
77.895	13.5	77.9375	23.5
77.9263	10.5	77.9688	24
77.9688	12.5	78.0038	23.75
78.0013	11	78.0375	21.25
78.0338	9	78.075	20.5
78.07	9	78.1025	22.75
78.1	11	78.1388	24.75
78.1313	13.5	78.17	23.5
78.1613	11	78.2013	21.25
78.1988	9.25	78.2388	23.75
78.235	12.75	78.27	26
78.27	11	78.3038	27

78.3013	10	78.3363	29.25
78.3325	11	78.3713	26
78.365	10.25	78.405	25.75
78.395	15	78.4375	28.5
78.4263	7.75	78.4688	25.75
78.4688	9.5	78.5038	32.75
78.5013	10.25	78.5375	28.75
78.5338	11.75	78.5725	26.75
78.57	8.5	78.6038	23.5
78.6	11	78.6388	27.5
78.6313	12	78.67	27.25
78.6613	9.25	78.7013	27
78.6988	13	78.7375	29.5
78.735	9.5	78.77	22
78.7688	8.75	78.8038	26
78.8013	11	78.8363	25.75
78.8325	12	78.8713	27
78.865	10.75	78.9075	23.25
78.895	8.25	78.9375	26.5
78.9275	12.75	78.9688	24.25
78.9688	8	79.0038	23.25
79.0013	10.25	79.0375	24.5
79.0338	10.5	79.0725	23
79.07	12.25	79.1038	24.75
79.1	12.25	79.1388	23.25
79.1313	12.75	79.17	22
79.1613	10	79.2013	26.25
79.1988	11.5	79.2375	25.25
79.2363	16	79.27	24.75
79.2675	13.75	79.3038	27.25
79.3013	12	79.3363	31.5
79.3325	13	79.3713	27.75
79.365	13.5	79.4075	26.25
79.395	12.5	79.4375	25.5
79.4275	11.5	79.4688	24.75
79.4688	10.75	79.5038	21.25
79.5013	13.75	79.5375	26.75
79.5338	15	79.5725	21.25
79.57	12.5	79.6038	22.25
79.6	9.25	79.6388	26
79.6313	13.75	79.67	21
79.6613	8.5	79.7013	22
79.6988	8.5	79.7375	23.25
79.7363	10.25	79.77	20.75
79.7675	10.75	79.8038	17.75
79.8013	11.25	79.8363	19.75
79.8325	13.25	79.8725	25.75
79.865	13.5	79.9075	24.25
79.895	13.25	79.9375	25.5
79.9275	9.25	79.9688	22.5

79.9713	14.25
80.0038	15.75
80.0363	13
80.0725	10
80.1025	13.25
80.1325	14
80.1625	11.75
80.2	11.25
80.2388	13.5
80.2688	13.25
80.3025	11.5
80.3338	12.75
80.3675	11.75
80.3975	10.25
80.4313	12.25
80.4713	9
80.5038	12.25
80.5363	13
80.5725	16
80.6025	12.75
80.6325	13.25
80.6625	11.75
80.7	14.5
80.7388	13.5
80.7688	12.25
80.8025	13
80.8338	11
80.8675	11.5
80.8975	13
80.9313	11.25
80.9713	11.25
81.0038	9.25
81.0363	11.5
81.0725	13.5
81.1025	16.5
81.1325	11.5
81.165	9.75
81.2	12.75
81.2388	10.5
81.2688	9.75
81.3025	10.25
81.3338	8
81.3675	11
81.3975	11.5
81.4313	12.75
81.4713	13.25
81.5038	13.75
81.5363	13.75
81.5725	13
81.6025	13

80.0063	21.75
80.0363	23.5
80.0738	23.25
80.1063	24.75
80.14	23.5
80.1713	22
80.2025	24.25
80.24	26.5
80.2725	28.5
80.3063	24
80.34	23.5
80.375	22
80.41	24.25
80.44	25.75
80.4738	28.75
80.5075	24
80.54	27.25
80.5763	20.75
80.6088	23
80.6425	23.25
80.6738	24.25
80.705	22.75
80.7413	21.25
80.7738	23.5
80.8088	27.25
80.8413	23.5
80.8763	23.75
80.9113	26
80.9413	27
80.975	21.75
81.0075	25.25
81.04	21.25
81.0788	22
81.1088	29.5
81.1425	21
81.1738	22.5
81.205	24
81.2413	22
81.2738	25.75
81.3088	22
81.3413	27
81.3763	24.25
81.4113	27.25
81.4413	21.5
81.4738	29.5
81.5075	29
81.54	25.75
81.5763	24.25
81.6113	27.5
81.6425	26.25

81.6325	12.25
81.665	11.75
81.7025	12
81.7388	10.75
81.7688	10.75
81.8025	10.5
81.8338	11.75
81.8675	11
81.8975	14.25
81.9313	12
81.9713	11.75
82.0038	12.75
82.0375	9.75
82.0725	11.25
82.1025	13.75
82.1325	13
82.165	10.75
82.2025	8.75
82.2363	11.75
82.2688	11.25
82.3025	10.5
82.3338	11
82.3675	10.5
82.3975	9.75
82.4313	14.5
82.4713	12
82.5038	12.5
82.535	10
82.5725	13.25
82.6025	11
82.6325	10.75
82.665	16
82.7013	13
82.7363	14
82.7688	13.5
82.8025	12.75
82.8338	8.5
82.8675	10.5
82.8975	14.5
82.9313	18.5
82.9713	12
83.0038	11.25
83.035	12
83.0725	11.75
83.1025	13.25
83.1325	11.5
83.1675	7.75
83.2013	10
83.2363	9.75
83.2688	5.25

81.6738	24.25
81.705	25.75
81.7413	26.25
81.7763	26.5
81.8088	23.75
81.8413	26.75
81.8763	25
81.9113	24
81.9413	33
81.9738	29.75
82.0075	27.75
82.04	30.75
82.0763	28.5
82.1113	30
82.1425	29.25
82.1738	25.75
82.205	30.75
82.2413	25
82.2738	26
82.3088	27.5
82.3413	27.5
82.3763	25
82.4113	22.75
82.4413	28.25
82.4738	25.5
82.5075	32.5
82.5388	26.75
82.5763	22
82.6113	25.75
82.6425	25.5
82.6763	23.25
82.705	25.5
82.7413	24.5
82.7738	17
82.8088	19.5
82.8413	22.5
82.8763	23.75
82.9113	21.25
82.9413	26.5
82.9738	27
83.0075	26.5
83.0388	23.75
83.0763	28.25
83.1113	26.5
83.1425	30.75
83.1738	25.75
83.205	27.5
83.2438	23
83.275	26.5
83.3088	30

83.3038	12	83.3413	26
83.3338	12.25	83.3763	21.25
83.3675	9.75	83.4113	29
83.3975	11.25	83.4413	28.5
83.4313	10.5	83.4738	32.25
83.4713	8	83.5075	24.75
83.5038	11.75	83.5388	23
83.535	10.75	83.5763	29.75
83.5725	9	83.6113	25.5
83.6025	9	83.6425	25
83.6325	11.25	83.6738	30.5
83.6675	8.5	83.705	25.5
83.7013	7.75	83.7413	25
83.7363	9	83.7738	29.5
83.7688	6.75	83.8088	26.5
83.8038	8	83.8438	25
83.8363	6.75	83.8763	26
83.8675	9	83.9113	26.25
83.9	8.5	83.9413	27.75
83.9313	9.5	83.9738	27
83.9713	10	84.0075	28
84.0025	8.75	84.0388	28
84.035	10.25	84.0763	25
84.0725	11.25	84.1113	26
84.1025	9.75	84.1425	30.5
84.1325	10.25	84.1738	26.25
84.1675	12.25	84.205	25.25
84.2013	14	84.2413	26.25
84.2363	11	84.2738	29
84.2688	12	84.31	26.5
84.3038	8.5	84.3438	27.5
84.3338	11.5	84.3763	33
84.3675	13.25	84.4113	28.25
84.4	6.5	84.4413	30.5
84.4313	8.75	84.4738	26.75
84.4713	12	84.5075	27.75
84.5025	12.25	84.5388	23.5
84.535	9.25	84.5763	24.75
84.5725	15.5	84.6113	26.25
84.6025	11.75	84.6425	26.25
84.6325	14.25	84.6738	21.75
84.6675	14	84.705	26.25
84.7013	15	84.7413	20.5
84.7363	11.5	84.7738	18.75
84.7688	13.25	84.81	26
84.8038	10.75	84.8438	25.75
84.8338	10.75	84.8763	26.5
84.8675	12.75	84.9113	25.25
84.9	10.5	84.9413	21.5
84.9338	7.5	84.9738	20.5

84.9713	12.25	85.0075	25.25
85.0025	11	85.0388	24.5
85.035	13.5	85.0763	23.75
85.0725	10.5	85.1138	26.75
85.1025	11.75	85.145	23.25
85.1325	12	85.1763	26.5
85.1675	9.25	85.2063	20.25
85.2013	10.75	85.2438	20.75
85.2363	13.5	85.275	26.25
85.2688	13.25	85.3088	21.75
85.3038	14.5	85.345	24.25
85.3338	13	85.3775	24
85.3675	13	85.4125	21.75
85.4	14.5	85.4438	22.5
85.4338	17	85.4763	26.25
85.4713	11.75	85.51	22
85.5025	12	85.5438	26.75
85.535	14.25	85.58	23.5
85.5725	12	85.6138	20
85.6025	10.5	85.645	21.75
85.6325	13	85.6763	22.25
85.6675	12	85.7063	23
85.7013	10.25	85.7425	28
85.7363	8.5	85.775	25
85.7688	11.5	85.8088	18.75
85.8038	12.75	85.845	23.75
85.8338	13.75	85.8775	25.25
85.8675	10.75	85.9125	21
85.9013	11.25	85.9438	26.5
85.9338	10.5	85.9763	27.5
85.9713	10.5	86.01	24.25
86.0025	15	86.045	23.25
86.035	14	86.0813	28
86.0725	11.75	86.1138	20
86.1025	9.75	86.145	22.75
86.1325	11	86.1763	23.5
86.1675	13	86.2063	23
86.2013	14.25	86.2425	22
86.2363	14	86.275	18.75
86.2663	11.5	86.3088	24
86.3013	13	86.345	23.25
86.3338	14.75	86.3775	25.75
86.3675	13.5	86.4125	24.75
86.4013	12.25	86.4438	22.75
86.4338	12	86.4763	22.25
86.4725	14.25	86.51	22.75
86.5025	12.25	86.5438	25.25
86.535	11.5	86.58	23
86.5725	14.75	86.6138	25.75
86.6025	11.75	86.645	23.75

86.6325	11	86.6788	24.75
86.6675	12.75	86.7063	25.25
86.7013	13.5	86.7425	22.5
86.7363	12.25	86.775	28.5
86.7663	11.25	86.8088	22
86.8013	15.25	86.845	25.5
86.8338	15.25	86.8775	22.25
86.8675	8.5	86.9125	27.5
86.9013	14.5	86.9438	25
86.9338	13.5	86.9763	24.25
86.9713	16.25	87.0075	28
87.0025	11.25	87.0438	28.25
87.035	12.75	87.08	27.25
87.0725	18.5	87.1138	27
87.1025	18	87.145	25.5
87.1325	17.5	87.1763	28
87.1675	14.25	87.2063	26.25
87.2013	13.75	87.2425	26.75
87.2363	15.75	87.275	21.25
87.2663	17.25	87.3113	26.25
87.3013	16.5	87.345	22
87.3338	16.25	87.3775	24.75
87.3675	11.25	87.4125	27
87.4013	14.5	87.4438	23.5
87.4338	13.5	87.4763	24
87.4713	13	87.5075	23
87.5025	14.25	87.5463	26.25
87.535	13	87.58	23
87.5725	17.25	87.6138	24.5
87.6025	14.25	87.645	23
87.6325	13.25	87.6763	22.75
87.6675	12.75	87.7063	25
87.7013	15.25	87.745	21.5
87.7363	15.75	87.775	21.5
87.7663	14.75	87.8113	25.75
87.8013	15.75	87.845	24.75
87.8338	18	87.8775	18.75
87.8675	17.75	87.9125	23.75
87.9013	13.5	87.9438	34
87.9338	11.5	87.9763	26
87.9713	13.5	88.0075	29.5
88.0025	17.25	88.0463	22.25
88.0375	11	88.08	25.75
88.0725	17.75	88.1138	27.25
88.1025	13.75	88.145	28.5
88.1325	14	88.1763	27.25
88.1675	20.25	88.2063	26.5
88.2013	18.5	88.2425	26.75
88.2363	14	88.275	23
88.2663	15	88.3113	29.5

88.3013	15.25	88.345	23.75
88.3338	14.5	88.3775	27.75
88.3675	15	88.4125	24.75
88.4013	13.5	88.4438	28
88.4338	14.5	88.4763	28.25
88.4713	13	88.5075	24
88.5025	14.25	88.5438	28.25
88.5375	14.25	88.5775	23.25
88.5725	16	88.615	28.75
88.6025	15.75	88.645	22.25
88.6325	18	88.6763	25.25
88.6675	13.25	88.7063	25
88.7025	15.25	88.7425	22.5
88.7363	16.5	88.775	25.25
88.7663	12.75	88.8113	23.25
88.8013	16	88.845	24.75
88.8338	14.75	88.8775	25.75
88.8675	10	88.9125	26
88.9013	15.75	88.9438	23.75
88.9338	18.25	88.9763	21.75
88.9713	16.25	89.0075	24.75
89.0025	14.5	89.0463	25.75
89.0363	12.25	89.0775	26.5
89.0725	13	89.1138	25.25
89.1025	17.25	89.145	21
89.1325	14.75	89.1763	22.5
89.1675	14	89.2063	22.75
89.2013	15.75	89.2425	21.25
89.2363	13.25	89.275	21.25
89.2663	16.75	89.3113	21.5
89.3013	14.25	89.345	26.75
89.3338	14.25	89.3775	21
89.3675	12	89.41	22.5
89.4013	11	89.4438	24.25
89.4338	18	89.4763	15.25
89.4713	13.5	89.5075	14
89.5025	17.25	89.545	24.75
89.5363	13.75	89.58	19
89.5725	15.25	89.6138	22.5
89.6025	13.75	89.645	19.5
89.635	17.5	89.6763	23.75
89.6688	12	89.7063	23.25
89.7013	10	89.7425	20.25
89.7363	13.5	89.775	18.75
89.7663	14.75	89.8113	24
89.8013	13.25	89.845	25.5
89.8338	15.5	89.8775	20.5
89.8675	14.25	89.91	25.75
89.9013	13.25	89.9438	26.25
89.9338	15	89.9763	23.25

89.9713	12.75	90.0075	24.5
90.0025	15.75	90.045	26.25
90.0363	17	90.0775	22.75
90.0725	14.5	90.1138	28.5
90.1025	17	90.145	24.5
90.135	10.75	90.1763	24.25
90.1688	14.5	90.2063	25.25
90.2025	15.75	90.2425	26.5
90.2363	14.25	90.2763	27
90.2663	13.5	90.3113	24.25
90.3013	15.25	90.345	26.25
90.3338	14.5	90.3775	24.5
90.3675	13.25	90.41	25.25
90.4013	15	90.4438	24.5
90.4338	18.75	90.4763	25
90.4713	16.5	90.5075	26
90.5025	14	90.545	26.5
90.5363	15.5	90.5775	20.75
90.5725	14.25	90.6138	21
90.6025	18.25	90.645	24.5
90.635	20.5	90.6763	21.5
90.6688	19	90.7063	20.5
90.7025	18.25	90.7425	19.75
90.7363	16	90.7763	25.5
90.7663	17.75	90.8113	28
90.8013	16.25	90.845	21.75
90.8338	17.75	90.8775	25.75
90.8675	14.25	90.91	27
90.9013	14.25	90.9438	26.5
90.9375	17.25	90.9763	25.75
90.9725	18	91.0075	23.25
91.0038	16.75	91.045	19
91.0388	17.75	91.0775	20.25
91.075	14	91.1138	20.75
91.105	15	91.145	23.25
91.1375	17.25	91.1763	23
91.1713	14.75	91.2063	24
91.205	16.5	91.2425	25.75
91.2388	15.25	91.2775	23.25
91.2688	12.25	91.3113	23
91.3025	19.75	91.345	17.5
91.335	14.5	91.3775	19.25
91.3688	13.75	91.41	25.75
91.4025	14	91.4438	22.5
91.4375	14.75	91.4763	23
91.4725	12.75	91.5075	19.75
91.5038	12.75	91.545	21.75
91.5388	11	91.5788	20
91.5725	11.5	91.6138	19.25
91.605	12.75	91.645	24

91.64	13	91.6763	19
91.6713	12.75	91.7063	20.25
91.705	16.25	91.7425	21
91.7388	10.75	91.7775	18
91.7688	14.25	91.8113	22.75
91.8025	13.75	91.845	21.25
91.835	14.25	91.8775	27
91.8688	13.5	91.91	23.5
91.9025	19.25	91.9438	23.5
91.9375	15	91.9763	23.25
91.9713	18.5	92.0088	25.5
92.0038	18.5	92.045	23.25
92.0388	12.5	92.0775	22
92.0725	13.75	92.1138	28.75
92.105	13.25	92.145	22
92.1375	12.75	92.1763	24.75
92.1725	10.25	92.2063	24.5
92.205	14	92.2425	25.25
92.2388	9	92.2775	26
92.2688	17.5	92.3113	24.25
92.3	13.75	92.345	27.75
92.335	16.25	92.3788	22
92.3688	13.5	92.41	26.25
92.4025	14.25	92.4438	26
92.4375	15.5	92.4763	24.25
92.4713	11	92.5088	23.75
92.5038	9.75	92.545	26.75
92.5388	11.5	92.5775	24.75
92.5725	13.75	92.6138	20
92.605	12.25	92.645	25.75
92.6375	13.75	92.6763	16
92.6713	12.75	92.7063	25.25
92.705	16.25	92.7425	23.25
92.7388	13	92.7775	21.5
92.7688	12.25	92.8113	23.75
92.8	11	92.845	23.25
92.835	10	92.8775	27.5
92.8688	10.5	92.91	27.25
92.9025	13	92.9438	27.75
92.9375	11.25	92.9775	23
92.9713	15.75	93.0088	23
93.0038	13.25	93.045	26.25
93.0388	19.5	93.08	23.75
93.0725	20	93.1138	21.25
93.105	11.5	93.1463	28.25
93.1375	14.25	93.1763	21.5
93.1713	13.5	93.2063	23.75
93.205	18	93.2425	26
93.2388	15.25	93.2775	25.75
93.2688	16	93.31	25.25

93.3	15.5	93.3438	23.25
93.335	10.5	93.38	24.25
93.3688	13.25	93.41	24.25
93.4025	13.25	93.4438	22.25
93.4375	14.5	93.4775	23.5
93.4713	13.25	93.5088	21.25
93.5038	12.75	93.545	24.75
93.5388	12.25	93.5775	23
93.5725	15.75	93.6138	23
93.605	11.5	93.645	24.75
93.6375	12.25	93.6763	21.25
93.6713	15	93.7063	20.5
93.705	13.75	93.745	16.75
93.7388	15.75	93.7775	18.75
93.7688	10.75	93.81	21.25
93.8	13.75	93.8438	22.5
93.835	15	93.8775	19
93.8688	17	93.91	23
93.9025	14.5	93.9438	21.25
93.9375	15.5	93.9775	21.25
93.9725	19.25	94.0088	18.75
94.0038	14	94.0425	22.75
94.04	12	94.0775	24.25
94.0725	15.5	94.1138	22.5
94.105	15	94.145	22
94.1375	11.75	94.1763	19.75
94.1713	18.75	94.2063	21.5
94.205	17	94.2463	22.25
94.2388	19.75	94.2788	27.5
94.2688	21.25	94.3113	26.25
94.3025	14	94.3463	24
94.335	14.5	94.3788	27.25
94.3688	15.75	94.4113	25.75
94.4025	16.5	94.445	25
94.4375	15	94.4813	18.25
94.4713	19.75	94.5113	25
94.5038	12.75	94.545	23.25
94.5388	10.75	94.5813	25
94.5725	15.5	94.6163	25.25
94.605	14.75	94.6475	25.5
94.6375	15.75	94.6788	27
94.67	12.25	94.7088	26.75
94.705	13	94.7463	26.75
94.7388	17	94.7788	27.5
94.7688	18	94.8113	28.75
94.8	13	94.8463	26
94.835	13.5	94.8788	28.75
94.8688	13.75	94.9113	26.5
94.9025	13.25	94.945	30
94.9375	12.75	94.9813	26.75

94.9713	12.5	95.0113	25.25
95.0038	17.25	95.045	32.5
95.04	14.25	95.08	31.5
95.0725	13.5	95.1163	24
95.105	13.75	95.1475	26.5
95.1388	12.25	95.1775	26.25
95.1725	15.25	95.2088	25.75
95.205	12.25	95.2463	29
95.2388	15	95.2788	23.5
95.2688	11.75	95.3113	24.5
95.3	12.5	95.3463	25.25
95.335	16.5	95.3788	23
95.3688	12	95.4113	21.5
95.4025	15.75	95.445	28.25
95.4375	15.75	95.4813	26.75
95.4713	16.75	95.5113	24.25
95.5038	14.5	95.545	28.5
95.5388	16.75	95.58	23
95.5725	18	95.6163	23.5
95.605	13.75	95.6475	24
95.6388	17.5	95.6775	22.75
95.6725	13.75	95.7088	28.5
95.705	18.25	95.7463	21.25
95.7388	14.75	95.7788	26.5
95.7688	14.75	95.8113	26.75
95.8	14.75	95.8463	28.25
95.835	18.5	95.8788	25.25
95.8688	20.5	95.9113	27
95.9025	13.75	95.945	25
95.9375	15.5	95.9813	26
95.9713	15.5	96.0138	25.75
96.0038	17.75	96.045	25.25
96.0388	15	96.0825	28.75
96.0725	18.75	96.1175	26.75
96.105	17.75	96.1488	24
96.1388	14.75	96.1788	23.5
96.1725	11	96.2113	23.5
96.205	12.25	96.2488	28
96.2388	10.5	96.2813	23.5
96.27	11	96.3138	27.25
96.3	11.5	96.35	27.75
96.335	13.75	96.3813	20.5
96.3688	11.25	96.4138	26
96.4025	15	96.4475	28.25
96.4375	10	96.4825	25.5
96.4713	16.25	96.515	22.75
96.5038	13.5	96.5463	25.5
96.5388	12.75	96.5825	24.75
96.5725	12	96.6175	23
96.605	14.25	96.6488	20.75

96.6388	18	96.6788	25
96.6725	12	96.7138	23
96.705	16.25	96.7488	28.5
96.7363	13.75	96.7813	25.75
96.7688	12.25	96.8138	24.25
96.8	13.25	96.85	27.25
96.835	13.25	96.8813	27.75
96.8713	12.5	96.9138	25.75
96.9025	16.25	96.9475	26.5
96.9375	13.5	96.9825	28.5
96.9713	21.5	97.015	24
97.0038	16.25	97.0463	20.75
97.0388	14.75	97.0825	19.75
97.07	15.25	97.1175	22.25
97.105	14.5	97.1488	25.75
97.1388	14.5	97.1788	29.75
97.1725	12.5	97.2163	26.25
97.205	13.75	97.2488	24.25
97.2363	12.5	97.2813	22.75
97.27	16.75	97.3138	21.75
97.3	13.75	97.35	27
97.3375	11.75	97.3813	22
97.3688	11.5	97.4138	24.25
97.4025	17.5	97.4488	23
97.4375	12.5	97.4838	20
97.4713	12.75	97.5175	25.25
97.5038	11	97.55	23.75
97.5388	15.5	97.5838	20
97.57	16.25	97.6188	22.5
97.605	11	97.6513	27.5
97.6388	11	97.6813	19
97.6725	9.5	97.7188	18.75
97.705	12	97.7513	21
97.7363	13.75	97.7825	18.75
97.77	13	97.8163	21.75
97.8	14.5	97.8525	23.5
97.835	8.75	97.8838	21.25
97.8688	9.75	97.915	21
97.9025	7.5	97.9488	19
97.9375	13.5	97.9838	22.75
97.9713	13.25	98.0175	24.5
98.0038	12.5	98.05	23.5
98.04	14.75	98.0838	26.75
98.07	14.75	98.1188	22.5
98.105	14	98.1513	26
98.1388	15.75	98.1813	25
98.1725	12.25	98.2188	22
98.205	13	98.2513	24.75
98.2388	17.5	98.2825	23
98.27	13.75	98.3175	26.25

98.3	11.75	98.3525	24.5
98.335	19	98.3838	22.75
98.3688	17	98.415	24.5
98.4025	15	98.4488	22
98.435	12.75	98.4838	24.25
98.4713	18.5	98.5175	26.75
98.5038	18	98.55	26.25
98.5388	15.75	98.5838	22.25
98.57	16.75	98.6188	23.75
98.605	18	98.6513	20
98.6388	18	98.6813	23.25
98.6725	17	98.7188	24
98.705	17.25	98.755	22
98.7375	19.25	98.7825	22.75
98.7725	18.25	98.8163	21.75
98.8025	13.75	98.8525	20.75
98.8363	13.25	98.8838	22.25
98.8713	17	98.915	21.5
98.9075	13.5	98.9513	21.75
98.9375	17.5	98.9838	18.25
98.9738	16	99.0175	20.75
99.0063	17	99.05	21.5
99.04	14	99.0838	21.5
99.0713	12	99.1188	22
99.1088	12.25	99.1513	23.5
99.14	10.25	99.1813	26
99.1738	12.5	99.2188	28.25
99.2063	13.75	99.2525	24.5
99.2375	11.5	99.2825	24.25
99.2725	12.25	99.3163	27.75
99.3025	12	99.3525	30.5
99.3363	13.5	99.3838	26.5
99.3713	11.5	99.415	25
99.4075	14	99.4513	25.75
99.4375	11.75	99.4838	26
99.4738	11	99.5175	24.25
99.5038	10.5	99.55	23
99.54	13.25	99.5838	24.5
99.5713	14	99.6188	27.25
99.6063	12	99.6513	25.5
99.64	10.5	99.6813	28
99.6738	13	99.7188	24.75
99.7063	9.25	99.7525	25.75
99.7375	11.75	99.7825	26.75
99.7725	8.25	99.8163	24
99.8025	12.25	99.85	25
99.8363	14	99.8838	25
99.8738	11.25	99.915	25.5
99.9075	14.25	99.9513	26.75
99.9375	12.75	99.9838	25

99.9738	11.25	100.0188	25.5
100.0038	15.5	100.05	27
100.04	12.25	100.0838	22.75
100.0713	13.5	100.1188	25
100.1063	13.75	100.1513	26.25
100.14	14.25	100.1825	20.25
100.1738	8	100.2188	25.25
100.2063	8.25	100.2525	23.5
100.2375	9.25	100.2825	23.25
100.2725	9.25	100.3163	24
100.3025	10.5	100.3475	21.75
100.3363	12	100.3838	21.25
100.3713	15.5	100.415	23
100.4075	8.75	100.4513	21.5
100.4375	17	100.4838	24.5
100.4738	15.75	100.5175	20.25
100.5063	15	100.55	20.25
100.54	11	100.5838	22.5
100.5713	11.5	100.6188	20.5
100.6063	13.75	100.6513	23
100.6425	14.5	100.6813	21.75
100.6738	11.75	100.7188	23.5
100.7038	12.75	100.7525	20.5
100.7375	15.25	100.7825	21.75
100.7725	16.75	100.8163	24.5
100.8025	11.25	100.8475	20
100.8363	15	100.8838	20.75
100.8713	13.75	100.915	20.5
100.9075	14.5	100.9513	29.25
100.9375	13.75	100.9838	22.5
100.9738	14.25	101.0175	21.5
101.0038	12.5	101.05	18.5
101.04	15.25	101.085	17
101.0713	10	101.1188	18.75
101.1063	11	101.1513	22
101.1425	10.5	101.1813	16
101.1738	8.25	101.2188	17.25
101.2038	11.5	101.2525	20
101.2375	15.25	101.2825	19
101.2725	9	101.3163	23
101.3025	12.5	101.3475	22
101.335	13.75	101.3838	20.5
101.3713	13	101.415	21.5
101.4075	12.5	101.4525	22
101.4375	13.5	101.4863	17.5
101.4738	15	101.5175	22
101.5038	16	101.55	17
101.54	16.75	101.5838	20.75
101.5713	11.75	101.6188	18.25
101.6063	14.25	101.6513	24.25

101.6425	15	101.6825	22.75
101.6738	19.5	101.7188	23
101.7038	13	101.7525	28
101.7375	15.25	101.7825	17.25
101.7713	15.5	101.8163	18.75
101.8025	11.5	101.8475	18.75
101.835	16.75	101.8838	16.5
101.8713	16.25	101.915	16.25
101.91	11.75	101.95	18.25
101.9375	16	101.9888	21.5
101.9738	12.75	102.0175	22.25
102.0038	13	102.05	22.5
102.04	15.5	102.0838	21
102.0713	13	102.1188	18
102.1063	10.75	102.1513	23.25
102.1425	10.75	102.1825	20.5
102.1738	12	102.2188	24
102.2038	10.75	102.2525	24
102.2375	9.25	102.2825	22.75
102.2713	10.75	102.3163	23.25
102.3025	11	102.3475	23.25
102.335	11.75	102.3838	23.75
102.3713	13.5	102.415	19.75
102.4075	14.25	102.45	22.75
102.4375	10.75	102.4863	22.25
102.4725	10	102.5175	20.75
102.5038	9.75	102.55	27.5
102.54	13.25	102.5838	22
102.5713	10	102.6188	24
102.6038	14.75	102.6513	19
102.6425	14.25	102.6825	19.5
102.6738	13.75	102.7188	19.5
102.7038	9.75	102.7525	21.75
102.7375	15.5	102.7825	23.5
102.7713	11.25	102.8163	24.25
102.8025	9.25	102.8475	21.75
102.835	14.5	102.8838	22.25
102.8713	10.75	102.915	27.25
102.9075	10.5	102.95	23.25
102.9375	11.5	102.9863	20.25
102.9725	12.25	103.0175	21.75
103.0038	9.75	103.05	22.5
103.04	9.25	103.085	23.5
103.0713	11	103.1175	18.75
103.1038	13	103.1513	22
103.1425	11.5	103.1825	25.25
103.1738	12.5	103.2188	22.5
103.2038	12	103.2525	25.5
103.2375	11.25	103.2825	26.75
103.2713	10.75	103.3163	26

103.3025	15	103.3475	22.25
103.335	12	103.3838	18.25
103.3713	17	103.415	24.25
103.4075	19.25	103.45	21
103.4375	14.25	103.4863	25.5
103.4725	15.5	103.5175	23.25
103.5038	15.25	103.55	23.25
103.54	16.25	103.5838	26.25
103.5713	18.5	103.6175	24
103.6038	19.25	103.6513	25
103.6425	17.75	103.6825	25.5
103.6738	14.75	103.7213	24
103.705	16.75	103.7525	23
103.7375	14.5	103.7825	22
103.7713	17.75	103.8163	20.25
103.8025	13	103.8475	22
103.835	13.75	103.8838	21.5
103.8738	15.5	103.915	23.75
103.9075	14	103.95	24.25
103.9375	12.5	103.9875	22.25
103.9725	14	104.0175	23
104.0038	15	104.05	25.75
104.0375	16.5	104.0838	24.5
104.0713	13.75	104.1175	27.75
104.1038	15	104.1513	22.5
104.1425	16	104.1825	24.25
104.1738	11	104.22	26.5
104.2038	13.5	104.2525	25.75
104.235	13.5	104.285	25.5
104.2713	15	104.3163	30.75
104.3025	11.75	104.3475	28.75
104.335	13	104.3838	28.75
104.3738	12.5	104.415	29.75
104.4075	15.75	104.45	30
104.4375	12	104.4863	24.5
104.4725	13.5	104.5175	22
104.5038	10.25	104.55	24.25
104.5375	10.75	104.5838	23.5
104.5713	12.5	104.6175	22.75
104.6038	11	104.6513	25.75
104.6425	14.75	104.6825	24.5
104.6738	12	104.72	24.25
104.7038	11.5	104.755	26
104.735	16	104.785	26
104.7713	19	104.8163	26.5
104.8025	13.75	104.8475	21.75
104.835	14.75	104.8838	25.5
104.8725	13.25	104.915	28
104.9075	17.25	104.95	25.25
104.9375	15.25	104.9863	24.5

104.9725	15
105.0038	14
105.0375	19.5
105.0713	14.25
105.1038	9.5
105.1425	11.5
105.1738	14
105.2038	11.75
105.235	10
105.2713	12.25
105.3025	13.75
105.335	14.5
105.3725	15.75
105.41	14.25
105.4375	15.25
105.4725	10.75
105.5025	14.5
105.5375	11
105.5713	12.75
105.6038	12.5
105.6425	15.25
105.6738	13.5
105.7063	12.25
105.735	13.75
105.7713	12
105.8025	12.75
105.835	18.5
105.8738	14.25
105.9075	16
105.9375	15
105.9725	17.5
106.0025	17.5
106.0375	21.75
106.0713	17
106.1038	16
106.1425	20.25
106.1738	12.5
106.2038	11
106.235	13.5
106.2713	13.5
106.3025	12.25
106.335	14.75
106.3738	11.5
106.4075	11
106.4375	12.5
106.4725	12.5
106.5025	18
106.5375	10
106.5713	15.25
106.6038	14

105.0175	23.25
105.05	28.5
105.0838	25.75
105.1175	24
105.1513	22.75
105.185	26
105.22	20.75
105.2525	20.5
105.285	22.25
105.3163	27.75
105.3475	23.5
105.3838	22.25
105.415	22.25
105.45	21.25
105.4863	26.25
105.5175	21
105.55	25.5
105.5838	23
105.6175	21.25
105.6513	28.25
105.685	23.25
105.72	26.5
105.7525	27.5
105.7875	27.25
105.8163	27.5
105.8475	28.75
105.8838	27.75
105.915	27.25
105.9513	26.25
105.9863	22.5
106.0175	28.25
106.05	26.25
106.0838	25.75
106.1175	21
106.1513	25.75
106.185	24
106.22	23
106.2525	24.25
106.285	24.75
106.3163	23.25
106.3475	23.75
106.3813	19
106.415	20.5
106.4513	22.25
106.4838	22.5
106.5175	22.75
106.55	21.25
106.5838	20.5
106.6175	19.75
106.6513	17.25

106.6425	10.25	106.685	20.5
106.6738	13.25	106.72	19
106.7038	12.75	106.7525	19.5
106.735	13.75	106.785	21.25
106.7688	13	106.8163	23
106.8025	11.5	106.8475	23.25
106.835	11.25	106.8825	20.25
106.8738	10	106.915	20.75
106.9075	11.5	106.9513	19.75
106.9375	11.25	106.9838	17.75
106.9725	10.25	107.0175	20
107.005	10	107.05	16.25
107.0375	11	107.0838	18.5
107.0713	8	107.1175	23.5
107.105	12.25	107.1513	19.75
107.14	11.25	107.185	20.25
107.1738	12	107.22	22.25
107.2038	11.25	107.2525	18
107.235	12.25	107.285	19
107.2688	8.5	107.3163	19.5
107.3013	15.5	107.3475	15.25
107.335	12.25	107.3813	20.25
107.3738	11	107.415	17.75
107.4075	11	107.4513	18.25
107.4375	10.5	107.4838	22.75
107.4725	12.5	107.5175	19.75
107.505	9	107.55	22.5
107.5375	11	107.5838	17.75
107.5713	12	107.6175	21.25
107.6038	13.25	107.6513	26.5
107.64	10.25	107.685	22.25
107.6738	9.75	107.72	23.5
107.7038	10.75	107.7525	21.25
107.735	10.25	107.785	23.75
107.7688	13.5	107.8163	22.75
107.8013	14.25	107.8475	21.5
107.8375	12	107.8813	19.75
107.8738	13.75	107.915	20.25
107.9088	13.25	107.9513	23.25
107.94	11.75	107.9838	22.75
107.9725	14.25	108.0175	19
108.005	17.25	108.05	21.25
108.0375	16.5	108.0838	24.25
108.0713	15	108.1175	16.75
108.1038	16	108.1513	24.25
108.14	12.25	108.185	23.25
108.1738	13.5	108.22	18.75
108.2038	13.75	108.25	22.5
108.235	14.25	108.285	23.75
108.2688	11.25	108.3163	25.5

108.3013	12	108.3475	24.25
108.335	18.25	108.3813	23.25
108.3738	14	108.4163	24.75
108.4075	15.25	108.4513	21.5
108.4375	14	108.4838	24
108.4725	15.5	108.5175	21
108.505	16.25	108.55	25.75
108.5363	12.25	108.5838	28.5
108.5713	13.25	108.6175	23.5
108.6038	13.25	108.6513	22.75
108.64	17.25	108.6875	24.5
108.6738	15	108.72	24.5
108.7038	11.75	108.75	23
108.735	12.5	108.785	24
108.7688	13.25	108.8163	24.25
108.8013	16.75	108.8475	22.75
108.835	14	108.8813	21.5
108.8738	16	108.9163	25.75
108.9075	12.25	108.9513	23.25
108.9375	14.5	108.9838	24.5
108.9725	11.75	109.0188	25.75
109.005	15.25	109.05	27.25
109.0363	16.75	109.0838	25.25
109.0713	16.25	109.1175	26.25
109.1063	13	109.1513	27.75
109.14	15.5	109.1875	22.75
109.1738	13.75	109.22	26
109.2038	13.25	109.25	26.5
109.235	13	109.285	24
109.2688	14.25	109.3163	25.5
109.3013	11.25	109.3475	22.5
109.335	14.75	109.3813	23.75
109.3738	15.5	109.4163	25.75
109.4063	11.75	109.4513	27
109.4375	14.75	109.4838	24.75
109.4725	12.75	109.5188	27.75
109.505	13.5	109.55	25.75
109.5363	13.25	109.5838	23.5
109.5713	12	109.6175	26.75
109.6038	11.25	109.65	27
109.64	14	109.6875	23.25
109.6738	14.75	109.72	30.75
109.7038	11	109.75	25.25
109.735	11.25	109.785	25
109.7688	14.5	109.8175	22
109.7988	15	109.85	24
109.835	16.75	109.8813	23.25
109.8738	14.25	109.9163	23.5
109.9063	10.75	109.9513	24
109.9375	16	109.9838	23.25

109.9725	15.5	110.0188	24.5
110.005	13.5	110.05	19.75
110.0363	15	110.0838	24
110.0713	14.75	110.1175	22.75
110.1038	15.5	110.15	18.25
110.14	18.75	110.1875	23
110.1738	14	110.22	16.5
110.2038	15.75	110.25	20.25
110.235	16.5	110.285	19
110.2688	14	110.3163	20.5
110.2988	11.25	110.3475	19.75
110.335	12.25	110.3813	23.5
110.3738	12.75	110.4163	19.75
110.4063	14.5	110.4513	18
110.4375	13	110.4838	20
110.4725	14.5	110.5175	18.75
110.505	13.25	110.55	21.75
110.5363	13.75	110.5838	24
110.5713	13	110.6175	21
110.6038	17	110.65	18.25
110.64	16.5	110.6875	22.25
110.6738	16.75	110.72	15
110.7038	13.5	110.75	22.25
110.735	13.75	110.785	20.75
110.7688	15.75	110.8163	17.75
110.7988	15.25	110.8475	21.5
110.835	17	110.8813	21
110.8738	12.25	110.9163	19.75
110.9063	13.25	110.9513	20.5
110.9375	13	110.9838	21
110.9725	13.5	111.0175	21.25
111.005	14.5	111.05	17.5
111.0363	13.25	111.0838	18.5
111.0688	16.75	111.1175	17.5
111.1038	14.5	111.1525	18
111.14	16.25	111.1875	18.25
111.1738	11.25	111.22	14.75
111.2038	14.25	111.25	21
111.235	14	111.285	17.25
111.2688	12.25	111.3163	19
111.2988	14.5	111.35	26.75
111.335	15.75	111.3825	18.75
111.3738	12	111.42	17.5
111.4063	15	111.4563	18.5
111.4375	13.5	111.485	15.5
111.4725	16.75	111.5188	20.25
111.505	13.5	111.5513	16.5
111.5363	9.75	111.585	14.25
111.5688	16.25	111.62	17.25
111.6038	11.5	111.6563	19

111.64	14.5
111.6713	14.5
111.7038	14
111.735	15.5
111.7688	16.25
111.7988	14
111.835	16.5
111.8738	16
111.9063	14.5
111.9375	15.75
111.9725	16.25
112.005	13.25
112.0363	13.25
112.0688	15.75
112.1038	17
112.14	11.25
112.1713	12.25
112.2038	9.75
112.2363	14.25
112.2688	12
112.2988	14.25
112.335	13.25
112.375	12.5
112.4063	11.25
112.4375	12.25
112.4725	12.25
112.505	9.5
112.5363	13.25
112.5663	11.5
112.605	15.5
112.64	11.5
112.6713	11.25
112.7038	11.75
112.7363	9.5
112.7688	10.25
112.7988	12.75
112.835	8.5
112.875	12.5
112.9063	12.5
112.9375	9.25
112.9725	11.25
113.005	10
113.0363	11.25
113.0663	10.75
113.105	9.75
113.14	9
113.1713	13
113.2038	9
113.2363	9.25
113.2688	13

111.6888	18
111.7225	13.75
111.7538	17.25
111.7888	19
111.8213	20
111.8538	18.5
111.885	14
111.9213	21
111.9563	20
111.9875	18.5
112.0213	22.75
112.0538	19.25
112.0838	19.25
112.1213	21
112.16	16
112.1913	18.5
112.2238	20
112.2538	19
112.2863	15.75
112.3213	20.5
112.3525	21
112.385	20.75
112.4213	19.75
112.4563	16.25
112.4875	23
112.5213	22
112.5538	20.5
112.5838	21.25
112.6213	19.75
112.66	20.25
112.6913	20.5
112.7238	21.5
112.7538	18.5
112.7863	20.5
112.8213	21
112.8525	23.5
112.885	18.75
112.9213	19
112.9563	21.25
112.9875	17.5
113.0213	21.5
113.0538	22.5
113.0838	20.75
113.1213	20.5
113.1575	20.5
113.1925	21.5
113.2238	20.75
113.2538	19.25
113.2863	21
113.3213	22.5

113.2988	9	113.3525	18.25
113.3338	8.75	113.3875	17.75
113.375	9.5	113.4213	14.5
113.4063	12.75	113.4563	18.5
113.4363	14	113.4875	18.25
113.4725	9	113.5213	14
113.505	16.25	113.5538	20.5
113.5363	16.5	113.5838	23.25
113.5663	14.5	113.6225	18.25
113.605	19	113.6575	22
113.64	20.5	113.69	20.5
113.6713	19.75	113.7238	21
113.7038	12.25	113.7538	21
113.7363	17.25	113.7863	22.25
113.7688	16	113.8213	20
113.7988	13	113.8525	17.75
113.8338	13.25	113.8875	19.75
113.875	14.25	113.9213	18
113.9063	14	113.9563	22
113.9363	12.25	113.9875	19.5
113.9725	11.5	114.0213	20.5
114.005	14.75	114.0525	25.5
114.0363	13.25	114.0838	21
114.0663	16.75	114.1213	22.75
114.105	12.25	114.1575	22.75
114.14	13.25	114.19	18
114.1713	14.75	114.2238	21.75
114.2038	11.5	114.2538	24.25
114.2363	14.75	114.2863	19.75
114.2688	15	114.3213	24.75
114.2988	12.5	114.3525	19.5
114.3338	15.75	114.39	22.5
114.375	12.75	114.4213	19
114.4063	14.25	114.4563	18.5
114.4363	13.25	114.4875	20.25
114.4725	14.25	114.5213	22
114.505	19.25	114.5525	21
114.5363	20	114.5838	19.25
114.5663	16.25	114.6213	28
114.6025	15	114.6575	26.5
114.6413	17.5	114.69	27.25
114.6713	17	114.7238	22.75
114.7038	12.75	114.7538	26.25
114.7363	15.5	114.7863	21
114.7688	19.5	114.8213	21
114.7988	16.75	114.8525	26.25
114.8338	15.5	114.89	21.5
114.875	15.5	114.9213	23.75
114.9063	15.5	114.9563	19.75
114.9363	11	114.9875	28

114.9725	16.75	115.0213	23.25
115.005	16.75	115.0525	24.25
115.0363	11.25	115.0838	28.75
115.0663	14.5	115.1225	30.75
115.1025	14	115.1575	23.5
115.14	11.25	115.19	24
115.1713	15.25	115.2238	23.25
115.2038	15.25	115.2538	25
115.2388	13.25	115.2863	25.25
115.2688	10.75	115.3213	25.25
115.2988	13	115.3525	24.75
115.3338	10	115.39	24.5
115.375	12.75	115.4213	23.75
115.4063	11.25	115.4563	28.75
115.4363	9.25	115.4875	24.5
115.4725	15.5	115.5213	24.25
115.505	15	115.5525	27
115.5363	13.25	115.5838	25.5
115.5663	14.75	115.6238	29.75
115.605	14.25	115.6575	27
115.64	12.25	115.69	29.25
115.6713	14	115.7238	22.5
115.7038	14.25	115.7538	24.75
115.7363	15.75	115.7863	27.5
115.7688	16.5	115.8188	25
115.7988	16	115.8525	25.5
115.8363	16.25	115.89	22.25
115.875	13.25	115.9213	27
115.9063	13	115.9563	23.75
115.9363	14.25	115.9875	25
115.9725	13	116.0213	23.25
116.005	12.5	116.0525	22.25
116.0363	10.75	116.0838	20.5
116.0663	9.75	116.1225	24.75
116.105	9.5	116.1575	20.25
116.14	9.75	116.19	23
116.1713	14	116.2238	22.25
116.2013	9	116.2538	23
116.2363	13.25	116.2863	24.75
116.2688	12	116.3188	21.5
116.2988	10.5	116.3525	23.25
116.3363	11	116.39	20.75
116.375	7.75	116.4213	25.75
116.4063	12	116.4538	26
116.4363	13	116.4875	23.25
116.4725	13	116.5225	25.75
116.505	15.75	116.5525	25.5
116.5363	15.75	116.5838	29
116.5663	13.75	116.6238	28.75
116.605	11.75	116.6575	27.25

116.6425	14.5	116.69	24.25
116.6713	11.5	116.7238	22.75
116.7013	10	116.7538	26.5
116.7363	12.75	116.7863	24.25
116.7688	15	116.8188	24.25
116.7988	13.5	116.855	25.75
116.8363	14.75	116.89	26
116.8738	11	116.9213	27.25
116.9063	11.5	116.9538	26.25
116.9363	10.25	116.9875	22.5
116.9725	16.75	117.0213	25.25
117.005	17	117.0525	26.5
117.0375	15	117.0863	25.75
117.0663	13	117.1238	25.5
117.105	14.75	117.1575	23.75
117.14	12.25	117.19	27.25
117.1725	15.5	117.2238	24.25
117.2013	14	117.2538	25.75
117.2363	12	117.2863	25.75
117.2688	16.5	117.3188	26.5
117.2988	17.75	117.355	21.5
117.3363	14	117.39	22.25
117.3738	16.25	117.4213	27
117.4063	13.75	117.4538	22.25
117.4363	14	117.4875	22.5
117.475	15.75	117.5213	22.5
117.505	15	117.5525	26
117.5363	14.25	117.5825	28
117.5663	14.25	117.6238	22.75
117.605	17.75	117.6575	24.25
117.64	19.75	117.69	25.25
117.6713	20.25	117.7238	25
117.7013	14	117.7538	25.75
117.7363	16.75	117.7863	21.5
117.7688	16	117.8188	21.5
117.7988	17.75	117.855	29
117.8363	16	117.89	23.75
117.8738	19.25	117.92	22
117.9063	19.5	117.9538	23.25
117.9363	17	117.9875	22.25
117.975	15.5	118.0213	21.75
118.005	14.5	118.0525	20
118.0363	15	118.0825	22.5
118.0688	14.5	118.1238	22
118.105	12.75	118.1575	22
118.14	12.25	118.19	24
118.1713	11.25	118.2238	23.75
118.2013	12.5	118.2538	27.5
118.2363	11.5	118.2863	21.75
118.2688	11.5	118.3188	22

118.2988	9.75	118.3575	26.75
118.3388	14	118.39	23.25
118.3738	12.25	118.42	23
118.4063	13.25	118.4538	20.25
118.4363	11	118.4875	23.75
118.475	13	118.5213	23.5
118.505	11	118.5525	23.25
118.5363	13.25	118.5838	28.5
118.57	17	118.6238	20
118.605	16	118.6575	24
118.64	13.5	118.69	20.75
118.6713	13.25	118.7238	20.75
118.7013	11.75	118.7538	21.25
118.7363	12.25	118.7863	20
118.7688	11.75	118.8188	20.5
118.7988	13.5	118.8588	18.5
118.8388	13.5	118.89	17.5
118.8738	17.25	118.92	19.5
118.9063	16	118.9538	21
118.9363	16	118.9875	19
118.9725	13.25	119.0213	19.25
119.005	16.5	119.0525	22
119.0375	15.5	119.0838	19.25
119.07	12.5	119.1238	18.75
119.105	17.25	119.1575	18.75
119.1375	12	119.19	24.5
119.1713	15.5	119.2213	22.75
119.2013	14.25	119.2538	21.75
119.2363	10.5	119.2863	23.25
119.2688	13	119.3188	20.75
119.2988	14.5	119.355	26.75
119.3388	10.25	119.39	25.25
119.3738	11.5	119.42	24.75
119.4063	13.75	119.4563	29.75
119.4363	14.5	119.4875	22
119.4725	16.75	119.5213	20
119.505	10.25	119.5525	20.75
119.5363	11	119.5838	20.25
119.57	11	119.6238	19.5
119.605	12.25	119.6575	19
119.6375	14.75	119.69	25.5
119.6713	13	119.7213	20.5
119.7013	14.5	119.7538	23.25
119.7363	12.5	119.7863	21
119.7688	10	119.8188	22.75
119.7988	12	119.855	17.5
119.8388	12.25	119.89	22
119.8738	8.75	119.92	26
119.9063	11	119.9538	19.75
119.9363	15.5	119.9875	15.5

119.9725	9.75	120.02	20.25
120.005	15.75	120.0525	17.25
120.0363	15	120.0838	17
120.07	14	120.1238	16.5
120.105	13.75	120.1575	19.25
120.1375	13.75	120.19	20.25
120.1713	12.75	120.2213	26.25
120.2013	16.5	120.2538	25.25
120.2363	11.75	120.2888	21.75
120.2688	16	120.3213	20.25
120.2988	13.5	120.355	20.75
120.3388	13.5	120.39	22.25
120.3738	13.25	120.42	18.25
120.4063	15	120.4538	24
120.4363	12	120.4875	19.75
120.4725	13.5	120.52	22.25
120.505	13.25	120.5525	20.25
120.5363	15.5	120.5838	20.5
120.57	13.75	120.6238	18
120.6075	12	120.6588	18.75
120.6375	12.5	120.69	23
120.6713	13	120.7213	21.25
120.7013	11.5	120.7538	22.25
120.7363	13	120.7863	17.25
120.7688	13.5	120.8213	16.5
120.7988	11	120.855	14.5
120.8388	15.25	120.89	20.25
120.8738	13.25	120.92	21.25
120.9063	10.5	120.9538	15.75
120.9363	12.75	120.9875	17.25
120.9725	14.75	121.02	21.25
121.005	9.75	121.0525	19
121.0363	15.5	121.0838	21.25
121.0713	12.25	121.1238	20.25
121.105	15	121.1575	20.25
121.1375	12.75	121.19	24
121.1713	11.25	121.2213	21.75
121.2013	13	121.2538	18
121.2363	9.25	121.2863	22.25
121.2688	12.25	121.3213	18.25
121.2988	10	121.355	21
121.3388	12.75	121.39	22
121.3738	12.25	121.42	18
121.405	13.25	121.4538	18.5
121.4363	10.25	121.4863	20
121.4725	12	121.52	19.25
121.505	13.5	121.5525	20.75
121.5363	11.75	121.5863	20.5
121.5713	15	121.6225	18
121.605	15.75	121.6575	18

121.6375	13.5	121.69	18.75
121.6713	12	121.7213	16.25
121.7038	12.5	121.7538	18.25
121.7363	15.25	121.7863	19.75
121.7688	13.25	121.8213	17.25
121.7988	15.25	121.855	18.5
121.8388	10.25	121.89	16.25
121.8738	12.5	121.92	20.25
121.905	16.5	121.9538	17.75
121.9363	15.75	121.9863	14.75
121.9725	15	122.02	14.25
122.005	14	122.0538	16.5
122.0363	14.5	122.0863	15.75
122.0713	11.5	122.1225	19.25
122.105	16.25	122.1575	18.75
122.1375	14.25	122.19	22
122.1713	13.5	122.2213	17.25
122.2038	12.25	122.2538	16
122.235	12	122.2863	19.25
122.2688	15	122.3213	20
122.3	17	122.355	19
122.3388	16	122.39	21.75
122.375	13.25	122.42	18.5
122.4063	11	122.4538	19.75
122.44	14.25	122.4863	20.75
122.4738	12.25	122.52	17
122.5063	15	122.5538	19
122.5375	13.75	122.5863	17.5
122.5725	10.75	122.6225	19.25
122.6075	12.5	122.6575	19.75
122.64	16	122.69	19.25
122.6738	12.5	122.7213	17.5
122.7063	13.75	122.7538	15
122.7375	14.5	122.7863	20.75
122.7713	10.5	122.8225	17.5
122.805	13.75	122.8575	20.5
122.8413	11	122.89	18.75
122.875	9.75	122.92	17
122.9063	14	122.9538	22.75
122.94	12.5	122.9863	17.25
122.9738	13.75	123.02	16.5
123.0063	11.5	123.055	19.25
123.0375	12.75	123.0888	17.75
123.0725	12.5	123.1225	18
123.1075	15.25	123.1575	18
123.14	14.25	123.19	16.75
123.1738	11.25	123.2213	18.75
123.2063	14.25	123.2538	16
123.2375	12.25	123.2838	19.25
123.2713	11.25	123.3225	20.5

123.3075	11	123.3538	23
123.3413	12.25	123.39	20.25
123.375	12	123.42	19.5
123.4063	11.25	123.4538	20.75
123.44	11.5	123.4863	14.5
123.4738	13.5	123.52	19
123.5063	13	123.5538	19.25
123.5375	13.5	123.5863	22.75
123.5725	14.25	123.62	20.75
123.6075	12	123.6575	23
123.64	13	123.69	24.5
123.6713	14.25	123.7213	21.75
123.7063	11.25	123.7538	25
123.7375	10.75	123.7838	21.5
123.7713	10.75	123.8225	21.25
123.8075	12.5	123.8538	21.75
123.8413	13	123.89	20.25
123.875	13.25	123.92	22.75
123.9063	16	123.9538	19.75
123.94	16.25	123.9863	24.75
123.975	16.5	124.02	18.75
124.0063	12.75	124.0538	25.5
124.0375	15.25	124.0863	18.75
124.0725	17.25	124.12	25
124.105	15	124.1588	22.75
124.14	15.75	124.19	22.5
124.1713	13.5	124.2213	21.5
124.2063	15.5	124.2513	28.25
124.2375	15.25	124.2838	24.75
124.2713	15.25	124.3225	24.75
124.3075	13.5	124.3538	27.25
124.3413	15	124.39	28.5
124.375	15	124.42	23.75
124.4063	15	124.4538	24.5
124.44	12.5	124.4863	26
124.4738	11.5	124.52	22.25
124.5063	14.5	124.5538	21.5
124.5375	17.75	124.5863	22
124.5725	13.5	124.62	24
124.605	15.5	124.6575	19.5
124.64	11	124.69	25
124.6713	15.5	124.7225	20
124.7063	15.75	124.7513	26.5
124.7375	13.5	124.7863	23.5
124.7713	14.25	124.8225	24.5
124.8075	13.75	124.8538	18.75
124.8413	18.75	124.8875	26.75
124.875	15.25	124.92	19.25
124.9063	12	124.9538	28.5
124.94	15.5	124.9863	20.25

124.9738	16.25	125.02	26.25
125.0038	19.5	125.0563	20
125.0375	19	125.0863	21.75
125.0725	15.5	125.12	21.75
125.105	16	125.1575	23.5
125.14	17.5	125.19	17
125.1713	15.25	125.2213	27
125.2063	15.75	125.2513	22
125.2375	16.75	125.2863	19.5
125.2713	14.25	125.3225	24.25
125.3075	15	125.3538	26.5
125.3413	15.5	125.3875	17.5
125.375	15	125.42	24.75
125.4063	12.5	125.4538	18.25
125.44	12.5	125.4863	23.75
125.4738	15.25	125.5188	19.75
125.5038	19.75	125.5563	24.5
125.5413	17.75	125.5863	23.5
125.5725	14.25	125.62	23.75
125.605	16.75	125.6575	25.25
125.6425	13.5	125.69	20.5
125.6713	19	125.7213	19.25
125.7063	17.25	125.7513	21.75
125.7375	18.25	125.7875	23.25
125.7713	16.75	125.8225	22
125.8075	11.75	125.8538	19.5
125.8413	16	125.8875	22.75
125.875	16	125.92	20
125.9063	15.25	125.9538	19
125.94	14.75	125.9863	18.75
125.9738	16	126.0188	19.75
126.0038	14.75	126.055	19
126.0413	16.5	126.0863	17
126.0725	16.5	126.1175	18
126.105	13.5	126.1575	17.75
126.14	17.25	126.19	20.25
126.1713	14.25	126.2213	17.75
126.2063	15.5	126.2513	16.25
126.2375	18	126.2863	17
126.2713	15.5	126.3225	19
126.3075	16	126.3538	16
126.3413	15.25	126.3875	18.5
126.375	10.75	126.42	17.5
126.4063	12	126.4538	23.75
126.44	20.25	126.4863	18.75
126.4738	16.5	126.5188	20
126.5038	14.25	126.555	20.25
126.5413	21.5	126.5863	19
126.5725	16	126.6175	19.25
126.605	16.25	126.6575	20.75

126.64	16.25	126.69	23.25
126.6713	21.25	126.7213	21.25
126.7063	15.5	126.7513	22
126.7375	15	126.7875	21.25
126.7713	14.25	126.8225	21.75
126.8075	14	126.8538	22.25
126.8413	16.25	126.8875	21.25
126.875	14	126.92	22.5
126.9063	15.75	126.9538	23.75
126.94	16.75	126.9863	22.75
126.9738	20.5	127.0188	20.25
127.0038	19	127.055	19.5
127.0438	22.25	127.0863	19.5
127.075	17.5	127.1175	23.25
127.1063	19.25	127.1575	21.5
127.1413	16.75	127.19	22.75
127.1725	15.5	127.2213	24.75
127.2088	16.5	127.2538	22.5
127.24	17.5	127.2875	17.75
127.275	19.25	127.3225	20.75
127.3088	19.75	127.355	25.75
127.3438	21	127.3888	20.25
127.3775	15.75	127.4238	19.5
127.4088	17.5	127.455	21.5
127.4413	17.75	127.4875	18.75
127.475	19.5	127.52	16.5
127.505	16	127.5575	16.25
127.5438	14.75	127.5888	21.25
127.575	19.25	127.62	18.25
127.6063	19.5	127.6575	21.75
127.6413	17.25	127.6925	20.75
127.6738	17.5	127.7238	22.75
127.7088	19.25	127.7538	21.75
127.74	16	127.79	22
127.7738	16.75	127.8238	21.5
127.8088	21.75	127.8588	22.75
127.8438	19.25	127.8888	22.5
127.8775	21.25	127.9238	23.75
127.9088	20.5	127.955	24
127.9413	19	127.9875	24
127.975	19	128.02	29.25
128.0075	19	128.0575	24.5
128.0438	17.25	128.0888	22.25
128.075	23.75	128.12	21.75
128.1063	20.25	128.1575	24.25
128.1413	23.5	128.1925	23.75
128.1738	21.25	128.2238	25.25
128.2088	20.5	128.2538	26
128.24	22.5	128.2875	29
128.275	23.75	128.3238	23

128.3088	18	128.355	21.25
128.3438	20.5	128.3875	23.5
128.3775	21.75	128.4238	24.5
128.4088	18	128.455	25
128.4413	15.5	128.4875	24.75
128.475	20.25	128.52	28
128.5075	20	128.5575	30
128.5438	18	128.5888	24.75
128.575	19.25	128.62	22.25
128.6063	19.75	128.6575	28.25
128.6425	18.5	128.6925	23.75
128.6738	16.25	128.7238	24.75
128.7088	22.5	128.7538	25.5
128.74	19.5	128.79	27.5
128.775	21.25	128.8213	27.5
128.8088	19.5	128.855	24.75
128.8438	18	128.8875	26.5
128.8775	18	128.9238	24.25
128.9088	17.25	128.955	22.25
128.9413	17	128.9875	22
128.975	19.75	129.0225	27
129.0075	14.5	129.0575	24.5
129.0438	20.25	129.0888	27.5
129.075	17.5	129.12	25.75
129.1063	17.5	129.1575	25.75
129.1438	18.75	129.1925	26.5
129.1738	15.5	129.2238	23.25
129.2088	21.25	129.2538	26.5
129.24	19.5	129.2875	25.5
129.275	20.25	129.3213	26.5
129.3088	20.75	129.355	19.25
129.3438	22.75	129.3863	20.75
129.3788	18.75	129.4238	20.75
129.4088	18.75	129.455	24
129.4413	21.5	129.4875	23
129.475	19.25	129.5225	20.5
129.5075	19.25	129.5575	23
129.5438	17.25	129.5888	26.5
129.575	16.25	129.62	26.25
129.6063	18.25	129.6575	21.25
129.6413	15.25	129.6925	22.75
129.6738	14.75	129.7238	21
129.7088	13.75	129.7538	20.75
129.7413	14	129.7875	17.75
129.775	16.75	129.8213	21.5
129.8088	15	129.855	22
129.8438	12.75	129.8863	19
129.8775	14.5	129.9238	17.25
129.9088	15.75	129.955	22.25
129.9413	13.25	129.9875	27.5

129.975	12.75	130.0238	20.75
130.0075	12.25	130.0575	24.75
130.0413	16.25	130.0888	24.75
130.075	9.75	130.12	25.75
130.1063	14	130.155	23.25
130.1413	18.25	130.1925	20.5
130.1725	11.25	130.2238	19
130.2088	17.5	130.2538	22
130.2413	18.25	130.2875	19.25
130.275	15.5	130.3213	22.75
130.3088	16.25	130.355	17.75
130.3438	15.5	130.3863	20.25
130.3775	17	130.4238	21.75
130.4088	16.5	130.455	24.25
130.4413	15	130.4875	25
130.475	13.75	130.525	26.75
130.51	13	130.5563	22
130.5413	15.5	130.5888	19.25
130.575	11.5	130.62	22
130.6063	16.75	130.655	20.75
130.6413	15.5	130.6925	25.75
130.6725	15.5	130.7238	20
130.7088	16.5	130.755	20.25
130.7413	17.25	130.7875	25.25
130.775	12.25	130.8213	21.75
130.8088	12.25	130.855	18.5
130.845	18	130.8863	20
130.8775	11.5	130.9225	21.75
130.9088	13.75	130.955	19
130.9413	12.5	130.9875	20
130.975	17	131.025	25.5
131.01	15	131.0563	22.5
131.0413	12	131.0913	23
131.075	13.25	131.12	21.5
131.1063	15.5	131.1563	17.25
131.1413	13	131.1925	18.25
131.1725	12.5	131.2238	17
131.2088	10.75	131.255	20.75
131.2413	12.25	131.2875	18.5
131.275	12.5	131.3213	20.75
131.3088	10	131.355	16.75
131.3438	14	131.3863	16.25
131.3775	12.5	131.4213	21
131.4088	13	131.455	12.25
131.4413	14	131.4875	20.75
131.4775	14	131.525	20.25
131.51	14.5	131.5563	21.25
131.5413	18.75	131.5875	18.75
131.575	17.25	131.62	22.5
131.6063	12.5	131.6563	19.75

131.6413	15.25
131.6725	13.75
131.7088	11.25
131.7413	18.5
131.775	13
131.8075	11.25
131.8438	13.5
131.8775	12.75
131.9088	14.5
131.9413	14
131.9775	15.5
132.01	12
132.0425	17
132.075	18
132.1063	18.5
132.1413	13
132.1725	14
132.2088	16.5
132.2413	14.75
132.275	15
132.3075	19
132.345	17.25
132.3788	15.5
132.4113	13.75
132.4438	14.75
132.4788	13.5
132.5113	16.5
132.5438	13
132.5775	16.25
132.61	14.5
132.6425	14
132.6738	11.75
132.7113	12
132.7463	14.75
132.7775	14
132.81	19
132.845	14.75
132.8788	13.75
132.9113	14.5
132.9438	17.25
132.9788	13.25
133.0113	17
133.0438	14.25
133.0775	14.25
133.11	11
133.1425	10.75
133.1763	16.5
133.2138	14.75
133.2475	11.75
133.2788	18.75

131.6975	22
131.7238	24.75
131.755	20.25
131.7875	19.25
131.8213	22
131.855	21.75
131.8863	25.25
131.9213	21.5
131.955	16.75
131.9875	20
132.025	20
132.0563	17.5
132.0875	20.25
132.12	23
132.1563	20
132.1925	21.5
132.2238	23.25
132.255	21.25
132.2875	21.5
132.3213	20.75
132.355	25.25
132.3863	22.75
132.4213	18.25
132.455	20.5
132.4875	20.25
132.525	22.25
132.5563	19.5
132.5875	19.75
132.62	23
132.6563	20.25
132.6925	20
132.7238	23.25
132.755	19.5
132.7875	19
132.8213	22.75
132.855	16.25
132.8863	21.25
132.9213	20
132.955	23
132.99	20.75
133.025	21
133.0563	22.25
133.0875	21
133.12	20.5
133.1538	20.75
133.19	22.25
133.2238	22.75
133.255	22
133.2875	24.25
133.3188	20.75

133.3138	9.5	133.3575	25
133.3463	12.25	133.3875	18
133.38	11.75	133.4238	20.5
133.4125	12	133.4575	24
133.445	14	133.4913	19
133.4813	12	133.5263	19.25
133.5138	13.75	133.5575	22.5
133.5463	10.25	133.5888	23.75
133.58	14.5	133.6225	22.5
133.6125	14.75	133.6575	25.5
133.645	15.75	133.6888	27
133.6763	11.25	133.7263	26.75
133.715	13.75	133.7575	26
133.7475	12	133.79	24.5
133.7788	11.5	133.8213	27.5
133.8138	11.5	133.8575	25
133.8463	12.5	133.8875	24.25
133.88	13.75	133.9238	27
133.9125	11.5	133.9575	23
133.945	15	133.9913	29.25
133.9813	13.5	134.0263	23.75
134.0138	10	134.0575	29.25
134.0463	16	134.0888	24.25
134.08	11.75	134.1225	21.75
134.1125	12	134.1575	24.75
134.145	15	134.1888	21.25
134.1763	13.5	134.2263	20.5
134.215	13.5	134.2575	27.75
134.2475	12.75	134.29	26.75
134.2788	11	134.3213	25.5
134.3113	13.75	134.3575	28.75
134.3463	13.5	134.3875	28.25
134.38	11	134.4238	30.25
134.4125	9	134.455	22.5
134.4463	12	134.4913	26
134.4813	11	134.5263	26
134.5138	9.75	134.5575	26.75
134.545	13.75	134.5888	26.75
134.58	11.5	134.6225	28.25
134.6113	9	134.6575	26.5
134.645	12.25	134.6888	26.75
134.6763	10	134.7263	23.5
134.715	12	134.7575	32.75
134.7475	9.75	134.79	27.25
134.7788	12	134.8213	27.5
134.8113	12.25	134.8575	31
134.8475	15.5	134.8875	32.25
134.8813	12.25	134.9238	23.75
134.9138	14	134.955	30.75
134.95	10	134.9913	28

134.9838	11.5	135.0263	31.25
135.015	9.5	135.0575	30
135.0463	11.25	135.0913	26.5
135.0825	9.25	135.1225	24
135.1138	10.25	135.1575	28.5
135.1475	12.5	135.1888	21.25
135.1788	10.25	135.2263	26.5
135.2175	8	135.2575	28.25
135.25	8.75	135.29	26.75
135.2813	9.75	135.3213	29.75
135.3138	9.5	135.3575	27
135.3475	9.75	135.3875	27
135.3813	9.25	135.4238	25.75
135.4138	13	135.4538	25
135.45	14.75	135.4913	23.25
135.4838	17.75	135.5263	26.75
135.515	12.75	135.5575	22.25
135.5463	13.75	135.5875	26.5
135.5825	10.25	135.6225	27
135.6138	17.5	135.6575	22.75
135.6475	12.75	135.6888	23.75
135.6813	14.75	135.725	19.5
135.7175	13.25	135.7575	22.25
135.75	13	135.79	15.75
135.7813	15	135.8213	24
135.8138	11.5	135.855	21.25
135.8475	12.25	135.89	20.75
135.8813	15.5	135.9238	17.5
135.9138	13	135.9538	19.75
135.9475	12	135.9913	22
135.9838	10.5	136.0263	17
136.015	12.25	136.06	18.5
136.0463	13.75	136.0875	23
136.0825	14.75	136.1225	16.5
136.115	8.5	136.1563	21.5
136.1475	13.25	136.1888	24.25
136.1813	14.25	136.2263	23.5
136.2175	16	136.2588	21
136.25	15.5	136.29	20.5
136.2813	11.5	136.3213	24.5
136.3138	12	136.355	20.75
136.3475	13.75	136.39	25.5
136.3813	10.25	136.4238	19.25
136.4138	15.25	136.4538	25
136.45	11.75	136.4913	20.75
136.4838	9	136.5263	26.25
136.515	12.25	136.5575	28
136.5463	13	136.5875	21.5
136.5825	14.75	136.6225	21.5
136.6138	14.5	136.6563	18.5

136.6475	10.25	136.6888	19.5
136.6813	13.25	136.725	17.25
136.7175	12.75	136.7588	16.25
136.75	15.5	136.79	20.25
136.7813	14.75	136.8213	21.75
136.8138	11	136.855	18.5
136.8475	12.5	136.89	20.75
136.8813	14	136.9238	21.75
136.9138	10.5	136.9538	18.25
136.9475	12.75	136.9888	21.75
136.9838	12	137.0263	18.5
137.015	14.5	137.0575	18.75
137.0463	12.75	137.0875	18
137.0825	9.5	137.1225	22.75
137.1138	12.75	137.1563	19.5
137.145	8.75	137.1888	20.25
137.1813	13	137.225	20.5
137.22	9	137.2588	21.75
137.25	14.75	137.29	20
137.2813	8.25	137.3213	25.25
137.3138	10.5	137.3525	22.75
137.3475	10	137.39	21.75
137.3813	13	137.4238	29
137.4163	12.75	137.4538	22.25
137.4475	12	137.4888	22.5
137.4838	14.75	137.5263	17.5
137.515	12.5	137.5575	24.75
137.5463	13.75	137.5888	19.25
137.58	15.75	137.6225	19.25
137.6138	17.25	137.6563	21.5
137.645	16	137.6888	22.25
137.6813	13.25	137.7238	21.25
137.7213	14	137.7588	21
137.75	14	137.79	18.25
137.7813	11.5	137.8238	20.75
137.8163	16.5	137.8525	24.75
137.8475	13.75	137.8913	18
137.8813	15.25	137.9238	23
137.9163	16.25	137.9538	19.25
137.9475	10.75	137.9888	24.75
137.9838	12	138.0263	21
138.015	14.5	138.0575	21.75
138.0463	12.5	138.0875	18.75
138.08	14	138.1225	22
138.1138	12.25	138.1563	20.75
138.145	13.75	138.1888	20
138.1813	13.75	138.2238	21.75
138.22	11.5	138.2575	19.5
138.25	14.75	138.29	19.75
138.2813	16.75	138.3213	17.5

138.3138	14	138.3525	19.25
138.3475	10.25	138.39	18.75
138.3813	11.75	138.4238	22.75
138.4163	11.25	138.4538	21.25
138.4475	14.5	138.4888	22
138.4838	11.25	138.5263	23.75
138.515	16.25	138.5575	23.75
138.5463	15.5	138.5875	24.25
138.58	14.25	138.6225	23.25
138.6138	12.25	138.6563	19
138.6463	15	138.6888	22.5
138.6813	14.5	138.7238	19.25
138.72	13.5	138.7575	17.75
138.75	16.5	138.79	18.25
138.7813	12.75	138.8213	18.75
138.8138	18.25	138.8525	19.25
138.8475	17	138.89	17
138.8813	12.5	138.9213	19
138.9163	13.25	138.9538	20
138.9488	14.25	138.9888	13.5
138.985	12	139.0263	20.25
139.015	15.25	139.0575	18.75
139.0463	19.5	139.0875	25.75
139.08	37.75	139.1225	15.75
139.1138	15	139.1563	16.75
139.1463	36.5	139.1888	22.25
139.1825	14.75	139.2238	18.75
139.2175	34.25	139.2575	19.75
139.25	14.75	139.29	19
139.2813	14.75	139.3213	19
139.3138	20.25	139.3525	20.25
139.3475	28.5	139.39	22.5
139.3813	20	139.4213	19.25
139.4163	19.5	139.4538	21.25
139.4488	13.25	139.4913	21.5
139.4838	13.75	139.5238	23
139.5175	15.75	139.5575	26
139.5463	23.25	139.5875	26.25
139.58	16.25	139.62	25.25
139.6138	17	139.6563	24.5
139.6463	16.5	139.6888	22
139.6838	12.25	139.7238	24.25
139.7175	11.75	139.7575	20.5
139.75	17.75	139.79	21.75
139.7813	18	139.8213	24
139.8138	21	139.8525	18.25
139.8475	18	139.89	19.5
139.8813	14	139.9213	19.75
139.9163	16	139.9538	18.75
139.9488	13.5	139.99	19

139.9838	14.5	140.0238	21
140.015	20	140.0575	22.5
140.0463	16.5	140.0875	23.75
140.08	10.5	140.12	19.75
140.1138	12.75	140.1563	19.75
140.1463	12.25	140.1888	19.25
140.1838	9.25	140.2238	17.25
140.2175	13	140.2575	17.25
140.25	12.25	140.29	19.5
140.2813	12.25	140.3213	21.5
140.3113	13.5	140.3525	19.5
140.3475	13.75	140.39	24.25
140.3838	12.5	140.4213	21.5
140.4163	11.5	140.4538	21
140.4488	13.75	140.49	18.5
140.4838	16.25	140.5238	21
140.515	15.75	140.5575	23.75
140.5463	14	140.5875	17.75
140.58	12.5	140.62	27.25
140.6138	14	140.6575	21.25
140.6463	13.75	140.6888	22
140.6863	11.25	140.7238	20
140.7175	12.75	140.7575	18.25
140.75	12	140.7888	18.75
140.7813	14.25	140.8213	20
140.8113	9.5	140.8525	18.75
140.8463	10	140.8913	18.25
140.8813	11.25	140.9213	20.25
140.9163	10	140.9538	16.75
140.9488	12.75	140.99	16.75
140.9863	12.25	141.0238	19.5
141.015	11	141.0575	19.25
141.0463	13.75	141.0875	17.5
141.08	11.75	141.12	24
141.1138	11.25	141.1563	21.25
141.1463	9.75	141.1875	21
141.1863	9.5	141.2238	19.75
141.2175	13.75	141.2575	21.5
141.25	12.25	141.2888	21
141.2813	11	141.3225	21.25
141.3113	9.25	141.3525	24.25
141.3463	11	141.3888	25
141.3813	12.25	141.4213	23.25
141.4138	15	141.4538	28.75
141.4488	17	141.49	24
141.4838	14.5	141.5238	25.25
141.515	12.5	141.555	22.25
141.5463	10.5	141.5875	21.25
141.58	15.25	141.6213	16.25
141.6138	11.5	141.6563	25.25

141.6463	10.5	141.6875	23.25
141.6863	10.25	141.7238	21.75
141.7175	12.25	141.7575	19.5
141.75	13.75	141.7888	23.5
141.7813	9.75	141.8213	23.5
141.8113	12	141.8525	20
141.8475	8.75	141.8888	21.25
141.8813	12.25	141.9213	22.5
141.9138	11.5	141.9538	22.5
141.9488	10	141.99	25.75
141.9825	10.75	142.0238	21.25
142.015	12.75	142.055	27.25
142.0463	10.75	142.0875	27.5
142.08	10.5	142.1213	21.25
142.115	11.5	142.1563	22.75
142.1463	11.25	142.1875	22.25
142.1863	10	142.2238	24.25
142.2175	13	142.2575	18.25
142.25	10.25	142.2888	21.75
142.2813	8.75	142.3213	24.5
142.3113	11.75	142.3525	26
142.3463	12.5	142.3888	23.5
142.3825	15	142.4213	29
142.4175	9.75	142.4538	22
142.4513	12.25	142.49	25.25
142.485	8.75	142.5238	21.75
142.5175	9.75	142.555	25.5
142.5488	11.5	142.5875	24.25
142.5838	8	142.6238	23.75
142.62	10	142.6588	27.75
142.6525	10	142.6938	24.5
142.6888	10.25	142.7275	21.25
142.72	9.75	142.7575	21.25
142.7538	10	142.7913	23
142.7888	10.25	142.8238	24.5
142.8163	8.75	142.8538	25.75
142.85	9.25	142.89	19
142.8838	11.75	142.9225	19.75
142.92	12.75	142.955	17.25
142.9538	8.5	142.9913	18.5
142.9875	11.75	143.025	20.25
143.0188	15	143.0563	16.5
143.0488	10.5	143.09	20.75
143.0838	14.25	143.1238	20
143.1175	14	143.1563	23.25
143.1525	13.25	143.19	16.5
143.1888	11.75	143.2275	18.25
143.22	10.25	143.2575	23
143.2538	12.75	143.2913	22
143.2863	9	143.3213	22.5

143.3163	7.5	143.3538	24.25
143.35	12.5	143.39	23.25
143.3838	8.5	143.4225	22.75
143.42	7.25	143.455	19.75
143.4538	7.25	143.4913	22.25
143.4875	7.75	143.525	18.75
143.5188	8.5	143.5563	22
143.5488	9.25	143.59	20
143.5838	8	143.6238	25
143.6175	8.5	143.6563	19.75
143.655	9.75	143.69	23.75
143.6888	9.75	143.7275	20.25
143.7213	6.5	143.76	21
143.7538	11	143.7938	23
143.7863	13.5	143.8238	20
143.8163	10	143.8563	18.25
143.8525	15	143.8925	22
143.8888	10	143.9238	17.5
143.9213	12.5	143.9563	20.25
143.9538	11.75	143.9925	20.75
143.9875	8.75	144.0263	21.5
144.0188	7.5	144.0575	19.25
144.0488	9.75	144.09	18.75
144.0838	10.25	144.125	17.25
144.1175	11.25	144.1588	19.25
144.1525	11.25	144.1925	16.25
144.1888	11	144.23	17
144.22	11	144.2625	18
144.2513	8.75	144.2938	15
144.2863	13.75	144.3238	17.5
144.3163	11.25	144.3563	18.75
144.3525	9.75	144.3925	20
144.3888	10	144.4238	16.75
144.4213	7.75	144.4563	19.5
144.4538	7.25	144.4925	21.75
144.4875	10	144.5263	25.25
144.5188	11.25	144.5575	23.5
144.5488	8.75	144.59	20.75
144.5838	10.75	144.625	24.25
144.6175	10.5	144.6588	24.25
144.6525	13.5	144.6925	21.25
144.6888	12	144.73	24.25
144.72	12.5	144.7625	20.75
144.7513	13	144.7938	18
144.7863	9	144.8238	20
144.8163	11	144.8563	21.75
144.8525	11.5	144.8925	24
144.8888	14.25	144.9238	19.5
144.9213	11.5	144.9563	21.25
144.9538	11	144.9925	24.75

144.9875	12.75	145.0263	24
145.0188	9.75	145.0575	22.5
145.0488	13.5	145.09	22.75
145.0813	12	145.125	21
145.1175	8.25	145.1588	20.25
145.1525	10.5	145.1925	19
145.1888	12.75	145.2275	14.75
145.22	13.25	145.2625	23.25
145.2525	13	145.2938	16.5
145.2863	12.25	145.3238	16.5
145.3163	13.75	145.3563	21.25
145.3525	11	145.3925	19
145.3875	10.5	145.4225	18.25
145.4213	9.5	145.4588	19.5
145.455	11	145.4925	19.25
145.4875	9.25	145.5263	18.25
145.5188	11	145.5575	19.5
145.5488	9	145.59	25.25
145.5825	11.5	145.6275	23
145.6175	13.75	145.6613	32
145.6525	13	145.6938	15.75
145.6888	13.25	145.73	14.75
145.72	12.5	145.765	21
145.7513	15	145.7963	23.5
145.7888	14	145.8263	24.75
145.8163	15.25	145.8563	21.5
145.8525	13.25	145.8938	20.75
145.8875	13.75	145.9263	25.25
145.9213	13	145.9613	25.75
145.9538	15.25	145.9938	25.5
145.9875	12.5	146.0275	20.75
146.0188	10.5	146.0588	19.75
146.0488	11.5	146.0913	27
146.0825	13.5	146.1275	27
146.1188	16	146.1613	25.5
146.1525	12.75	146.1938	30.5
146.1888	17.25	146.23	25.75
146.22	15	146.2625	25.25
146.2513	14.75	146.2963	27.5
146.2863	13	146.3263	29.75
146.3163	12.75	146.3563	32.25
146.3525	10.5	146.3938	29
146.3875	10.25	146.4263	27
146.4213	11	146.4613	28.5
146.4538	14.25	146.4938	24.75
146.4875	11.75	146.5275	27.75
146.5188	10.25	146.5588	22.5
146.5488	12.75	146.5913	24.25
146.5825	11.5	146.6275	24
146.6188	13	146.6613	27

146.6525	13	146.6938	25.25
146.6888	12.75	146.73	25.75
146.72	12.25	146.7625	31
146.7513	13	146.7963	31.5
146.7863	11.25	146.8263	32
146.8163	13	146.8563	31.25
146.8525	10.25	146.8938	29
146.8875	10.5	146.9263	26
146.9213	10.25	146.9613	27
146.9538	12.5	146.9925	31.75
146.9875	10.5	147.0275	25.25
147.0175	14.5	147.0588	24.25
147.0488	12.25	147.0913	25
147.0825	12.25	147.125	22.25
147.1188	14.5	147.1613	26.25
147.1525	12.25	147.1938	27.5
147.1888	13	147.23	19.25
147.22	10.5	147.2625	26.5
147.2513	12.5	147.2963	21.75
147.2863	13	147.3263	22.5
147.3188	13	147.3563	19.25
147.3513	13.25	147.3938	20.75
147.3875	13.75	147.4263	19
147.4213	10	147.4613	25
147.4538	14.5	147.4925	19.25
147.4875	12.5	147.5275	22.25
147.5175	11.5	147.5588	18.25
147.5488	14.75	147.5913	19.75
147.5825	14.25	147.625	23.25
147.6188	16.75	147.6613	20.5
147.65	13.25	147.6938	19.5
147.6888	14	147.73	22
147.72	11.5	147.7625	15.25
147.7513	13.75	147.7963	19.25
147.7863	12.5	147.8263	22
147.8188	10	147.8563	21
147.8513	12.75	147.8938	15.75
147.8875	12.25	147.9263	16.25
147.9213	10.25	147.9613	23.5
147.9538	14.5	147.9925	25.75
147.9875	11.25	148.0275	18
148.0175	8.75	148.0588	19
148.0488	12.5	148.0913	20
148.0825	11	148.125	17.5
148.1188	11	148.1613	20.5
148.15	14.25	148.1938	18
148.1875	12.25	148.23	18.25
148.22	15	148.2625	19.75
148.2513	12.25	148.2963	19.75
148.2863	10	148.3263	18.5

148.3188	14
148.3513	12.5
148.3875	11.25
148.4213	11.5
148.4538	12.5
148.4875	13.5
148.5175	10.25
148.5488	12.5
148.5825	10.75
148.6188	13
148.65	9
148.6875	11.25
148.72	13.25
148.7513	12.5
148.7863	12.5
148.8213	17.5
148.8525	13.25
148.8888	13
148.9238	15.25
148.9563	9.5
148.9888	13.25
149.0188	13.25
149.0525	15.75
149.085	12.5
149.1213	14.25
149.1538	12.75
149.1888	11.75
149.2225	12.5
149.2538	12.5
149.2888	12.75
149.3213	10.5
149.355	13.75
149.3888	12.75
149.4238	11
149.4563	8.75
149.4888	11.75
149.5188	12.5
149.5525	11.5
149.585	13.5
149.6213	13
149.6538	9.5
149.6888	10.75
149.7225	14.5
149.7538	12.5
149.7863	10.5
149.8213	13.5
149.855	13.75
149.8888	11.75
149.9238	9.5
149.9563	12.25

148.3563	19.5
148.3938	19.75
148.4238	13.5
148.4613	15
148.4925	19
148.5275	21.25
148.5588	18.75
148.5913	17.75
148.625	17.75
148.6613	21.5
148.6988	19.5
148.73	16.75
148.76	18.25
148.7963	18
148.8263	18.75
148.8563	16
148.8913	21.75
148.9238	20
148.9613	20.25
148.9925	19.75
149.0275	22.5
149.0588	20.75
149.0913	20.5
149.125	19.25
149.1613	22.5
149.195	22.75
149.23	17
149.26	28.5
149.2963	23.5
149.3263	18.75
149.3563	21
149.3913	24.25
149.4238	20.25
149.4613	22
149.4925	20.5
149.5275	22
149.5588	21
149.5913	22.75
149.625	22
149.6613	21
149.695	17.25
149.73	18.75
149.76	18.5
149.7963	21.5
149.8263	19.75
149.8563	22
149.8913	19.25
149.9238	23
149.9613	20.25
149.9925	26.75

149.9888	12.75	150.0275	24.25
150.0188	11.75	150.0588	24.5
150.0525	15.25	150.0913	25
150.085	11.75	150.125	25.75
150.12	14.75	150.1613	25
150.1538	9.75	150.195	21
150.1888	12	150.2313	20
150.2225	13	150.26	23
150.2563	11.5	150.2963	24.25
150.2875	15.25	150.3263	24
150.3213	18	150.3563	19.25
150.355	12.25	150.3913	18.25
150.3888	10.75	150.4238	23.25
150.4238	14	150.4613	20.25
150.4563	11.75	150.4925	22
150.4888	10	150.5263	20.75
150.5188	11.5	150.5588	20.5
150.5525	13.25	150.5913	19
150.5863	10.5	150.625	24.75
150.62	10.5	150.66	19.25
150.6525	10.5	150.695	20
150.69	13.25	150.73	14.5
150.7225	11.5	150.76	19
150.7538	13.25	150.7963	20.75
150.7875	11.75	150.8263	18.25
150.8213	11.75	150.8563	17.75
150.855	11.25	150.8913	17
150.8888	14.75	150.9288	20.75
150.9238	15.25	150.9613	21.25
150.9563	13.75	150.9925	19.25
150.9888	11.25	151.0263	20.75
151.0188	16.25	151.0588	24.75
151.0525	16.5	151.0913	22.25
151.085	14.5	151.1238	20.25
151.12	16.75	151.16	20
151.1525	15.5	151.195	22.5
151.19	12.5	151.23	21.5
151.2225	14.25	151.26	22
151.2538	15.25	151.2963	19
151.2875	11.25	151.3263	22.25
151.3213	13.25	151.3563	24
151.355	15.25	151.3938	18.25
151.3888	14.75	151.4263	22.25
151.4238	12.5	151.4613	18.5
151.4563	13.75	151.4925	22.25
151.4888	19.5	151.525	23.25
151.5213	15.5	151.5588	19.75
151.5525	17	151.5913	20.5
151.585	12.75	151.6238	20.5
151.62	12	151.66	27

151.6525	13.5	151.695	21.75
151.69	15	151.73	20.25
151.7225	15	151.76	18.5
151.7538	10.25	151.7963	18.75
151.7875	15.25	151.8263	16.5
151.8213	14	151.8563	21.25
151.855	17.5	151.8913	22.5
151.8888	14.75	151.9263	21.25
151.9238	17.25	151.9613	19.75
151.9563	13.5	151.9925	24.75
151.9888	14.75	152.025	20.5
152.0213	15.25	152.0588	19.5
152.0525	13.75	152.0913	24
152.085	12	152.1238	22.5
152.1225	16.25	152.16	23.25
152.1525	12.5	152.1975	18.75
152.19	16.5	152.23	24.25
152.2225	12.5	152.26	21.25
152.2538	17.75	152.2963	19.5
152.2875	14.5	152.3263	21.75
152.3213	14.75	152.3563	18.75
152.355	19.5	152.3913	20.5
152.3875	15.5	152.4263	19.75
152.4238	14	152.4613	17.5
152.4575	12.75	152.4925	17
152.4888	14	152.525	20.25
152.5213	12.25	152.5588	22.75
152.5525	16	152.5913	17.75
152.585	17.25	152.625	21.5
152.62	17.25	152.66	19.75
152.6525	16	152.695	23
152.69	12.75	152.73	18.75
152.7238	11.75	152.76	18
152.7538	14.5	152.7938	18.5
152.7875	21	152.8263	19.75
152.8213	15.75	152.8563	18.25
152.855	13.5	152.8913	17.5
152.8875	14.5	152.9263	20.25
152.9238	16	152.9613	20
152.9563	15.5	152.9925	21
152.9888	14	153.0275	24.25
153.0213	14.5	153.0588	22
153.0525	14.75	153.0913	19.5
153.085	16.25	153.125	20.5
153.12	18.25	153.16	21.75
153.1525	16	153.195	23.5
153.19	16.5	153.23	20.25
153.2225	15.5	153.26	18.75
153.255	21.5	153.2938	18.75
153.2875	18.25	153.3263	19.25

153.3213	15.25	153.3563	24.5
153.355	18.5	153.3913	19.25
153.3875	17.25	153.4238	23.5
153.4238	15.75	153.4613	20.75
153.4563	16.25	153.4925	23
153.4888	13.75	153.525	24
153.5213	15	153.5588	19.75
153.5513	11.25	153.5913	19
153.585	14.5	153.625	22.5
153.62	10.75	153.6613	20.25
153.6525	11	153.695	19.75
153.69	11.75	153.73	24.75
153.7225	14.5	153.76	21.5
153.755	11	153.7938	22.75
153.7875	11.75	153.8263	20.25
153.8213	14.25	153.8563	19.25
153.855	18.75	153.8913	20.75
153.8875	14	153.9238	20.25
153.9213	12.75	153.9613	19.75
153.9563	17.5	153.9925	18.5
153.9888	17.75	154.025	19.5
154.0213	14.75	154.0588	23
154.0513	18.5	154.0913	18
154.085	14.75	154.125	23.25
154.12	20.75	154.1613	22
154.1525	16	154.195	20.5
154.19	18.25	154.23	20.75
154.2225	16.75	154.26	24.5
154.255	15.5	154.2938	23.5
154.2875	16.75	154.3263	21.25
154.3213	18.5	154.3563	19.5
154.355	15.5	154.3913	22.5
154.3875	15.5	154.4238	16.25
154.4213	14	154.4613	25
154.4563	15	154.4925	19.25
154.4913	13.5	154.525	20
154.5213	17.5	154.5588	26
154.5513	14	154.5913	21
154.585	14.25	154.625	22.75
154.62	11.5	154.6613	23.5
154.65	15.75	154.695	25
154.69	14	154.73	24.75
154.7225	12.75	154.76	20.5
154.755	12.5	154.7938	25.5
154.7875	17.5	154.8263	23
154.8213	13.5	154.8563	19.75
154.855	13.75	154.8913	19.75
154.8875	15.5	154.9238	22.75
154.9213	14.75	154.9613	21.25
154.9563	14.25	154.9925	18.75

154.9913	12.5	155.025	21.75
155.0213	14.25	155.0588	22
155.0513	13.5	155.0913	20.5
155.085	12	155.125	22
155.12	15.25	155.1613	25.25
155.15	16.25	155.195	21.25
155.19	14.5	155.23	21
155.2225	12.25	155.26	23.75
155.255	15.5	155.2938	28
155.2875	17.5	155.3263	27.5
155.3213	12.25	155.3563	19.25
155.355	15.5	155.3913	22.25
155.3875	13.75	155.4263	25.25
155.42	13.25	155.4613	23.5
155.4563	12.75	155.4925	19.5
155.4913	17.75	155.525	25.5
155.5213	13.5	155.5575	19.75
155.5513	14.5	155.5913	22.5
155.585	12	155.625	21.5
155.62	10.75	155.6625	17.75
155.65	17.5	155.695	20.25
155.69	12.25	155.73	22.25
155.7225	15	155.76	20.75
155.755	13.25	155.7938	22.25
155.7875	13	155.8263	19.75
155.8213	13.75	155.8563	19.5
155.855	14.75	155.8913	20.75
155.8875	14	155.9263	21
155.9213	13	155.9638	18.75
155.9563	15.75	155.9925	22.5
155.9913	15.5	156.025	19.75
156.0213	16.5	156.0575	20.25
156.0525	13.75	156.0913	18.5
156.085	17.25	156.125	22.75
156.1188	13.75	156.1613	17.5
156.15	11.5	156.195	18.25
156.19	13	156.23	20.5
156.2238	13.25	156.26	22.25
156.255	14	156.2938	17.75
156.2875	11.75	156.3263	16.25
156.3213	15.5	156.3563	17.25
156.355	13.75	156.3938	17
156.3875	11	156.4263	19.25
156.42	13.5	156.4613	20
156.4563	11.75	156.4925	21.25
156.4913	15.25	156.525	22.5
156.5213	14.25	156.5575	18
156.5513	11.5	156.5913	21.75
156.585	11.75	156.6263	20.25
156.6188	13.75	156.6613	22.75

156.65	14.75	156.695	26.25
156.6888	14.75	156.73	23.5
156.7238	16.5	156.76	16.25
156.755	17.5	156.7938	19.25
156.7875	15	156.8263	20
156.8213	14.5	156.8563	22
156.855	16.25	156.8938	22.25
156.8875	17.25	156.9288	21.75
156.92	15.25	156.9613	20.25
156.9563	13	156.9925	22.25
156.9913	13.5	157.025	24.75
157.0213	16	157.0575	22.75
157.0513	11.25	157.0913	27
157.085	14.75	157.1263	23
157.1188	13.5	157.1613	23.75
157.15	15.5	157.195	22.25
157.1888	15.75	157.23	17.5
157.2238	13	157.26	23
157.255	15.5	157.2938	21
157.2875	15	157.3263	23.75
157.3188	15.5	157.3563	21.5
157.355	12.25	157.3938	21.25
157.3875	12.75	157.4263	22
157.4175	15	157.4613	17.5
157.4588	11.25	157.4925	25
157.4913	14	157.525	19.5
157.5213	16	157.56	19.75
157.5513	15	157.5913	24.5
157.585	13.75	157.6263	23
157.6188	14.75	157.6613	19.25
157.65	14.75	157.695	21.75
157.6888	14.5	157.73	24.5
157.7238	15.75	157.76	19.5
157.755	12	157.7938	14.75
157.7875	16.5	157.8263	20
157.8188	19	157.8563	19.5
157.8525	16.25	157.8938	17.25
157.8875	14.5	157.9263	19.75
157.9175	18.5	157.9613	23
157.9588	13.25	157.9925	21.75
157.9913	13.25	158.025	17.75
158.0238	15	158.0575	24.5
158.0538	11	158.0913	24.5
158.0875	15	158.1263	19
158.1213	12.75	158.1625	22
158.1525	10.75	158.1975	24.75
158.1913	15.5	158.23	23.25
158.2263	12.5	158.26	19.75
158.2575	12.75	158.2938	22.5
158.2888	10.75	158.3263	20.5

158.32	11.5	158.3563	26
158.3538	13.25	158.3938	23
158.3888	15.5	158.4263	20.75
158.4213	11.5	158.4613	25.25
158.46	15	158.4925	21.75
158.4925	13	158.525	16.25
158.5238	14	158.5575	20
158.5538	12.25	158.5913	18.75
158.5875	13	158.6263	21.25
158.6213	16.25	158.6625	20.75
158.6525	13.75	158.6975	20.75
158.6925	13.75	158.73	17.5
158.7263	15.25	158.76	20
158.7575	13.25	158.7938	19.5
158.7888	13	158.8263	18.75
158.82	12.5	158.8563	17.75
158.8538	14.75	158.8938	19.25
158.8888	12.25	158.9288	19.5
158.9225	12	158.9613	19.5
158.96	14.25	158.9925	21.5
158.9925	14.5	159.025	18
159.0238	12.5	159.0575	20.25
159.0538	11.75	159.0913	16.25
159.0875	13.5	159.1288	21.25
159.1213	12.25	159.1625	21.75
159.1525	10	159.1975	17
159.1925	11.5	159.23	18.5
159.2263	11.5	159.26	22.25
159.2575	10	159.2938	20
159.2888	9	159.3263	22.5
159.32	12	159.3563	19.75
159.3538	9.5	159.3938	22
159.3888	11.25	159.4288	25
159.4213	11.25	159.4638	24.5
159.4575	12.25	159.495	19.75
159.4925	12	159.5288	18.75
159.5238	11.75	159.56	21.5
159.5538	12	159.5938	21
159.5888	14.75	159.6288	20.25
159.6213	9.25	159.6638	19.25
159.6525	16.5	159.6988	20.75
159.6925	14.5	159.7313	16.75
159.7263	12.5	159.7625	22.5
159.7575	15.25	159.795	18.5
159.7888	12.75	159.8313	18
159.82	17	159.8575	17
159.8538	13.75	159.8963	16.25
159.8888	15	159.9288	16
159.9213	16.5	159.9638	18.25
159.9575	15	159.995	14.25

159.9925	16.25	160.0288	14.25
160.0238	16	160.06	19.75
160.0538	16	160.0938	16
160.0875	19.25	160.1313	18
160.1213	16.25	160.1638	17
160.1525	17.5	160.1988	16.25
160.1925	16.75	160.2313	17
160.2263	14.25	160.2625	16.5
160.2575	17.5	160.295	16
160.2888	16	160.3275	13.25
160.32	16	160.3575	16.25
160.3538	18.5	160.3988	14.5
160.3863	18.75	160.4288	13
160.4238	20.25	160.4638	14.5
160.4575	18.75	160.4975	11
160.4925	15	160.5288	15.5
160.5238	19.75	160.56	13.75
160.5538	17.25	160.5938	13.25
160.5875	21.25	160.6313	18.75
160.6213	19.75	160.6638	16.25
160.6525	19	160.6988	15.25
160.6925	20.25	160.7313	21.5
160.7263	19.75	160.7625	14.5
160.7575	15.75	160.795	20.25
160.7888	15.75	160.825	17.5
160.82	18.75	160.8575	19
160.8538	16.5	160.8988	20
160.8863	15.75	160.9288	16.25
160.9238	19	160.9638	17
160.9575	14	160.995	18.5
160.9925	17.25	161.0288	19.5
161.0238	15.5	161.06	15.25
161.0538	14.75	161.0938	15
161.0875	15.5	161.1313	19
161.12	15.25	161.1638	15.75
161.1525	12.75	161.1988	17.75
161.1925	13.75	161.2313	16.75
161.2263	15	161.2625	13.25
161.2575	15	161.295	16
161.2888	16	161.325	12.75
161.3213	16	161.3575	17.5
161.355	15.25	161.3988	17.25
161.39	14.5	161.4288	14.25
161.4275	14	161.4638	20.25
161.4588	14.5	161.495	11.25
161.4938	20.25	161.5288	15
161.5263	28.25	161.56	16
161.5563	19.5	161.5938	15.5
161.5925	15.75	161.6313	19.5
161.6225	16	161.6638	14.75

161.655	19.75	161.6988	16.75
161.695	19.25	161.7313	18
161.7288	16.5	161.7625	16.25
161.76	17.5	161.795	18
161.79	17.25	161.825	20.25
161.8213	16.25	161.8575	14.25
161.855	17.5	161.8988	16.75
161.8875	15	161.9288	15.75
161.9275	14.25	161.9638	16.75
161.9588	21	161.995	14
161.9938	18.75	162.0288	18
162.0263	17.5	162.0625	21.75
162.0563	18.25	162.0938	18.25
162.09	18.5	162.1325	11.75
162.1225	15.75	162.1638	17.75
162.1575	15.75	162.1988	20
162.195	15.25	162.2313	22.75
162.2263	15	162.2625	17
162.26	15.75	162.295	21
162.29	14.5	162.325	21
162.3213	18.5	162.36	18.25
162.355	13	162.3988	21.25
162.3875	11	162.4288	20.5
162.4275	13.5	162.4638	20.5
162.4588	17.25	162.495	21
162.4938	12.75	162.5263	19.75
162.5263	15	162.56	14
162.5563	15.75	162.5938	15.5
162.59	14.5	162.6313	22.5
162.6225	17.25	162.6638	20.5
162.6575	17.5	162.6988	19.5
162.695	12	162.7313	18.75
162.7263	18.5	162.7625	22.75
162.76	12.75	162.795	28.75
162.79	14	162.825	25.5
162.8213	16	162.86	19
162.855	16	162.8988	17.25
162.8875	17.5	162.9288	23.25
162.9275	17	162.9638	23.25
162.9588	15.75	162.995	18.75
162.9963	17.5	163.0288	19.75
163.0263	17.25	163.06	19.5
163.0563	15.5	163.0938	14.75
163.09	18	163.1313	15.5
163.1225	16.5	163.1638	17
163.16	19.25	163.1988	19.25
163.195	14	163.2313	15.5
163.2263	14.25	163.2625	19.75
163.26	16.75	163.295	19.5
163.29	13	163.325	18.5

163.3213	13.25	163.3613	21.5
163.355	18.5	163.3988	16.75
163.3888	15.25	163.4288	17.5
163.4275	14.75	163.4638	15.25
163.4588	12.25	163.495	16.25
163.4938	14	163.5263	16.5
163.5263	16.5	163.56	15.75
163.5563	15.5	163.5938	17
163.59	15.25	163.6313	15.5
163.6225	14.5	163.6663	13
163.6588	14.5	163.6988	13.5
163.6925	14.75	163.7313	15.5
163.7263	11.5	163.7625	14.25
163.76	16.5	163.795	14.75
163.79	16.25	163.8275	14.75
163.8213	12.75	163.8638	15
163.855	13.75	163.8988	13.75
163.8863	17.25	163.9288	12.75
163.9275	16.5	163.9638	13.5
163.9588	14	163.995	22
163.9938	18.25	164.0263	14.75
164.0263	15	164.06	17
164.0563	18	164.0938	16.75
164.09	14.75	164.1313	20
164.1225	14	164.1638	20
164.1588	15.25	164.1988	20.75
164.1925	14	164.2313	20.75
164.2263	14.75	164.2625	16
164.26	15.25	164.295	19
164.29	9.75	164.3275	17.5
164.3213	16.5	164.3638	14.75
164.355	11.25	164.3988	15.75
164.3863	14.5	164.4288	20.25
164.4275	13	164.4638	16.75
164.4613	11	164.495	14
164.4963	12	164.5288	19
164.5288	11.75	164.56	19.5
164.5588	12.75	164.5938	15.5
164.5913	14	164.6313	21.25
164.6238	12.75	164.6638	19.75
164.66	16	164.6988	20.25
164.6938	14	164.7313	18.25
164.7288	14.75	164.7625	19.75
164.7613	12.75	164.795	19.25
164.7913	12.5	164.8275	18.5
164.8238	14.25	164.8638	17.75
164.8575	17	164.8988	16.5
164.8888	13.75	164.9288	17
164.93	14.5	164.9638	20
164.9613	14	164.995	19.5

164.9963	19	165.0263	17.25
165.0288	15.5	165.06	19.5
165.0588	16	165.0963	17.5
165.0913	16.25	165.1313	15.25
165.1238	19.5	165.1638	18.5
165.16	12.5	165.1988	18.25
165.1938	15.5	165.2313	17.5
165.2288	14	165.2625	19.25
165.2613	16.5	165.2963	19
165.2913	16	165.3275	18.5
165.3238	13.75	165.3638	17.75
165.3575	12.75	165.3988	19
165.3888	14	165.4288	17.25
165.43	14.5	165.4638	19.75
165.4613	9.5	165.495	20.75
165.4963	14.75	165.5263	25
165.5288	9.5	165.56	17
165.5588	10.75	165.5963	18.25
165.5913	14	165.6313	17.25
165.6263	12	165.6638	26.5
165.66	10.75	165.6988	24
165.6938	16.75	165.7313	19.25
165.7288	13.75	165.7625	20.75
165.7613	16.5	165.795	22.75
165.7913	19.75	165.8275	21.25
165.8238	16	165.8638	19.75
165.8575	17.25	165.8963	17.75
165.8888	18	165.9288	20.75
165.93	18.5	165.9638	18.25
165.9613	19.25	165.995	19.25
165.9963	14.75	166.0263	20.75
166.0288	17.25	166.06	19
166.0588	22.75	166.0963	20.5
166.0913	14.25	166.1313	20.5
166.1263	15.5	166.1638	17.25
166.16	18	166.1988	20
166.1938	13.5	166.2313	21.5
166.2288	17.5	166.2625	18.75
166.2613	17.75	166.295	20.5
166.2913	12.5	166.3275	19.5
166.3238	18.25	166.3638	21.5
166.3575	19.5	166.3963	24.5
166.3888	12.75	166.4288	21
166.4275	25.5	166.4638	17.5
166.4613	18.25	166.4975	22.25
166.4963	21.75	166.5288	17.5
166.5288	23.5	166.5613	15.25
166.5588	12.5	166.5975	20.25
166.5913	28.25	166.6325	20
166.6263	20.75	166.6663	19

166.6588	12.5	166.7	16.75
166.6938	24.75	166.7325	20
166.7288	20.5	166.7638	18.5
166.7613	15	166.7975	22
166.7913	20.75	166.8313	13.5
166.8238	16.25	166.8663	17.75
166.8575	16.5	166.9	17.5
166.8888	19.5	166.9313	21.5
166.9275	24	166.9663	18.75
166.9613	23.25	166.9975	18
166.9963	23.5	167.0288	19.5
167.0288	20.75	167.0613	21.5
167.0588	21.75	167.0975	20.25
167.0913	22.75	167.1325	14.25
167.1263	20	167.1663	20.5
167.1588	19.25	167.2	17.5
167.1938	20.5	167.2325	19.25
167.2288	18.5	167.2638	18.5
167.2613	19	167.2975	13.5
167.2913	21	167.3313	14.5
167.3238	19.5	167.3663	21.5
167.3575	16.25	167.4	20.75
167.3888	16.5	167.4338	18
167.4275	15	167.4663	17
167.4613	18	167.4975	17.25
167.4938	16.75	167.5288	16
167.5288	16.5	167.5613	16.75
167.5588	17	167.5975	21.25
167.5913	17	167.6325	18.75
167.6263	16.25	167.6663	19.5
167.6588	16	167.7025	22
167.6938	17.5	167.7325	21.75
167.7288	20.75	167.7638	21
167.7613	17.25	167.7975	22.25
167.7925	18	167.8338	21.5
167.8238	16.25	167.8675	23.5
167.8575	18	167.9013	23.75
167.8888	21	167.9363	22
167.9275	20.5	167.9688	24.75
167.9613	19.25	168	24
167.9938	20.5	168.03	22.25
168.0288	16.5	168.0625	24
168.0588	16.5	168.1038	22.75
168.0913	20	168.135	26.25
168.1263	19	168.1675	27.25
168.1588	19.25	168.1988	22.25
168.1938	18.75	168.2338	23
168.2288	19.75	168.2663	28
168.2613	19.75	168.3	33
168.2913	17.5	168.335	24

168.3238	14.25	168.3675	20
168.3588	16.5	168.4013	20.5
168.3888	17	168.4363	19.25
168.4275	16	168.4688	20
168.4613	14.5	168.5	22.25
168.4938	19.75	168.53	17.75
168.5288	16.5	168.5663	19.25
168.5588	16.25	168.6038	23
168.5913	17.25	168.635	23
168.6263	17.5	168.6675	17.5
168.6588	13.5	168.6988	22.25
168.6963	16.25	168.7338	25
168.7288	15	168.7663	23.75
168.7613	17.5	168.8	24.25
168.7913	16	168.835	22
168.8263	11.5	168.8675	20.75
168.8588	14.5	168.9013	18.75
168.8888	14	168.9363	28.25
168.925	16	168.9688	23.5
168.9613	16.5	169	21
168.9938	18	169.03	21
169.0288	19.75	169.0663	25.75
169.0588	16	169.1038	19.75
169.0913	19.25	169.135	28.5
169.1263	17.5	169.1675	36
169.1588	14.25	169.1988	21
169.1963	18.5	169.2338	23.5
169.2288	16.5	169.2663	23.25
169.2613	17.25	169.3	40.75
169.2913	18.75	169.335	47.5
169.3263	13	169.3675	18
169.3588	18		
169.3888	12	169.4363	45.5
169.425	12.25	169.4688	35.75
169.4613	13.5	169.5	31.75
169.4938	13.25	169.53	40.25
169.5288	15.25		
169.5588	18.5	169.6038	29
169.5913	15	169.635	35.75
169.6263	13	169.6675	32.25
169.6588	13	169.6988	25
169.695	14.5	169.7338	28.25
169.7288	13.75	169.7663	17.5
169.7613	15	169.8013	18.25
169.7913	15.75	169.835	25.5
169.8263	17.25	169.8675	24.25
169.8588	15.75	169.9013	17.5
169.8888	12.75	169.9363	25
169.925	14.25	169.9688	21.75
169.9613	14.75	170	22

169.9938	12.25	170.03	23.5
170.0288	13	170.0663	24.75
170.0588	14.25	170.1038	21
170.0913	13	170.135	27.5
170.1263	15	170.165	25.75
170.1588	15.5	170.1988	20
170.195	12.75	170.2338	31.75
170.2288	9.5	170.2663	38
170.2613	12.75	170.3013	32
170.2913	14.5	170.3363	24
170.3263	15.25	170.3675	31
170.3588	15.25	170.4013	31.25
170.3888	17.75	170.4363	23
170.425	15.75	170.4688	21.25
170.4613	15.25	170.5	21.5
170.4938	17	170.53	17.5
170.5288	17.25	170.5663	23.5
170.5588	12	170.6038	14.5
170.5913	11.75	170.635	17.5
170.6263	15	170.665	20
170.6588	14.75	170.6988	16.25
170.695	14.5	170.7338	16.75
170.7288	12.75	170.7663	17.5
170.76	10	170.8038	16.75
170.7913	10.25	170.8388	19.25
170.8263	11.75	170.8675	16.5
170.8588	10.25	170.9013	13
170.8888	13	170.9363	18.75
170.925	14	170.9688	15.75
170.9613	12	171	13.75
170.9938	12	171.03	15
171.0288	13.25	171.0663	16.75
171.0588	12.25	171.1038	17.75
171.0913	13	171.135	14
171.1263	13	171.165	12.25
171.1588	11.5	171.1988	17.25
171.1963	11.25	171.2338	15.5
171.2288	10	171.2663	16.5
171.26	10.75	171.3038	20
171.2913	12	171.3363	18.5
171.3263	9.5	171.3675	20.5
171.3588	6.25	171.4013	21.75
171.3888	14.25	171.4363	18.75
171.425	14	171.47	17.25
171.4613	13.5	171.5	19.25
171.4938	13.25	171.5325	16.25
171.5288	13.5	171.5663	17
171.56	17.25	171.6038	20
171.5913	12.5	171.635	18.5
171.6263	12.25	171.665	19

171.66	10.75	171.6988	20.75
171.695	13.5	171.7338	16
171.7288	12.5	171.7663	14.5
171.76	11.75	171.8038	17.5
171.7913	9	171.8363	24
171.8263	11	171.8675	17.5
171.8588	9.75	171.9013	16
171.8913	10.5	171.9363	15
171.9263	12.75	171.9688	17.75
171.9613	11.75	172	15.5
171.9938	7.75	172.0338	15.5
172.0288	13	172.0663	16.25
172.06	13.75	172.1038	16.75
172.0913	11	172.135	16.5
172.1263	13.75	172.165	16.75
172.1588	11.25	172.1988	20.25
172.1938	14.5	172.2338	19
172.2288	15	172.2663	17.5
172.26	16.75	172.3038	17.25
172.2913	9.5	172.3363	20.5
172.3263	9.5	172.3675	17.25
172.3588	10.25	172.4013	15.25
172.3913	14.75	172.4338	18.25
172.4263	13.75	172.4675	22.5
172.4613	13.5	172.5	20.5
172.4938	13.5	172.5325	23
172.5288	16.75	172.5688	22.5
172.56	15.25	172.6038	19.5
172.5913	13.75	172.635	26
172.6263	11.75	172.665	21.25
172.6588	12.75	172.6988	21.25
172.6938	14.5	172.7338	17.75
172.7288	12.5	172.7688	23.75
172.76	11.75	172.8038	19
172.7913	9.25	172.8363	21.75
172.8263	17	172.8675	15.25
172.8588	14	172.9013	20.5
172.8888	14.75	172.9338	18
172.9263	13.5	172.9675	27.75
172.9613	13	173	21.5
172.9938	11	173.0338	20.25
173.0288	12.5	173.0688	16.75
173.06	10.5	173.1038	19.25
173.0938	13.75	173.135	19.25
173.1263	11	173.165	20.75
173.1588	11.75	173.1988	19.75
173.1938	12.5	173.2338	20.25
173.2288	12.25	173.2675	21
173.26	9.25	173.3038	17.75
173.2913	10	173.3363	22

173.3263	14	173.3675	22.5
173.3588	12	173.4013	25.75
173.3913	15.25	173.4338	23.75
173.4263	11.25	173.4688	27
173.4613	10.75	173.5	22
173.4938	11.25	173.5338	31.75
173.5288	13.75	173.5688	21
173.56	13.25	173.6038	32.5
173.5938	12	173.635	20
173.6275	14	173.665	26.25
173.665	16.25	173.6988	21.5
173.695	12.25	173.7338	27
173.73	16.5	173.7675	23.25
173.7625	13.75	173.805	30.75
173.7963	10.25	173.8363	21
173.8288	12.75	173.8675	27
173.8613	10.75	173.9013	18.25
173.8963	14.5	173.9338	28.25
173.9288	12	173.9675	20
173.9638	11	174	25.75
173.9963	13.75	174.0338	24.75
174.03	10	174.0688	29
174.0613	10.5	174.1038	20.5
174.095	11	174.135	27.75
174.1275	11	174.1663	26.75
174.1625	11.75	174.1988	31.5
174.195	11.75	174.2338	23.75
174.23	11	174.2675	31.5
174.2625	8.75	174.305	26.5
174.2963	9.5	174.3363	24.5
174.3288	9.5	174.3675	26.25
174.3613	11.25	174.4013	28.75
174.3938	8.25	174.4338	27
174.4288	12.25	174.4675	25.25
174.4638	11.5	174.5025	24.5
174.4963	13	174.5338	26.75
174.53	14.25	174.5688	26.5
174.5613	16.25	174.6038	22
174.595	16.5	174.6375	27
174.6275	15	174.665	27
174.6625	11.5	174.6975	23.5
174.695	15	174.7338	22.5
174.73	14	174.7675	19.75
174.7625	13.25	174.805	22
174.7963	11	174.8363	18.75
174.8288	13	174.8675	21.5
174.8613	12.75	174.9013	21.5
174.8938	10	174.9338	18.25
174.9288	14.5	174.9675	25.25
174.9638	14	175.0025	21.5

174.9963	13.25	175.0388	19.25
175.0275	11.25	175.0725	16.25
175.0613	11.25	175.105	21.5
175.095	15.75	175.1375	18.75
175.13	12	175.1675	22
175.1625	14.5	175.2013	23
175.195	14.75	175.2338	21.5
175.23	11.75	175.2713	23.5
175.2625	15.5	175.3075	23
175.2963	14	175.3388	21.75
175.3288	12.5	175.37	23.75
175.3613	14.75	175.4025	22.25
175.3938	16.5	175.4325	23.5
175.4288	12.5	175.4688	25.25
175.4638	17	175.505	23.25
175.4963	17.25	175.54	20
175.5275	12.5	175.57	22
175.5613	14	175.605	21.75
175.595	15.5	175.6375	21.25
175.63	12.25	175.67	22
175.665	14.5	175.7	25.75
175.695	14.5	175.735	27
175.73	15	175.7713	20.75
175.7625	13	175.8075	22
175.7963	14	175.8388	22.5
175.8288	10.5	175.87	25.25
175.8613	16.75	175.9025	23.5
175.8938	15.5	175.9325	25.75
175.9288	12.25	175.9688	24.25
175.9638	11.75	176.005	22.75
175.9963	12.75	176.04	23.75
176.0275	12	176.07	19
176.0613	11.25	176.105	26.25
176.095	13	176.1375	25
176.13	11.75	176.17	27.75
176.165	12	176.2025	20.5
176.195	13.25	176.2375	24
176.23	11.5	176.28	25.25
176.2625	11.25	176.31	26.25
176.2963	10.5	176.3413	23
176.3288	7.75	176.3725	24
176.3625	12	176.4038	25.75
176.3938	12.25	176.4338	22.5
176.4288	13.25	176.47	27
176.4638	13.25	176.5088	26.25
176.4963	13.5	176.5413	26
176.5288	10.75	176.5713	21.5
176.5613	11	176.6063	21.5
176.595	11.5	176.64	22.25
176.63	12	176.67	20.5

176.665	11.75	176.7025	19.25
176.695	14.75	176.7375	18
176.73	12.25	176.7763	22.5
176.7625	11.25	176.81	24.25
176.7963	13	176.8413	25
176.8288	10.25	176.875	23.5
176.8625	16	176.9038	26.5
176.8938	13.75	176.9338	26.25
176.9288	12.25	176.9725	24.75
176.9613	14	177.01	24.25
176.9963	11	177.0413	28.5
177.0288	12.25	177.0713	18.75
177.0613	12.75	177.1063	24.5
177.095	13.75	177.14	23.25
177.13	13.75	177.17	21.75
177.165	9.25	177.2025	23.25
177.195	12.75	177.2388	18.5
177.23	8.5	177.2763	23.75
177.2625	15	177.31	21
177.2963	10.25	177.3413	20.25
177.33	11.5	177.3725	21.75
177.3625	11.25	177.4038	22
177.3963	11	177.4338	25.5
177.4288	11.5	177.47	21.75
177.4638	10	177.51	23
177.4963	12.5	177.5413	22.5
177.5288	12	177.5713	23
177.5613	9.75	177.6063	22
177.595	12.75	177.64	22.25
177.63	8	177.67	24.75
177.665	8	177.7025	19
177.695	9.25	177.7388	25
177.73	10.25	177.7763	26
177.7625	9.25	177.81	21
177.7963	8.5	177.8413	17.75
177.83	8.25	177.8725	21.25
177.8625	11.25	177.9038	21.75
177.8938	6.5	177.9338	21.5
177.9288	7.75	177.97	23.25
177.9613	9.25	178.01	21
177.9963	10	178.0413	24.25
178.0288	8.5	178.0713	18
178.0613	9	178.1063	22.5
178.0975	10.25	178.14	25
178.13	10.75	178.17	23.25
178.165	10	178.2025	22.25
178.195	9.5	178.2388	22.5
178.23	10.25	178.2763	22
178.2625	11	178.31	24.5
178.2963	10.75	178.3413	22.5

178.33	13.5	178.3725	20.75
178.3625	12	178.4038	28.75
178.3938	10.5	178.4338	25.5
178.4288	10.25	178.4725	24.75
178.4613	12.25	178.5088	22.5
178.4963	8.5	178.5413	20
178.5288	11.25	178.5713	22.25
178.5613	12.25	178.6063	20
178.5975	12.5	178.64	19.75
178.63	10.25	178.67	25
178.665	8.5	178.7075	17.75
178.695	9.75	178.7388	20.5
178.73	12.75	178.7763	20.5
178.7625	11.25	178.81	21.5
178.7963	8	178.8413	21.75
178.83	9	178.8725	25.25
178.8625	11.25	178.9038	19.75
178.8938	9	178.9338	22
178.9288	7.5	178.9725	20.75
178.9613	12.75	179.0113	24.75
178.9963	9.5	179.0438	23
179.0288	9	179.0738	22.25
179.0613	9.25	179.1075	19.75
179.0975	9	179.1413	20
179.13	8.5	179.1713	21.5
179.165	8.25	179.2075	24.5
179.1963	9	179.2425	20.25
179.23	9	179.2775	20.25
179.2625	10.75	179.3113	24.5
179.2963	8.25	179.3438	24
179.3313	9.5	179.375	25.5
179.3625	11.5	179.4063	21.75
179.395	10.75	179.4363	24
179.4288	10.5	179.4775	22.25
179.4613	11.75	179.5113	23
179.4963	9.25	179.5438	24
179.5288	11.5	179.575	22.25
179.5613	8.75	179.6075	22.25
179.5975	9	179.6413	27.25
179.63	8.25	179.6713	24
179.6625	9	179.7088	29.25
179.6963	10.25	179.745	22.25
179.73	7.5	179.7775	22.75
179.765	12.25	179.8113	24
179.7963	10	179.8438	24
179.8313	9.25	179.875	22
179.8625	13.5	179.9063	22.5
179.895	11.5	179.9363	24.5
179.9288	10.25	179.975	26.75
179.9613	7.25	180.0138	23.25

179.9963	12	180.0425	22.5
180.0288	12	180.0738	22.5
180.0613	11.25	180.1075	20.75
180.0975	10	180.1413	20.25
180.13	9.25	180.1713	20.25
180.1625	10.25	180.2088	22.75
180.1963	16	180.2425	23
180.23	12.5	180.2775	21
180.265	9.5	180.3113	21.5
180.2963	10.25	180.3438	22.5
180.3313	8	180.375	22.5
180.3625	11	180.4063	18.25
180.395	12.25	180.4388	22.5
180.4288	9.25	180.475	23.5
180.4613	9.5	180.5113	19.25
180.4963	11.25	180.5425	22.5
180.5288	12.25	180.5738	19.25
180.5663	14.75	180.6075	18.75
180.5975	13	180.6413	25.75
180.63	10.25	180.6713	23.75
180.6638	12.75	180.7088	20.75
180.6963	14.75	180.7425	24.5
180.73	15	180.7775	20.5
180.765	12.25	180.8113	21
180.7963	14.25	180.8438	23.25
180.8313	11.75	180.875	22.75
180.8625	11.75	180.9063	24.5
180.895	11.5	180.9425	25.75
180.9288	11.75	180.975	20.75
180.9613	15.5	181.0113	21.5
180.9963	11.25	181.0425	22.5
181.0288	10	181.0738	22
181.0663	14.75	181.1075	22.5
181.0975	12.25	181.1413	22
181.1313	11	181.1713	21
181.1625	11.25	181.2063	24
181.1963	10	181.2413	22.25
181.23	15.75	181.2775	23
181.265	10.75	181.3113	18.75
181.2963	12.75	181.3438	22.75
181.3313	12.25	181.375	20
181.3625	9.75	181.4063	24
181.395	10.5	181.44	20.5
181.4288	13	181.475	25.75
181.4625	12.25	181.5113	23.5
181.4988	13.5	181.5438	24
181.5313	14.25	181.5738	20.5
181.5675	8	181.6075	25.25
181.5988	15.75	181.6413	18.75
181.635	12.75	181.6738	20

181.6663	12.5	181.7063	19.5
181.7	22	181.7413	21.5
181.735	13	181.7775	20.75
181.7675	11.75	181.8113	23.75
181.8013	15.75	181.8438	22
181.835	15	181.875	19.25
181.8675	13.75	181.9063	19
181.8988	16.25	181.94	20.5
181.9325	12.75	181.975	23.5
181.9663	13	182.0113	18.75
182.0013	12.75	182.0425	21.5
182.035	13.5	182.0738	19.75
182.07	15	182.1075	16.75
182.1013	16.25	182.1425	24
182.1363	13.75	182.175	22.75
182.1663	13.25	182.2063	21.5
182.2025	12.5	182.2413	19.25
182.235	15.5	182.2775	23.5
182.2675	14.25	182.3113	20.75
182.3013	12.25	182.3438	21.25
182.3375	14	182.375	14.75
182.3675	11.5	182.4063	22.25
182.3988	15.5	182.44	21.25
182.4313	15.5	182.475	23.75
182.4663	10.5	182.5113	25.5
182.5013	13.75	182.5425	29.75
182.535	14.75	182.5738	23
182.57	11.75	182.6075	23
182.6013	13.75	182.6413	21.75
182.6363	10.25	182.675	21
182.6663	11.75	182.7063	17.75
182.7025	11.25	182.7413	20.25
182.735	9.75	182.7775	22.75
182.7675	10.25	182.8125	22.5
182.8038	11.5	182.8425	20.75
182.835	6.75	182.875	22.25
182.8675	11.25	182.9063	20
182.8988	14	182.94	21.25
182.9313	14.25	182.975	20.5
182.9663	14.5	183.0138	22
183.0013	12.5	183.0425	25
183.035	12	183.0738	28.5
183.07	12.25	183.1075	23
183.1013	13	183.1413	21.75
183.1363	12.5	183.175	21.5
183.1663	14.75	183.2063	23
183.2025	11.25	183.2413	20.25
183.235	13.75	183.2775	22
183.2675	14.25	183.3113	20.5
183.3038	10.5	183.3425	16

183.335	16.5	183.375	15.25
183.3688	14.75	183.41	18.25
183.3988	14.25	183.44	19.25
183.4313	13.5	183.475	20
183.4663	11	183.5113	20.25
183.5013	10	183.5425	16.25
183.535	14.5	183.5738	18
183.57	10.75	183.6075	15.75
183.6013	13	183.6413	15.5
183.6363	14.75	183.675	20.5
183.6663	15	183.7063	20.25
183.7025	13.25	183.7413	18.25
183.735	10.75	183.7775	25.5
183.77	13.25	183.8088	21.75
183.8038	12.75	183.8425	22.25
183.835	14.25	183.875	19.75
183.8688	14	183.91	21.75
183.8988	15	183.94	22.75
183.9313	14	183.975	22
183.9663	14.5	184.0125	23.25
184.0013	10.25	184.0425	25.25
184.0375	11.5	184.0738	24.75
184.07	11.5	184.1075	24.5
184.1013	12.75	184.1413	23.75
184.1363	10.25	184.175	21.25
184.1663	10.75	184.2063	23.5
184.2	12.25	184.24	20.5
184.235	13	184.2775	20.25
184.27	11.75	184.3088	18.75
184.3038	13.5	184.3425	16.5
184.335	9.5	184.375	19.25
184.3688	13.75	184.41	14
184.3988	13	184.4425	16.25
184.4338	12.5	184.475	18.25
184.4663	10.75	184.5125	14.75
184.5013	13	184.5425	17.25
184.5388	13.25	184.5738	22.5
184.57	14	184.6075	20
184.6013	12.75	184.6413	18.75
184.6363	13.5	184.675	22.75
184.6663	12	184.7063	23
184.7	13.5	184.74	18.25
184.735	14.25	184.7775	15.75
184.77	12	184.8088	21
184.8038	12.75	184.8425	18.25
184.835	14.25	184.875	19.25
184.8688	18	184.91	21.5
184.8988	14.25	184.94	23.75
184.9338	14.75	184.975	21.25
184.9688	14	185.0125	20.25

185.005	14.5	185.0425	16
185.0413	16.25	185.0738	19.25
185.0725	15.5	185.1075	21.75
185.1063	13.25	185.145	16
185.1375	14.25	185.175	22.25
185.1675	12.25	185.2063	18
185.2013	16.5	185.24	20.25
185.2375	15.5	185.2775	21.25
185.2713	11.75	185.3088	18.75
185.305	13.25	185.3425	23.75
185.3363	11.25	185.375	23.75
185.3713	13.75	185.41	21.5
185.4013	12.25	185.44	20.5
185.4375	16.75	185.475	16
185.4688	13.25	185.51	22
185.5063	14.25	185.5425	21.25
185.5413	15.25	185.5738	22.5
185.5725	15.5	185.6075	17.5
185.6063	16.75	185.645	16.75
185.6375	12.5	185.675	19
185.6675	11.5	185.7063	26.25
185.7013	10.25	185.74	13.5
185.7375	10	185.7775	19
185.7713	13.75	185.8088	19.75
185.805	10.25	185.8425	16.5
185.8363	10	185.875	19
185.8713	13.75	185.91	20.5
185.9013	11	185.94	20.5
185.9375	12	185.975	18.5
185.9688	11.5	186.01	18
186.0063	11.5	186.0425	19.25
186.0413	12.25	186.0738	19
186.0725	8.5	186.1075	18
186.1063	7.5	186.145	16.75
186.1375	9.5	186.175	22.5
186.1675	12	186.2063	18
186.2013	12.25	186.24	18
186.2375	15.5	186.2775	15.5
186.2713	15.5	186.3088	19.25
186.305	10.25	186.3425	18.25
186.3363	16.25	186.375	20.25
186.3713	13.25	186.4125	22
186.4013	13	186.44	23.25
186.4375	15	186.475	20.25
186.47	19.75	186.51	22.75
186.5063	12.5	186.5425	21.5
186.5413	12	186.5738	19
186.5725	11.5	186.6075	17.75
186.6063	12.75	186.645	20.5
186.6375	13.5	186.675	16.75

186.6675	11	186.7063	21.25
186.7013	11.5	186.74	17.25
186.7375	8	186.7763	15.5
186.7738	13.25	186.8088	16.75
186.805	8.75	186.8425	19
186.8363	8.5	186.875	16.75
186.8713	7.75	186.91	19.75
186.9013	11.5	186.94	19
186.9375	10	186.975	17
186.9675	9.5	187.01	17.5
187.0063	7.75	187.0425	16.5
187.0413	11.25	187.0738	17.5
187.0725	12	187.11	20.25
187.1063	10	187.145	21
187.1375	10.25	187.175	19
187.1675	12	187.2063	19.5
187.2013	12.25	187.24	18
187.2375	8.25	187.2763	19.5
187.2738	14.5	187.3088	19.5
187.305	10	187.3425	20.75
187.3388	12.25	187.3788	20
187.3713	6.5	187.41	23.25
187.4013	9.5	187.44	22.25
187.4375	11	187.475	20.5
187.4675	12.25	187.51	22.75
187.5063	14.5	187.5425	24
187.5413	8.75	187.5725	23.5
187.5725	10.75	187.6075	19
187.6063	9.5	187.645	25.5
187.64	8.75	187.675	20.5
187.6675	8	187.7063	23.25
187.7013	10.75	187.74	24.75
187.74	10.5	187.7763	25.5
187.7738	10.5	187.8088	21.5
187.805	7	187.8425	20.75
187.8388	10.25	187.8788	25
187.8713	8.5	187.91	24.25
187.9013	10.25	187.9425	26
187.9375	7.75	187.975	23.25
187.9675	8	188.01	20.5
188.0063	8	188.04	22.75
188.0413	10	188.0725	23.5
188.0725	11.75	188.1075	23.25
188.1063	9.75	188.145	24.75
188.1375	10.5	188.175	21.5
188.17	9	188.2063	22.25
188.2013	14.5	188.24	21.75
188.2375	12	188.2763	23.25
188.2738	9.25	188.3088	20
188.305	9.75	188.3425	23.75

188.3388	11	188.3788	23.25
188.3713	12.75	188.41	27
188.4013	15.25	188.44	31
188.4375	11.5	188.475	29
188.4675	10.75	188.51	31
188.5063	9.5	188.54	26.25
188.5413	10	188.5725	24.75
188.5725	10	188.6075	23.5
188.6063	9.5	188.645	18
188.6375	11.75	188.675	29
188.6725	10	188.7063	26
188.7013	9.5	188.7425	26.5
188.7375	10.25	188.7763	27.75
188.7738	11	188.8088	27
188.805	10	188.8425	27.5
188.8388	9.75	188.8788	20.5
188.8713	9.5	188.91	25.25
188.9013	8.75	188.94	23.25
188.9375	11	188.975	27.25
188.9675	11	189.01	23.5
189.0075	7.75	189.04	25
189.0413	12.25	189.0725	24
189.0725	11.25	189.1075	23.75
189.1063	9.75	189.145	26.75
189.1375	6	189.175	23.25
189.17	11.5	189.2063	23.5
189.2013	8.5	189.2425	24.75
189.2375	4	189.2763	19
189.2763	9.75	189.3075	18.75
189.3063	7.75	189.3425	22.75
189.34	7.75	189.3788	20
189.3738	8	189.41	19.25
189.4038	8.75	189.44	19.5
189.44	10.5	189.475	20.5
189.4725	8.5	189.51	15.75
189.51	7	189.54	23.75
189.5463	6.5	189.5725	18.25
189.575	7	189.61	18.25
189.6088	9.25	189.645	20.75
189.6388	8.75	189.675	17.75
189.6725	8.75	189.7063	22.5
189.705	10	189.7425	21.75
189.7388	10.5	189.7763	23.75
189.7763	9.25	189.8075	17
189.8063	8.5	189.8425	21
189.84	11	189.8788	25.75
189.8738	9.25	189.91	26.75
189.9038	9.5	189.94	21.25
189.94	8.75	189.975	23.5
189.9725	8.25	190.01	22.25

190.0088	8.5
190.0438	9.5
190.075	8.75
190.1088	12
190.1388	12.25
190.1725	10.5
190.205	7.5
190.2388	13.25
190.2763	12.5
190.3063	11.5
190.34	10.5
190.3738	10.75
190.4038	13.25
190.44	10.25
190.4725	10.75
190.5063	13.75
190.5438	13.25
190.575	10.5
190.6088	11.5
190.6388	9.5
190.6725	9.75
190.705	12.5
190.7388	12
190.7763	10.5
190.8063	13.25
190.84	12.75
190.875	12.5
190.905	12.25
190.94	12
190.9725	13.5
191.0063	13.25
191.0438	15.25
191.0763	13.25
191.1088	15.5
191.1388	14.25
191.1725	10.5
191.2075	13.5
191.2413	12.75
191.2763	12.75
191.3063	12.75
191.34	13.5
191.3738	12.75
191.405	9.5
191.44	12.25
191.4725	9.5
191.5063	11.25
191.5438	13.25
191.5763	13
191.6088	10.25
191.6388	10.25

190.04	24.25
190.0725	20
190.11	17.25
190.145	20.25
190.175	19.5
190.2063	21.5
190.2425	20.5
190.2763	17.5
190.3075	15
190.3425	18.75
190.3788	15.5
190.41	19.25
190.44	20
190.475	21.25
190.51	20.5
190.54	19.75
190.57	22.25
190.6075	20.5
190.645	20.75
190.675	20
190.7088	20
190.7425	20.25
190.7763	22
190.8075	21.5
190.8425	21
190.8788	20
190.91	19.5
190.94	21.75
190.9725	26.5
191.01	24
191.04	21
191.07	26.5
191.11	24.25
191.145	21.25
191.175	21
191.2063	23.25
191.2425	20.25
191.2763	18.25
191.3075	26.5
191.3425	23.75
191.3788	22
191.41	23.5
191.44	24.5
191.475	25.25
191.51	19.75
191.54	19
191.57	16.25
191.61	22.5
191.645	23.5
191.675	20

191.6725	18	191.7063	21
191.705	9.75	191.7425	19
191.7413	12	191.7763	23.25
191.7763	11.25	191.8075	20.5
191.8063	12.75	191.8438	15.5
191.84	12.25	191.8788	22.75
191.8738	17.5	191.91	19.75
191.905	13.25	191.94	22.25
191.9413	10	191.9738	19
191.9725	14.25	192.01	21.75
192.01	9.5	192.04	19.25
192.0438	11.25	192.07	17.5
192.0763	13.75	192.11	17.75
192.1088	14.25	192.145	16.75
192.1388	16.25	192.175	15.5
192.1725	13	192.2063	14.75
192.205	11	192.2425	23.75
192.2413	12.5	192.2763	20
192.2738	12.25	192.3075	15.5
192.3063	8.25	192.3413	21.5
192.34	10.25	192.3788	20.5
192.3738	13.5	192.41	22.25
192.405	10.25	192.44	20.75
192.4413	12.25	192.4738	18.25
192.4725	10.25	192.51	20
192.5063	11.25	192.54	24.5
192.5438	11.25	192.57	23.5
192.5763	12	192.61	20.75
192.6088	10.75	192.645	23.5
192.6388	11.25	192.675	23
192.6725	10.75	192.7063	19.5
192.705	11.25	192.7425	21
192.7413	8.5	192.7763	21.5
192.7738	10.75	192.8075	22
192.8063	9.5	192.8413	25.5
192.84	7.5	192.8788	22.25
192.8738	11.5	192.91	17.5
192.905	11.5	192.9425	24.75
192.9413	13.25	192.9738	24.25
192.9725	14.75	193.01	20.25
193.0075	10.5	193.04	22.75
193.0438	10	193.07	22.25
193.0763	11.25	193.11	26.75
193.1088	8	193.145	21.75
193.1388	12	193.175	21
193.1725	11	193.2063	22.75
193.205	8.5	193.2425	19.75
193.2413	8.5	193.2763	20.25
193.2738	11	193.3075	20.5
193.3063	9.75	193.3413	20.75

193.34	9.25
193.3738	10
193.405	11.5
193.4413	9.25
193.4725	9.25
193.5063	9.75
193.545	8
193.5763	12.25
193.6088	10.75
193.6413	10
193.6725	12.25
193.705	9.5
193.7413	8
193.7738	10
193.8063	10.5
193.84	7
193.8738	9.25
193.905	7.25
193.9413	9.25
193.9738	10.5
194.0063	9.25
194.0438	7.75
194.0763	10.25
194.1088	11.75
194.1413	8.5
194.1725	11
194.205	9.75
194.2413	12.5
194.2738	7.5
194.3063	11.25
194.34	8.25
194.3738	10.75
194.405	11.25
194.4413	8.5
194.4738	9.75
194.5063	10.25
194.5438	5.75
194.5763	6.25
194.6088	6
194.6413	8.5
194.6725	5.25
194.705	9.75
194.7413	11.5
194.7738	8
194.8063	9
194.84	12.75
194.8738	12.25
194.905	13.25
194.9413	8.25
194.9738	10

193.3788	20.5
193.41	22
193.44	21
193.4738	15.25
193.51	16.25
193.54	16.5
193.57	13.75
193.6088	17.5
193.645	18.25
193.675	19.5
193.7063	21
193.74	21.5
193.7763	22.5
193.8075	22.25
193.8388	23
193.8788	21.5
193.91	22
193.94	19.5
193.9738	21
194.01	23.75
194.04	21.75
194.0725	23.5
194.1088	18.25
194.145	21.75
194.175	17.5
194.2063	26
194.2425	18.75
194.2763	19.25
194.3075	23.5
194.3388	22
194.3788	22.5
194.41	24.25
194.44	23.75
194.4738	22.25
194.51	22.5
194.54	20.75
194.5725	22.25
194.6088	23.25
194.645	27.75
194.675	28
194.7088	29.25
194.74	25.25
194.7763	28.25
194.8075	23.5
194.8388	22.25
194.8788	21.75
194.91	18
194.94	23
194.9738	16.75
195.01	16.5

195.0088	13.5	195.04	18.25
195.0438	10.25	195.0725	21.75
195.0763	8.75	195.1088	18.75
195.1088	10.5	195.145	19
195.1413	11.25	195.175	24.75
195.175	11	195.2088	21.75
195.205	11	195.24	21.25
195.2413	12.75	195.2763	21.75
195.2738	10.75	195.3075	22.75
195.3063	12.5	195.3388	22.25
195.34	17.75	195.3763	20.75
195.3738	13	195.41	22.25
195.405	10.5	195.44	23.5
195.4413	12.5	195.4738	18
195.4738	13.5	195.51	22.5
195.5063	16	195.54	20.75
195.5413	9.75	195.5725	20.75
195.5763	13.75	195.6113	19.25
195.6088	13.75	195.645	18.75
195.6413	12	195.675	20.25
195.675	12.5	195.7088	18.5
195.705	13.25	195.74	21.25
195.7425	13	195.7763	22.25
195.7763	10.5	195.81	17.5
195.8088	10.75	195.8388	20
195.8413	15	195.8763	19.25
195.875	11.75	195.91	19.25
195.9063	13.25	195.9425	20.75
195.9425	13	195.9763	14.75
195.9763	14.5	196.0125	20.25
196.01	11.5	196.0425	16
196.0425	10.25	196.0775	17.25
196.0788	12.5	196.1088	18.5
196.1113	12	196.1463	19.25
196.1438	11	196.1763	17.25
196.1775	13.5	196.21	22.5
196.2075	14	196.2438	17
196.2425	10.25	196.2775	21
196.2763	10.25	196.31	18.75
196.3088	10.5	196.3425	23.25
196.3413	10	196.3788	18.75
196.3763	12.75	196.415	17.5
196.4063	10	196.445	21
196.4425	10.25	196.4763	19.75
196.4763	13.5	196.5125	21.75
196.5088	10.5	196.5425	22
196.5425	13.25	196.575	20.5
196.5788	10.5	196.6088	23
196.6113	12	196.6463	16.75
196.6438	13	196.6763	17.75

196.6775	11.75	196.71	17
196.71	11.75	196.7438	15.25
196.7425	11.75	196.7775	20.5
196.7763	12.5	196.81	18.25
196.8088	13.75	196.84	20.25
196.8413	12.25	196.8788	20.5
196.8763	11.5	196.9125	16.75
196.9063	12.75	196.9425	13.5
196.9425	11	196.9763	18.25
196.9763	10	197.0138	13
197.0088	7.25	197.0425	15.5
197.0425	12.25	197.075	16
197.0788	10.75	197.1088	19.25
197.1113	9.5	197.145	13.75
197.1438	12.5	197.1763	15.75
197.1775	15	197.21	19.25
197.21	15	197.2438	19
197.2425	12.75	197.2775	20.25
197.2763	12.25	197.3113	19
197.3088	13.75	197.34	18.5
197.3413	13.5	197.3788	19.25
197.3788	14	197.4125	15.75
197.4088	13.5	197.445	20
197.4463	15.75	197.4763	19.25
197.4788	11.5	197.5125	16
197.5113	13.75	197.5425	18.75
197.545	13.5	197.575	21.25
197.58	10.75	197.6088	17
197.6125	14	197.645	19.25
197.645	14.75	197.6763	19.25
197.68	12.75	197.71	20.25
197.7113	14.5	197.7438	25.75
197.7438	13.75	197.7775	16
197.7775	11.75	197.81	18.5
197.8113	11.25	197.84	19.5
197.8438	11.75	197.8788	19.5
197.8788	12	197.9125	20.5
197.9088	10.5	197.945	19.25
197.9463	9.25	197.9763	18.75
197.9788	16	198.0125	18
198.0113	7.5	198.0425	20
198.045	10.5	198.075	18.75
198.08	11	198.1075	14.75
198.1125	11.25	198.145	21.75
198.145	8.75	198.1763	21.75
198.18	9	198.21	24.25
198.2113	10.25	198.2438	21.25
198.2438	10.75	198.2775	23.5
198.2775	9.5	198.31	22.75
198.3113	10	198.34	17.75

198.3438	12	198.3788	21.75
198.3788	13.5	198.4125	17.75
198.4088	10.25	198.445	21.5
198.4463	11.5	198.4763	21.5
198.4788	12	198.5125	22.75
198.5113	7	198.545	19.5
198.5475	11	198.575	19.25
198.58	12.25	198.6075	20.75
198.6125	12.25	198.645	20.5
198.645	12	198.6763	18.5
198.6775	10.5	198.71	25
198.7113	8	198.7438	23
198.7438	10.25	198.7775	20
198.7775	7.75	198.81	20
198.8113	9.25	198.84	22.5
198.8438	10.25	198.8788	22
198.8788	10	198.91	22
198.9088	11.5	198.945	19.25
198.9463	9	198.9763	23.75
198.9788	9	199.01	20.25
199.0113	7	199.045	17.5
199.045	9.75	199.075	18.5
199.08	11	199.1075	20.5
199.115	9.75	199.145	16.75
199.145	9	199.1763	22
199.1788	13	199.21	20.25
199.2113	10.5	199.2438	19
199.2438	8.5	199.2775	23
199.2775	14	199.31	23
199.3113	8.75	199.34	23
199.3438	10	199.3788	22.25
199.3788	11.5	199.41	20.75
199.4088	11.5	199.445	24.5
199.4463	12	199.4763	29.5
199.4788	10.75	199.51	24
199.5113	8.25	199.545	28.75
199.545	11.5	199.575	23
199.58	11.75	199.61	23
199.615	10.5	199.6475	19.5
199.645	11.75	199.68	24.75
199.6788	9.75	199.7138	26.5
199.7113	13.25	199.7463	26.75
199.7438	11	199.7813	23.75
199.775	10.75	199.8138	23.5
199.81	11.75	199.8425	27
199.8438	10.25	199.8788	25.5
199.8788	11.25	199.9113	25
199.9088	13.5	199.9463	28.25
199.9463	10.5	199.9775	23.75
199.9788	11.5	200.0125	23

200.0113	13.5	200.0463	22.25
200.045	12.75	200.0763	24.75
200.08	12.25	200.11	20.5
200.115	11	200.1475	22.5
200.145	12.25	200.18	22
200.1788	9.75	200.2125	25.5
200.2113	12.25	200.2463	26
200.2438	9.25	200.2813	23.5
200.275	12.5	200.3125	23.5
200.31	13	200.3425	23.5
200.3438	9.5	200.3788	22
200.3788	11	200.4113	25.5
200.4088	12.5	200.4463	21.25
200.4463	10.5	200.4775	24.5
200.4788	10.5	200.5125	24.25
200.5113	10.5	200.5463	25.5
200.545	13.75	200.5763	28.75
200.58	13.75	200.61	21.5
200.615	10.75	200.6475	23.75
200.645	11.25	200.68	24.25
200.6788	10.5	200.7125	24.75
200.7113	12	200.7463	27.75
200.7438	11	200.7813	24.5
200.775	11.75	200.8138	22.5
200.81	14.25	200.8425	20.25
200.8438	12	200.8763	21.5
200.8788	14.75	200.9113	21.25
200.9088	12	200.9463	26
200.9463	13	200.9775	23.5
200.9788	12.5	201.0125	21.5
201.0113	14.75	201.0463	23
201.045	15.5	201.0763	26
201.08	16.25	201.11	22
201.115	18.5	201.1475	26.5
201.145	16.75	201.1788	22.75
201.1788	15	201.2125	19.5
201.2113	16.25	201.2463	21.75
201.2438	14.5	201.2813	24.75
201.275	14.25	201.3125	23.25
201.31	14.75	201.3425	24.25
201.3438	13	201.3763	23
201.3788	12.5	201.4113	21.75
201.4088	13.75	201.4463	20.25
201.4463	11.5	201.4775	21.75
201.4788	12.5	201.5125	21.5
201.5113	12.25	201.5463	24.75
201.545	12.75	201.5763	21.5
201.5788	15.75	201.61	15
201.615	16	201.6475	20.75
201.645	20.5	201.6788	20.5

201.6788	11.25	201.7125	23.25
201.7113	12.75	201.7463	18.5
201.7438	14.5	201.78	21.25
201.775	9.5	201.8125	18
201.81	9	201.8425	20.75
201.8438	11.75	201.8763	22.25
201.8788	14	201.9113	18
201.9088	13	201.9463	19.75
201.9463	12	201.9775	22.25
201.9788	9.5	202.0138	24.75
202.0113	11.5	202.0463	20.5
202.045	12.75	202.0763	20.75
202.08	13.25	202.11	22
202.115	11.25	202.1475	19
202.145	13.5	202.1788	22.75
202.1788	8.5	202.2125	23
202.2113	11.75	202.2463	24.5
202.2438	16.5	202.28	22
202.275	14	202.3125	22.5
202.31	10.75	202.3425	18.5
202.3463	14.25	202.3763	20.25
202.3788	14.5	202.4138	16.75
202.4088	12.5	202.4463	22.5
202.4463	11.75	202.48	15.25
202.4788	10.5	202.5163	20.5
202.5113	13	202.5488	19.25
202.5438	14.25	202.5788	20.75
202.5788	12.75	202.6113	19.25
202.615	12	202.6438	19.5
202.645	14	202.6825	16.5
202.6788	14.5	202.715	22.5
202.7113	10.75	202.7475	22
202.7438	14.25	202.7813	21.75
202.775	13.5	202.8138	20.5
202.8125	11.25	202.845	18.75
202.8463	13.25	202.8788	22
202.8788	12.75	202.9163	17.75
202.9113	10.5	202.9475	16.75
202.9463	12	202.98	15.5
202.9788	12	203.0163	21.75
203.0113	12.75	203.0488	21
203.0438	10.75	203.0788	16.5
203.0788	9.25	203.1113	21.75
203.115	10	203.1438	20
203.145	11	203.1825	21.25
203.1788	10.25	203.215	15.25
203.2113	10	203.2475	16
203.2438	12.75	203.2813	14.5
203.275	14	203.3138	17.25
203.31	14.75	203.345	19.25

203.3463	14	203.3788	21.5
203.3788	16.75	203.4163	19.25
203.4113	12	203.4475	20.25
203.4463	10.25	203.48	18
203.4788	12.25	203.5163	23.25
203.5113	12.25	203.5488	19.75
203.5463	14.25	203.5788	21.25
203.5788	11.75	203.6113	21.5
203.615	13.5	203.6438	21
203.645	11.25	203.6825	24.5
203.6788	11.25	203.715	20.25
203.7113	15.5	203.7475	22.75
203.7438	9.5	203.7813	21.75
203.775	12.75	203.8138	23.5
203.81	11.75	203.845	21.5
203.8463	15.25	203.8788	22.5
203.8788	10.75	203.915	25
203.9113	10.75	203.9475	18.75
203.9463	8.75	203.98	23.25
203.9788	11	204.0163	22.5
204.0113	12	204.0488	24
204.0438	10	204.0788	18.25
204.0788	13	204.1113	24.75
204.115	11.25	204.1438	22.5
204.145	10	204.1825	18.75
204.1788	12.75	204.215	18.5
204.2113	12.5	204.2475	17.5
204.2438	11.5	204.2813	21
204.275	10.5	204.3138	24
204.31	13.25	204.345	21.75
204.3463	10.75	204.3788	20.5
204.3788	13.5	204.4163	21
204.4113	9.5	204.4475	19.5
204.4463	12	204.48	26.75
204.4788	9.75	204.5163	21.5
204.5113	14.25	204.5488	19.75
204.5438	10	204.5788	22.5
204.5788	11.25	204.6113	24.25
204.615	11.5	204.6438	21
204.645	13.75	204.68	21.75
204.6788	9	204.715	21.75
204.7113	11.25	204.7475	19.25
204.7438	9.5	204.7813	14.75
204.775	13.75	204.8138	20.5
204.81	11.25	204.845	20.5
204.8463	11.5	204.8788	25.25
204.8788	12.5	204.915	19.5
204.9113	13	204.9475	18.5
204.9463	13.75	204.98	24
204.9788	12.25	205.0163	20.25

205.0113	13.5	205.0463	22.25
205.0438	11.25	205.0788	21.5
205.08	11.5	205.1113	21.25
205.115	13	205.1438	21.5
205.145	10	205.18	21.75
205.1788	12.75	205.215	23.75
205.2138	12.25	205.2475	18
205.2463	13.75	205.2813	20.75
205.2775	12.75	205.3138	23.5
205.3088	13.25	205.345	25
205.3475	15.25	205.3788	25.5
205.38	13	205.4138	19.75
205.4125	14.5	205.4475	22.75
205.4475	16.75	205.48	21.5
205.48	11.75	205.5163	22.25
205.5125	13.75	205.5463	20.25
205.5463	12	205.5813	18.25
205.5825	16	205.6113	20.5
205.6175	15	205.645	23.25
205.65	15	205.6775	24.5
205.6813	14.5	205.715	22.5
205.7138	15.75	205.7475	22.25
205.7463	10.5	205.7813	20.25
205.7775	14.25	205.8138	21.5
205.8088	12	205.8463	21
205.8475	12.5	205.8788	18.5
205.88	15.5	205.9138	15.25
205.9125	10.25	205.9475	19.25
205.9475	13	205.98	20.5
205.98	12	206.0163	21.75
206.0125	10.5	206.0463	22
206.0463	11	206.0788	22.25
206.0825	11.25	206.1113	20.5
206.1175	9.25	206.145	22
206.15	12.25	206.1775	20
206.1813	12	206.215	24.5
206.2138	17.25	206.25	22
206.2463	11.75	206.2813	21.75
206.2775	11.5	206.3163	18
206.3088	11.75	206.345	20.5
206.3475	10.75	206.3788	18.25
206.38	11	206.4138	21.75
206.4125	10.75	206.4475	19.25
206.4475	9	206.48	19
206.48	12.25	206.5163	22.25
206.5125	10.5	206.5463	23
206.5463	8.25	206.5788	21.75
206.5825	12	206.6125	24
206.6175	8	206.645	21.75
206.65	9.75	206.6775	21

206.6813	9.5	206.715	17
206.7138	12	206.75	21.75
206.7463	14.25	206.7813	17.5
206.7775	11.5	206.8138	16.5
206.8088	8.5	206.845	22
206.8475	14.5	206.8788	18
206.8838	15.5	206.9138	16.75
206.9125	12.25	206.9463	17.25
206.9475	15.75	206.98	17
206.98	15.5	207.0163	23.75
207.0125	13.5	207.0463	13.5
207.0463	10.25	207.0788	21.5
207.0825	12.5	207.1113	20.5
207.1175	11.5	207.145	18.5
207.15	9.75	207.1775	18.5
207.1813	11.25	207.215	20.25
207.2138	11.75	207.25	23
207.2463	14	207.2813	21.25
207.2775	10.5	207.3138	16.25
207.3088	13.5	207.345	19.75
207.3475	11.75	207.3788	19.5
207.3825	19.5	207.4138	19.25
207.4125	23.5	207.4463	20.25
207.4475	18.75	207.48	20.25
207.48	14.75	207.5163	20.75
207.5125	14	207.5463	20
207.5463	15.25	207.5788	21.5
207.58	16	207.6138	19.5
207.6175	14.25	207.645	22.5
207.65	10	207.6775	23
207.6813	13	207.715	26
207.7138	15.75	207.75	24.5
207.7463	10.5	207.7813	21
207.7775	12.25	207.8125	27
207.8088	12.75	207.845	26.5
207.8475	8	207.8788	22.25
207.8825	17.75	207.9138	20.5
207.9125	27.5	207.9463	22.25
207.9475	11.75	207.98	22
207.98	14.75	208.0163	20
208.0125	18	208.0463	22.25
208.0463	16.5	208.0788	21.5
208.08	15.5	208.1113	17.75
208.1175	12.5	208.145	19.75
208.15	10.5	208.1775	14.5
208.1813	10.75	208.215	16
208.2138	11.25	208.25	15.75
208.2463	11	208.2813	16.75
208.2775	13.25	208.3125	18.5
208.3113	12.5	208.345	20.25

208.3475	9.75	208.3788	24.5
208.3825	11.75	208.4138	18.5
208.4125	18.5	208.4463	19.5
208.4463	16	208.48	21.25
208.48	13.25	208.5163	15.75
208.5125	11.75	208.5463	18.25
208.5463	19	208.5788	18
208.58	15.75	208.6113	18
208.6175	9.75	208.645	20.25
208.65	16.75	208.6775	20
208.68	14.5	208.7125	17
208.7138	12.75	208.75	19
208.7463	12.25	208.7813	19.25
208.7775	10.5	208.8125	19
208.8113	14	208.845	23.5
208.845	10.75	208.8788	18
208.8825	13.25	208.9138	21.5
208.9125	12.25	208.9463	16
208.9463	16.25	208.98	18
208.98	12.25	209.0163	19.25
209.0125	10.5	209.0463	16.25
209.0463	15.5	209.0788	20
209.08	16.25	209.1113	20.5
209.1175	13.25	209.145	22.5
209.15	11.75	209.1775	15.25
209.18	16	209.2125	19.5
209.2138	14.75	209.25	20
209.2463	10.25	209.2813	22
209.2775	13.25	209.3125	16.75
209.3113	14.25	209.345	18
209.345	12.75	209.3788	20.5
209.3825	10.5	209.4138	27.25
209.4125	12	209.4463	22.75
209.4475	10.5	209.48	22.5
209.48	11.5	209.5163	20.75
209.5125	12.75	209.5463	18.5
209.5463	11.75	209.5788	20.75
209.5825	10.25	209.6113	18.25
209.6175	9.75	209.645	20.75
209.65	11.25	209.6775	21.25
209.68	13.5	209.7125	21
209.7138	12.75	209.75	22
209.7463	13.25	209.7813	20.5
209.7775	13.25	209.8125	20.75
209.8113	15.25	209.845	22.75
209.845	12	209.8788	21.25
209.8825	13.75	209.9138	21
209.9125	12.75	209.9463	20.25
209.9475	14.5	209.9813	21.75
209.98	12.5	210.0163	23

210.0125	11.5	210.0463	21.25
210.0463	10.25	210.0788	23.25
210.08	11.75	210.1113	22.25
210.1175	9.5	210.145	23.75
210.15	11	210.1775	27.75
210.18	10.5	210.2125	20.25
210.2138	9.75	210.25	24.25
210.2463	10.25	210.2813	22.5
210.2775	8.25	210.3125	22.25
210.3113	6.25	210.345	20
210.3438	10.25	210.3788	25.25
210.3825	9	210.4138	22.25
210.4125	8	210.4463	23.25
210.4463	10.25	210.4813	22.5
210.48	7.75	210.5163	24.75
210.5125	9.75	210.5463	24
210.5475	11.75	210.5763	25.25
210.58	7.75	210.6125	22.5
210.6175	11.25	210.645	26.5
210.65	7.5	210.6775	22.25
210.68	11	210.7125	24
210.7138	9	210.75	26
210.7463	7.75	210.7813	19.25
210.7775	8.5	210.8125	22.25
210.8113	7.5	210.845	21.75
210.8438	9.5	210.8788	22.5
210.8825	8.75	210.9138	21.5
210.9125	8.75	210.9463	21
210.9463	8.25	210.9813	20.25
210.98	11	211.0163	25.5
211.0125	11.75	211.0463	21.75
211.0475	10.75	211.0763	24.5
211.0813	10.75	211.1113	20.5
211.115	11	211.145	21.25
211.15	10.25	211.1775	20.25
211.18	10.5	211.2125	22.5
211.2138	12	211.2475	21.5
211.2463	12.5	211.2813	18.5
211.2775	9.75	211.3125	18.25
211.3113	15.75	211.345	21.75
211.345	14.25	211.3788	19.75
211.385	10	211.4138	20.5
211.415	8.75	211.4463	17.5
211.45	9	211.4813	20.75
211.4813	13.25	211.5163	19
211.5138	16.25	211.5463	18
211.5488	12.75	211.5763	20.5
211.5825	8.5	211.6138	19.75
211.6163	12.75	211.645	17
211.6513	8.75	211.6775	24.25

211.6813	10.25	211.7125	19.5
211.7163	9	211.7475	18.25
211.7488	11	211.7813	16
211.78	9.5	211.8125	17.25
211.8138	10.25	211.845	20.25
211.8475	9.75	211.8788	19.75
211.885	14	211.9138	17
211.915	16.5	211.9463	24.25
211.95	12.75	211.9813	22.25
211.9813	13.5	212.0163	20.5
212.0138	22.25	212.0463	21.5
212.0488	14.25	212.0763	20
212.0825	11.5	212.1138	22
212.1175	14	212.145	20.5
212.1513	12	212.1775	20.5
212.1813	13	212.2125	22
212.2163	12.25	212.2475	21.25
212.2488	9.5	212.2813	20.25
212.28	13.25	212.3125	18.25
212.3138	12	212.345	19.75
212.345	11.5	212.3788	16.25
212.385	9.75	212.4138	16.75
212.415	8.25	212.4463	18.25
212.4488	8.25	212.4813	18.5
212.4813	12.25	212.5138	15.5
212.5138	8.5	212.5463	16.25
212.5488	13.75	212.5763	19.5
212.5825	11.25	212.6138	19
212.6175	10.75	212.645	23.25
212.6513	9.25	212.6775	19.75
212.6813	8.75	212.7125	19.5
212.7163	9.75	212.7475	22.5
212.7488	10	212.7813	17.25
212.7825	7.25	212.8125	22.25
212.8138	16	212.845	23.75
212.845	15	212.8788	21
212.885	8.5	212.9138	23
212.915	11.5	212.9463	19.75
212.9488	10	212.9813	23
212.9813	15.25	213.0138	24.75
213.0138	20.25	213.0463	21.5
213.0488	15	213.0763	22
213.0825	10.75	213.1163	21
213.1175	10	213.1438	23.75
213.1513	13.5	213.1775	22.5
213.1813	10	213.2125	22.75
213.2163	10.25	213.2475	25
213.2488	11	213.2813	23.5
213.2825	10.25	213.3125	24.25
213.3138	10.75	213.3438	23.5

213.345	13.75	213.3788	26.25
213.3838	13	213.415	24
213.415	11.25	213.4463	22
213.4488	10.25	213.4838	25.75
213.4813	12	213.5138	24.75
213.5138	12.25	213.5463	21.75
213.5488	13.75	213.5763	21
213.5825	10	213.6138	20.25
213.6175	9.25	213.6438	23.5
213.6513	8.5	213.6775	26.25
213.6813	10.75	213.7125	20.75
213.7163	15.5	213.7475	21
213.7488	12.25	213.7813	25.75
213.7825	10	213.8125	27.25
213.815	15	213.8438	25.25
213.845	21	213.8788	22.25
213.8863	11.25	213.9138	26.5
213.915	11.5	213.9463	21
213.9488	13.75	213.9838	24
213.9813	13.75	214.0138	24.25
214.015	16.25	214.0463	24
214.0488	27.25	214.0763	25
214.0838	18.75	214.1138	24.5
214.1175	11.75	214.1438	27.25
214.1513	16.75	214.18	21.75
214.1813	18.75	214.2125	24.5
214.2163	15.75	214.2475	29.25
214.2488	14.5	214.28	27.5
214.2825	15	214.3125	22.5
214.315	18.5	214.3438	29
214.345	15	214.3788	23.25
214.3838	18.75	214.4138	26.75
214.415	11.75	214.445	20.25
214.4488	13	214.4838	25.5
214.4813	13.75	214.5138	26.25
214.5138	11.75	214.5463	27.5
214.5488	10.25	214.5763	29.25
214.5825	10.75	214.6138	28
214.6175	11	214.6438	24.75
214.6488	9.75	214.6775	34
214.6825	11.5	214.7125	23
214.7163	9	214.7475	21.25
214.7488	14.75	214.78	28.25
214.7825	10.75	214.8125	29
214.815	11	214.8438	25.75
214.845	12	214.8788	25.75
214.8838	12.75	214.9138	24.75
214.915	10.25	214.9463	24
214.9488	9.25	214.9838	21
214.9813	12.25	215.0138	28.5

215.015	12.25	215.0463	28
215.0488	11.25	215.0763	30
215.0825	10	215.1138	25.5
215.1163	9.25	215.145	29.75
215.1488	8.75	215.1775	30.5
215.1825	11.25	215.2125	24.75
215.2163	10.25	215.2475	20.75
215.2488	13.25	215.28	24.75
215.2825	11.25	215.3125	24
215.315	10.75	215.3438	24.5
215.345	7.5	215.3788	23.75
215.3838	12.75	215.4113	23
215.415	8.5	215.4463	24.25
215.4488	8.25	215.4838	22.75
215.4813	12.5	215.5138	20.75
215.515	12	215.5463	24.5
215.5488	10.75	215.5763	25.5
215.5825	9.25	215.6138	24.25
215.6188	9	215.6438	21.75
215.6488	10	215.6775	26.75
215.6825	12.25	215.7125	20
215.7163	10.25	215.7475	24.5
215.7488	11	215.78	22.75
215.7825	13.25	215.8125	22.5
215.815	14.25	215.8438	23.75
215.8475	12.25	215.8788	22.75
215.8838	13.5	215.9125	28.75
215.915	10.75	215.9463	26
215.9488	10.25	215.9838	20.75
215.9813	14.5	216.0138	23
216.015	9	216.0438	27.25
216.0488	12.75	216.0763	25.5
216.0825	14	216.1138	20.75
216.1163	12	216.1438	27.75
216.1488	11.5	216.1775	26.75
216.1825	8.5	216.2125	30.5
216.2163	9.75	216.2475	22
216.2488	11.25	216.28	28.25
216.2825	9.25	216.3125	23
216.315	9.75	216.3438	22.75
216.3475	12.25	216.3788	22.5
216.3838	12.5	216.4125	24.5
216.4138	13	216.4463	21.5
216.4488	12.25	216.4838	24.5
216.4813	14	216.5138	22.75
216.515	12.75	216.5438	23.75
216.5488	10.75	216.5763	23.25
216.5825	14.75	216.6113	23.25
216.6163	15.25	216.6438	23.5
216.6488	11.75	216.6775	27.5

216.6825	19.75	216.7125	21.5
216.7163	13.75	216.7475	23.75
216.7488	18.25	216.78	26.75
216.7825	15.75	216.8125	21.75
216.815	14.75	216.8438	25.25
216.8475	15	216.8788	28.75
216.8838	13	216.9113	22.75
216.9138	14	216.9463	28.75
216.9488	11.25	216.9838	24
216.9813	11.75	217.0138	23.25
217.015	11.75	217.0438	24.5
217.0513	12.5	217.0763	27
217.0825	11	217.1113	29
217.1163	11	217.1438	23.5
217.1488	9.5	217.1775	24.75
217.1825	13.75	217.2125	29
217.2163	14	217.2475	26.75
217.2488	12.75	217.28	27.75
217.2825	14.25	217.3125	23.5
217.315	18.5	217.3438	25.25
217.3475	12.25	217.3788	23.5
217.3838	14.75	217.4113	23.5
217.415	13.25	217.4463	21.25
217.4488	13.25	217.4838	19.25
217.4813	12.5	217.5138	22.25
217.515	13.25	217.5463	20.25
217.5513	14.5	217.5763	15.75
217.5825	9	217.6113	18.25
217.6163	11.5	217.6438	16.25
217.6488	13.25	217.6763	19
217.68	13.25	217.7125	22.75
217.7163	12.5	217.7475	21.75
217.7513	10	217.78	18
217.7813	12.75	217.8125	20.5
217.815	12.25	217.8438	24
217.8475	10.25	217.8788	22.5
217.8813	12	217.9113	20.75
217.9138	9.25	217.9463	23.5
217.9488	14	217.9838	24.25
217.9813	12.25	218.0138	24
218.015	9.75	218.0438	23.25
218.0513	10.5	218.0763	26.5
218.0838	11	218.1113	23.5
218.1163	8	218.1463	21.25
218.1488	9.75	218.1763	25
218.18	11.75	218.2125	19.75
218.2188	9.25	218.2475	25.75
218.2513	11.5	218.28	24.25
218.2813	13	218.3113	20
218.315	11.5	218.3438	23.5

218.3475	8
218.3813	11.25
218.4138	11.5
218.4488	10.5
218.4813	10.75
218.515	8.75
218.5513	10.75
218.5838	11.25
218.6163	9.75
218.6488	13.75
218.68	16
218.7163	11.75
218.7513	13
218.7813	12
218.815	13
218.8475	16.25
218.8813	12.5
218.9138	10.25
218.9513	12.5
218.9813	12.25
219.015	11.75
219.0513	10.5
219.0838	9
219.1163	12
219.1488	9.25
219.18	9.75
219.2163	10
219.2513	14.5
219.2813	15.25
219.3175	14
219.3475	12
219.3813	12.75
219.4138	16
219.4488	11
219.4813	16.5
219.515	13.75
219.5513	16.25
219.5838	15.75
219.6163	12.5
219.6488	13.75
219.68	13.75
219.7163	17.5
219.7513	17
219.7813	17.75
219.8175	12.75
219.8475	11.75
219.8813	14.5
219.9138	12
219.9488	15
219.9813	14.25

218.3788	25
218.4125	26.5
218.4463	22.75
218.4838	25.5
218.5138	26.25
218.5438	27
218.5763	29
218.6113	29
218.6438	27
218.6763	26.5
218.7125	22
218.7475	22.75
218.78	24.25
218.8113	25.75
218.8438	20.5
218.8813	30.75
218.9125	24.5
218.9463	25.25
218.9813	22.5
219.0138	26
219.0438	25.5
219.0763	24
219.1113	24.5
219.1438	26.75
219.1763	25.75
219.2125	22.75
219.2475	23.5
219.28	24.25
219.3113	25
219.3438	23.5
219.3788	21.75
219.4125	25.25
219.4463	20.5
219.4813	21
219.5138	23.25
219.5438	22.5
219.5763	25.75
219.6113	22.75
219.6438	23.5
219.6763	20.5
219.7125	24.75
219.7488	21.5
219.78	25
219.8113	18.75
219.8438	21.25
219.8763	18.5
219.9125	26.5
219.9438	25.5
219.9813	19.5
220.0138	17.75

220.015	12	220.0438	18.75
220.0513	13	220.0763	21.25
220.0838	9.75	220.1113	20
220.1163	9.75	220.1438	23.75
220.1488	9.5	220.1763	19
220.18	11.25	220.2125	21.75
220.2163	11.25	220.2475	22.75
220.2513	9.5	220.28	36.25
220.2838	8.5	220.3113	24.5
220.3175	10	220.3438	28
220.3475	5.75	220.3763	20.5
220.3813	11	220.4125	21.75
220.4138	11	220.4425	21.25
220.4488	7.5	220.4813	28
220.4813	9.5	220.5138	20.25
220.515	9	220.5438	25.5
220.5513	8.25	220.5763	27.5
220.5838	9.5	220.6113	53
220.6163	10.25	220.6438	46.5
220.6488	13.25	220.6763	40.5
220.68	12.25		
220.7138	13.5	220.7475	34.25
220.7513	13	220.78	30
220.7838	7.25	220.8113	28
220.8175	11	220.8438	32.25
220.8475	14.75	220.8763	33.25
220.8813	11.75	220.9125	40.5
220.9138	13.25	220.9425	24.75
220.9488	11	220.9813	36.25
220.9838	14.75	221.0138	48.25
221.015	11.25	221.0438	24.75
221.0488	14	221.0738	28
221.0838	13.75	221.1113	28.75
221.1163	14.25	221.1463	27.25
221.1488	13.25	221.1763	24.5
221.18	12.25	221.2125	40
221.2138	14.25	221.2475	32.75
221.2513	16.25	221.28	41.5
221.2838	14.5	221.3113	34.5
221.3175	14	221.3438	35.75
221.3475	14.5	221.3763	34.5
221.3813	14	221.4125	39.75
221.4138	12.75	221.4425	20.75
221.4488	13.25	221.4813	27.25
221.4838	14	221.5138	29.5
221.515	12.25	221.5438	26
221.5488	11.5	221.5738	20.75
221.5838	11.5	221.6113	33
221.6163	11	221.6463	24.75
221.6475	10.75	221.6763	20.5

221.68	12	221.7125	27.25
221.7138	12.5	221.7488	26
221.7513	16.75	221.78	21.75
221.7838	11	221.8113	25.75
221.8175	11.25	221.8438	24
221.8475	11.5	221.8763	26.75
221.8813	11.5	221.9125	28.25
221.9138	14	221.9425	25.25
221.9488	14.25	221.9813	37.75
221.9825	14.25	222.0138	30.75
222.015	16	222.0438	24.25
222.05	11.5	222.0738	28.75
222.0838	12.75	222.1113	40.25
222.1163	14	222.1463	24.5
222.1475	11.75	222.1763	43.5
222.18	15	222.21	28.75
222.2138	11.5	222.2463	39
222.2513	9.25	222.28	26.5
222.2863	12.75	222.3113	26.25
222.3175	9.25	222.3438	27.25
222.3475	12.75	222.3763	32.75
222.3813	12.5	222.4125	26
222.4138	12.5	222.4425	37.75
222.4488	12.25	222.4813	40.25
222.4825	16.75	222.5138	30.25
222.5163	16.75	222.5438	32.25
222.55	14	222.5738	30.75
222.5838	14.25	222.6113	26.25
222.6163	10.25	222.6463	25.75
222.6475	13	222.6763	26.25
222.68	13	222.71	21.25
222.7138	9.25	222.7463	27.5
222.7513	11.5	222.78	25.5
222.7838	11.25	222.8113	34.75
222.8175	11	222.8438	37.75
222.8475	14.25	222.8763	27.75
222.8813	12	222.9125	37.25
222.9138	13.75	222.9425	35
222.9488	11.5	222.9813	25.25
222.9825	13	223.0138	33.25
223.0163	9.5	223.0463	28.5
223.05	14.75	223.0738	29
223.0838	11.75	223.1113	31
223.1163	17.5	223.1463	25.25
223.1475	19.25	223.1763	27
223.18	15.25	223.21	30.5
223.215	13.5	223.2463	24.75
223.2488	14.25	223.28	26.25
223.2838	14	223.3113	28
223.3175	15.25	223.3438	29.75

223.3475	12.25	223.3763	25
223.3813	17.75	223.4125	24.75
223.4138	15.25	223.4425	25
223.4488	15	223.4813	24.25
223.4825	11.25	223.5138	25.25
223.5163	11.25	223.5438	27.5
223.55	14	223.5738	22.75
223.5838	13	223.6113	23
223.6163	17	223.6463	23
223.6475	15.25	223.6763	28.75
223.68	13.75	223.71	23.5
223.7138	15	223.7463	28.25
223.7488	14.75	223.78	23.25
223.7838	15.25	223.8113	19.5
223.8163	12.25	223.8438	27
223.8475	16.75	223.8763	25.25
223.8813	12.5	223.9125	25.25
223.9138	9.75	223.9425	25.5
223.9488	13.25	223.9813	25.5
223.9825	16.5	224.0138	21.75
224.0163	14.75	224.0438	23.5
224.05	16.5	224.0738	23.5
224.0838	15.75	224.1113	20.25
224.1163	14.25	224.1463	18.5
224.1475	15.25	224.1763	21
224.18	15.25	224.21	20.5
224.2138	14	224.2463	21.25
224.2488	16	224.28	21.25
224.2863	15.5	224.3113	20.75
224.3163	15	224.3438	20.75
224.3475	15	224.3763	25
224.3813	18.25	224.4125	23.75
224.4163	12	224.4425	18.25
224.4488	9.75	224.48	21.25
224.485	11	224.5113	21.5
224.5138	12.5	224.5438	22.25
224.55	9.5	224.5738	23
224.5838	11.75	224.6113	21.25
224.6163	7.75	224.6463	21
224.6475	13.5	224.6763	20.75
224.68	13.5	224.71	21
224.7138	11.25	224.7463	18.25
224.7488	9.75	224.78	20.25
224.7863	12.75	224.8113	21.5
224.8163	14.5	224.8438	18.5
224.8475	13	224.8763	18.5
224.8813	14.5	224.9125	19.25
224.9163	13.5	224.9425	17.75
224.9488	10.5	224.98	17.5
224.9825	11.25	225.0113	19

225.0138	10.25
225.05	10.25
225.0838	17
225.1163	16
225.1475	11.5
225.18	9.75
225.215	13.25
225.2513	14.5
225.2863	11.25
225.3163	14.5
225.3475	15
225.3813	12.25
225.4138	10.75
225.4488	11.75
225.4825	12.75
225.5138	22.75
225.55	26
225.5838	13.75
225.6163	14
225.6475	11.5
225.68	9.75
225.715	12.25
225.7513	11.5
225.7863	10
225.8163	9.5
225.8475	10.75
225.8825	11.75
225.9138	17.5
225.9488	15.5
225.9825	11.75
226.0138	11.5
226.05	15.25
226.0813	21.75
226.1163	19.25
226.1475	16
226.18	7
226.215	19.5
226.2513	20.5
226.285	6.5
226.3163	11.75
226.3475	9.25
226.3813	8.75
226.4138	13.75
226.4488	11
226.4825	12.5
226.5163	9
226.55	13
226.5813	10.5
226.6163	11.5
226.6475	10

225.0438	19.25
225.0738	25.5
225.1113	21.5
225.1463	24
225.1763	23.5
225.21	24.75
225.2463	20.5
225.2788	20.75
225.3113	23.5
225.3438	23.25
225.3763	21
225.4125	26.75
225.4425	25.75
225.48	22.25
225.5113	22.5
225.5438	24.5
225.5738	22.75
225.6113	21.75
225.6463	21.25
225.6763	28
225.71	25
225.7463	23.25
225.7788	24.5
225.8113	28.75
225.8438	22.5
225.8763	25.75
225.9125	24.5
225.9425	21.75
225.98	18.5
226.0113	24.5
226.0438	24.25
226.0763	22
226.1113	27.25
226.1463	25
226.1763	25.5
226.21	22.75
226.2463	21
226.2788	22.25
226.3113	22
226.3438	23
226.3775	23.25
226.4125	20.75
226.4425	22.5
226.48	23.5
226.5113	18.5
226.5438	23
226.5763	19.25
226.6113	20.5
226.6463	17.75
226.6763	19

226.68	15.75	226.7075	21
226.715	14.25	226.7463	22.25
226.7513	12	226.7788	22.25
226.785	13.5	226.8113	22.25
226.8163	11.5	226.8438	20.5
226.8475	16	226.8775	23
226.8813	15.25	226.9125	19
226.9138	16.25	226.9425	16.75
226.9488	11.25	226.98	18
226.9825	11.25	227.0113	17.75
227.0188	15.5	227.0413	20
227.0525	13.25	227.0763	17.75
227.0825	11.25	227.1113	19.25
227.1175	12.5	227.1463	17
227.1488	13.5	227.1763	17
227.185	10.25	227.2075	18
227.2163	11.25	227.2438	23
227.2525	9.25	227.2788	18.75
227.2863	9.25	227.3113	18.75
227.3188	10	227.3438	16.25
227.35	12	227.3775	16.5
227.3838	8.25	227.4125	18.75
227.4175	11.25	227.4425	17.25
227.4513	9.75	227.48	19.5
227.485	9.25	227.5113	18.25
227.5188	8.75	227.5413	19.5
227.55	7.5	227.5763	19
227.5825	9	227.6113	23
227.6175	13	227.6463	22
227.6488	8.25	227.6763	22.75
227.685	9.25	227.7075	18.5
227.7163	11.5	227.7438	23.75
227.7525	8.5	227.7788	17
227.7863	10.75	227.8113	22
227.8188	10	227.8463	25
227.8488	11.25	227.8775	20.5
227.8838	10.25	227.9125	21
227.9175	10.75	227.9425	22.75
227.9513	9.25	227.98	24.25
227.9863	12.25	228.0113	21.75
228.0188	11.5	228.0413	25
228.05	10.75	228.0763	25.75
228.0825	11.25	228.1113	27.25
228.1175	10.25	228.1463	25.75
228.1488	10.25	228.1763	18.75
228.185	10.5	228.2075	24.25
228.2163	9.75	228.2438	17.5
228.2525	10	228.2788	19.5
228.2863	9.5	228.3113	18.5
228.3188	10.75	228.3475	23.5

228.3488	10.25	228.3775	23
228.3838	13	228.4125	21.5
228.4175	12.25	228.4425	18.5
228.4513	10.5	228.48	22.75
228.4863	13.75	228.5113	21.75
228.5188	14	228.5413	17.75
228.55	12.25	228.5763	19.5
228.5825	13	228.6113	15.75
228.6175	11.25	228.6463	18
228.6488	12.25	228.6763	18
228.685	11.5	228.7075	20.5
228.7163	12.5	228.745	23
228.7525	13.75	228.7788	22
228.7863	11.5	228.8088	19.5
228.8188	13.25	228.845	29.25
228.8488	12.75	228.8775	24
228.8838	13	228.9125	25
228.9175	12.25	228.9425	26
228.9513	14.5	228.98	20.75
228.9863	11.25	229.0113	20.75
229.0188	13	229.0413	20.25
229.05	12.75	229.0763	20.5
229.0825	11.25	229.1113	23
229.1175	12.25	229.1463	23.25
229.1488	12	229.1763	23.75
229.185	9.75	229.2075	18.75
229.2163	11.25	229.2438	20.25
229.2525	10.5	229.2788	19.25
229.2888	11.5	229.3088	18
229.3175	13.25	229.3463	21
229.3488	13	229.3775	17.5
229.3838	11.75	229.4125	20.5
229.4163	14.25	229.4425	19.25
229.4513	10.5	229.48	17
229.4863	13	229.5113	18.5
229.5188	13.25	229.5438	16.25
229.55	11.75	229.5763	20.75
229.5825	9.5	229.6113	18.25
229.615	12.5	229.6463	19.25
229.6488	12.5	229.6763	21.5
229.685	13.5	229.7075	22
229.7163	13.75	229.7438	19.5
229.7538	11.25	229.7788	21.75
229.7863	11.75	229.81	22.25
229.8175	11	229.845	23.5
229.8488	12.75	229.8775	23.25
229.8838	15.75	229.9125	23.75
229.9163	16.75	229.9425	18
229.9513	11.75	229.9775	20.75
229.9863	11.5	230.0113	22.75

230.0188	13.75	230.0413	20.5
230.05	16.5	230.075	22.5
230.0825	13.75	230.1113	24.5
230.115	13	230.1463	26
230.1488	12.5	230.1763	23
230.185	12.5	230.2075	22.25
230.2163	12.25	230.2438	24.25
230.2538	13.5	230.2788	24.5
230.2863	14	230.31	17.25
230.3175	10.25	230.345	20
230.3488	15	230.3775	20.5
230.3838	17.25	230.4125	24.5
230.4175	13.5	230.4425	19.75
230.4513	13.5	230.4775	19.25
230.4863	15	230.5113	20.5
230.5188	11.75	230.5413	18
230.55	15.25	230.5775	20.75
230.5825	16.25	230.6113	18.25
230.615	12.5	230.6463	19.75
230.6488	11.75	230.6763	18.75
230.685	17	230.7075	19.5
230.7163	15.5	230.7438	17.75
230.7538	12	230.7788	17.5
230.7863	11.5	230.81	19
230.8175	17.25	230.845	20.5
230.8488	14	230.88	20.75
230.8838	11.5	230.915	21.25
230.9175	16.5	230.945	21
230.9513	13	230.98	18.25
230.9863	13.75	231.0138	17.5
231.0188	14.5	231.0438	22.25
231.05	10.25	231.0775	19.75
231.0825	12.25	231.1125	19
231.115	16.5	231.1475	23.5
231.1488	12.75	231.1788	21.5
231.185	12.5	231.2113	20.75
231.2188	13	231.245	20.25
231.2538	13.5	231.28	22.25
231.2863	11.75	231.3113	19
231.3175	10.5	231.3475	21.5
231.3488	13.5	231.38	19.25
231.3838	11.75	231.415	21
231.4175	13.5	231.445	21.5
231.4513	14.25	231.48	18.5
231.4863	11.75	231.5138	21.25
231.5188	10.25	231.5438	22.5
231.5525	11.5	231.5775	20.5
231.5825	13.25	231.6125	27.25
231.615	12	231.6475	22.75
231.6488	10.25	231.6775	20.25

231.685	15	231.7113	20.25
231.7188	15.5	231.745	20.25
231.7538	14.25	231.78	19.5
231.7863	12.25	231.8113	17
231.8188	15	231.8475	17.25
231.8488	12	231.88	18.25
231.8813	14.25	231.915	15.75
231.9175	16.5	231.95	17.25
231.9513	15.5	231.98	18
231.9863	13.5	232.0138	18
232.0188	12.25	232.0438	19.75
232.05	13.25	232.0775	20.75
232.08	11.25	232.1125	20.5
232.115	11.25	232.1463	21.75
232.1488	13.75	232.1775	23.75
232.185	17.75	232.2113	21.25
232.2188	14.25	232.245	24.25
232.2538	10	232.28	19.5
232.2863	10.5	232.3113	21.75
232.3175	10.75	232.345	22
232.3488	10.5	232.38	22.25
232.3813	13.75	232.415	24.5
232.4175	11.5	232.4463	22
232.4513	10	232.48	21.5
232.4888	10.75	232.5138	22.75
232.5188	10.5	232.5438	20.5
232.55	13.25	232.5775	21.5
232.58	14	232.6125	18.5
232.615	12	232.6463	17.25
232.6513	11	232.6775	17.75
232.685	18.75	232.7113	18
232.7188	20.75	232.7438	18.5
232.7538	10	232.78	16.5
232.7863	12	232.8113	18.75
232.8175	17	232.845	19.25
232.8488	13.5	232.88	19.25
232.8813	11.75	232.915	20.25
232.9175	13	232.9463	22
232.9513	11.25	232.98	18.5
232.9888	11.5	233.0138	16
233.0188	10.25	233.0463	21.75
233.05	9.75	233.08	18.75
233.08	13.25	233.1125	18.25
233.115	15.5	233.1463	16.75
233.1513	10.25	233.1775	13.5
233.1825	9.75	233.2113	17.75
233.2213	12.75	233.2438	17.75
233.2538	13.5	233.28	14.25
233.2863	10.5	233.3113	16.5
233.3175	16	233.345	14

233.3488	17.25	233.38	16.25
233.3813	11.75	233.415	18.5
233.4175	14.75	233.4463	18.25
233.4513	11.75	233.48	15.25
233.4888	10.75	233.5138	16
233.5188	11.75	233.5463	18.5
233.55	13.25	233.58	19.25
233.58	13.5	233.6125	14.75
233.615	14.5	233.6463	13.75
233.6513	9.75	233.6775	18.75
233.6825	12.5	233.7113	19.75
233.7188	14	233.7463	19.75
233.7538	11.25	233.78	15.75
233.7863	11.5	233.8113	19.25
233.8175	13	233.845	16.5
233.8488	9.5	233.88	13.75
233.8813	13.25	233.915	18.75
233.915	15.25	233.9463	18
233.9513	17	233.98	18.75
233.9888	16	234.0138	19.5
234.0213	11.25	234.0463	20.5
234.05	10.5	234.08	20.5
234.08	13	234.1125	17.25
234.115	9.5	234.1463	18.5
234.1513	8.5	234.1775	17.25
234.1825	8.25	234.2113	21.5
234.2188	9.5	234.2438	19.5
234.2538	8.5	234.28	16
234.2863	12.25	234.3113	19.75
234.3175	8.75	234.3475	21.5
234.3488	11	234.38	18.5
234.3825	23.5	234.415	20.25
234.415	13.5	234.4463	24
234.4513	18	234.48	21.5
234.4888	21	234.5138	21.25
234.5188	9.5	234.5463	22
234.55	13.5	234.58	20.75
234.58	18.75	234.6125	17.25
234.615	12	234.6463	23.25
234.65	16.5	234.68	23
234.6825	9.5	234.7113	24.25
234.7188	12.5	234.7438	23
234.7538	10.25	234.78	25
234.7863	13.75	234.8113	20.25
234.8175	10.5	234.845	25.75
234.8488	11	234.88	23
234.8825	14.25	234.915	21.5
234.915	11.5	234.9463	24.25
234.9513	10.75	234.98	19.75
234.9888	26.25	235.0138	27.25

235.0188	14	235.0463	25.5
235.05	11.25	235.08	19
235.08	13.5	235.1125	18.5
235.115	15.5	235.1475	24
235.15	12.75	235.18	19.75
235.1825	12.75	235.2113	18.25
235.22	12.5	235.2438	22
235.2538	17.5	235.28	24.75
235.2863	12.25	235.3113	24
235.3175	12.5	235.345	18.25
235.3488	12.25	235.38	24.25
235.3825	13.75	235.415	23.25
235.415	12.5	235.4463	21
235.4513	13	235.48	22.25
235.4888	8.25	235.5113	21.25
235.5188	13	235.5475	23.5
235.55	12.75	235.58	21.5
235.58	10.25	235.6113	21.75
235.6175	13	235.6463	24
235.65	12.25	235.68	23.5
235.6825	11.5	235.7113	23.5
235.72	10.75	235.7438	20.25
235.7538	12.25	235.78	22.25
235.7863	12.5	235.8113	17.5
235.8175	9.5	235.845	22.5
235.8488	12.5	235.88	22.5
235.8825	13.25	235.915	27.75
235.915	12	235.9463	23.25
235.9513	9	235.98	18
235.9888	14.5	236.0113	19.5
236.0188	11	236.0463	23.25
236.05	12	236.08	20.25
236.08	12	236.1113	24.5
236.1175	12.5	236.1463	22
236.15	11.5	236.18	22.75
236.1825	17.25	236.2138	24
236.22	14	236.2463	27.25
236.2538	15	236.2813	27.25
236.2863	15	236.3113	21.5
236.3175	15.75	236.345	24.25
236.3488	15.5	236.38	25.5
236.3825	14	236.4163	28.25
236.4175	13.75	236.4463	25.75
236.4525	14.75	236.4813	25.25
236.4913	17.75	236.5138	27.75
236.5188	17.25	236.5488	22.75
236.55	15	236.5825	26.75
236.58	14	236.6125	23
236.6175	14	236.6488	21.5
236.65	14	236.6813	24

236.6825	17.75	236.7138	23.75
236.72	15	236.7525	22.5
236.7538	12.25	236.7825	23
236.7863	11.75	236.8138	25.5
236.8188	13.25	236.8475	25.5
236.8488	10.75	236.8825	26.75
236.8825	13.25	236.9188	28.75
236.915	14	236.9488	26.5
236.95	13	236.9838	30.25
236.9888	12.5	237.0138	26.25
237.0188	16.25	237.0488	25.25
237.05	13.5	237.0825	25.25
237.0825	11.75	237.1125	25.5
237.1175	13.5	237.1488	27.75
237.15	16.5	237.1813	26
237.1825	16.75	237.2138	22
237.22	13.5	237.2475	24.25
237.2538	14.25	237.2825	27
237.2863	11.5	237.3138	26
237.3175	14.5	237.3475	24.75
237.3513	11	237.3825	27.5
237.3825	11.5	237.4188	30.75
237.415	10.5	237.4488	26.5
237.45	13	237.4838	26
237.4888	14.25	237.5138	26.25
237.5188	11	237.5488	26.25
237.55	16.5	237.5825	28.75
237.5825	13.5	237.6125	22.25
237.6175	14.5	237.6488	21.75
237.65	15.5	237.6813	19.5
237.6825	14	237.7138	25.75
237.72	16.25	237.7475	24.5
237.7538	16.75	237.7813	20.25
237.7863	13	237.8138	26.5
237.8175	16.75	237.8475	24.75
237.8513	17.25	237.8825	22
237.8825	19.25	237.9188	26.25
237.9125	16.25	237.9488	21.25
237.9525	15	237.9838	22
237.9888	20.5	238.0138	28.5
238.0188	15.5	238.0488	24
238.05	18.75	238.0825	25.25
238.0825	12.25	238.1125	19.5
238.1175	17.5	238.1513	25.75
238.15	13.25	238.1813	22.75
238.1825	11.5	238.2138	24
238.22	14.25	238.2475	22
238.2538	10.25	238.2813	24
238.2863	12.25	238.3138	23
238.3175	10.5	238.3475	28.75

238.3513	11.5	238.3825	23.75
238.3825	11.75	238.4188	23.25
238.4125	11.5	238.4488	21.75
238.4525	13.25	238.4838	23
238.4888	11	238.5138	25.25
238.5188	12.75	238.5488	25.25
238.55	15.25	238.5825	23.75
238.5825	10	238.6125	24.5
238.6175	12.25	238.6513	24
238.65	11	238.6813	25
238.6825	9.75	238.7138	22.5
238.72	13.25	238.7475	27
238.7538	13.5	238.7813	26.5
238.7863	12	238.8138	26.75
238.8175	16.5	238.8475	26
238.8513	10.25	238.8825	22.25
238.8825	11.25	238.9188	26.75
238.9125	12.5	238.9488	25.75
238.9525	13.5	238.9838	28.75
238.9888	14.75	239.0138	24.25
239.0188	15.25	239.0488	25.5
239.05	11.75	239.0825	25.5
239.0838	12.5	239.1125	25
239.1175	11	239.1513	19.5
239.15	12	239.1813	23.25
239.1825	10	239.215	21.5
239.2225	13.25	239.2475	22.5
239.2538	10.25	239.2813	21.25
239.2863	12.25	239.3138	22.25
239.3175	16.5	239.3475	27.75
239.3538	11	239.3838	23.25
239.3825	10.5	239.4188	22.5
239.4125	12.75	239.4488	21.75
239.4525	14.75	239.4838	23.5
239.4888	11	239.5138	27.25
239.5188	12.5	239.5463	25.5
239.55	12.25	239.5825	26.75
239.5838	11.5	239.6125	24.5
239.6175	13	239.6513	21.5
239.65	10.75	239.6813	25.25
239.6825	11	239.715	28.75
239.7188	12.5	239.7475	24
239.7538	10.25	239.7813	26
239.7863	10	239.8138	24.5
239.8188	11.25	239.8475	22
239.8513	11	239.8838	23.25
239.8825	13	239.9188	21.5
239.9125	12.5	239.9488	21.5
239.9525	11.25	239.9863	27
239.9888	11.25	240.0163	19.5

240.0188	11	240.0463	25.75
240.05	11.5	240.0825	24.5
240.0838	11	240.1125	23.5
240.1175	12.75	240.1513	22.5
240.15	14.25	240.1813	28
240.1838	14	240.2175	27.25
240.2188	13	240.2475	24.25
240.2538	13.75	240.2813	25
240.2863	16	240.3138	23.75
240.3188	14.25	240.3475	21.75
240.3513	15.75	240.3838	26
240.3825	14.75	240.4188	28.5
240.415	15	240.4488	26.25
240.4525	17.5	240.4838	26.75
240.4888	13.25	240.5163	26.25
240.5188	12.5	240.5488	23.25
240.55	15.5	240.5825	28.75
240.5838	15	240.615	24.25
240.6175	13.5	240.6513	24.5
240.65	14.5	240.6813	26.25
240.6838	13.5	240.715	22.5
240.7188	14.25	240.7475	21.5
240.7538	10.5	240.7813	21.5
240.7863	18	240.8138	23.75
240.8188	17.75	240.8475	27.25
240.8513	11.75	240.8838	26.25
240.8825	12	240.9188	27.25
240.9125	12.25	240.95	22
240.9525	10.25	240.9838	21.25
240.9888	13.5	241.0163	22.5
241.0188	12	241.0488	18.5
241.05	12	241.0825	23.75
241.0838	11.75	241.1175	20.5
241.1175	12	241.1513	18
241.15	13.5	241.1813	18.25
241.185	13.25	241.215	16.75
241.2188	12.75	241.2475	19
241.2538	13.5	241.2813	18.75
241.2863	10.75	241.3125	15.25
241.3213	14.25	241.3475	16.25
241.3513	13.25	241.3838	19
241.3825	11.5	241.4188	16.25
241.4125	14.5	241.4538	19.25
241.4525	13.25	241.4838	21.5
241.4888	13.75	241.5163	20.5
241.52	14	241.55	19
241.5525	13.75	241.5825	20.25
241.5838	12.25	241.615	19.5
241.6175	10.75	241.6513	21.25
241.65	19	241.6813	16

241.685	12.25	241.715	19.5
241.7188	13.25	241.7475	34
241.7538	10.5	241.7813	39.25
241.7863	13.75	241.8125	34
241.8213	15.5	241.8488	38.75
241.8513	12.75	241.8838	21.5
241.8825	11	241.9188	33.75
241.9125	11.25	241.9513	33.75
241.9525	10	241.9838	34
241.9888	14.25		
242.0188	8	242.0488	46.5
242.0525	12.75	242.0825	40.25
242.0838	10.75	242.115	37.5
242.1175	13	242.1538	45.5
242.15	10	242.1813	19.75
242.185	11.25	242.215	19.75
242.2175	9.5	242.2475	19.75
242.2538	10	242.2813	18
242.2863	9.75	242.3125	40.25
242.3213	11.25	242.3488	43.25
242.3513	9	242.3838	27
242.3825	10.75	242.4188	37.5
242.415	9.5	242.4513	44.5
242.4525	8.5		
242.4888	10.25	242.5188	43.25
242.5188	9.75	242.5488	30.25
242.5525	10.75	242.58	53.5
242.5838	9	242.615	47
242.6175	7	242.6513	42
242.65	9.5	242.6813	21.25
242.685	10	242.715	17
242.7175	10.25	242.7475	15.75
242.7538	8.75	242.7813	22.5
242.7863	9.75	242.8125	24.75
242.8225	8.75	242.8488	15
242.8513	6.5	242.8838	21
242.8825	7.25	242.9188	25.5
242.915	9.75	242.9513	36.5
242.9525	8.75	242.9838	23
242.9863	6	243.0163	22.75
243.0188	6.75	243.0488	35.75
243.0525	9.5	243.0825	17.5
243.0838	7.5	243.115	31
243.1175	10	243.1513	36.5
243.15	8.25	243.1838	21
243.185	7.25	243.215	18.75
243.2175	7	243.25	19.25
243.2538	7	243.2825	18
243.2863	7	243.3125	25.25
243.3213	7.25	243.3488	23.25

243.3513	9.75
243.3825	10.75
243.415	7
243.4525	6.75
243.4863	8.25
243.5188	6.25
243.5538	9
243.5863	9.75
243.6175	11.25
243.65	9.25
243.685	10.5
243.7175	9.5
243.7538	11.75
243.7863	10.5
243.8213	10.75
243.8513	10.75
243.8825	12
243.915	15.5
243.95	10.5
243.9863	12.25
244.0188	10.25
244.0538	12.5
244.0838	16.5
244.1175	11.75
244.1513	10.25
244.185	11.75
244.2175	9.75
244.2538	9.75
244.2863	9
244.3213	10
244.3513	10.5
244.3825	10
244.415	10.5
244.45	10.75
244.4863	10.25
244.5188	10
244.5538	8.5
244.5838	11.75
244.6175	15
244.6513	11.5
244.685	11
244.7175	15
244.7538	15.75
244.7875	20
244.8213	11.25
244.8513	16
244.8825	13
244.915	14
244.95	16.25
244.9863	13.75
243.3838	14
243.4188	15.25
243.4513	12.5
243.4838	19.75
243.5163	16
243.5488	16.25
243.5825	19.25
243.615	18.75
243.6538	16.5
243.6838	15.25
243.715	17.25
243.75	21
243.7825	17.5
243.8125	16.75
243.8488	15
243.8838	13.75
243.9188	11.25
243.9513	16.25
243.9838	13.25
244.0163	14.5
244.0488	18
244.0825	16
244.115	15.75
244.1513	15
244.1838	16
244.215	12.25
244.25	16.75
244.2825	18.75
244.3175	16
244.3475	16.5
244.385	16.5
244.4225	21.75
244.4525	15.75
244.4863	16
244.5175	21
244.55	15
244.585	15.25
244.6175	18
244.6538	18
244.6863	16.25
244.7175	19.75
244.755	19.25
244.785	19.75
244.8175	15
244.8475	18
244.885	17.25
244.9225	14.75
244.9525	16.75
244.985	15.75
245.02	18.25

245.0188	14	245.05	16.5
245.0538	13.75	245.085	18.5
245.0838	14.5	245.1175	18.25
245.1175	10.25	245.1538	17
245.1513	13	245.1863	17.5
245.185	11	245.2175	16.5
245.2175	9.75	245.2525	18.5
245.2538	15.25	245.285	20
245.2875	11.75	245.3175	20
245.3213	14	245.3475	19.25
245.3513	15.75	245.385	21.75
245.385	12.75	245.4225	17.5
245.415	10.25	245.4525	20.5
245.45	12.5	245.4888	22.5
245.4863	11.75	245.5213	25.25
245.5188	12.5	245.5525	22.5
245.5538	15	245.5863	20.75
245.5838	11	245.6188	21
245.6175	12.75	245.6563	21.25
245.6513	11.5	245.6888	23.75
245.685	13	245.72	20.75
245.7175	9.25	245.7588	18.5
245.7538	10.5	245.7875	23.25
245.7875	12.25	245.82	17.75
245.8213	10.25	245.85	21.75
245.8513	8.5	245.8875	25
245.885	8.25	245.9238	22.5
245.915	10.75	245.9538	19.5
245.95	9	245.9888	24
245.9863	8.25	246.0213	19.25
246.0188	7.5	246.0525	20.5
246.0538	9.5	246.0863	15
246.0838	9.75	246.1175	21.5
246.12	6.5	246.1563	20.25
246.1513	5	246.1888	21.75
246.185	8.75	246.22	22.75
246.2175	9.25	246.255	21.25
246.2538	7.25	246.2875	23.5
246.29	9.75	246.32	22.5
246.3213	12.5	246.3513	17.5
246.3513	10	246.3875	21
246.385	9.75	246.4238	26.25
246.4175	12.75	246.4538	25.5
246.45	14	246.4888	27
246.4863	12.75	246.5213	23.25
246.5188	12.25	246.5525	24.5
246.5538	13	246.5863	28.25
246.5838	11.75	246.6175	25.25
246.62	10.75	246.6563	21.5
246.6513	15.25	246.6888	20.5

246.685	11	246.72	20
246.7175	12.25	246.7575	26
246.7563	11.75	246.79	20
246.79	13.25	246.82	21
246.8213	11.75	246.85	23.25
246.8513	14.75	246.89	26.25
246.885	12	246.9238	21.5
246.9175	11.25	246.9538	24.5
246.95	11.5	246.9888	22
246.9863	11.5	247.0213	24
247.0188	10.5	247.0525	18
247.0538	11.5	247.0863	19.5
247.0838	10.25	247.1175	21
247.12	15	247.1563	19.25
247.1513	12.25	247.1888	21.25
247.185	12	247.2213	18.75
247.2163	10.75	247.2575	25.75
247.2538	12.5	247.29	22
247.29	9.75	247.32	24
247.3213	10.75	247.35	23.75
247.3513	10.5	247.3875	24.75
247.3875	11.25	247.4238	20.25
247.4238	9.5	247.4538	23.75
247.4525	7.25	247.4888	24.5
247.4888	13.75	247.5213	24.25
247.5213	9.5	247.5525	26
247.555	11	247.5863	20.75
247.585	11	247.6175	25.75
247.6213	16	247.6563	23.5
247.6525	17.25	247.6888	25.5
247.6863	9.75	247.7213	23
247.7175	13.25	247.7575	21
247.7538	13.25	247.79	28.25
247.7925	12.75	247.82	23.25
247.8238	13.5	247.85	21
247.8563	11.25	247.8875	22.75
247.8875	12.25	247.9238	30.75
247.9225	10.75	247.9575	22.5
247.9525	11.75	247.9888	23.5
247.9888	13.75	248.0225	25
248.0238	12	248.0525	23.25
248.055	12	248.0863	23.25
248.085	13.5	248.1188	22
248.1213	12.75	248.1563	22.25
248.1525	11	248.1888	24.5
248.1863	13.5	248.2213	21.25
248.2175	10	248.2575	18
248.2538	12	248.29	21.75
248.2925	12.75	248.32	23
248.3238	11.5	248.35	18

248.355	11.25	248.3875	16.75
248.3875	10.25	248.4238	15.75
248.4225	12.25	248.4538	16.5
248.4525	10.75	248.4888	16.5
248.4888	10.25	248.5225	19.75
248.5238	10	248.5525	19.25
248.555	9	248.5875	20.75
248.585	9.5	248.62	22.25
248.6213	11.25	248.6563	18.5
248.6525	8.5	248.6888	16.25
248.6863	12.5	248.7213	16.75
248.7175	10	248.7575	17.25
248.7538	10.75	248.79	20.25
248.7925	12	248.82	14.75
248.8238	10.25	248.85	15.5
248.855	10.25	248.8875	19
248.8875	12.25	248.9238	19
248.9225	12.75	248.9538	20.25
248.9525	10.5	248.99	20.5
248.9888	10.25	249.0225	18.75
249.0263	12.5	249.0525	17.75
249.055	11.25	249.0863	22
249.0875	8.5	249.1225	17.75
249.1213	10	249.1563	20.25
249.1525	11.5	249.19	20.75
249.1863	10.75	249.2213	21.25
249.2175	13	249.2575	21.75
249.2538	11.75	249.29	23.25
249.2925	13.25	249.32	23.25
249.3238	15.5	249.35	24.5
249.355	12.5	249.3875	19.5
249.3875	13.75	249.4238	21.75
249.4225	12.5	249.4538	24.5
249.4525	15.25	249.49	17.25
249.4888	11.25	249.5225	21.75
249.5238	13	249.5525	26.25
249.555	12.75	249.5863	25
249.5875	12.5	249.6225	23.75
249.6213	10.25	249.655	28.25
249.6525	10.75	249.6888	23.5
249.6863	9.25	249.7213	20.5
249.72	9.5	249.76	25.75
249.755	12.5	249.79	27.5
249.7938	10	249.82	22.75
249.825	10.5	249.85	25.25
249.8575	9.5	249.8875	24.25
249.89	10.25	249.9238	27.5
249.9238	11.5	249.9538	28.5
249.9538	13	249.99	27.5
249.9913	14.5	250.0225	24

250.0263	14	250.0525	31.25
250.0575	10.5	250.0863	25.5
250.09	10.75	250.1225	30.25
250.1225	12.25	250.155	23.5
250.1563	12.5	250.1888	22.25
250.1888	11.5	250.2213	25.75
250.22	11.25	250.26	26.25
250.255	9.25	250.29	24.25
250.2938	9.75	250.32	23.25
250.3263	8.75	250.3525	20
250.3575	11.5	250.3875	23.25
250.39	12	250.4238	23.5
250.4238	11.75	250.4538	21
250.4538	14.25	250.49	23.25
250.4888	7	250.5225	22.25
250.5275	12.5	250.5525	18.25
250.5575	12.75	250.5863	21.5
250.59	9.25	250.6225	21.5
250.6225	5.75	250.655	24.75
250.6563	10.5	250.6888	24
250.6888	11.5	250.7238	25.5
250.7213	11.75	250.76	27
250.755	12.75	250.79	23
250.7938	12	250.82	21.75
250.8263	12.25	250.8525	26.75
250.8575	11.5	250.8875	18.75
250.89	13	250.9213	23
250.9238	12.25	250.9538	22.5
250.9538	11.75	250.99	31.25
250.9888	14.5	251.025	24.25
251.025	12.75	251.0525	29.25
251.0575	13.25	251.0863	22.75
251.09	15	251.1225	22.75
251.1225	12.5	251.155	26.25
251.1563	14.25	251.1888	17.5
251.1888	16.5	251.2238	22
251.2213	11.5	251.26	16.5
251.255	11.25	251.29	19.75
251.2938	13.5	251.32	10.75
251.3263	13.5	251.3525	17
251.3575	10.5	251.3875	18.5
251.39	11.5	251.4213	18.75
251.4238	11.25	251.4538	15.75
251.4538	10.25	251.4913	17.5
251.4888	12.25	251.5225	14
251.525	11.25	251.5525	15.25
251.56	8.25	251.5863	17
251.59	8.5	251.6225	19
251.6238	10	251.655	19.75
251.6563	9	251.6888	20

251.6888	10
251.7213	12.75
251.7575	12.25
251.7938	12
251.8263	12.5
251.8575	11
251.89	13.5
251.9238	14.25
251.9538	12.75
251.9888	11
252.025	12
252.06	13
252.0925	9.5
252.125	11.75
252.1588	11.25
252.1913	12.25
252.2238	9.25
252.26	9.25
252.2975	14.25
252.3288	11.75
252.36	12.25
252.3913	11.75
252.425	12
252.455	11.5
252.49	10
252.5288	11
252.5613	12
252.5913	16
252.625	17.75
252.6613	14.75
252.6913	16.25
252.7238	13.75
252.76	13
252.7975	12.75
252.8288	16
252.86	14.5
252.8913	9.75
252.925	14.75
252.955	15.75
252.99	14.75
253.0288	14.75
253.0613	15.25
253.0913	13
253.125	14.5
253.1613	13.25
253.1913	13.75
253.2238	14
253.2625	13.25
253.2975	18.5
253.3288	14.25

251.7238	24.25
251.76	21.25
251.79	18
251.82	21.5
251.8525	20.5
251.8875	18.5
251.9188	21.75
251.9538	18.75
251.9913	24
252.0225	24
252.0525	23.25
252.0875	26.75
252.1225	24
252.155	26.25
252.1888	23.75
252.2238	25.5
252.26	23.75
252.29	26.25
252.32	22.25
252.3525	20
252.3875	23
252.4188	26
252.4538	25.75
252.4913	21.75
252.5225	18.75
252.5525	23.75
252.5875	16.75
252.6225	22
252.655	22.25
252.6888	18.75
252.7238	17.75
252.76	22.75
252.7913	21.75
252.82	17.75
252.8525	22.75
252.8875	21.25
252.9188	21.75
252.955	20
252.9913	18.25
253.0225	21.5
253.0525	19.75
253.0875	14.5
253.1225	21
253.155	19.25
253.1875	17
253.2263	22
253.26	17.5
253.29	23.5
253.32	25
253.3525	20.75

253.36	13.75	253.3875	25.75
253.3913	13.5	253.4188	25
253.425	14	253.4575	27.25
253.455	10.75	253.4913	24.5
253.49	12.75	253.5225	28
253.5288	13.75	253.5525	29
253.5613	10.75	253.5875	24.5
253.5913	13.75	253.6225	23
253.625	10	253.655	25.25
253.6613	17.25	253.69	26
253.6913	15.25	253.7263	22.5
253.7238	15.75	253.76	25.75
253.7625	15	253.79	28
253.7975	10.75	253.8213	26.5
253.8288	13.25	253.855	25
253.86	11	253.8875	23
253.8938	13.75	253.9188	28.5
253.925	10.5	253.9575	28
253.955	12.25	253.9913	28.5
253.99	10.5	254.025	24.75
254.0288	10.25	254.0525	25.25
254.0613	12.75	254.0875	27.75
254.0913	10.25	254.1225	31.75
254.125	13.5	254.155	26
254.1613	11.5	254.1875	27.5
254.1913	13	254.2263	32.5
254.2238	12.25	254.26	24
254.265	12.5	254.29	30
254.2975	11.25	254.325	26.25
254.3288	10.75	254.3575	28
254.36	10	254.39	24.75
254.3913	9.25	254.4213	30.75
254.425	10	254.4625	30.25
254.455	10.75	254.4938	28.75
254.49	10.75	254.525	27.75
254.5288	12.75	254.555	26.25
254.5613	11.5	254.5888	25.75
254.5913	11.75	254.6238	29.5
254.625	12	254.6563	26.75
254.6613	9.75	254.6888	21.5
254.6913	9	254.7275	25
254.7238	11	254.7613	24.25
254.765	7.25	254.7913	25.75
254.7975	10	254.825	25.25
254.8288	9	254.8575	23.5
254.86	8.75	254.89	26.5
254.8913	10	254.9213	26.5
254.925	9.25	254.9625	24.5
254.955	9.75	254.9938	26.5
254.99	12.5	255.025	24

255.0288	11.75
255.0613	11.5
255.0913	14.25
255.125	10
255.1613	13
255.1913	10.5
255.2238	11
255.2625	9.5
255.2975	10.75
255.3288	9
255.36	10.75
255.3913	10.25
255.425	9.75
255.455	8.5
255.4925	8.25
255.5288	10.5
255.5613	13.25
255.5913	11.5
255.625	9
255.6613	9.5
255.6913	11.5
255.7238	10.5
255.7625	10.75
255.7975	12.25
255.8313	11.75
255.86	11.5
255.8938	10
255.925	9
255.955	9.75
255.995	12.25
256.0288	11.75
256.0613	11.5
256.0913	11.5
256.125	10.5
256.1613	14.25
256.1913	13.5
256.2238	11.25
256.2625	11.25
256.2963	11.25
256.3288	10.75
256.3625	11.5
256.3913	13.5
256.425	12.75
256.455	10
256.495	10.75
256.5288	15.5
256.5613	10.5
256.5913	12
256.625	11.5
256.6613	12.5

255.055	21.75
255.0888	26
255.1238	26
255.1563	23.5
255.19	24.5
255.2275	27.75
255.2613	25.5
255.2913	27.75
255.325	28
255.3575	28.5
255.39	27.75
255.4213	30.5
255.46	21.25
255.4938	24.25
255.525	24.25
255.555	24.5
255.5888	27.75
255.6238	25.25
255.6563	25.5
255.69	28
255.7275	23
255.7613	22
255.7913	26.75
255.825	26.5
255.8575	31.5
255.89	21
255.9213	26.5
255.96	26
255.9938	29.25
256.025	28
256.055	28.75
256.0888	29.25
256.1238	28
256.1563	29.5
256.19	25.5
256.2275	33.25
256.2613	28.75
256.2938	28
256.325	28.75
256.3575	23.5
256.39	25.5
256.4213	29
256.46	24.25
256.4938	27.25
256.525	18.25
256.555	24.75
256.59	22.75
256.6238	20.25
256.6563	22.5
256.69	20.5

256.6913	13.25	256.7275	19
256.725	11.5	256.7613	21.75
256.7625	8.5	256.7913	25.75
256.7963	13.25	256.825	21.25
256.8288	12.25	256.86	28.25
256.86	9.25	256.89	25.5
256.8913	10.25	256.9213	23.25
256.925	9	256.96	26
256.955	12	256.9938	25.75
256.995	8.25	257.025	27.75
257.03	8.75	257.0575	27.25
257.0613	8	257.09	24.75
257.0913	10.75	257.1238	26.25
257.125	9.25	257.1563	26
257.1613	7.5	257.19	29.5
257.1913	9.75	257.2263	22.75
257.225	9.75	257.2613	24
257.2625	12	257.2938	24.5
257.2963	13	257.325	25.25
257.3288	8	257.36	25
257.36	11	257.39	29.5
257.3913	9.5	257.4225	29.5
257.4275	13.25	257.46	29.5
257.455	9.75	257.4938	26
257.495	9.25	257.525	26
257.5288	12.5	257.5575	25.75
257.5613	12.25	257.59	21
257.5913	10.5	257.6238	24.25
257.6275	11	257.6563	23.75
257.6613	14.25	257.69	25.25
257.6913	12.25	257.7263	24
257.725	16.75	257.7613	27.25
257.7625	13	257.7913	25
257.7963	14.5	257.825	28.75
257.8288	10.5	257.86	25.75
257.86	14.75	257.89	26
257.8913	14.25	257.9225	28.25
257.925	11.5	257.96	29
257.955	11.25	257.9938	25.75
257.995	12.25	258.025	23.25
258.0288	12.25	258.0575	22.5
258.0613	13.25	258.0925	29.25
258.0913	12.5	258.1238	25
258.125	11.25	258.1575	23.75
258.1613	11	258.1913	25.5
258.1913	11.75	258.2263	28.5
258.225	8	258.2613	25.25
258.2625	12	258.2913	22.5
258.2963	11.5	258.3275	20.25
258.3288	12	258.36	25

258.36	10.75	258.39	22.75
258.3913	9	258.4225	24
258.425	12	258.46	26.25
258.4575	7.5	258.4938	21.75
258.495	11.25	258.525	24.25
258.5288	11.5	258.5575	20.25
258.5613	9.5	258.59	24
258.5913	9.25	258.6238	20
258.625	13.75	258.6563	24.75
258.6613	13.75	258.6913	23.75
258.6913	9.25	258.7263	26.25
258.7275	9	258.7613	22
258.7625	10.75	258.7913	21
258.7963	9.5	258.8275	26.5
258.8288	12	258.86	23.25
258.86	11.75	258.89	24.75
258.8913	9	258.9225	23
258.925	9.75	258.96	26
258.9575	8	258.9938	28.25
258.995	11.75	259.025	27.25
259.0288	11.75	259.0575	28
259.0613	10.25	259.09	26
259.0913	10.75	259.1238	25.75
259.125	11.75	259.1563	27.25
259.1613	12.75	259.1913	28.5
259.1913	10.75	259.2263	23.75
259.2275	10.25	259.2613	22
259.2625	15.5	259.2913	23.5
259.2963	11.25	259.3275	25.75
259.3288	13.5	259.36	24.25
259.36	13.5	259.39	22.5
259.3913	12.75	259.4225	24
259.425	10.25	259.46	26.5
259.46	12.5	259.4913	21.25
259.495	12.5	259.525	22.5
259.5288	12.5	259.5575	25.25
259.5613	9	259.59	26
259.5913	11.5	259.6238	22.5
259.625	9	259.6563	25.5
259.6613	11.25	259.6913	25.75
259.6925	8.75	259.7263	24.5
259.7275	11	259.7613	17
259.7625	10	259.7913	22.25
259.7963	8.75	259.8275	27.75
259.8288	11.5	259.86	23.75
259.86	10	259.89	22
259.8938	9.5	259.9225	25.25
259.9288	15.75	259.96	18.5
259.9613	10.75	259.9913	20.75
259.9975	11.5	260.025	20.5

260.0313	9.25	260.0575	20
260.0638	12.25	260.09	21.5
260.0938	10.5	260.1238	23
260.1263	9.75	260.1588	23
260.1625	13.75	260.1913	15.75
260.195	11.5	260.2263	17.5
260.23	9	260.2613	20
260.2638	11.5	260.2913	15.5
260.2975	9.25	260.3275	19.25
260.33	11.25	260.36	16.75
260.3625	11.5	260.39	18.5
260.3938	12	260.4225	18.75
260.4288	7.25	260.46	17.75
260.4613	8.5	260.4913	18.75
260.4975	11.75	260.525	22
260.5313	10.75	260.5588	22.25
260.5638	11.5	260.59	20.25
260.5938	11	260.6238	15.25
260.6263	12.25	260.6588	17.25
260.6625	12.75	260.6913	20
260.695	13	260.7263	20.25
260.73	10	260.7613	19
260.7638	10.75	260.7913	17
260.7975	12	260.8275	15
260.8325	11.5	260.86	22
260.8625	10	260.89	20.5
260.8938	13.25	260.9225	16.25
260.9288	10	260.96	19.25
260.9638	14.25	260.9913	15.75
260.9975	16.75	261.025	18.75
261.0313	13.5	261.0588	18.75
261.0638	13.5	261.09	19.75
261.0938	14	261.1238	21.5
261.1263	15.25	261.1588	21
261.1625	9.75	261.1913	23.75
261.195	11.75	261.2263	24.25
261.2325	11.5	261.2588	19.5
261.2638	12.5	261.2913	20.25
261.2975	10.75	261.3275	21.5
261.33	10.75	261.36	21.5
261.3625	12.75	261.39	18.75
261.3938	10	261.4225	25.25
261.4288	11.75	261.4588	12
261.4638	13.25	261.4913	20.5
261.4975	15.25	261.525	17.5
261.5313	12.5	261.5625	21.5
261.5613	13.25	261.59	14.25
261.5938	13.75	261.6238	24
261.6263	10.75	261.6588	20.5
261.665	12	261.6913	21.5

261.695	10.75	261.7263	16.5
261.7325	10.75	261.7588	23.25
261.7638	12.25	261.7913	18.25
261.7975	10.75	261.8275	18.25
261.83	9.75	261.86	17.25
261.8625	12	261.89	20.25
261.8938	12	261.9225	21.75
261.9288	9.25	261.9588	18.5
261.9638	11.25	261.9913	24.25
261.9975	11.5	262.025	20.5
262.0313	8	262.0588	20.5
262.0613	10.5	262.09	19.25
262.0938	12.25	262.1238	19.25
262.1263	9.5	262.16	20.75
262.165	10.75	262.1913	23.75
262.195	9.75	262.2263	20.75
262.2338	11.25	262.2588	17.75
262.2638	12.75	262.2913	19
262.2975	12	262.3275	22.75
262.33	10.5	262.3613	16.5
262.3625	9.25	262.39	18.25
262.3938	10.5	262.425	19
262.4288	10.5	262.4588	19.5
262.4638	11.75	262.4913	17.25
262.5	12.25	262.5275	19.75
262.5313	10.5	262.5588	19.75
262.5613	10.5	262.59	22.75
262.5938	8.25	262.6238	22
262.6263	10.75	262.6588	20.75
262.665	9	262.6913	20.75
262.6963	10.25	262.7263	21.25
262.7325	9	262.7588	22.5
262.7688	10.25	262.7938	21.75
262.8	12.25	262.8275	18.75
262.8325	11.75	262.86	19.25
262.865	9.25	262.8913	20.5
262.8963	10	262.925	19
262.93	12.5	262.9588	15.25
262.9675	10.5	262.9913	22.25
263	9.75	263.0238	26.25
263.0325	12.25	263.0588	20.5
263.0625	11.75	263.09	21.25
263.095	11	263.1238	24.75
263.1288	9.5	263.1588	24.75
263.1675	12.25	263.1913	21.5
263.1988	10	263.2263	27.5
263.2338	11.5	263.2588	22
263.2688	12.25	263.2938	19.75
263.3	14.5	263.3275	20
263.3325	12	263.3625	18.75

263.365	10.75	263.3913	22.5
263.3963	11.25	263.425	26.5
263.43	8.75	263.4588	23.25
263.4675	8	263.4913	26.75
263.5	9.25	263.5238	25.5
263.5325	13	263.5588	21
263.5625	8	263.59	24
263.595	13.75	263.6238	24.75
263.6288	12.25	263.6588	24.25
263.6675	12	263.6913	22.5
263.7	11.75	263.7263	25
263.7338	14.5	263.7588	25.75
263.7688	14.25	263.795	24.5
263.8	9.25	263.8275	19.5
263.8325	11.75	263.86	20.75
263.865	13	263.8913	23.5
263.8963	13	263.925	25.5
263.93	13.75	263.9563	23
263.97	10	263.9913	21
264	15.25	264.0238	23.25
264.0325	13.75	264.0588	24.75
264.0625	12.75	264.09	24.25
264.095	11.75	264.1238	21.75
264.1288	11.25	264.1588	26.25
264.1675	13	264.1913	24.25
264.2	14	264.2263	26
264.2363	11.25	264.2588	25.5
264.2688	13.25	264.2938	27.75
264.3	12.75	264.3275	27.5
264.3325	12.75	264.36	18.75
264.365	11.25	264.3913	23.5
264.3963	11.5	264.425	21
264.43	12.25	264.4563	22.75
264.4675	13.75	264.4913	22.25
264.5	10.25	264.5238	20.75
264.5325	12.5	264.5588	21.25
264.5625	10.25	264.59	25.5
264.595	10.5	264.6238	26.75
264.6313	11.25	264.6588	29.25
264.6675	10.25	264.6913	29.75
264.7	15.75	264.7263	21.75
264.7338	11.5	264.7588	23.25
264.7688	12.75	264.7913	26.25
264.8	12	264.8275	22.25
264.8325	10.75	264.86	24.5
264.865	15	264.8913	26.75
264.8963	14.25	264.925	28.25
264.9325	13.5	264.9563	28
264.9675	14.75	264.9913	26.75
265	10.75	265.0238	25.25

265.0325	20.5	265.0588	23.5
265.0625	20.5	265.09	24.75
265.095	12.25	265.125	25.25
265.1313	12.75	265.1588	21.75
265.1675	15.5	265.1913	22
265.2	17.25	265.2263	24.25
265.2338	15.5	265.2588	22.25
265.2688	11.5	265.2913	21.75
265.3	12.75	265.3275	28.75
265.3325	16	265.36	24
265.365	14	265.3913	28.25
265.3963	14.75	265.425	19.5
265.4325	12.5	265.4563	23.25
265.4675	10.5	265.4913	22.25
265.5	9	265.525	19.25
265.5325	9.25	265.5588	22.75
265.5625	11.75	265.59	22
265.595	8.25	265.6263	25
265.6313	10	265.6588	23.25
265.6675	11	265.6913	23.5
265.7	11.5	265.7238	24
265.7338	34.25	265.7588	29.25
265.7688	30.25	265.7913	26.5
265.8	15.75	265.8275	24
265.83	28.5	265.86	26.5
265.865	15.25	265.8913	22.5
265.8963	13	265.925	26
265.9325	20.75	265.9563	24
265.9675	14	265.9913	25.5
266	13.25	266.025	22.25
266.0325	11.25	266.0588	22.5
266.0625	18.75	266.09	26.25
266.095	15.5	266.1263	26
266.1313	15	266.1588	27.5
266.1675	12	266.1913	25.75
266.2025	16	266.2238	27.25
266.2338	14.25	266.2588	25.5
266.2688	14.5	266.2938	25.25
266.3	13.75	266.3275	31
266.33	16.25	266.36	25
266.3663	10.5	266.3913	22.25
266.3963	12	266.425	23.75
266.4325	10.75	266.4563	27.5
266.4675	10.75	266.4913	23.75
266.5	10.5	266.525	21.75
266.5325	11.25	266.5588	17.75
266.5625	12	266.59	19.75
266.595	10.25	266.6263	20.5
266.6313	14.5	266.6588	21.75
266.6675	15.25	266.6913	22

266.7	10.75	266.7238	18.5
266.7338	12	266.7588	23.5
266.7688	11.25	266.7913	18.75
266.8	12.5	266.8275	22
266.83	9	266.86	20.75
266.8663	12	266.8913	23.75
266.8963	10.75	266.925	19.5
266.9325	13.75	266.9563	22
266.9675	12	266.9913	19.5
267	10.75	267.025	19.25
267.0325	10.75	267.0575	18
267.0625	11.75	267.09	16.25
267.095	11.5	267.1263	18.75
267.1313	10.75	267.1588	18.25
267.1688	14.75	267.1913	24.5
267.2	15.75	267.2238	19.5
267.2338	13.5	267.2588	18.75
267.2688	11.25	267.2913	17
267.3	13.75	267.3275	18.5
267.33	9	267.36	21.75
267.3663	11.75	267.3913	16.25
267.3963	13.75	267.425	25.5
267.435	11	267.4563	23.25
267.4675	11.25	267.4913	20
267.5	10.5	267.525	25.75
267.5325	13.25	267.5575	24.25
267.5625	10.75	267.59	18
267.5975	12.75	267.6263	24.75
267.6313	14.25	267.6588	23.5
267.6688	11.5	267.6913	25.25
267.7	9.75	267.7238	20.75
267.7363	14.25	267.7588	21.75
267.7713	13	267.7913	25
267.8025	12.25	267.8275	24.25
267.835	12.75	267.86	20
267.8688	9.25	267.8913	19
267.9	13.25	267.925	22.25
267.9375	12.75	267.9563	17
267.97	12	267.9913	18.25
268.0013	10.5	268.025	17.75
268.0338	9.75	268.0575	20.25
268.0638	13.25	268.09	16.75
268.0988	11.75	268.1263	19.25
268.1325	11.75	268.1588	21.75
268.17	9.75	268.1913	19.5
268.2013	15	268.2238	22
268.2363	13	268.2613	18.75
268.2713	11	268.2925	21
268.3038	10.75	268.3275	18.25
268.335	10.75	268.36	21.75

268.3688	11	268.3913	25
268.4	9.75	268.425	20.75
268.44	10	268.4563	21.5
268.47	9	268.4913	21.25
268.5013	10.25	268.525	20.25
268.5338	11.25	268.5575	25.75
268.5638	11.75	268.5925	21.75
268.5988	12	268.6263	22
268.6338	12	268.6588	19.25
268.67	9.75	268.6913	17.75
268.7013	10	268.7225	16
268.7363	10.75	268.7613	22.25
268.7713	10.75	268.7925	17.5
268.8025	11	268.8275	19.75
268.835	12	268.86	19.25
268.8688	11.25	268.8913	18.5
268.9025	14	268.925	17.75
268.9375	10.5	268.9563	20.75
268.9713	12	268.9913	21
269.0013	11.5	269.025	17.75
269.0338	8.25	269.0575	18.25
269.0638	11.75	269.09	18
269.0988	11.5	269.1263	16.25
269.1325	10	269.1588	18.25
269.17	9.5	269.1913	20
269.2013	11.5	269.2225	17.25
269.2363	10.75	269.2613	20.25
269.2713	9	269.2925	20
269.3025	11	269.3275	18.75
269.335	10.75	269.3613	20
269.3688	10.5	269.3913	18.75
269.4	10.25	269.425	19.75
269.4375	8.25	269.4563	20.5
269.47	9	269.4913	17.75
269.5013	10.75	269.525	21
269.5338	10.25	269.5575	22.5
269.5663	10.5	269.59	16
269.5988	8.75	269.6263	22.5
269.6325	9.5	269.6588	18
269.67	9.75	269.6913	18.75
269.7013	9.5	269.7225	21.25
269.7363	10.75	269.7613	20.25
269.7713	10	269.7925	19.5
269.8025	8	269.8275	17
269.835	8.25	269.8613	19.75
269.8688	8.5	269.8913	16.75
269.9	12.25	269.925	19
269.9375	11	269.9563	20.5
269.97	11.5	269.9913	19
270.0013	13.5	270.025	22.25

270.0338	7.5	270.0575	22.25
270.0663	11.25	270.09	20.75
270.0988	12.75	270.1263	17
270.1325	9.25	270.1588	21
270.17	12	270.1913	18.25
270.2013	10.5	270.225	18.5
270.2363	12	270.2613	21.5
270.2713	8.25	270.2925	16.75
270.3025	9	270.325	19.5
270.335	10	270.3613	21.5
270.3688	12	270.3913	20.5
270.4	11	270.425	22.5
270.4375	13.5	270.4563	24.5
270.47	10.75	270.4913	21
270.5013	11.5	270.525	24.5
270.5338	11	270.5575	25.25
270.5688	12.25	270.59	21
270.6013	10.5	270.6263	21.75
270.6363	12	270.66	23.75
270.6713	11.25	270.6913	25.75
270.705	12	270.7263	19.75
270.7388	8.25	270.7613	22.25
270.7738	9.25	270.7925	19.75
270.8038	10	270.825	23.5
270.8363	10	270.8613	18.25
270.8713	7	270.8913	21.75
270.9025	11.25	270.925	20.75
270.94	10	270.9575	18.5
270.9713	12.25	270.9925	26.25
271.0038	8.5	271.0275	21.25
271.0363	10.25	271.0575	23.75
271.0688	11.25	271.09	22
271.1013	11.5	271.1263	18.75
271.1363	14.25	271.1588	22.75
271.1713	11.5	271.1913	18
271.205	15	271.225	18.5
271.2388	12.25	271.2613	26
271.2725	14.75	271.2925	20.5
271.3038	16	271.325	17.75
271.3363	11.5	271.3613	24.5
271.3713	13	271.3913	21.25
271.405	9.5	271.425	19.75
271.44	12.25	271.4563	19
271.4713	10.25	271.49	17.75
271.5038	11.5	271.525	20.75
271.5363	11.5	271.5575	26.75
271.5688	9.5	271.59	22
271.6013	12.5	271.6313	18.75
271.6363	13.25	271.6588	20.25
271.6725	12.75	271.6913	22.5

271.705	11	271.725	21.25
271.7388	9.75	271.7613	19.5
271.7725	9.5	271.7925	19.25
271.8038	7.5	271.825	21.5
271.8363	10.75	271.8613	18.75
271.8713	10	271.8913	16
271.905	10.75	271.9225	20.5
271.94	11	271.9563	17.5
271.9713	11.25	271.99	16.25
272.0038	11.75	272.025	18
272.0363	12.5	272.0575	21.25
272.0688	10.25	272.09	22.75
272.1	9.25	272.1263	20.75
272.1363	11.25	272.1588	21
272.17	13.5	272.1913	22.5
272.2075	13.75	272.225	19.25
272.2388	11.75	272.2613	19.75
272.2725	11.75	272.2925	16.75
272.305	13.5	272.325	17.75
272.3363	13.25	272.3613	20.75
272.3713	10.75	272.3913	20.75
272.405	10.5	272.4225	16.75
272.44	11.75	272.4563	21
272.4713	10.25	272.49	20.25
272.5038	13.75	272.5275	23.75
272.5363	13	272.5575	22.5
272.5688	13.5	272.59	23.25
272.6	10.5	272.6263	18.5
272.6363	9.25	272.6588	20
272.67	11.25	272.6913	21.5
272.705	14	272.725	23.25
272.7388	11	272.7613	17.75
272.7725	9	272.7925	21.5
272.805	10.25	272.8288	20.5
272.8363	12.75	272.8613	21.25
272.8713	11.75	272.8938	26.5
272.905	8.75	272.925	22.5
272.94	8.75	272.9588	23
272.9713	10.75	272.995	17.5
273.0038	10.5	273.03	22
273.0363	10.75	273.06	18.5
273.0688	10.25	273.095	20.75
273.1	10.25	273.1275	24.75
273.1363	11	273.16	19.5
273.17	9	273.1925	19.75
273.205	8.5	273.2288	19.75
273.2388	7.5	273.2625	22.25
273.27	7.25	273.2938	20.5
273.305	11.25	273.3263	23.75
273.3363	12.5	273.3638	20.5

273.3725	6.5	273.3963	23
273.405	9	273.425	20.5
273.44	11.5	273.4588	21.25
273.4713	7.5	273.4925	19
273.5038	9	273.53	20.5
273.5363	10.25	273.56	21.75
273.5688	11.25	273.5938	23.25
273.6	9.5	273.6275	23.75
273.6363	9	273.66	21.75
273.67	12	273.6925	22
273.705	10.25	273.7288	23.5
273.7388	9.75	273.7625	22.25
273.77	9	273.7938	21.25
273.805	8.25	273.8263	22
273.8363	8	273.8638	21.75
273.8725	7.75	273.8938	23.25
273.905	9	273.925	22.5
273.94	9.25	273.9588	20.75
273.9713	5.75	273.9925	21.5
274.0038	6.5	274.03	24.75
274.0363	5	274.06	17.75
274.0688	8.5	274.0938	19.75
274.1	9.25	274.1275	18.75
274.1363	6.5	274.16	19.75
274.17	7.75	274.1925	16.75
274.205	6.75	274.2288	20.25
274.2388	9.5	274.2625	16.5
274.27	6	274.2938	18.75
274.305	10.25	274.3263	21.75
274.3363	9	274.3613	20.5
274.3725	12.25	274.3938	18.5
274.405	10.75	274.425	21.5
274.44	17.75	274.4588	19.75
274.4713	12.75	274.4925	19.5
274.5038	12	274.53	19.5
274.5363	15.5	274.56	18.75
274.5688	13.25	274.5938	19
274.6013	15.25	274.6338	21
274.6363	15.25	274.66	24
274.67	11	274.6925	21
274.705	14	274.7288	24
274.7388	11.25	274.7613	24.5
274.77	12.5	274.7938	23.5
274.805	11.25	274.8263	27.5
274.8363	10.75	274.8613	23
274.8725	12.75	274.8938	25.25
274.905	20.5	274.925	25.5
274.94	8.5	274.9613	26
274.9713	17	274.9925	23.75
275.0038	13.75	275.03	22.5

275.0363	10	275.06	20.75
275.0688	11.5	275.0938	20.75
275.1	9	275.1275	20.5
275.1363	13.5	275.16	21.75
275.17	11.5	275.1925	24.75
275.205	12.5	275.2288	21.5
275.2388	10.25	275.2625	22.5
275.27	10.5	275.2938	16.75
275.305	14	275.3263	28.5
275.3363	13	275.3613	26.75
275.3725	10.75	275.3938	22.75
275.405	14.5	275.425	26.5
275.44	10	275.4613	19.5
275.4713	12.25	275.4925	22
275.5038	14	275.53	24.25
275.5363	10.5	275.56	20.5
275.5688	10.25	275.5963	25.5
275.6	13.25	275.63	24
275.6363	11	275.6625	22.75
275.67	9.5	275.6938	20.25
275.705	10	275.73	20
275.7388	12	275.765	18.5
275.77	13.75	275.7963	22.25
275.805	9	275.8275	20.25
275.8363	8.5	275.8625	19.75
275.8725	9.25	275.895	14.75
275.9075	8.75	275.9275	21
275.94	8.5	275.9638	24.5
275.9713	11.75	275.9963	17.75
276.0038	13.5	276.0288	26.25
276.035	12.25	276.0625	25
276.0688	14.5	276.0963	21.5
276.1013	14.5	276.13	22.5
276.1363	15.5	276.1625	25.5
276.17	12.75	276.1938	26.5
276.205	13	276.23	22.25
276.2388	15.25	276.2663	21.5
276.27	18.75	276.2963	26
276.305	14.25	276.3275	26.25
276.3363	12	276.3625	23.25
276.3725	14	276.395	24
276.405	12.75	276.4275	21.25
276.44	12.25	276.4638	20.5
276.4713	11.75	276.4963	20
276.5038	13.75	276.5288	25.75
276.535	11.75	276.5625	25
276.5688	12	276.5963	22
276.6013	12.5	276.63	19
276.6375	13.25	276.6625	19.5
276.67	13.25	276.6938	25.25

276.7075	10.5	276.73	25.75
276.7388	14.25	276.765	22.75
276.77	8.25	276.7963	17.5
276.805	9	276.8325	22.25
276.8363	12.75	276.86	21.75
276.8725	12.5	276.895	19.25
276.905	13.25	276.9275	21.75
276.94	11.75	276.965	21.25
276.9713	13.75	276.9963	20.75
277.0038	13.25	277.0288	23.75
277.0375	17.25	277.0625	22.75
277.0688	14.5	277.0963	22
277.1038	14.25	277.13	21
277.1375	15.75	277.1625	24.75
277.17	14.25	277.1938	23
277.205	11.75	277.23	20.25
277.2388	10	277.265	19.5
277.27	9	277.295	25
277.305	12.5	277.3288	22.75
277.3363	10.5	277.36	23.25
277.3725	10.75	277.395	22.75
277.405	9	277.4275	20
277.44	11.25	277.465	24.5
277.4713	13	277.4963	19.75
277.5038	14.5	277.5288	22
277.5375	13.5	277.5625	20.75
277.5688	13	277.5963	18.25
277.6038	14.25	277.63	18
277.6388	13.25	277.6625	16.25
277.67	15.5	277.6938	17.5
277.705	16.25	277.73	20.75
277.7388	13.5	277.765	18.75
277.77	14.75	277.795	16.75
277.805	12.25	277.8288	21.5
277.8363	14.5	277.86	21
277.8725	12.5	277.895	20
277.905	12.5	277.9275	18.25
277.94	12.75	277.965	21
277.9713	15.75	277.9963	16
278.0038	11.5	278.0313	23.75
278.0375	9	278.0625	20.25
278.0688	9.5	278.0963	20.5
278.1038	13	278.13	22.25
278.1375	10.75	278.1625	17
278.17	11	278.1938	20.25
278.205	9.5	278.23	21.25
278.2388	10	278.265	21
278.27	8.75	278.295	19
278.305	14.75	278.3288	21.75
278.3363	12	278.36	21

278.3725	12.25	278.395	19.75
278.405	13.25	278.4275	22.25
278.4413	15	278.465	22.25
278.4713	9	278.4963	23.25
278.5038	11	278.5288	20.5
278.5375	11	278.5625	23.5
278.5688	12.25	278.5963	18
278.6038	15.75	278.6288	18.75
278.6375	15.5	278.6625	14.5
278.67	14	278.6938	21.5
278.705	15.25	278.7325	15
278.7388	15	278.765	16.75
278.77	13.25	278.795	14.25
278.8025	12.5	278.8288	14.25
278.8363	12.75	278.86	16.75
278.8738	10.5	278.895	22.75
278.9075	14	278.93	15
278.9425	14.5	278.965	21.25
278.9738	8	278.9963	16.75
279.0063	12.5	279.0288	14
279.0388	10	279.0625	14.5
279.0713	12.75	279.0963	17.5
279.1063	10.25	279.1288	18.25
279.14	12	279.1625	18.25
279.1713	11	279.1938	16.25
279.2063	10.25	279.2325	15.75
279.24	8.75	279.265	17.5
279.2725	7.25	279.295	20
279.3063	9.75	279.3288	16.5
279.3375	9.5	279.36	16.75
279.3738	14.75	279.395	16.5
279.4075	14	279.43	15.75
279.4425	15.75	279.4625	16
279.4738	13.75	279.4963	18.75
279.5063	13.75	279.5288	18.5
279.5388	14.25	279.5625	18.75
279.5738	8.75	279.5963	20.25
279.6063	12.75	279.6275	17.75
279.64	13.75	279.6625	17.25
279.6713	15.75	279.6938	20.75
279.7063	14.5	279.7325	18.5
279.74	13.5	279.765	18.75
279.7725	10.75	279.795	15
279.8063	9.5	279.8325	23.25
279.8375	10	279.86	17
279.8738	13.75	279.895	18.25
279.9075	12.75	279.93	16.25
279.9425	11.75	279.965	20.5
279.9738	10	279.9963	14.25
280.0063	10.25	280.0288	18

280.0388	12.5	280.0625	20
280.0713	6.5	280.0963	20.5
280.1063	12	280.1275	18.25
280.14	8.75	280.1638	15.75
280.1713	9	280.1938	18.5
280.2063	7.75	280.2325	19.5
280.24	9	280.265	14.5
280.2738	8.75	280.295	17
280.3063	7	280.3275	19.25
280.3375	8.25	280.36	17.75
280.3738	7.75	280.395	18.75
280.4075	8.5	280.43	18.5
280.4425	12.25	280.4625	20.25
280.4738	13.5	280.4963	17.5
280.5063	14.5	280.5288	20.75
280.5388	9.75	280.5625	17
280.5725	11.5	280.5963	20
280.6063	10.5	280.6275	18.5
280.64	12.75	280.6638	21.5
280.6713	9.25	280.6963	19.75
280.7063	9.25	280.7325	16.25
280.74	7.5	280.765	21.25
280.7725	9.25	280.7975	17
280.805	10.25	280.8275	19.25
280.8375	9.25	280.86	14.5
280.8763	8.25	280.895	17
280.9075	6.5	280.93	17
280.9425	9	280.9625	16.75
280.9738	9.5	280.9963	20.25
281.0063	11.25	281.0288	15.25
281.0388	12.5	281.0625	17.25
281.0713	8.75	281.0963	16.25
281.1063	11	281.1275	20.5
281.14	11.75	281.1638	17.25
281.1713	7.75	281.1963	20.25
281.2063	11	281.2325	25.75
281.24	14.5	281.265	21
281.2725	12	281.2975	18.25
281.305	11	281.3275	23.5
281.3375	12.75	281.36	21.5
281.3763	9.5	281.395	25.5
281.4075	10.75	281.43	23.25
281.4425	7	281.4625	22.75
281.4738	9.25	281.4963	19.25
281.5063	14	281.5288	20.5
281.5388	11.25	281.565	23.5
281.5713	10	281.5938	23
281.6063	9.75	281.6275	23.25
281.64	9.75	281.6638	25.25
281.6713	11.75	281.6963	25.5

281.7063	10.75	281.7325	26
281.74	9.25	281.765	22.25
281.7738	13.5	281.7975	26.75
281.805	9.5	281.8275	25.25
281.8375	9.75	281.86	22.5
281.8763	9.5	281.8975	20.75
281.9075	9.75	281.93	21
281.9425	10.25	281.9625	24.5
281.9738	9.25	281.9963	21.75
282.0063	12	282.0288	19.5
282.0388	8.5	282.065	23.75
282.0713	8.5	282.0938	25.75
282.1063	9.25	282.1288	23.25
282.14	9.75	282.1638	20.25
282.1713	11.5	282.1963	20.75
282.2063	10.25	282.2313	26.25
282.24	11.75	282.265	24.5
282.2738	9.75	282.2975	19
282.305	11.5	282.3275	21.5
282.3375	12.75	282.36	23.25
282.3763	12.25	282.395	23.75
282.4075	12.75	282.43	22.25
282.445	8	282.4625	24
282.4738	7.75	282.4963	20
282.5063	13.5	282.53	25
282.5388	7.75	282.5625	21.25
282.5713	10	282.5938	22.5
282.6063	10	282.6275	22.5
282.64	7.75	282.6638	18.5
282.6713	11.5	282.6963	20
282.7063	10	282.7313	17
282.74	6.25	282.765	24.25
282.7738	11	282.7975	20
282.805	8.25	282.8275	23.75
282.8375	7.25	282.86	19.25
282.8763	11	282.8925	17.5
282.9075	9.75	282.93	19.5
282.9425	9.25	282.9625	24
282.9738	8	282.9963	20.75
283.0063	7.25	283.03	15.25
283.0388	10.25	283.0625	21.5
283.0688	8.5	283.0938	17.75
283.1063	8.25	283.1275	20.25
283.14	9	283.1638	19.75
283.1713	6.25	283.1963	21
283.2063	6.25	283.2313	19.75
283.24	4.75	283.265	18.75
283.2738	9.25	283.2975	17.25
283.305	8.75	283.3275	18.75
283.3375	6.75	283.3575	17

283.3763	8.5	283.3925	22.5
283.4075	11.75	283.43	22
283.4425	9.75	283.4625	17.75
283.4738	8	283.4963	18.75
283.5063	11.75	283.53	15.5
283.5388	11.75	283.5625	14
283.5688	9.5	283.5938	18.25
283.6038	6.75	283.6288	18.25
283.64	8.5	283.6638	17.5
283.6713	8.5	283.6963	16.25
283.7063	8.5	283.7313	15.25
283.74	12.75	283.7675	17.75
283.7738	15.5	283.7975	15.5
283.805	12.25	283.8275	15
283.8375	10.25	283.8575	18.75
283.8763	11	283.8925	17.5
283.9075	9	283.93	14.5
283.9425	12	283.9625	20
283.9738	11	283.9963	19.75
284.0063	12.25	284.03	15.25
284.0388	12.75	284.0625	13.75
284.0688	13	284.0938	16.75
284.105	9.25	284.1288	16.5
284.14	11	284.1638	19.75
284.17	12.5	284.1963	23
284.2063	11.75	284.2313	17.5
284.24	10.25	284.2675	15.75
284.2738	8.5	284.2975	21.25
284.305	11	284.3275	17.75
284.3375	10.75	284.3575	21
284.3763	8.25	284.395	18
284.4075	8	284.43	22.5
284.4425	10	284.4625	18.25
284.4738	9	284.4938	23.75
284.5088	7.5	284.53	19.75
284.5388	8.75	284.5625	25
284.5688	7.75	284.5938	25
284.605	8.5	284.6275	22.25
284.64	5.75	284.6638	25.5
284.67	6.5	284.6963	22.75
284.7063	5.25	284.7313	24.25
284.74	7.25	284.7675	24.25
284.7738	5.5	284.7975	21.75
284.805	5	284.8275	19.75
284.8375	6	284.86	20
284.8763	5.5	284.8925	22.25
284.9075	5.75	284.93	21.75
284.9425	5	284.9625	24.25
284.9738	5	284.995	18
285.0088	7	285.03	22

285.0388	8	285.0625	21.5
285.0688	9	285.0938	22
285.105	5.5	285.1275	18.5
285.1425	5	285.1638	18.75
285.17	8	285.1963	18.5
285.2063	6.5	285.2325	17.75
285.24	5.75	285.27	24.5
285.2738	8.75	285.3	18.5
285.305	6.75	285.33	16.5
285.3375	5.75	285.3625	19.25
285.3763	6.5	285.395	25.25
285.4075	6.5	285.4338	22.75
285.4438	4.5	285.465	25
285.4738	6.5	285.4975	18
285.5088	8	285.5313	20.25
285.5388	4.75	285.5638	21
285.5688	4.75	285.5975	18
285.605	6.75	285.6288	14.75
285.64	5.75	285.6675	19.75
285.67	7.75	285.6975	22.75
285.7063	7	285.7325	21.25
285.74	6.25	285.77	22.75
285.7738	7.25	285.8	18
285.805	6.75	285.83	15.5
285.8363	10	285.8625	16.25
285.8763	9.75	285.895	16.75
285.9075	8	285.9338	15.75
285.9438	10.75	285.965	14.5
285.9738	8.25	285.9975	14.25
286.0088	6	286.0313	19.25
286.0388	9	286.0638	12.5
286.0688	9.75	286.0975	17.25
286.105	7.5	286.1288	18
286.14	7.25	286.1675	15
286.17	9.5	286.1975	16
286.2063	9	286.235	20.5
286.24	9	286.27	15.25
286.2738	8.5	286.3	16.75
286.305	8.25	286.33	17.5
286.3363	7	286.3625	16.25
286.3763	9.25	286.3925	17.75
286.4075	8	286.4338	14.25
286.445	9.25	286.465	17
286.4738	10.5	286.4975	15
286.5088	7	286.5313	16.5
286.5388	10.75	286.5638	19.5
286.5688	9.5	286.5975	16.25
286.605	6.75	286.6288	18
286.64	8	286.6663	19.25
286.67	8	286.6975	16.5

286.7063	7.25	286.7325	20.5
286.7413	8.5	286.7675	17
286.7738	8.5	286.8	20.75
286.805	5.5	286.83	20
286.8375	4.75	286.8625	24
286.8763	7	286.8925	23.75
286.9075	7.75	286.9338	22.25
286.9438	6.25	286.965	18.75
286.9738	8.5	286.9975	19
287.0088	6.75	287.0313	20.75
287.0388	4.25	287.0638	22
287.0688	6	287.0975	18.25
287.105	6.25	287.1288	15
287.14	7.75	287.1638	15.5
287.17	3.75	287.1975	18.25
287.2063	6.25	287.2325	17.25
287.2413	6	287.2675	19.5
287.2738	6.75	287.3	18
287.305	6.25	287.33	20.25
287.3375	6.5	287.3638	20.75
287.3763	7	287.3925	22.25
287.4075	8.5	287.4338	17
287.4438	5.5	287.465	18.25
287.4738	5	287.4975	18.25
287.5088	8.5	287.5313	18.5
287.5388	8.5	287.5638	20.5
287.5688	7.25	287.5975	20.75
287.605	8.25	287.6288	23
287.64	9	287.6638	21.25
287.67	10.25	287.6975	23.5
287.7063	9.5	287.7325	21.25
287.7413	6.5	287.7675	22.75
287.7738	7.75	287.8	17
287.805	7	287.8313	19.75
287.8375	10	287.8625	18.75
287.875	7.75	287.8925	20.5
287.9075	9	287.9338	18
287.9438	6.75	287.9688	18.75
287.9738	7.25	287.9975	20
288.0088	10.75	288.0313	24.75
288.0388	10.5	288.0663	22.25
288.0688	8.25	288.0975	17.75
288.105	9.75	288.1288	20.25
288.14	9.5	288.1638	18
288.17	8.5	288.1975	19.75
288.2063	9.25	288.2325	24.25
288.2413	10.75	288.2675	19.25
288.2738	8.5	288.3	18.5
288.305	7.5	288.3313	22
288.3375	9.25	288.3625	20

288.3775	8	288.3925	20.5
288.4075	6	288.4338	24
288.4413	6.25	288.4663	20
288.4763	11.25	288.4975	22.25
288.5088	8.75	288.5313	22.5
288.5388	12	288.5638	19
288.5688	9.5	288.5975	24
288.605	11	288.6288	19.25
288.64	9.25	288.6625	23.5
288.67	8.75	288.6975	21
288.7063	8.75	288.7325	20.5
288.7413	11.5	288.7675	19.25
288.7738	7.25	288.8	21.75
288.805	9.75	288.8313	20
288.84	13.5	288.8625	25.25
288.8763	7.5	288.8925	17.5
288.9088	10	288.9338	20.75
288.9438	11.75	288.9663	23
288.9813	8	288.9975	18.75
289.01	10	289.0313	22.25
289.0425	10.25	289.0663	24.5
289.07	10.5	289.0975	18.5
289.1075	9.5	289.1288	14.5
289.1425	10	289.1625	18.75
289.1738	11.75	289.2	20.25
289.2075	10	289.2325	21.75
289.2438	10.25	289.2675	16.25
289.2763	9.5	289.3	19.75
289.3075	8.75	289.3313	22.5
289.34	11.75	289.3625	13.75
289.3763	11.75	289.3925	17.5
289.4113	11.25	289.4338	17
289.4438	13	289.4663	15.75
289.4788	8.75	289.4975	17.75
289.51	9.75	289.5313	16.25
289.54	10.75	289.5663	15
289.57	11.25	289.5975	17
289.6075	8.5	289.6288	16.25
289.6425	8.25	289.6625	16.75
289.6738	9	289.7	17.5
289.7075	11.5	289.7325	19
289.7438	9.5	289.7675	18.5
289.7763	8	289.8	19.5
289.8075	8	289.8313	18.25
289.84	7	289.8625	18.5
289.8763	9.25	289.8925	22.75
289.9113	7.25	289.9338	19.75
289.9438	9.25	289.9663	19.5
289.9788	8.5	289.9975	21.25
290.01	9	290.0313	22

290.04	10	290.0663	19
290.0725	10.25	290.0975	20.25
290.105	10.25	290.1288	19.25
290.1425	11.75	290.1625	21.75
290.1738	9.5	290.2	19.75
290.21	9.25	290.2325	20
290.2438	9.75	290.2675	19.25
290.2763	11.25	290.3	22.75
290.3075	11	290.3313	21.75
290.34	10	290.3625	16
290.3763	9.25	290.3925	20
290.4113	9.75	290.4363	20.75
290.4425	9.75	290.4663	15
290.4788	11.75	290.4975	17.25
290.51	9	290.5313	19.5
290.54	4.75	290.5663	16.75
290.5725	10.5	290.5975	19.5
290.605	9.25	290.6288	17.5
290.6425	12.25	290.6625	15.75
290.6738	11.25	290.7	20.5
290.71	13.75	290.7325	20.25
290.7438	13.75	290.7675	16.5
290.7763	13.75	290.8013	24.75
290.8075	13.25	290.8313	23.75
290.84	11.5	290.8625	24
290.8763	11.25	290.8925	21.25
290.9113	11.5	290.9338	17.25
290.9425	13.25	290.9663	22
290.9788	9.25	290.9975	19.25
291.01	11.5	291.0313	24.25
291.04	9.5	291.0688	21.75
291.0725	11.25	291.0975	20.75
291.105	10	291.1288	20.75
291.1425	9.5	291.1625	19.5
291.1738	9.5	291.2	17.5
291.21	10.5	291.2363	23.25
291.2463	9.75	291.2688	20.25
291.2775	8.25	291.3038	24.25
291.3088	11	291.3338	27.25
291.3413	10.5	291.365	17.75
291.3788	9.25	291.3975	21.5
291.4138	10.75	291.4325	19.25
291.4475	9	291.4675	18
291.48	10.5	291.5	20.75
291.5125	7.5	291.5338	19.25
291.5425	10.5	291.5675	21
291.575	10.25	291.5988	19
291.6075	10.5	291.63	23
291.645	8	291.6688	25.5
291.675	10.5	291.7025	21

291.7125	9.75	291.7363	22.25
291.7463	9.5	291.7713	25.75
291.7775	10.5	291.8038	24.25
291.8113	16.25	291.8338	22.5
291.8413	13	291.865	22.75
291.8788	11	291.8975	19
291.9138	11	291.9325	23.5
291.9475	11.75	291.9675	20.25
291.98	10.5	292	16.5
292.0125	9.5	292.0363	22
292.0425	10	292.0675	24
292.075	9.5	292.0988	21
292.1075	12.25	292.13	23.25
292.145	10.75	292.165	20.5
292.1775	13	292.2025	18
292.2125	9	292.2363	22.5
292.2463	10.75	292.2688	20.5
292.2775	10	292.3038	20.5
292.31	12	292.3338	21.75
292.3413	12.75	292.365	20.5
292.3763	15	292.3975	21.5
292.4138	11.75	292.4325	21.5
292.4463	14.5	292.4675	21.75
292.48	11	292.5	28.75
292.5125	11.25	292.5363	25.75
292.5425	12.25	292.5675	22
292.575	12	292.5988	19.5
292.6075	13	292.63	23.5
292.645	13.25	292.6663	22.5
292.675	14	292.7063	23.5
292.7125	15	292.7363	22
292.7463	12	292.7688	28
292.7775	12.75	292.8038	26
292.81	12.25	292.8338	25.5
292.8413	12.25	292.865	25.5
292.8763	10.25	292.8975	25.25
292.9138	13.25	292.9313	24.5
292.9463	9.75	292.9675	23.75
292.98	11.75	293	22
293.0125	12.75	293.0363	21.5
293.0425	10.5	293.0675	20.5
293.075	11.5	293.0988	23
293.1075	11.75	293.13	16.25
293.145	12.5	293.1663	19.75
293.1775	12.5	293.2013	21.5
293.2125	9.75	293.2363	24
293.2463	11	293.2688	18.25
293.2775	12	293.3038	16.75
293.31	12.5	293.3338	22.25
293.3413	9.5	293.365	20.75

293.3763	10	293.3975	16
293.4138	11.25	293.4313	18.75
293.4463	13	293.4675	23
293.48	12.5	293.5	17.5
293.5138	8.5	293.5363	20.5
293.5425	11	293.5675	21.75
293.575	7.5	293.5988	21
293.6075	7.5	293.63	22
293.645	10	293.6663	22.5
293.6775	6.5	293.7013	20.25
293.7125	10	293.7363	18.25
293.7463	8	293.7688	21
293.7775	7	293.8038	25.75
293.81	10.5	293.8338	25
293.8413	9	293.865	18
293.8763	9.75	293.8975	21.5
293.9138	11	293.9313	23.5
293.9463	12	293.9675	20.5
293.98	9.75	294	22.75
294.0125	12	294.0363	21
294.045	12	294.0675	18.75
294.075	13	294.0988	21
294.1075	8.5	294.13	19.25
294.1425	9.25	294.1663	17
294.1775	10.25	294.2013	16
294.2125	12.5	294.2363	21.25
294.2463	12.75	294.27	20
294.2775	9.75	294.3038	21
294.31	8.75	294.3338	21
294.3413	8.25	294.365	21.25
294.3763	9	294.3975	18.75
294.415	12.5	294.4313	24.5
294.4463	11.75	294.4675	20
294.48	9.25	294.5	21
294.515	12.25	294.5363	21.5
294.5475	7.75	294.5675	20.25
294.5775	10	294.5988	18.75
294.6113	10.25	294.63	21.75
294.645	11	294.6663	21.75
294.68	11.25	294.7	22.5
294.715	10.5	294.7363	17
294.7488	9.25	294.77	22.25
294.7788	13	294.8038	21.5
294.8113	12	294.8338	18
294.8425	10.5	294.865	20.5
294.8788	8.75	294.8975	27.25
294.9163	9.25	294.9313	26.5
294.9488	10	294.9675	22.25
294.9813	12.75	295	28.5
295.015	8.75	295.0363	22.5

295.0475	10	295.0675	21.5
295.0775	12.5	295.0988	21.5
295.1113	9.75	295.13	23.75
295.145	8	295.1663	18.25
295.18	9.75	295.2	19.25
295.2125	8.5	295.2363	23.25
295.2488	9.5	295.2713	20.5
295.28	10.25	295.3038	21.25
295.3113	8.75	295.3338	22.25
295.3425	11.25	295.3663	19.25
295.3813	9.5	295.4	20.25
295.4163	9.25	295.4313	22
295.4475	10.75	295.465	23
295.4813	10	295.5	24.75
295.515	11	295.5363	21.25
295.5475	10.75	295.5675	18.75
295.5775	10.5	295.5988	17.5
295.6113	10	295.63	22.25
295.6475	8.25	295.6663	22.25
295.68	10.75	295.7	21.5
295.7125	8.5	295.735	20.5
295.7488	9.75	295.77	20.25
295.78	11.5	295.8038	20
295.8113	9.5	295.8338	19.25
295.8425	8.25	295.865	19.25
295.8813	8.75	295.9	23
295.9163	10.25	295.9325	19.75
295.9475	8	295.965	20.75
295.9813	11	296	26.25
296.015	12.5	296.0363	26.25
296.0475	11	296.0675	26
296.0775	13.75	296.0988	20
296.11	12	296.13	17.75
296.1475	9.25	296.1663	21.75
296.18	11.25	296.2	22.25
296.2125	10.75	296.235	22.25
296.2488	10.25	296.27	23
296.28	12.25	296.3038	23.25
296.3113	10.5	296.3338	23.25
296.3425	13.5	296.365	22.75
296.3825	10.25	296.4	23.75
296.4163	12.75	296.4325	23.25
296.4475	13	296.4625	19.25
296.4813	13.5	296.5025	22.25
296.5163	10.25	296.5388	27.25
296.5475	12.25	296.5675	21.5
296.5775	12.5	296.5988	20.75
296.61	12.25	296.63	25.25
296.6475	10.75	296.6663	25
296.68	13.25	296.7	19.25

296.7125	13.25	296.735	22
296.7488	14	296.77	20.25
296.78	9	296.8038	19.75
296.8113	12.75	296.8338	19.75
296.8425	10	296.865	24.75
296.8825	8.5	296.9025	18
296.9163	12	296.9325	22.75
296.9475	13.5	296.9625	23.5
296.9838	11	297.005	22.75
297.0175	11.25	297.0363	23
297.0475	14.5	297.0675	18
297.0775	10	297.0988	23.5
297.11	13.25	297.13	23.75
297.1475	12	297.1663	19.25
297.18	11	297.2	24
297.2125	9.75	297.235	20.75
297.2488	9.75	297.27	20.5
297.28	13.25	297.3038	23.25
297.3113	11.75	297.3338	19.25
297.3425	7.5	297.3675	19.5
297.3825	12.25	297.4	21.25
297.4163	8	297.4325	19.5
297.4475	13.5	297.4625	23
297.4813	12.5	297.505	21.75
297.5175	11	297.5363	23.25
297.5475	11	297.5675	21.5
297.5775	11	297.5988	18.5
297.61	10.5	297.63	21.5
297.6475	10	297.6663	23.25
297.68	10.25	297.7	23.75
297.7125	11.25	297.7338	22.75
297.7488	11.25	297.77	26.25
297.78	14.25	297.8038	22.5
297.8113	14	297.8338	16.5
297.8425	13.5	297.865	19.5
297.8825	14.25	297.9	16.75
297.915	14	297.9325	17.25
297.9488	15.5	297.9625	16.75
297.9813	13	298.0025	18.5
298.0175	14.5	298.0363	20.25
298.0475	15	298.0675	19.5
298.0775	14.75	298.0988	15
298.1138	13.25	298.13	15.5
298.1463	13	298.1663	19
298.18	14	298.2013	15.75
298.2125	12.25	298.2313	17.25
298.2488	12	298.27	20
298.28	9.25	298.3038	21.75
298.3113	12	298.3338	20.25
298.3425	17	298.365	20.5

298.3825	10.75	298.4	21.25
298.415	12.75	298.4325	25.75
298.4475	9.75	298.465	19
298.4813	10.75	298.5025	20
298.5175	11.75	298.535	20.75
298.5475	11.25	298.5675	18.25
298.5775	14	298.6013	19.75
298.6138	14.5	298.6313	15
298.6463	15.75	298.6663	15.5
298.68	14	298.7	20.5
298.7125	15.5	298.7313	23
298.75	15.5	298.77	16.75
298.78	12.75	298.8038	17
298.8113	10.5	298.8338	20.5
298.8425	16.75	298.865	16
298.8825	14.25	298.9	18
298.915	14.75	298.9325	21.5
298.9475	11.75	298.9625	18.5
298.9813	12.75	299.0025	17.5
299.0175	12	299.035	18.25
299.0475	11.25	299.0675	20.75
299.0775	11.5	299.0988	19
299.1138	11.5	299.1313	18.5
299.1463	11	299.1663	17.5
299.18	10.75	299.2	20.25
299.2125	12.75	299.2325	18.25
299.25	9.5	299.27	19.5
299.28	11.5	299.3038	15
299.3113	12.5	299.3338	20
299.3438	13	299.365	19.5
299.3825	11	299.4	18.5
299.415	12.75	299.4325	22.25
299.4475	13	299.4625	19.75
299.4813	10.25	299.5013	21.25
299.5188	11.5	299.5375	20.25
299.5475	9	299.57	22
299.5775	11.5	299.6	20.25
299.6138	8.25	299.6325	16.5
299.6463	9.5	299.6675	17.25
299.68	7.25	299.7013	18.75
299.71	9.25	299.7338	19
299.75	9	299.7713	16.25
299.78	9.25	299.805	19.5
299.8113	8	299.8363	18.25
299.8438	10	299.8675	19.25
299.8825	19	299.9025	21.25
299.915	7.5	299.935	15.75
		299.965	18
299.9813	24.5	300.005	20
300.0175	20	300.0375	17.25

300.0475	47.5	300.07	17.5
300.0775	9	300.1	16.5
300.1138	27.25	300.135	18.5
300.1463	26.25	300.1675	19.25
300.18	11	300.2013	16.5
300.21	31	300.235	16.25
300.25	16.25	300.2713	18.25
300.28	10.75	300.305	16.75
300.3113	21.5	300.3363	21.25
300.3438	9.75	300.3675	24.75
300.3825	14.5	300.4025	18.75
300.415	17	300.435	19.5
300.4475	10.75	300.465	21.75
300.4838	12.5	300.505	26.5
300.5175	11.25	300.5375	22.25
300.5475	13.25	300.57	22.5
300.5775	13.5	300.6	20.75
300.6138	13.75	300.635	23
300.6463	10	300.6675	18.25
300.6775	11.75	300.7013	20.5
300.7125	10.75	300.735	20.5
300.75	12	300.7713	26.25
300.78	9.75	300.805	25
300.8113	10.5	300.8363	20
300.8463	11.25	300.8675	25.5
300.8825	7.75	300.9025	26.25
300.915	10.5	300.935	26.75
300.9475	13.75	300.965	19.25
300.9838	12	301.0025	20.25
301.0175	13.75	301.0375	17.5
301.0475	11.25	301.07	19
301.08	9.5	301.1	18.25
301.1138	11.75	301.135	17.75
301.1463	11	301.1675	17.75
301.1775	12.5	301.2013	17
301.21	11	301.235	21.75
301.25	13.25	301.2713	18
301.28	12.5	301.305	16
301.3113	14.25	301.3363	17.5
301.3463	9.25	301.3675	17.75
301.3825	10	301.4025	25.75
301.415	11.25	301.435	20.5
301.4475	10.5	301.465	14.25
301.4838	9.25	301.5	16.75
301.5175	9	301.5375	14.75
301.5475	11	301.57	19.5
301.58	9.75	301.6	19.5
301.6138	12.25	301.635	19.25
301.6463	11	301.67	20.5
301.6775	9.25	301.7013	21.75

301.71	10.25	301.735	13.75
301.75	13.25	301.7713	21.75
301.78	8.75	301.805	25.25
301.8113	11.25	301.8363	22.75
301.8463	8	301.8675	22.75
301.8825	9.75	301.9025	19.75
301.915	11.75	301.935	22.75
301.9475	10	301.965	16.5
301.9838	8.75	302	19.25
302.0175	14.75	302.0375	22.75
302.0475	12	302.07	20.5
302.08	17	302.1025	17.25
302.1138	14.25	302.1375	17.75
302.1463	13.75	302.1725	13.75
302.1775	12.25	302.2038	16
302.215	15.5	302.2375	13.75
302.25	20.75	302.2738	13.5
302.28	11.5	302.3075	16
302.3138	12.25	302.3388	18
302.3463	12.75	302.3688	19
302.3825	13	302.4038	15
302.415	13.5	302.4363	19.25
302.445	14.25	302.4688	21.25
302.4838	9.75	302.5013	16.75
302.5175	13.5	302.5388	21
302.5475	12.5	302.5713	19
302.58	10.75	302.6025	16.5
302.6138	11	302.6375	16
302.6463	13.25	302.6725	14.25
302.6775	12.25	302.7038	17.25
302.715	13.75	302.7375	18.5
302.75	13.5	302.7725	15.5
302.78	13.75	302.8075	17.25
302.8138	11	302.8388	17
302.8463	14.75	302.8688	15.5
302.8825	10.75	302.9038	19
302.915	11.5	302.9363	19
302.945	11.5	302.9688	16.5
302.9838	10.75	303.0013	14.5
303.0175	12.75	303.0388	17.25
303.0475	13	303.0713	17.25
303.0825	15.25	303.1025	16.5
303.1138	13.5	303.1375	18.5
303.1463	17	303.1725	13.75
303.1775	17.5	303.2038	18.75
303.215	15.75	303.2375	14.75
303.25	12.25	303.2725	13.75
303.28	14	303.305	18.5
303.3138	11.75	303.3388	18.5
303.3463	13.25	303.3688	16

303.3825	15.75
303.415	13.25
303.445	15
303.4838	16.75
303.5175	14.25
303.5488	14
303.5825	13.25
303.6138	12.25
303.6463	15.25
303.6775	14.75
303.715	12.75
303.75	12
303.78	9.25
303.8138	10.75
303.8463	8
303.8825	11
303.915	12.25
303.9463	10.5
303.9838	12.5
304.0175	13.25
304.0488	12.75
304.0825	13.5
304.1138	11
304.1463	12.5
304.1775	13
304.2138	9.75
304.25	12
304.28	11
304.3138	12.5
304.3463	13.25
304.3825	11.75
304.415	12
304.4463	9
304.4838	12.5
304.5175	10.75
304.5488	11.25
304.5825	11.25
304.6138	10.25
304.6463	12.75
304.6775	13.25
304.7138	13.5
304.75	8
304.78	15.5
304.8138	11.25
304.8463	13.75
304.8825	15.5
304.915	15.5
304.9463	14.5
304.9838	15
305.0175	18

303.4038	17
303.4363	16
303.4688	17.5
303.5013	14
303.5388	15.25
303.5713	17
303.6025	15.75
303.6375	20.75
303.6725	18
303.7038	19.75
303.7375	19
303.77	18.5
303.805	19.5
303.8388	21.5
303.8688	25.25
303.9038	27.5
303.9363	22.5
303.9688	30
304.0013	28.75
304.0363	16.5
304.0713	28
304.1025	18.25
304.1375	23.5
304.1725	20.5
304.2038	16
304.2375	21.75
304.27	22.5
304.305	17.5
304.3388	20.5
304.3713	22.25
304.4038	17.5
304.4363	13.75
304.4688	21.75
304.5013	27.25
304.5363	15.5
304.5713	13
304.6025	20.25
304.6375	15.75
304.6725	19.75
304.7038	18
304.7375	18
304.77	15.75
304.805	24.25
304.8388	26
304.8688	18.75
304.9038	20
304.9363	19
304.9688	26
305.0013	18.25
305.0363	27.5

305.0488	15.25
305.0825	14.75
305.1138	15.25
305.1463	14.5
305.1775	15
305.2138	11.25
305.25	15
305.2825	14
305.3138	13
305.3463	14.25
305.3825	12.75
305.415	14.5
305.4463	13
305.4838	16
305.5175	11.75
305.5488	14.5
305.5825	12.75
305.6138	14.5
305.6463	13.25
305.68	13.5
305.7138	18.25
305.75	14
305.7825	13.5
305.815	16.5
305.8463	12
305.8825	12.75
305.915	13
305.9475	10.5
305.9838	11.25
306.0175	10
306.0488	14.25
306.0825	12.25
306.1138	14.25
306.1463	10.5
306.18	13.25
306.2138	13.25
306.25	12.75
306.2825	12.25
306.315	15
306.3463	11.75
306.3825	14.5
306.415	12.5
306.4475	15.5
306.4813	15.75
306.5188	14.25
306.5488	15
306.5825	14.25
306.6163	19
306.6488	13
306.6813	14

305.0713	27.25
305.1025	21.5
305.1375	19.25
305.1725	25
305.2038	27.5
305.2375	17.5
305.27	19
305.305	15
305.3388	18.5
305.3688	15.75
305.4038	19.25
305.4363	19
305.4688	34.25
305.5013	22.5
305.5363	17.75
305.5713	30.25
305.6025	21.5
305.6375	29
305.6725	19.75
305.7038	24
305.7375	28.5
305.77	20.75
305.8038	18
305.8388	23.75
305.8688	20
305.9038	21.25
305.9363	22.25
305.9688	22.75
306.0013	25.25
306.0363	21.25
306.07	22.5
306.1025	23.75
306.1375	20.75
306.1725	24.5
306.2038	30.25
306.2388	23.5
306.27	22
306.3038	24
306.3388	20.5
306.37	41.75
306.4038	32.5
306.4363	21.5
306.4688	20.75
306.5038	16.5
306.5363	17
306.57	19.25
306.6025	28.5
306.6363	25
306.6725	23.5
306.7038	16.5

306.7163	17	306.7388	24
306.7525	14.25	306.77	19.25
306.785	11.5	306.8038	18.25
306.8175	11.5	306.8388	24.75
306.8488	9.75	306.87	19
306.885	15.75	306.9038	20.25
306.92	12.5	306.9363	26
306.9513	14	306.9688	17.75
306.985	10	307.0013	16.25
307.02	11.75	307.0375	16.25
307.0513	13.75	307.0675	15.25
307.085	12.5	307.1025	16.5
307.1188	13	307.1363	21.25
307.1513	12.5	307.1725	19.5
307.1838	10	307.2038	19
307.22	11	307.2388	19.5
307.255	11.25	307.27	21
307.2875	10.75	307.3038	17.75
307.32	9.5	307.3388	18.5
307.3513	11.5	307.37	27.25
307.385	11.25	307.4038	24.75
307.42	8.25	307.4363	19.25
307.4513	10.25	307.4688	22.5
307.485	11.5	307.5013	20.5
307.5213	14.25	307.5363	17.5
307.5513	6.25	307.5675	17
307.5875	11.5	307.6025	21.5
307.6188	8.5	307.6363	23
307.6513	7.75	307.6725	20.5
307.6838	9.25	307.7038	17.75
307.72	13	307.7388	19.75
307.7538	9.75	307.77	24
307.7875	12.25	307.8038	33.75
307.82	10.5	307.8388	38.75
307.8513	9	307.87	30.25
307.885	12	307.9038	22.75
307.9175	12	307.9363	20.25
307.9513	9.5	307.9688	27.5
307.985	9.75	308.0013	31.75
308.0213	12.25	308.0363	19.75
308.0513	11.25	308.0675	17.5
308.085	11	308.1025	18.75
308.1188	9.25	308.1363	22.75
308.1525	13.5	308.1725	31
308.1838	10.5	308.2038	28
308.22	14.25	308.2388	20.25
308.2538	13.5	308.27	16.5
308.2875	11.25	308.3038	18.5
308.32	12.5	308.3363	21.75
308.3513	14	308.37	18.25

308.385	11	308.4038	19.5
308.42	14.25	308.4363	20
308.4538	12.75	308.4688	22.25
308.485	11.5	308.5013	20
308.5213	13.5	308.5363	25.5
308.5513	13.75	308.5675	22.5
308.585	11.25	308.6025	16.5
308.6188	10.5	308.6363	31.25
308.6525	10.25	308.6725	36.25
308.6838	11.75	308.7038	39.75
308.72	10.25	308.7388	34.25
308.7538	9	308.77	18.75
308.7875	8	308.8038	16.75
308.82	9.25	308.8338	15.75
308.8513	12.25	308.87	18.5
308.885	11.25	308.9038	24
308.9175	12	308.9363	21.5
308.9538	13.25	308.9713	21.5
308.99	10	309.0013	20
309.0213	11.75	309.0363	20.75
309.0513	12.75	309.0675	20.25
309.085	11.75	309.1025	15.25
309.1188	9	309.1363	21.5
309.1525	13.75	309.1725	33
309.1863	12.5	309.2038	21
309.22	12.75	309.2388	17.5
309.2538	11.75	309.27	19.75
309.2875	11	309.3038	17.75
309.32	12.75	309.3338	21.75
309.3513	6.75	309.37	20
309.385	9.25	309.4063	18.25
309.4175	11.25	309.4363	21.25
309.4538	11.25	309.4713	17.75
309.485	11	309.5013	21.75
309.5188	12	309.5363	18.75
309.5513	12.5	309.5675	22.25
309.585	7.5	309.6025	25.75
309.6188	9.75	309.6363	26
309.6525	11.5	309.6725	16.5
309.6863	13	309.7038	21
309.72	11.5	309.7388	15
309.7563	9.5	309.77	15.25
309.79	12.5	309.8038	17.25
309.82	9	309.8338	18.25
309.8513	9.5	309.87	22.25
309.885	11.25	309.9063	13
309.9175	7.25	309.9363	17.25
309.9538	8.75	309.9713	16.75
309.985	11.5	310.0013	20.25
310.0188	8.75	310.0363	14.5

310.0513	11	310.0675	30
310.085	10.5	310.1038	23
310.1188	9.5	310.1363	32.75
310.1525	8.75	310.1725	24
310.1863	10.25	310.2038	32.25
310.22	9.5	310.2388	47.75
310.2563	9	310.27	28.75
310.2875	9.25	310.3038	17.5
310.32	11.5	310.3338	22.25
310.3513	12.25	310.37	12.75
310.385	10.25	310.4063	24.25
310.4175	10	310.4363	17.75
310.4538	9.25	310.4713	17
310.485	12.25	310.5013	18.75
310.5188	10.5	310.5363	22.25
310.5513	10	310.5675	38.5
310.585	11.25	310.6025	29.5
310.6188	9.75	310.6363	20.5
310.6525	14.25	310.6725	14.75
310.6863	11.75	310.7038	16.75
310.72	9.75	310.7388	21
310.7563	12.25	310.77	30
310.7875	16.5	310.8038	22.75
310.82	10.75	310.8338	15
310.8513	12.5	310.87	21.75
310.885	14	310.9063	22.75
310.9175	12.5	310.9363	19.75
310.9538	7.5	310.9713	18.25
310.985	11.5	311.0013	27.75
311.0188	11.75	311.0363	34.25
311.0513	11	311.0675	43.25
311.085	11.75	311.1025	37.25
311.1188	12	311.1363	17.5
311.1525	11.5	311.1725	16.75
311.1863	11	311.205	23
311.22	15.5	311.2388	22
311.2563	10	311.27	31.25
311.2875	10.75	311.3038	22.75
311.32	13.25	311.3338	19.75
311.3513	10.5	311.37	17.25
311.385	12.5	311.4063	17
311.42	10.75	311.4363	28
311.4538	13.25	311.4713	19
311.485	13.75	311.5013	18.25
311.5188	13.75	311.5363	24.75
311.5513	12.75	311.5675	24.75
311.585	13.25	311.6025	25
311.6188	10	311.6363	25.75
311.6525	12.75	311.6725	23.5
311.6863	11.5	311.705	24.75

311.72	13.75	311.7388	24.75
311.7563	10	311.77	28
311.7863	16.5	311.8038	29
311.82	14.5	311.8338	24
311.8513	14	311.8675	26.5
311.885	15	311.9063	23.75
311.9175	14.5	311.9363	33.75
311.9538	16	311.9713	41
311.985	13.25	312.0013	36.25
312.0188	14.75	312.0363	26.75
312.0513	14.5	312.0675	21.25
312.085	13.75	312.1025	27.25
312.1188	13.75	312.1363	26.75
312.1525	10.5	312.1725	25.75
312.1863	12.5	312.2075	27
312.22	16	312.2388	24.25
312.2563	11	312.27	23.25
312.2863	12.5	312.3038	33.75
312.32	11	312.3338	33.5
312.3513	14.5	312.3675	22
312.385	11.25	312.4063	19.25
312.4188	11.5	312.4363	20.5
312.4538	12	312.4713	22
312.4863	8.75	312.5013	22
312.5188	14.5	312.5363	16.5
312.5513	14.25	312.5675	20.25
312.585	10.75	312.6025	17
312.62	10.5	312.6363	25.5
312.6525	9	312.6725	23.25
312.6863	11.25	312.7075	19
312.72	11.5	312.7388	17.5
312.7563	7	312.77	18.5
312.7863	10	312.8038	22
312.82	10.5	312.8338	18
312.8513	8.25	312.865	24
312.885	6.5	312.9088	20.75
312.9188	8	312.9363	27.5
312.9538	8.25	312.9713	26.75
312.9863	7.25	313.0013	23.75
313.0188	9.25	313.0363	19.75
313.0513	11	313.0675	23.5
313.0875	8.5	313.1025	19.75
313.1213	9.25	313.135	20.75
313.1538	11	313.1725	21.5
313.19	8.75	313.2075	22.25
313.2225	9.75	313.24	17
313.26	11.25	313.27	22
313.2875	11.5	313.3038	19.25
313.3213	10	313.3338	17.75
313.3538	12.25	313.365	19.25

313.3875	12.25	313.4063	18
313.4225	10.5	313.4388	19.25
313.455	13.5	313.4713	20.25
313.4888	14	313.5013	19
313.5213	11.75	313.5363	24
313.5538	11.75	313.5675	22.25
313.5875	13.75	313.6025	18
313.6213	12.75	313.635	16.75
313.6563	12.25	313.6725	19.75
313.6913	13.25	313.7075	17.25
313.7225	10.75	313.7388	17
313.7575	11.5	313.77	23.5
313.7875	9.75	313.8038	18.5
313.8213	11.75	313.8338	23.25
313.8538	12.25	313.865	18
313.8875	8.5	313.9063	18
313.9225	10.25	313.9388	21.5
313.955	10	313.9713	16
313.9888	10.5	314.0013	20.25
314.0213	11.25	314.0363	18.5
314.0538	11.75	314.0675	16.5
314.0875	10.5	314.1025	17.25
314.1213	11	314.135	16.75
314.1563	11.25	314.1725	18
314.19	10.25	314.2088	16.5
314.2225	9.5	314.2388	20
314.2575	12	314.27	18.25
314.2875	10.25	314.3038	20.5
314.3213	12	314.3338	19.75
314.3538	13	314.365	17.5
314.3875	12.5	314.4063	16.25
314.4225	16	314.4388	19.5
314.4575	17.25	314.4713	19
314.4888	14.5	314.5013	19.75
314.5225	12.75	314.5363	20.25
314.5513	17.75	314.5675	19.75
314.5875	15.75	314.6025	16.5
314.6213	13.25	314.635	19.75
314.6575	15	314.6725	18.75
314.6913	12.5	314.7088	17.5
314.7263	10.75	314.7388	19
314.7588	14.5	314.77	21.25
314.7888	10.25	314.8038	18.75
314.8238	12.25	314.835	22.75
314.8588	13	314.865	16.5
314.89	14.75	314.9038	18.75
314.925	10.5	314.9388	18.5
314.9575	13.75	314.9713	20
314.9913	10.75	315.0013	27
315.0238	13.75	315.0363	20.75

315.0538	12.75	315.0675	21.25
315.0888	12.5	315.1025	23
315.1225	9	315.1338	20.25
315.1575	12.25	315.1725	18.75
315.1925	14.75	315.2075	19.25
315.2263	12.5	315.2388	21.25
315.2588	14	315.27	21
315.2888	15	315.3038	17.75
315.3238	12.75	315.335	18
315.3588	12	315.365	18.25
315.39	11.75	315.4038	15
315.425	12.5	315.4388	19.75
315.46	12.75	315.4713	22
315.4913	11	315.5013	20
315.5238	9	315.5363	28
315.5538	12.75	315.5675	32.5
315.5888	9.5	315.6025	32
315.6225	11	315.6338	33
315.66	13.75	315.6725	36.5
315.6913	11	315.7075	23
315.7263	13	315.7388	22
315.7588	16	315.77	24.25
315.7888	8	315.8038	19.25
315.8238	11	315.835	23.5
315.8588	14.25	315.865	22.75
315.89	13	315.9013	38
315.925	15.75	315.9388	39
315.96	10.75	315.9713	42
315.9913	10.75	316.0013	21.75
316.0238	10.25	316.0363	24.75
316.0538	13.5	316.0675	23
316.0888	11.5	316.1025	23.5
316.1225	10.5	316.1338	20
316.16	7.5	316.1738	23
316.1913	9.75	316.2075	28.75
316.2263	10	316.2388	27
316.2588	11	316.27	21.5
316.2888	11.75	316.3038	20.25
316.3238	13.75	316.335	21.25
316.3588	12	316.365	18
316.39	14.25	316.4013	21
316.4275	13.25	316.4388	21.75
316.46	12.25	316.4713	29
316.4913	12.25	316.5013	27
316.5238	11.5	316.5363	24.5
316.5538	12.25		
316.5913	11.5		
316.6225	11.5		
316.66	17.75		
316.6913	13.5	316.7075	50

316.7263	9	316.7388	22.75
316.7588	14.25	316.77	39
316.7888	11.25	316.8038	36.5
316.8238	11	316.835	33
316.8588	15	316.865	26.75
316.89	15.25	316.9013	23.75
316.9275	18	316.9388	26.5
316.96	17.25	316.9713	52.5
316.9913	14		
317.0238	15		
317.0538	13.75		
317.0913	12.75		
317.1225	13		
317.1575	10.5		
317.1913	10	317.2075	37.5
317.2263	12.25	317.2388	40.5
317.2588	14.25	317.27	54.25
317.2888	12	317.3038	21.25
317.3238	13.25	317.335	43.25
317.3588	16.5	317.365	46
317.39	13.75	317.4013	21.5
317.4275	12.5	317.4388	26.75
317.46	16.5	317.4713	23.75
317.4913	11.75	317.5013	27.25
317.5238	12	317.5363	23.25
317.5538	11.5	317.5675	20
317.5913	13	317.6025	41
317.6225	14.5		
317.6575	14.5		
317.6913	15.5	317.7075	47.25
317.7263	12.5	317.7388	23.75
317.7588	10.25	317.77	30.75
317.7888	11.5	317.8038	45
317.8238	12.75	317.835	34.75
317.8588	12.75	317.865	19.75
317.89	12.5	317.9013	24
317.9275	11.75	317.9388	35.5
317.96	14.25	317.9713	47
317.9913	10.25		
318.0238	15.75	318.0363	55.25
318.0538	15.25	318.0675	27.75
318.0913	13.25	318.1025	23.75
318.1225	13.5	318.135	40.75
318.1575	16.5	318.1713	26
318.1938	10.75	318.2075	25.75
318.2263	12.5	318.2388	29.75
318.2588	16.5	318.27	39.5
318.2888	12.75		
318.3263	13.5		
318.3588	15	318.365	23.75

318.3913	13.5	318.3988	29.5
318.4275	14.75	318.4388	19.75
318.46	15	318.4713	26.75
318.4913	13.5	318.5013	21.25
318.5238	15.5	318.5363	26.25
318.5538	13	318.5675	23.75
318.5913	13.25	318.6025	22.25
318.6225	10.75	318.635	23.75
318.6575	11.75	318.67	47.25
318.6938	16.25	318.7075	53.5
318.7263	12.75	318.7388	20.25
318.7588	9	318.77	55
318.7888	13.25	318.8038	37
318.8238	13.25	318.835	19.25
318.8588	16.25	318.865	39.5
318.8913	13.75		
318.9275	13.25		
318.96	11.75	318.9713	48.25
318.9913	13.5	319.0013	39.5
319.0238	12.75	319.0363	22.5
319.0538	12.5	319.0675	23.75
319.0913	15.25	319.1025	29.25
319.125	15.5	319.135	39
319.1588	11	319.17	35.75
319.1938	14	319.2075	27.25
319.2263	10	319.2388	49
319.2588	14.25	319.27	39.5
319.2888	12.5	319.3038	27.75
319.3263	8.75	319.335	24.5
319.3588	11.25	319.365	38
319.3913	13.25	319.4038	43.25
319.4275	10	319.4388	22.75
319.46	13.25	319.4713	43.5
319.4913	10.5	319.5013	38.75
319.5238	10.5	319.535	21.75
319.5538	12.5	319.57	21.25
319.5913	11	319.6025	38.25
319.6225	14.25	319.635	26.75
319.6588	12.5	319.67	24
319.6938	14	319.7075	22
319.7263	15	319.7388	21
319.7588	13.75	319.77	21.5
319.7888	14.75	319.8038	17.25
319.825	13	319.8363	19.75
319.8588	13	319.8675	17
319.8913	12.5	319.9038	19.5
319.9275	17.25	319.9413	17.25
319.96	17.25	319.9738	17
319.9913	10.75	320.0038	17.75
320.0238	15	320.0388	18.75

320.0538	10	320.0725	20
320.0913	15	320.105	26.5
320.1225	13.75	320.1363	21
320.1588	16.75	320.1713	19.5
320.1938	13.5	320.2088	18
320.2263	11.75	320.24	15.5
320.2588	13.25	320.2725	22
320.2888	15	320.305	21.5
320.325	13.75	320.3363	22.5
320.3588	10	320.3675	18.25
320.3925	11	320.4038	22.25
320.4275	13.5	320.4413	17.25
320.46	10.75	320.4738	21.25
320.4913	13.75	320.5038	16
320.5238	11.5	320.5388	20.25
320.5538	14.75	320.5725	17.75
320.5913	9.75	320.605	19.75
320.625	14.75	320.6363	16
320.6588	11.5	320.6713	17.5
320.6938	9.75	320.7088	17.5
320.7263	14.25	320.74	18.5
320.7588	11.5	320.7725	20.75
320.7888	11.5	320.805	18.75
320.825	12.25	320.8363	21
320.8588	10.5	320.8675	19.25
320.8913	13.25	320.9038	20.5
320.9275	11.25	320.9388	20
320.96	10.75	320.9738	20.25
320.9913	12.5	321.0038	23.5
321.0238	12.5	321.0388	22.75
321.0563	9	321.0725	19.75
321.0913	12.5	321.105	21.75
321.125	13.5	321.1363	23.25
321.1588	10.5	321.1713	24.25
321.1938	11	321.2088	25
321.2263	9	321.24	23.75
321.2588	11.25	321.2725	19
321.2888	10.5	321.305	19
321.325	9.75	321.3363	22
321.3588	12	321.3675	20.25
321.3938	12.25	321.4038	21.75
321.4275	12.25	321.4375	19.5
321.46	12.25	321.4738	21.25
321.4913	13	321.5038	24.5
321.5238	11.5	321.5388	21.75
321.5563	11.5	321.5725	22.75
321.5913	11.75	321.605	20.25
321.625	11.5	321.6363	18.5
321.6588	10	321.6713	20.75
321.6938	13.75	321.71	16.75

321.7263	11.5	321.74	18.25
321.7588	9.75	321.7725	20
321.7888	8.75	321.805	22
321.825	13.5	321.8363	17
321.8588	9.25	321.8675	19.75
321.8938	9.25	321.9038	19
321.9288	14	321.9388	18
321.96	11.25	321.9738	15.25
321.9913	11.25	322.0038	15.5
322.0238	12.25	322.0388	20
322.0563	13.25	322.0725	25
322.0913	12	322.105	22.5
322.125	10	322.1363	16.25
322.1588	10.25	322.1713	19.25
322.1938	10	322.2088	15
322.2263	11.75	322.24	21.5
322.2588	11.25	322.2725	18
322.2888	10.25	322.305	12.5
322.325	9.5	322.3363	14.75
322.36	10.75	322.3675	15
322.3938	10.75	322.4038	19.25
322.4275	12	322.4375	20.25
322.4613	8.5	322.4738	16
322.4925	9.25	322.505	18.25
322.525	10.75	322.5388	18.75
322.5588	11.5	322.575	16.5
322.5938	13	322.6075	19.75
322.6275	8.5	322.6413	18.5
322.6625	11.5	322.6738	15.75
322.6963	11.75	322.7088	16.75
322.7288	11.5	322.7438	17.5
322.7613	11.75	322.775	15.75
322.7913	11.5	322.8063	18.75
322.8263	10.25	322.8375	16.25
322.8613	9.5	322.8688	17
322.895	11	322.905	18.25
322.9313	12.25	322.9388	15
322.9613	10.25	322.975	19
322.9925	11.75	323.0075	20.25
323.025	10	323.0413	16.75
323.0588	10.75	323.0775	19.75
323.0938	10.25	323.1088	16.75
323.1275	10.5	323.1425	19
323.1625	14.5	323.1763	19.5
323.1975	10.75	323.2113	17.5
323.2288	9.75	323.2438	16.25
323.2613	13.5	323.2763	22
323.2913	12.5	323.3075	17.5
323.3263	15.5	323.34	20
323.3613	17.25	323.3738	21.25

323.395	12.5	323.4088	17.5
323.43	13.75	323.44	15
323.4613	11.75	323.4775	18.25
323.4925	11.25	323.5075	19.25
323.525	14.25	323.5413	12.5
323.5588	11.75	323.5788	19.25
323.5938	8.5	323.6088	18
323.6288	9	323.645	19.75
323.6625	12	323.6788	15.25
323.6963	8.5	323.7113	19
323.7288	10.5	323.7438	17.75
323.7613	8.75	323.7763	15.5
323.7913	13.75	323.8088	17.25
323.8263	12.75	323.84	14
323.8613	13.25	323.8738	13
323.895	9.5	323.9088	15.25
323.93	9.5	323.94	11.75
323.9613	10.5	323.9763	15.5
323.9925	12.25	324.0075	16
324.025	13	324.0413	15
324.0588	13.25	324.0775	15.25
324.0963	12.25	324.1088	16.5
324.1288	13.25	324.145	19.5
324.1625	10.5	324.1763	17.25
324.1963	11	324.2113	15
324.2288	13.75	324.2438	19
324.2613	11	324.2763	18.25
324.2913	9.75	324.3088	19
324.325	11	324.34	21
324.3613	12	324.3738	20
324.3963	10.5	324.4088	19.25
324.43	15.5	324.44	15.75
324.4613	15	324.4763	17.75
324.4925	13.5	324.5075	18.5
324.525	12	324.5413	15.75
324.5588	13.25	324.5775	15
324.5963	10.5	324.6088	18.25
324.6288	13	324.645	12.5
324.6625	13	324.6763	16
324.6963	11.5	324.7113	20.75
324.7288	11	324.7438	12.5
324.7613	11.5	324.7763	16.75
324.7913	12.5	324.8075	17.25
324.825	11.75	324.84	13.75
324.8613	11	324.8738	13.25
324.8975	13.75	324.9088	15.25
324.93	9.5	324.9413	14
324.9613	12.5	324.9763	13.5
324.995	10.25	325.0075	15.5
325.025	12.5	325.0413	16.25

325.0588	13.5	325.0775	16.75
325.0963	10.25	325.1088	18.25
325.1288	10.25	325.145	18
325.1625	11.75	325.1763	17
325.1963	11.5	325.2113	18
325.2288	10.5	325.2438	14.75
325.2613	10.75	325.2763	12.5
325.2913	10.25	325.3075	12.75
325.325	8.5	325.34	9.75
325.3638	8.75	325.3738	10.5
325.3975	12.75	325.4088	14
325.43	8.75	325.44	12
325.4613	13.25	325.4763	10.75
325.4925	8.75	325.5075	12.25
325.525	10.75	325.5413	13.75
325.5613	12	325.5775	9.25
325.5963	12.75	325.61	13.5
325.6288	15.75	325.645	13.25
325.6625	11.25	325.6763	14.75
325.6938	13	325.7113	13.25
325.7288	12	325.7413	14
325.7613	7.75	325.7763	15.5
325.7913	10.75	325.8075	11
325.825	8.25	325.84	12.25
325.8638	11.5	325.8738	14
325.8975	8.75	325.9088	15
325.93	8	325.9413	14.25
325.9613	8	325.9763	10.75
325.9925	8.75	326.0075	15.75
326.025	6	326.0413	12.75
326.0613	8.75	326.075	18
326.0963	7.75	326.1088	18.5
326.1288	10.5	326.145	14.5
326.1625	8.25	326.1763	18.25
326.1938	7.5	326.2113	15
326.2288	8	326.2413	16.75
326.2613	12	326.2763	16.25
326.2913	11.5	326.3075	15
326.3275	10.75	326.34	17.75
326.3638	10.25	326.375	17.5
326.3975	10	326.4088	15.25
326.43	13.75	326.4413	13.75
326.4613	11.75	326.4763	14.75
326.4925	12	326.5075	17.25
326.525	17.25	326.5413	15
326.5613	16	326.575	15.25
326.5988	10.75	326.6088	16.5
326.6288	12.25	326.645	16.75
326.6625	15	326.6763	14
326.6938	9.25	326.7113	18.25

326.7288	11	326.7413	18.5
326.7613	10.5	326.7763	17.75
326.7925	10.25	326.8075	20.5
326.8275	6.5	326.84	17.5
326.8638	10.5	326.875	18
326.8975	16.75	326.9088	17.75
326.93	11	326.9413	14.75
326.9613	10.5	326.9763	13.75
326.9925	12	327.0063	15.75
327.025	9.25	327.0438	16.75
327.0613	12	327.075	20
327.0988	13.5	327.1088	17.25
327.1288	11	327.1475	19.5
327.1625	10.75	327.1763	19.25
327.1938	13.75	327.2113	16.25
327.2288	13.75	327.2413	18
327.2625	11.75	327.2763	16.25
327.2925	10.75	327.31	13.5
327.3275	9.5	327.34	18.75
327.3638	12.75	327.375	19
327.3975	10	327.4088	17.5
327.43	10.5	327.4413	16.75
327.4613	11	327.4763	16
327.4925	8.25	327.5063	16.25
327.525	10	327.5438	14.25
327.5613	10.5	327.575	18.75
327.5988	10.5	327.6088	14.25
327.63	12.75	327.645	19.75
327.6625	11.75	327.6763	18.5
327.6938	12.75	327.7113	18.5
327.7288	11	327.7413	16.75
327.7613	11.5	327.7763	21
327.7925	10	327.8075	17.5
327.8275	10	327.8388	17.25
327.8638	13.75	327.875	14.5
327.8975	11.75	327.9113	17.75
327.93	11.25	327.9425	19
327.9613	12.5	327.9763	15.5
327.9925	9.75	328.0063	15.25
328.025	10.5	328.0438	16.75
328.0613	13.25	328.075	14
328.0963	12.5	328.1088	17
328.13	12	328.145	16
328.1625	11	328.1763	18.5
328.1938	11.75	328.2113	13.25
328.2288	10	328.2413	18
328.2613	10.75	328.2763	16.5
328.2925	9.25	328.3075	15.75
328.3288	8.5	328.3388	14
328.3638	8.25	328.375	16

328.3975	10	328.4088	19
328.43	12.5	328.4413	14.75
328.46	9.5	328.4763	19
328.4925	9.75	328.5063	14.25
328.525	12	328.5438	11.25
328.5625	8.75	328.575	19
328.5963	11.75	328.6113	18.75
328.63	9.5	328.6438	13.5
328.6625	9.75	328.6763	16.75
328.6938	10.25	328.7113	14.5
328.7288	13.75	328.7413	15.25
328.7613	13	328.7738	13.25
328.7925	12	328.8075	13.5
328.8288	10.75	328.8388	16
328.8638	9.75	328.875	14.75
328.8975	9.25	328.9088	17.25
328.93	11	328.9413	17.5
328.96	12	328.9788	17.5
328.9925	10	329.0113	15.25
329.025	12.25	329.045	18.75
329.0625	10.5	329.0763	18
329.0963	13	329.11	21.75
329.13	10	329.1475	26.25
329.1625	9.75	329.1775	26.5
329.195	11.75	329.2125	20.75
329.2288	10	329.2425	24.25
329.2613	14	329.2775	16.5
329.2925	10	329.31	16.75
329.3288	9.25	329.3413	16.75
329.3663	11.25	329.3775	16
329.3975	9	329.4113	26.25
329.43	11.5	329.4463	28.25
329.46	14	329.4788	23.5
329.4938	10.25	329.5088	18.25
329.525	12.5	329.545	32.5
329.565	9	329.5763	13.25
329.5963	12	329.61	23
329.63	9.75		
329.6625	8.75		
329.6938	11	329.7125	32
329.7288	8.5	329.7425	21.5
329.7613	11	329.7775	54.5
329.7938	10.5	329.81	33.25
329.8288	9.75	329.8413	32.75
329.8663	8.5	329.8775	31.25
329.8975	10.5	329.9113	34.25
329.93	10.75	329.9463	29.5
329.96	11	329.9788	22.5
329.9925	10.5	330.0088	27.75
330.025	13	330.045	27.5

330.065	15.25	330.0763	21.5
330.0963	10	330.11	35.25
330.13	14	330.1475	53.25
330.1625	10.25	330.18	39.25
330.1938	10.25	330.2125	17.5
330.2288	10	330.2425	22
330.2613	11.75	330.2775	29
330.2925	11.5	330.31	12.5
330.3288	12.5	330.3425	23.5
330.3663	8.5	330.3775	19.5
330.3975	11.25	330.4113	36
330.43	11	330.4463	33.5
330.46	13	330.4788	46.75
330.4925	8.75	330.5088	53
330.525	8.5	330.5438	19
330.565	9.75	330.5763	36.25
330.5963	10.75	330.61	46.75
330.63	9.25	330.6475	22
330.6625	11.5	330.68	15.75
330.6938	11.5	330.7125	22.75
330.7288	11.5	330.7425	19.5
330.7613	8.75	330.7775	18
330.7925	13	330.81	19.25
330.8288	9	330.8425	15.75
330.8663	9.5	330.875	19.25
330.8975	8.75	330.9113	19
330.93	11.5	330.9463	16.75
330.96	9.5	330.9788	16.25
330.9925	11.5	331.0088	19
331.025	12.5	331.0438	12.5
331.065	12.25	331.0763	16.25
331.0963	10.75	331.11	14.75
331.13	14	331.145	15.5
331.1625	13	331.18	16.5
331.1938	12.25	331.2125	15
331.2288	13	331.2425	24
331.2613	11	331.2775	16
331.2963	13	331.31	14.5
331.3288	10.5	331.3425	18.75
331.3663	10.25	331.375	19
331.3975	11.75	331.4138	18.75
331.43	14	331.4463	22.5
331.46	8.5	331.4788	20.75
331.4925	10.5	331.5113	20
331.525	12.75	331.5438	18.5
331.565	12.25	331.5788	16.75
331.5975	11.75	331.61	20.25
331.63	10	331.645	18.75
331.6625	9.25	331.68	21
331.6938	11.5	331.7125	25.25

331.7263	12.75	331.7425	39.5
331.7613	10.75	331.7775	45
331.7963	13.5	331.81	29.75
331.8288	9.75	331.8425	24.25
331.8663	11	331.875	25.75
331.8975	12.75	331.9138	21
331.93	9.25	331.9463	32
331.96	9.5	331.9788	30
331.9925	9.5	332.0113	18.75
332.025	13.75	332.0438	19.25
332.065	13	332.0788	21
332.0975	13.25	332.11	21.75
332.13	8.25	332.145	15.75
332.1625	14	332.18	26.5
332.1938	14	332.2125	32
332.2263	11	332.2425	32.75
332.2613	13.5	332.2775	22.25
332.2963	11.5	332.31	21.5
332.3288	13.5	332.3425	18
332.3638	12.25	332.3725	18.75
332.3975	11.5	332.4138	15
332.43	13	332.4463	18.25
332.46	6	332.4788	17.75
332.4925	8.25	332.5113	15.5
332.5275	9.5	332.5438	18.75
332.565	7.75	332.5788	20.75
332.5975	10.25	332.61	15
332.63	10.75	332.6438	18.75
332.6625	12.5	332.68	15.75
332.6938	13.25	332.7125	16
332.7263	12.75	332.7425	16.25
332.7613	10.25	332.7775	18.75
332.7963	11.25	332.8075	19.25
332.8288	14.75	332.8425	23
332.8638	10.75	332.8725	16.75
332.8975	8.5	332.9113	18.75
332.93	9.5	332.9463	18
332.96	10.75	332.9788	17.75
332.9925	12.25	333.0113	21.5
333.0275	12	333.0438	21
333.065	11.25	333.0788	19.25
333.0975	10.5	333.11	19.5
333.13	10.25	333.1438	17.75
333.1625	11	333.18	16.75
333.1938	10.75	333.2125	19.75
333.2263	7.25	333.2425	20.25
333.2613	8	333.2775	15.75
333.2963	9	333.31	22.5
333.3313	9	333.3425	20
333.3638	8	333.3725	24.5

333.3975	10	333.4113	29.75
333.43	8	333.4463	19.5
333.46	9	333.4788	16.75
333.4925	10.5	333.5113	12.75
333.53	7.75	333.5438	19
333.565	11	333.5788	23
333.5975	6.75	333.61	18.75
333.63	9.25	333.6438	16
333.6625	11.25	333.68	14
333.6938	13	333.7125	21
333.7263	12.5	333.7425	16
333.7613	11	333.7775	16
333.7963	11.25	333.81	19
333.8313	16.75	333.8425	19.25
333.8638	12.25	333.8725	17.25
333.8975	11.75	333.9113	22.25
333.93	16.25	333.9463	20.5
333.96	14.25	333.9788	16
333.9925	9	334.0113	16
334.0313	12.25	334.0438	17.5
334.065	10.5	334.0788	13.25
334.0975	12.25	334.11	15
334.13	13.25	334.145	15.25
334.1625	13.5	334.18	15.25
334.1938	9.5	334.2125	15
334.2263	11	334.245	15.25
334.2625	9.25	334.2775	18.75
334.2963	11	334.31	11.75
334.3313	10.75	334.3425	15
334.3638	13.25	334.3725	14.75
334.3988	9.25	334.4113	15.75
334.43	14.5	334.4463	18.5
334.46	12.5	334.4788	15.5
334.4925	11.25	334.5113	20.75
334.5313	9	334.545	18.25
334.565	8	334.5763	14.75
334.5975	11.5	334.61	16.5
334.63	9	334.645	14.5
334.6625	10.25	334.6788	17.5
334.6938	12.25	334.7125	19.5
334.7263	14.25	334.7463	17.75
334.7625	13.5	334.7775	19.25
334.7963	9.25	334.81	14.25
334.8313	8.5	334.8425	18.75
334.8638	13.75	334.8725	13.25
334.8988	12.5	334.9113	17.75
334.93	10	334.9463	17
334.96	10.5	334.9788	17.75
334.9925	9.75	335.0113	21
335.0313	10.75	335.045	28.5

335.0663	9.75	335.0763	15.25
335.0975	11	335.11	27.25
335.13	12.25	335.145	24.75
335.1625	12.25	335.1788	14.5
335.1938	9.5	335.2125	14
335.2263	13	335.245	11.75
335.2625	9.5	335.2775	10.25
335.2963	9	335.31	14.5
335.3313	8.5	335.3425	14
335.3638	9.25	335.3725	13.5
335.3988	9.5	335.4088	14
335.43	12.75	335.4463	13
335.46	11	335.4788	18.25
335.4925	11.75	335.5138	13.5
335.5313	9.5	335.545	13.25
335.5663	10.5	335.5763	12.25
335.5975	11.75	335.61	17.75
335.63	10	335.645	16.75
335.6625	6	335.6788	15.25
335.6938	11.25	335.7125	16
335.7263	8.75	335.745	12.5
335.7625	10.25	335.78	14.75
335.7975	12	335.81	17.5
335.8313	13	335.8425	16.5
335.8638	9	335.875	20
335.8988	10.5	335.9088	17.75
335.93	11	335.9463	14.75
335.96	9	335.9788	15.25
335.9913	12.75	336.0138	17.75
336.0313	11.25	336.045	17.25
336.0663	13.25	336.0763	18.25
336.0975	12	336.11	14.75
336.13	12.5	336.145	15.75
336.1625	12.25	336.1788	18
336.1938	13.75	336.2125	13.75
336.2263	10.75	336.245	14.75
336.2625	10	336.28	17.5
336.2975	10.5	336.31	17
336.3313	9.25	336.3425	20.75
336.3638	10.75	336.375	16.5
336.3988	11.25	336.4088	13.5
336.43	9.75	336.4438	16.75
336.46	11	336.48	11.75
336.4938	10.75	336.5113	14.75
336.5313	10.25	336.545	14.5
336.5663	9.5	336.5763	16.25
336.5975	9.75	336.61	11.75
336.63	9.25	336.6425	11.75
336.6625	9.25	336.6788	16.5
336.6938	9.5	336.7125	12.25

336.7263	8	336.745	16.5
336.7625	10	336.78	16
336.8	14.25	336.81	16
336.8313	16.75	336.8425	18.5
336.8638	11.5	336.875	17
336.8988	12.25	336.9088	17.5
336.93	15.5	336.9438	13.5
336.96	13.75	336.9788	17.25
336.9938	14	337.0113	16.25
337.0313	10	337.045	12
337.0663	12.5	337.0763	18.75
337.0975	12.25	337.11	15
337.13	11	337.1425	14.75
337.1625	12	337.1788	13.75
337.1938	14.5	337.215	16.75
337.2263	11.5	337.245	19.25
337.2625	15	337.28	18
337.3	12.75	337.31	19.75
337.3313	15.75	337.3425	14.75
337.3638	12.75	337.375	15.5
337.3988	15.25	337.4088	14.5
337.43	11.5	337.4438	18.75
337.46	14.5	337.48	16.75
337.4938	10.5	337.5125	16.75
337.5313	14.25	337.545	18.25
337.5663	18.25	337.5763	17.25
337.5975	14	337.6113	18
337.63	13.25	337.6425	19.5
337.6625	12.5	337.6788	14.75
337.6938	11.25	337.7125	15.25
337.7263	11.5	337.745	17
337.7625	12	337.78	16.5
337.8	11.25	337.81	15.75
337.8313	12	337.8425	15.5
337.8638	12	337.875	16
337.8988	14	337.9088	17.5
337.93	11.25	337.9438	16.75
337.96	11.25	337.98	16.5
337.9938	13.25	338.0125	16.5
338.0313	13.5	338.045	15.25
338.0663	8.75	338.0763	15.5
338.0975	10.75	338.1125	14.75
338.13	10.75	338.1463	18.75
338.1625	10	338.18	16.5
338.1938	11	338.2138	17.5
338.2288	8.75	338.2463	16
338.265	11.25	338.2825	13.5
338.3013	10.5	338.3125	17.25
338.3313	7.5	338.3438	17
338.3638	9	338.3775	13.25

338.3988	13.5	338.4113	15.5
338.43	11.75	338.4463	16
338.46	9.25	338.4825	11.25
338.4938	15	338.515	12.75
338.5338	15.5	338.5463	13.75
338.5663	11.25	338.5775	10
338.5975	11.25	338.6125	11.75
338.63	11	338.6463	14.25
338.6625	13.5	338.68	13
338.6938	11.75	338.7125	13.75
338.7288	13.5	338.7488	13.75
338.765	15.25	338.7825	15
338.8	13	338.8125	11.5
338.8313	16.5	338.8438	12.75
338.8638	13.75	338.8775	13.5
338.8988	15.25	338.9113	16
338.93	11.75	338.9463	14.75
338.96	11.5	338.9825	11
338.9938	13.75	339.015	13.75
339.0338	12.75	339.0463	13.75
339.0663	11.75	339.0775	13.25
339.0975	12.25	339.1125	16.25
339.1325	13.5	339.1463	10.75
339.1625	13.25	339.18	14
339.1938	10.5	339.2125	11.5
339.2288	12	339.2488	16.25
339.265	12.25	339.2825	13.25
339.3	12.5	339.3125	16.25
339.3313	11	339.3438	13.75
339.3663	13.25	339.3775	14.25
339.3988	11.5	339.4113	17.75
339.43	14.25	339.4463	19
339.46	18.25	339.4825	16
339.4938	15.5	339.515	16
339.5338	14.25	339.5463	15.25
339.5663	11	339.5775	16.75
339.5975	10.5	339.6125	16.5
339.6325	13.75	339.6463	18.75
339.6625	11.5	339.68	15.25
339.6938	8.75	339.7125	18.25
339.7288	12.5	339.7488	17.25
339.765	9.5	339.7825	16.75
339.8	11.25	339.8125	16.5
339.8313	9.5	339.8463	15.25
339.8663	13	339.8775	15.25
339.8988	12.5	339.9113	16.75
339.93	10.25	339.9475	14.25
339.96	10.5	339.9863	18
339.9938	11	340.0163	17.25
340.0338	13.25	340.0475	15.25

340.0663	12	340.0788	15
340.0975	10	340.1138	15.5
340.1325	12.25	340.1475	16.5
340.1625	13	340.1825	22
340.1938	14.75	340.2163	24.75
340.2288	12	340.2513	14.5
340.265	11.5	340.285	18
340.3	14.25	340.315	16.5
340.3313	12.5	340.3488	20
340.3663	13.5	340.38	22
340.3988	12.5	340.4125	18
340.43	12	340.4475	20
340.4613	12.25	340.4863	17.25
340.4938	12.75	340.5163	20
340.5338	12	340.5475	19.25
340.5663	13	340.5788	20.5
340.5975	10.5	340.6138	17
340.6325	10.25	340.6475	19.25
340.6625	12.75	340.6825	19.25
340.6938	14	340.7163	18.25
340.73	10.75	340.7513	20.75
340.7663	14.5	340.785	22.75
340.8	15	340.815	16.5
340.8313	10.25	340.8488	19.75
340.8663	11.5	340.88	18.5
340.8988	16	340.9125	21.5
340.93	13.75	340.9475	19.25
340.9613	16.25	340.9863	20
340.9938	15	341.0163	20
341.0338	13	341.0475	20.25
341.0663	15.5	341.0788	20.75
341.0975	16.25	341.1138	18.75
341.1325	14	341.1475	17.75
341.1625	16	341.1825	20.25
341.1938	10.75	341.2188	16.5
341.2288	12	341.2513	20.25
341.2663	8.25	341.285	19.25
341.3	7.25	341.315	25
341.3313	8.75	341.3488	21
341.3663	10	341.38	16.5
341.3988	8.5	341.4125	21
341.43	7.75	341.4475	21.5
341.4613	9.25	341.4838	21.25
341.4938	10.25	341.5163	20
341.5338	11	341.5475	26.5
341.5663	8.25	341.58	18.25
341.5975	12	341.6138	20.75
341.6325	11.75	341.6475	22.25
341.6625	13.25	341.6825	17.75
341.6938	11	341.7188	22.25

341.7288	7.5	341.7513	17.5
341.7638	10.75	341.785	22.25
341.8	8.25	341.815	24.75
341.8313	11	341.8488	22.75
341.8688	9.5	341.88	21
341.8988	11.75	341.9125	19.75
341.93	12.25	341.95	21.75
341.9613	14	341.9838	19.5
341.9938	10.75	342.0163	20.5
342.0313	12.25	342.0475	16
342.0663	13	342.08	19.75
342.0975	8.5	342.1138	19.25
342.1325	5.5	342.1475	20.75
342.1625	10.5	342.1825	20.5
342.1938	7.25	342.2188	20.25
342.2288	9	342.2525	23.5
342.2638	7.25	342.285	19
342.3	11.75	342.315	19.25
342.3313	10	342.3488	22
342.3688	8.25	342.38	21.5
342.3988	6.75	342.4125	21
342.43	10.25	342.4525	24.5
342.4613	10.5	342.4838	18
342.4938	8	342.5163	21.25
342.5313	9.25	342.5475	20
342.5663	8.5	342.58	20
342.5975	7	342.6138	22
342.6325	7.25	342.6475	21.25
342.6625	7.5	342.6825	21.5
342.6938	7.75	342.7188	19.5
342.7288	10	342.7513	18
342.7638	10.5	342.785	18.5
342.8	5.75	342.815	18.75
342.8313	9.5	342.8488	21.75
342.8688	11	342.88	19.5
342.8988	8.75	342.9125	23.25
342.93	11.5	342.9525	22
342.9613	14.75	342.9838	21.25
342.9938	11	343.0163	21.5
343.0313	11.5	343.0475	22
343.0663	13	343.08	20
343.0975	16.5	343.1138	20.75
343.1325	13.25	343.1475	17.25
343.1625	14	343.1825	21
343.1938	15	343.2188	16.75
343.2288	16	343.2513	18.5
343.2638	12.75	343.285	18.25
343.3	13.25	343.315	14.25
343.3313	14.25	343.3488	20
343.3688	15.5	343.38	16.25

343.3988	13	343.4125	14.5
343.43	11.5	343.4525	18
343.4613	16	343.4838	14.25
343.4963	13.25	343.5163	16.75
343.5313	14.25	343.5475	15.75
343.5663	11.75	343.58	16.5
343.5975	13.25	343.6138	17
343.6325	13.5	343.6475	19
343.6625	10.75	343.6825	17.25
343.6963	10.5	343.7188	20
343.7288	13.75	343.75	19.25
343.7638	11.75	343.785	24.75
343.8	13.25	343.8175	17.75
343.8313	13.25	343.8488	19
343.8688	11.25	343.88	17.25
343.8988	13	343.91	21.75
343.93	15	343.9525	19.25
343.9613	13.5	343.9838	18.75
343.9963	14	344.0163	18.5
344.0313	14.75	344.0475	19.5
344.0663	15.25	344.08	24.75
344.0975	11.5	344.1138	19.5
344.1325	16.5	344.1475	17.5
344.1625	14	344.1838	21.75
344.1963	14.5	344.2188	19.5
344.2288	12.5	344.25	23.75
344.2638	13.75	344.285	18.5
344.3	14.75	344.3175	20
344.3313	13.25	344.3488	21.5
344.3688	15.25	344.38	17.5
344.3988	11.25	344.41	21
344.43	11.25	344.4525	23.5
344.4613	19.5	344.4838	18.75
344.4963	15.5	344.5163	20.5
344.5313	10.75	344.55	23.25
344.5663	11.75	344.5825	26
344.5988	12.5	344.6138	20
344.6325	14	344.6475	22.5
344.6625	12.5	344.6838	26.25
344.6963	12.5	344.7188	22
344.7288	9.5	344.75	17.5
344.7638	11	344.785	20.5
344.8	11.5	344.8175	20
344.8313	13.75	344.8488	20.5
344.8688	11.25	344.88	19
344.8988	13.25	344.91	22
344.93	12.5	344.9525	19.5
344.9613	11.75	344.9838	23
344.9963	12.25	345.0163	20.5
345.0313	15.75	345.0475	20.25

345.0663	11.25	345.08	20.25
345.0988	10.25	345.1138	18.5
345.1325	10.75	345.1475	21
345.1625	10	345.1838	19.5
345.1963	15	345.2188	21.25
345.2288	11.5	345.25	19.5
345.2638	12	345.2863	17.5
345.3	12	345.3175	19.25
345.3313	10.75	345.3488	17.5
345.3688	13	345.38	18.5
345.3988	12.5	345.41	21.5
345.43	14.5	345.4525	17.25
345.4613	9	345.4838	18.5
345.4963	12.75	345.5163	19
345.5338	12	345.5475	17.25
345.5663	18	345.58	17.75
345.5988	16.5	345.6138	20
345.6325	13.5	345.6475	21.25
345.6625	10.75	345.6838	17.5
345.6963	15.25	345.7188	21
345.7288	15.5	345.75	21.25
345.7638	13.25	345.785	18.25
345.7988	13.25	345.8175	18.25
345.8313	15	345.8488	18.5
345.8688	19.25	345.88	17.75
345.8988	16.5	345.9125	14.25
345.93	17.25	345.9525	20
345.9613	16	345.9838	13.25
345.9963	12.25	346.0163	17.75
346.0338	12.75	346.0475	16.75
346.0663	11.75	346.08	15
346.0988	12	346.1138	15.25
346.1325	11.25	346.1475	15.5
346.1625	11	346.1838	16.75
346.1963	8.25	346.2188	16.25
346.2288	10.5	346.25	18
346.2663	8.5	346.285	14.5
346.2988	9.75	346.3175	17
346.3313	12.25	346.3488	15.75
346.3688	12	346.38	17
346.3988	11.25	346.4125	18.5
346.43	11	346.4525	16.75
346.4613	12.75	346.4838	19.5
346.4963	11.75	346.5138	18.25
346.5338	13.25	346.5488	18
346.5663	14	346.58	19.25
346.5988	16.25	346.6138	19.5
346.6325	10.75	346.6475	15.25
346.6625	9.25	346.6838	15.5
346.6963	13	346.7188	12.5

346.73	14.75	346.75	21.5
346.7638	14	346.785	23.75
346.7988	11.25	346.8175	15.75
346.8338	14	346.85	19.75
346.8688	11.5	346.88	20.25
346.8988	11.75	346.9125	18.5
346.9313	12.25	346.95	20
346.9613	11.25	346.985	15.5
346.9975	10.5	347.0138	16.5
347.0338	12	347.0488	18
347.0663	11	347.0825	18
347.0988	13.75	347.1138	17
347.1325	13.25	347.1475	18.5
347.1625	11.75	347.1838	17.5
347.1963	10.75	347.2188	21
347.23	11.25	347.25	16.25
347.2638	13.25	347.285	15.5
347.2988	9.25	347.3175	14.75
347.3338	9	347.3488	16
347.3688	11.25	347.38	16.5
347.3988	10.75	347.4125	15.25
347.43	10.75	347.45	14
347.4613	11.25	347.4838	18.75
347.4963	11.25	347.5138	18.25
347.5338	11.75	347.5488	16
347.5663	14	347.58	18
347.6013	16.25	347.6138	22.5
347.6325	14	347.6475	22
347.6625	17.5	347.685	23.25
347.6963	16.5	347.7188	20.75
347.73	19.25	347.75	19.75
347.7638	15	347.785	15.75
347.7988	16.5	347.8175	20
347.8338	18.5	347.8488	18
347.8688	20	347.88	16.75
347.8988	14.5	347.9125	19.75
347.93	16.25	347.95	17.25
347.9613	15	347.9838	18.75
347.9963	14.75	348.0138	19.25
348.0338	12.5	348.0488	20
348.0663	13.5	348.08	21.75
348.1013	11.25	348.1138	17.25
348.1325	13.25	348.1475	17.75
348.1625	9.75	348.185	24
348.1963	9.5	348.2188	21
348.23	12.25	348.25	20.75
348.2638	14	348.285	19
348.2988	12.25	348.3175	15.5
348.3338	13.5	348.3513	18
348.3688	12.5	348.38	18.25

348.3988	14.75	348.415	21.75
348.43	12.25	348.4525	17.5
348.4613	15.25	348.4838	14.75
348.4963	12.5	348.5138	17
348.5338	12.5	348.5488	15.75
348.5638	16	348.58	16
348.6013	11.25	348.6138	19.5
348.6325	16.75	348.6475	17
348.6625	15.25	348.6838	14
348.6963	15.75	348.7188	16
348.73	13.75	348.75	12.25
348.7638	14.75	348.7825	14.5
348.7988	13.75	348.8175	17.25
348.8338	11.5	348.8488	14
348.8688	9.25	348.88	17.5
348.8988	16.5	348.915	16.75
348.93	13.5	348.95	17.75
348.9613	11.5	348.9838	17.75
348.9963	13.5	349.0138	18.75
349.0338	13.75	349.0488	15.25
349.065	11.25	349.08	15
349.1013	15.5	349.1138	19
349.135	10.75	349.1475	21.75
349.1625	12	349.1838	16.5
349.1963	15.25	349.2188	16
349.23	14.75	349.25	14.5
349.2638	14.75	349.2825	17.75
349.2988	14.75	349.3175	18.5
349.335	13.25	349.3488	16
349.3713	13	349.38	18
349.3988	15.5	349.415	17.25
349.43	11.25	349.45	20.5
349.4613	11.75	349.4838	18.75
349.4963	15.5	349.5138	20.5
349.5338	9.25	349.5488	20
349.565	12.25	349.58	17.75
349.6013	13.5	349.6138	21.75
349.6325	14.75	349.6488	20.75
349.6625	13.25	349.6838	22.5
349.6963	14	349.7188	24.25
349.73	16.25	349.75	23.5
349.7638	17.75	349.7825	24
349.7988	15.25	349.8175	24.5
349.8338	12	349.8488	23.25
349.8713	14.5	349.88	22.5
349.8988	12.75	349.915	18.25
349.93	12.5	349.95	25
349.9613	10.5	349.9838	18.25
349.9963	10.75	350.0138	25.5
350.0338	11.75	350.0488	22.75

350.065	14.25	350.08	25.75
350.1013	13.25	350.1138	22.5
350.1325	11	350.1488	26
350.1625	11.5	350.1838	22.5
350.1963	11.5	350.2188	27.25
350.23	13.5	350.25	17.25
350.2638	11.75	350.2825	21.5
350.2988	11.25	350.3175	21.25
350.3338	14.75	350.3488	17
350.3688	14.75	350.38	24
350.3988	14	350.415	23.75
350.43	12.75	350.45	22.25
350.465	13	350.4838	21.25
350.4963	14.75	350.5138	25.5
350.5338	14.25	350.5488	26.75
350.565	15	350.58	20.25
350.6013	11.75	350.6138	19.5
350.6325	11.25	350.6488	18.5
350.6625	11.25	350.6838	19.5
350.6963	10.25	350.7188	22.5
350.73	12.5	350.75	25.25
350.7638	10	350.7825	20.25
350.7988	14.5	350.8175	19.75
350.8338	8	350.8488	21
350.8688	11	350.88	24
350.8988	8.75	350.915	23
350.93	12.5	350.95	21
350.9638	11.5	350.9838	19.75
350.9963	12	351.0138	15.5
351.0338	13.5	351.0488	24.5
351.065	12.5	351.08	20
351.1013	8.75	351.1138	19
351.1325	9.75	351.1488	20.25
351.1625	8.75	351.1838	22.75
351.1938	10.5	351.2188	21.25
351.23	9.75	351.25	17.25
351.2625	10.25	351.2825	21.25
351.2988	13.25	351.3175	21.25
351.3313	10.25	351.3488	20.75
351.3688	13.75	351.38	18.25
351.3988	14	351.4175	20.25
351.4313	15.75	351.45	23
351.465	14	351.4838	18.75
351.4988	10.5	351.5138	13.75
351.5363	14.25	351.5488	16
351.5675	11.5	351.58	15.25
351.6038	12	351.6138	20.5
351.635	12	351.6488	17.75
351.665	13.5	351.6838	16
351.6963	8.75	351.7175	21

351.7325	6	351.75	19.25
351.7638	13.75	351.785	19.75
351.8025	10	351.8175	17
351.835	11	351.8488	17.5
351.87	12.5	351.88	18
351.9	13	351.9175	18.5
351.9313	10.75	351.95	14.5
351.965	13.5	351.9838	15.75
351.9988	11.75	352.0138	17.75
352.0363	9	352.0475	16.5
352.0675	6.5	352.08	20.5
352.1038	11.75	352.1138	19.5
352.135	8	352.1488	21.5
352.165	13.25	352.1863	20
352.1963	12.75	352.2175	23.75
352.2325	11	352.25	22.5
352.2638	11.5	352.285	24.25
352.3025	11	352.3175	18
352.335	11.5	352.3488	23.5
352.37	11.5	352.38	18.25
352.4	10.75	352.4175	17.75
352.4313	16.75	352.45	20.5
352.465	14.25	352.4838	21.75
352.4988	10.25	352.5138	19
352.5363	15.5	352.5475	18.25
352.5675	11.75	352.58	18.25
352.6038	16	352.6138	17
352.635	8.25	352.6488	18.75
352.665	12	352.6838	21
352.6963	7.5	352.7175	18.75
352.7325	12.5	352.75	23.25
352.7638	9.25	352.785	17.5
352.8025	10	352.8175	22.75
352.835	10.5	352.85	18.75
352.87	10.5	352.88	22.5
352.9	12.25	352.9175	20.25
352.9338	13.75	352.95	19.75
352.965	14.5	352.9838	25
352.9988	16	353.0138	20.5
353.0363	15.5	353.0475	20.75
353.0675	15	353.0825	22.75
353.1025	11.25	353.1138	18
353.135	12.25	353.1488	26
353.165	10.5	353.1838	21.25
353.1963	12.5	353.2175	19.75
353.2325	11.75	353.25	20
353.2638	10.25	353.285	23.25
353.3025	10.75	353.3175	20
353.335	12	353.3488	20
353.37	9.25	353.38	24.5

353.4	13	353.4175	24
353.4313	10.75	353.4475	22.25
353.465	9.75	353.4838	24.5
353.4988	18.75	353.5138	24.5
353.5338	12.5	353.5475	23.5
353.5675	12.75	353.58	23.5
353.6025	15.25	353.6138	24.5
353.635	12.5	353.6488	22.75
353.6663	11.25	353.6838	22.25
353.6963	11.5	353.7175	22.5
353.7325	15.5	353.75	27.25
353.7638	13.5	353.785	28.5
353.8025	12	353.8175	23.75
353.835	15.75	353.8488	20
353.87	16.5	353.88	21
353.9	12.75	353.9175	24
353.9313	13.75	353.9475	24.25
353.965	12.25	353.9838	20.5
353.9988	14.5	354.0138	21.5
354.0338	11.5	354.0475	22
354.0675	12.5	354.08	19
354.1025	15	354.1138	23.5
354.135	11.5	354.1488	23.75
354.165	13	354.1838	24
354.1963	18.75	354.2175	22
354.2325	18	354.25	22.75
354.2638	17.25	354.285	20.5
354.3025	13.25	354.3175	21.5
354.335	16.5	354.3488	16.5
354.37	12.75	354.38	27.5
354.4	14.75	354.4175	19
354.4313	13.25	354.4475	22
354.465	15	354.4838	20.25
354.4988	13.25	354.5138	21.75
354.5325	13.75	354.5475	26.25
354.5675	14	354.58	21.5
354.6025	16	354.6138	22
354.635	10.5	354.6488	20.5
354.665	12	354.6838	19.25
354.6963	11.5	354.7175	20.25
354.7325	13	354.75	20.25
354.7638	11.5	354.785	18
354.8025	15.5	354.8175	19.25
354.835	14.25	354.85	15.75
354.87	9.5	354.88	19.5
354.9	10.25	354.9175	16.75
354.9313	10.75	354.9475	17.75
354.965	10.75	354.9838	19.25
354.9988	10.75	355.0138	21.5
355.0325	9.25	355.05	15.5

355.0675	11.75	355.08	16.5
355.1025	9.5	355.1138	22.75
355.135	12.75	355.1488	19.75
355.165	10.25	355.1838	20.25
355.1963	9.25	355.2175	18
355.2325	10.5	355.25	17
355.2638	11.75	355.285	19.75
355.3025	9.5	355.3175	22.5
355.335	10.25	355.3488	17
355.3675	7.5	355.38	18.5
355.4	13	355.4175	23.25
355.4313	9.5	355.4475	22
355.465	11.25	355.4838	16
355.4988	11.75	355.5138	17.25
355.5325	11	355.5475	20.25
355.5675	11	355.58	19
355.6025	12.5	355.6138	20.25
355.635	11.25	355.6488	14.75
355.665	13	355.6838	20.5
355.6963	10.75	355.7175	18.75
355.7325	11.25	355.75	23.5
355.7638	12.5	355.785	20.75
355.8025	14.25	355.815	15.25
355.835	10.75	355.8488	22.25
355.8675	15.25	355.88	18.25
355.9	15.5	355.9175	20.5
355.9313	11	355.9475	22
355.965	14.5	355.9838	20
355.9988	12.75	356.015	18.5
356.0325	13	356.0488	21.75
356.0675	13.25	356.085	18.75
356.1025	13	356.115	23.25
356.135	13.5	356.15	20
356.165	12.25	356.1875	24.5
356.1963	13.25	356.22	20.25
356.2325	14.75	356.2525	19.5
356.2638	14	356.2875	15.5
356.3025	13.25	356.3175	16.5
356.335	12.5	356.3525	18
356.3675	12.5	356.385	20.25
356.4	13.75	356.42	15.5
356.4313	12.75	356.45	15.5
356.4638	13.5	356.485	16.25
356.4988	14	356.515	12.75
356.5325	9.75	356.5488	17
356.5675	12.75	356.5825	21.5
356.6025	10.5	356.615	19.75
356.635	9.75	356.6513	19.25
356.665	9.75	356.685	20.75
356.6963	9.75	356.72	22

356.7325	11	356.7525	26
356.7638	10.25	356.7875	17
356.8025	11.5	356.8175	21.5
356.835	9.5	356.8525	21.75
356.8675	11.5	356.8875	18.25
356.9	13.25	356.92	22.75
356.9313	12.5	356.95	20.5
356.9638	11.25	356.985	23.5
356.9988	13.5	357.015	17.75
357.0325	8.75	357.0488	21.5
357.0675	10.5	357.0825	17.5
357.1025	13.75	357.115	20.75
357.1338	13	357.1525	21.25
357.165	14.75	357.185	22.25
357.1963	13	357.22	22.25
357.2325	14	357.2525	19
357.2638	16.75	357.2875	22
357.3025	15.5	357.3175	20.25
357.335	16	357.3525	19
357.3675	15	357.385	20.75
357.4	15.5	357.42	20.5
357.4313	20	357.45	19
357.4638	17.25	357.485	23.5
357.4988	16	357.515	19.25
357.5325	11.75	357.5488	19.5
357.5675	14.5	357.5813	17.5
357.6025	14.5	357.615	20.25
357.6338	17.25	357.6525	19.5
357.665	15	357.685	18.25
357.6963	13.25	357.72	13.25
357.7325	12	357.7525	19.25
357.7638	14.75	357.7875	25.5
357.8	13	357.8175	18.75
357.835	14.5	357.8525	13.75
357.8675	14	357.885	15.25
357.9	14.75	357.92	15.25
357.9313	17	357.95	18.5
357.9638	13	357.985	17
358.0013	15	358.015	18.25
358.0325	18.5	358.0488	17
358.0675	12.5	358.0813	22.25
358.1025	12.75	358.115	22.75
358.1338	12.25	358.1525	18.5
358.165	13.25	358.185	16.75
358.1963	14.5	358.22	15.75
358.2325	13.25	358.2525	18
358.2638	13.75	358.2875	15.5
358.3	11.75	358.3175	20.25
358.335	14.75	358.3525	23.25
358.3675	10.75	358.385	19.25

358.4	9.25	358.42	19
358.4313	8.5	358.45	21.25
358.4638	11.75	358.485	17.5
358.5013	11	358.515	21
358.5325	10.75	358.5488	19.25
358.5675	11	358.5813	21.75
358.6025	11.25	358.615	18.25
358.6338	10.25	358.6525	19.25
358.665	13	358.685	20.25
358.6963	11.25	358.72	15
358.7325	14.25	358.7525	22.25
358.7638	10.75	358.7875	25.25
358.8	11.25	358.8175	22.75
358.835	10	358.85	19
358.8675	13.5	358.885	18.25
358.9	15.75	358.92	20
358.9313	15.75	358.95	18.5
358.9638	12.5	358.985	18
359.0025	15.5	359.015	20.25
359.0325	14.25	359.0488	17
359.0675	19.75	359.0813	16.5
359.1025	11.25	359.115	21.25
359.1338	13.75	359.1525	17.5
359.165	13.5	359.185	19.5
359.1963	14	359.22	17.5
359.2325	12.5	359.2525	17.75
359.2638	13.25	359.2875	17
359.3	9	359.3175	17.75
359.335	13	359.35	18.75
359.3675	12.75	359.385	18.75
359.3975	10	359.42	18
359.4313	11.5	359.45	19
359.4638	11.25	359.485	20.5
359.5013	12.5	359.515	19.25
359.5325	12.25	359.5488	22.25
359.5675	11.25	359.5813	17.25
359.6025	12.75	359.615	19.5
359.6338	11.5	359.6525	19.5
359.665	14.5	359.685	19
359.6963	13.5	359.72	17.5
359.7325	11.75	359.7525	18.5
359.7638	11.5	359.785	14.5
359.8	13	359.8175	19.25
359.835	11.25	359.85	17.25
359.8675	9	359.885	17
359.8975	12.5	359.92	15.5
359.9313	14.75	359.95	19.5
359.9638	17	359.985	15.25
360.0013	13	360.015	15
360.0325	16.5	360.0488	14

360.0675	12.75	360.0813	17.75
360.1025	14.5	360.1138	18
360.1338	14.75	360.1525	19.75
360.165	15.5	360.185	14.75
360.1963	14	360.22	18.75
360.2325	12.75	360.2538	16.75
360.2638	16.25	360.285	17.75
360.3	16.75	360.3188	16.25
360.335	14.25	360.35	21.25
360.3675	14.25	360.385	19.5
360.3975	10.5	360.4225	17.75
360.4313	12	360.45	17.5
360.4663	14	360.485	18.25
360.5013	9	360.515	16
360.5325	15	360.5488	15.25
360.5663	8.75	360.5813	14.75
360.6025	12.25	360.6138	21.5
360.6338	14.25	360.6525	17.25
360.665	14.5	360.685	20
360.6963	11.25	360.72	16.75
360.7325	11.25	360.7525	17
360.7638	9.5	360.785	16
360.7988	10.75	360.8175	20.75
360.835	13.75	360.85	17.25
360.8675	13.25	360.885	18
360.8975	13	360.92	20.75
360.9313	13.75	360.95	19.5
360.9638	13.25	360.985	17
361.0013	13.25	361.015	14.75
361.0325	10.5	361.0488	15.5
361.0663	9.5	361.0813	19
361.1025	12	361.1138	15.25
361.1338	11.75	361.1525	15.25
361.1638	9.5	361.185	19.5
361.1975	10	361.22	20.5
361.2325	9.5	361.2525	20.25
361.2638	11.5	361.285	21
361.2988	7.25	361.3175	17.75
361.335	10.25	361.3525	17
361.3675	12.25	361.3825	17.75
361.3975	10.25	361.42	20.25
361.4313	11	361.45	19
361.4638	9.25	361.485	20.75
361.5013	8.75	361.515	19.5
361.5325	10	361.5488	17.5
361.5663	11.25	361.5825	23
361.6025	8.5	361.615	19.75
361.6363	7.75	361.6525	22.5
361.6663	9.5	361.685	18
361.6988	10	361.72	18.5

361.735	9.75	361.7525	19.75
361.7663	11.25	361.785	17.5
361.8013	11.5	361.8175	21.5
361.8375	8	361.85	16.75
361.8688	11.75	361.8825	19.75
361.8988	9	361.92	16
361.9325	8.25	361.95	20
361.965	11.25	361.9825	15.5
362.0025	12.75	362.015	18.75
362.0338	11.5	362.0488	17
362.0675	8.75	362.0825	17.25
362.105	14.75	362.1138	15
362.1363	12.75	362.1513	16
362.1663	13	362.185	19.25
362.2	11.25	362.22	19.75
362.235	10.5	362.2525	14.25
362.2663	11.75	362.285	16.25
362.3013	12.75	362.32	18.75
362.3375	9.5	362.35	15.75
362.3688	11.25	362.385	18.25
362.3988	9	362.42	19.25
362.4325	13	362.45	19
362.465	10.75	362.4825	20
362.5025	10.25	362.515	22
362.5338	11.25	362.5488	16
362.5675	9	362.5825	20.75
362.605	12.5	362.6138	18.5
362.6363	12	362.6513	23.5
362.6663	10.75	362.685	21.5
362.7	11.75	362.72	19.75
362.735	10.75	362.7525	19.75
362.7663	9.75	362.785	22.75
362.8013	15.25	362.8175	17.25
362.8375	9.75	362.8513	22
362.8688	16.25	362.8863	22.5
362.8988	13.75	362.9225	22
362.93	11.5	362.9525	16.25
362.965	14.25	362.985	16
363.0025	11.25	363.0175	17
363.0338	11.25	363.0513	18
363.0675	11.75	363.085	17.75
363.105	9.5	363.1163	19.5
363.1363	11.25	363.1538	12.25
363.1663	9.5	363.1888	15.75
363.2	10.75	363.2225	17.5
363.235	12	363.2538	17.5
363.2663	9	363.2888	17.75
363.3025	11.25	363.3188	19
363.3375	13.25	363.3513	18.25
363.3688	10.25	363.3888	18

363.3988	10.75	363.42	17.5
363.4325	15	363.4525	22.25
363.465	11.75	363.485	15.5
363.5025	12.5	363.5175	12.5
363.5338	14	363.5513	17
363.565	10.75	363.585	21.25
363.605	14.5	363.6163	18.75
363.6363	14.25	363.6538	19
363.6663	15.25	363.6863	16.5
363.7	11	363.7213	13.75
363.735	11.5	363.7538	15
363.7663	9.5	363.7888	16.5
363.8013	8.75	363.8188	15.75
363.835	10.5	363.8513	18.25
363.8688	12.75	363.8888	17
363.8988	8.25	363.92	18.75
363.93	11.5	363.955	15.75
363.965	16.5	363.985	17
364.0025	14.75	364.0175	14.75
364.0338	10.25	364.0513	15
364.065	12.5	364.085	17.75
364.105	12.25	364.1163	15
364.1363	14.5	364.1538	18
364.1663	13.5	364.1838	18.25
364.2	9.5	364.2213	14.25
364.235	13.5	364.2538	14.75
364.2663	12.75	364.2888	15
364.3013	11.5	364.3213	17.5
364.335	13.25	364.3513	18.5
364.3688	15	364.3888	14
364.3988	15.25	364.42	14.75
364.43	12.75	364.4525	14
364.465	17.5	364.485	14
364.5025	16.5	364.5175	14.25
364.5338	16.5	364.5513	16.25
364.565	19.75	364.585	13.75
364.605	14.5	364.6163	14
364.6363	16.75	364.6538	15.25
364.6663	17.75	364.6838	14
364.7013	17	364.7213	15.25
364.735	15	364.7538	17.5
364.7663	18.5	364.7888	14.5
364.8013	16.5	364.8238	11.75
364.835	21.75	364.8513	11.5
364.8688	22	364.8888	14
364.8988	19.25	364.92	12.5
364.93	15.5	364.9513	12.25
364.965	18.25	364.985	11.75
365.0025	13.5	365.0175	17
365.0338	14.25	365.0513	12.25

365.0663	17.75	365.085	10.75
365.105	15.25	365.1163	10
365.1363	12.25	365.1538	15.25
365.1663	13	365.1838	11
365.1988	20	365.2213	10
365.235	14.5	365.2538	14
365.2663	16.75	365.2888	10
365.3013	14.25	365.3213	12.5
365.335	19	365.3513	9.75
365.3688	18	365.3888	14.25
365.3988	17.5	365.42	11.5
365.43	15	365.4538	13.25
365.465	17.25	365.485	12.25
365.5025	17.25	365.5175	13.25
365.535	16.25	365.5513	14.25
365.565	16	365.5875	12.75
365.605	17.75	365.6163	11.5
365.6363	15.75	365.6538	16
365.6663	13.5	365.6838	13.5
365.6988	14.5	365.7213	17.5
365.735	16	365.7538	16.25
365.7663	12.75	365.7888	13
365.8013	10.25	365.8213	16.25
365.8338	10.75	365.8513	15.25
365.8688	11.75	365.8888	12.75
365.8988	9.75	365.92	17.75
365.93	8.5	365.9538	13.75
365.965	11	365.985	13.25
366.0025	11.75	366.0175	15.75
366.035	10.5	366.0513	16.5
366.065	13.75	366.085	16.5
366.105	9.75	366.1163	15.75
366.1363	14	366.1538	12.25
366.1663	7.75	366.1838	11
366.1988	12	366.2188	15
366.2325	17.75	366.2538	15.5
366.2663	12.75	366.2888	15.5
366.3013	12	366.3213	13.5
366.3363	15.5	366.3513	11.25
366.3688	16	366.3888	10.75
366.3988	11.75	366.42	14
366.43	11.5	366.4538	10.25
366.465	14.5	366.485	13.5
366.5025	10.25	366.5175	16
366.535	9.75	366.5513	14
366.565	8.5	366.585	13
366.605	12	366.62	15.25
366.6363	7.5	366.6538	17
366.6663	14.25	366.6838	14.5
366.6988	14.25	366.7188	17

366.7325	11.75	366.7538	13.25
366.7663	8.5	366.7888	16.5
366.8013	17	366.8213	14.75
366.8338	14.25	366.8513	16.75
366.87	13	366.8888	15.25
366.9013	13.5	366.92	18.5
366.93	15.5	366.9538	21.5
366.965	12.75	366.9838	15.5
367.0025	10.75	367.0175	17.25
367.035	12.25	367.0538	18.5
367.065	14.75	367.085	21.25
367.105	15.5	367.1188	17.75
367.1363	12.5	367.1538	20.25
367.1663	11.75	367.1838	18.5
367.1988	13.5	367.2188	14
367.2325	14.25	367.2525	16.5
367.2663	15.5	367.2888	17.25
367.3013	14.25	367.3213	15.75
367.3338	16	367.3513	21
367.3688	16.5	367.3888	14.75
367.3988	15.75	367.42	18.5
367.43	13.5	367.4538	16
367.465	16.75	367.4863	14.5
367.5025	17.75	367.5175	18.5
367.535	14.5	367.5538	16.25
367.565	19	367.585	15
367.6025	21.75	367.6188	16.75
367.6363	15.25	367.6538	16
367.6663	17.5	367.6838	18.5
367.6988	15.25	367.7188	16.75
367.7325	16.75	367.75	19
367.7663	15.5	367.7888	19.5
367.8013	17	367.8213	14.5
367.8338	13	367.8513	16.75
367.8688	15.75	367.8888	15.75
367.8988	16.5	367.92	17.25
367.93	13.25	367.9538	16.25
367.9625	15.25	367.985	15.75
368.0025	16.75	368.0175	16
368.035	13.75	368.0538	14.75
368.065	16.75	368.085	15.25
368.1025	14.25	368.1188	17.5
368.1363	11.5	368.1538	21.5
368.1663	13.5	368.1838	17.75
368.1988	11.5	368.2188	18
368.2325	14.75	368.25	17.25
368.2663	13.5	368.2888	17.5
368.3013	12.25	368.3213	16
368.3338	11.25	368.3513	14.5
368.3688	14.75	368.3888	15.5

368.3988	14	368.42	18.25
368.43	15.5	368.4538	17
368.4625	13.5	368.4838	18.5
368.5025	16	368.5175	17
368.535	11.75	368.5563	18.5
368.565	16	368.585	17
368.6025	12.25	368.6188	17.75
368.6363	13.25	368.6538	17.75
368.6688	16.5	368.6838	17.25
368.7013	11.5	368.7188	19.25
368.7325	10.75	368.75	17
368.7663	14.25	368.7875	20.5
368.8013	15.25	368.8213	18.25
368.8338	11.5	368.8513	19.75
368.8688	11.25	368.8888	19.5
368.8988	10.25	368.92	11.75
368.93	9.5	368.9538	16
368.9625	16	368.9838	16
369.0025	10.5	369.0163	18.75
369.035	12	369.0538	15.75
369.065	15	369.085	16
369.1025	14.75	369.1188	13.75
369.1375	11.75	369.1538	17.75
369.1663	16	369.185	18
369.1988	11.5	369.2188	16.5
369.2325	11.75	369.25	17.25
369.2663	14.25	369.2875	15.5
369.3013	13	369.3225	19.5
369.3313	13.75	369.3513	18.25
369.3675	13.25	369.3888	18.25
369.3988	11	369.42	17.25
369.43	13.75	369.4538	19.5
369.4625	11.75	369.4838	16.25
369.5013	9.75	369.5163	17.25
369.535	10.25	369.5538	16.75
369.565	13	369.585	14.25
369.6025	14.75	369.6188	14.75
369.6363	13.25	369.6538	13.25
369.6663	13.75	369.685	15.5
369.6988	14.75	369.7188	16
369.7325	12.75	369.75	18.75
369.7663	13.25	369.79	19.5
369.8013	15.75	369.8213	17.25
369.8313	14.5	369.8513	16.75
369.8675	17	369.8888	18.5
369.8988	11.5	369.92	21.5
369.93	14.25	369.9538	18
369.9625	13.5	369.9838	16.25
370.0013	15	370.0163	14.75
370.035	12.5	370.0538	19.75

370.065	13.25	370.085	16.5
370.1025	11.75	370.1188	17
370.1363	12.25	370.1538	21
370.1663	12	370.185	14.75
370.1988	14.75	370.2188	19.5
370.2325	13.5	370.25	16.25
370.2663	10	370.2863	18.75
370.3013	14.75	370.3213	17.25
370.3313	12.75	370.3513	13.75
370.3675	12.25	370.3888	16
370.3988	13.75	370.42	17
370.43	11.5	370.4538	16.5
370.4625	14	370.4838	14
370.5013	10.25	370.5138	13.5
370.535	12.25	370.5538	15.75
370.565	12.75	370.585	13.75
370.6025	8.25	370.6188	12.25
370.6363	10	370.6538	14.25
370.6663	12.25	370.685	15
370.6988	10.75	370.7188	14
370.7325	11.5	370.75	15.5
370.7663	10.75	370.7863	14
370.8013	11.5	370.8213	14
370.8313	10	370.8513	15.5
370.8675	15	370.8888	14.25
370.8988	12.5	370.92	16.75
370.93	15.25	370.9538	14
370.9625	14.25	370.9838	13.25
371.0013	11.5	371.0138	15.5
371.035	16.25	371.0538	17
371.065	15	371.085	12.25
371.1025	14	371.1188	12.25
371.1338	11.75	371.1538	10.25
371.1663	13.25	371.185	11
371.1988	12	371.2188	10.75
371.2325	9.5	371.25	9.75
371.2663	11.75	371.2863	10.5
371.3013	11.5	371.3213	9.25
371.3313	12.25	371.3513	11.25
371.3675	12.75	371.3888	11
371.3988	8	371.42	12
371.4325	8.5	371.4538	16.5
371.4625	9.25	371.4838	11
371.5013	6.5	371.5138	14.75
371.535	9.5	371.5538	13.25
371.565	10	371.585	12.25
371.6025	11.75	371.6188	12.25
371.6338	11.25	371.6538	15.25
371.6663	11	371.685	13.75
371.6988	10.5	371.7188	17.5

371.7325	11.75	371.7513	12.75
371.7663	11.75	371.7863	14.25
371.8013	9.5	371.8213	16.75
371.8338	7.75	371.8513	10.25
371.8675	9.5	371.8888	15
371.8988	11.25	371.92	16.25
371.9325	10.25	371.9538	14.75
371.9625	11.25	371.9838	15.25
372.0013	12.5	372.0163	16.5
372.035	14	372.0513	11.5
372.065	10	372.085	11.75
372.1025	9	372.1188	13
372.135	12	372.1538	19
372.1663	11.5	372.185	15.25
372.1988	10.75	372.2188	18.75
372.2325	11.5	372.25	18
372.2638	10.5	372.2863	15
372.3013	10.75	372.3213	21
372.3338	13.5	372.3513	16
372.3675	12.5	372.3888	15.75
372.3975	8	372.4225	17.25
372.4325	9.25	372.4538	15.5
372.465	8.5	372.4838	19
372.5013	8	372.5163	16.5
372.535	8	372.5513	13.5
372.565	10	372.585	17.25
372.6038	12	372.6188	14.75
372.635	7.75	372.6538	13.25
372.6675	7.75	372.685	14.5
372.7	9.5	372.7188	15.75
372.7338	7	372.75	10.75
372.765	8	372.7863	13.5
372.8025	7.75	372.8213	13.25
372.8363	9.25	372.8513	15.25
372.87	7	372.8888	13
372.9	6.5	372.9225	12.5
372.935	8.75	372.9538	11
372.9675	8.75	372.9838	11.25
373.0038	10.75	373.0163	19.5
373.0375	8.75	373.0513	15.25
373.0675	9.5	373.085	15.75
373.1038	6.5	373.1188	15.75
373.135	12	373.1538	15.25
373.1675	9.25	373.185	14
373.2	11.5	373.2188	17.25
373.2338	11.75	373.25	17
373.265	13	373.2863	18
373.3025	12.5	373.3213	19.25
373.3363	9.75	373.3513	17.5
373.37	11.5	373.3888	18

373.4	9.25	373.4225	16.25
373.435	11.75	373.4538	17
373.4675	9.75	373.4838	19
373.5038	13.75	373.5163	18
373.5375	11.5	373.5513	16.75
373.5675	12	373.585	18.5
373.6038	10.75	373.6188	20.75
373.635	11.5	373.6538	19
373.665	13	373.685	17.75
373.7	13.25	373.7188	15.25
373.7338	10.5	373.75	16
373.765	10	373.7863	15.5
373.8025	14.75	373.8213	17.75
373.8363	10.5	373.8513	13.75
373.87	9.75	373.8888	18.5
373.9	9.75	373.9225	15.5
373.935	10.5	373.9538	15.75
373.9675	8.25	373.9838	17.25
374.0038	8	374.0163	17.75
374.0375	8.75	374.0513	18.75
374.0675	9.25	374.085	17
374.1038	10.5	374.1188	14.5
374.135	9.5	374.1538	13
374.165	6.75	374.185	13
374.2	6.25	374.2188	15
374.2338	7.75	374.25	13
374.27	7.75	374.2863	14
374.3025	13.25	374.32	13.5
374.3363	9.5	374.3513	13.75
374.37	9	374.3888	14.75
374.4	10	374.4225	13.75
374.435	8.5	374.4538	18
374.4675	8.5	374.4838	14.25
374.5038	9.25	374.5163	13.75
374.5363	9.5	374.5513	16.25
374.5688	8	374.585	11
374.6038	11	374.6188	13.75
374.635	8.75	374.6538	11.5
374.665	7.5	374.685	9.75
374.7	9.5	374.7188	13.5
374.7338	11.25	374.75	9.75
374.765	10.25	374.7838	14.25
374.8025	8.5	374.82	11.25
374.8363	7.75	374.8538	14.25
374.87	7.75	374.8888	11.75
374.9	10	374.9225	14.75
374.9325	10	374.9538	14.5
374.965	10	374.9838	18
375.005	8.5	375.0163	12.5
375.0363	7.75	375.0513	13.25

375.0688	9.75	375.085	11.75
375.1013	6.75	375.1213	15.5
375.135	13	375.1538	14.5
375.165	9.25	375.185	14
375.2	9.25	375.2188	17.75
375.2338	8.5	375.2513	16.5
375.265	10.75	375.2838	16
375.3025	8.25	375.32	14
375.3363	9.25	375.3525	14.5
375.37	7.75	375.3888	18.25
375.4	9.75	375.4225	15.75
375.4325	8.25	375.4538	17
375.4675	8.5	375.4838	16
375.5038	8.25	375.5163	16
375.5363	8.25	375.5488	18.25
375.5688	6.75	375.585	11.5
375.6013	7.75	375.6213	15.5
375.635	5.75	375.6538	19
375.665	8.5	375.685	11.75
375.7	9	375.7188	15.5
375.735	6.75	375.7513	13.75
375.765	10	375.7838	13
375.8025	7.5	375.82	17.75
375.8363	6.5	375.8525	18
375.87	5.5	375.8888	16.25
375.9	6	375.9225	13
375.9325	6.5	375.9538	20
375.965	7.75	375.9838	15
376.0038	8.25	376.0188	16.25
376.0363	5.75	376.0488	11.75
376.0713	6.5	376.085	13.75
376.1013	8	376.1213	15
376.135	7.75	376.155	15.75
376.1675	9.5	376.185	15.5
376.2	7	376.2188	16
376.235	9.5	376.2513	18.5
376.265	9	376.2838	19
376.3025	7.25	376.32	15
376.3363	8.75	376.3525	19.75
376.37	8.5	376.3888	17.75
376.4	10.25	376.4225	17
376.4325	10.25	376.4538	19.25
376.465	9.5	376.4838	14
376.5038	7.25	376.5163	16.5
376.5363	12.5	376.5488	20.5
376.5688	8.25	376.585	19
376.6013	11	376.6213	16.25
376.635	8.75	376.655	18.25
376.6675	9.5	376.685	16.5
376.6988	9.75	376.7188	19

376.735	10.5	376.7513	20
376.765	9.75	376.7838	19.25
376.805	8.5	376.82	14.5
376.8363	9.25	376.8525	17.5
376.87	8	376.8888	13.75
376.9	9.75	376.9225	18
376.9325	11	376.9538	16.25
376.9675	8.75	376.9838	15.5
377.0038	10.25	377.0163	14.5
377.0363	12.75	377.0488	17
377.0688	11.25	377.085	14.5
377.1013	11	377.1213	13
377.135	11	377.155	13
377.1675	9	377.185	14.75
377.1988	9	377.2188	11.25
377.235	10	377.2538	17.5
377.2675	10.25	377.285	18.75
377.3063	8.25	377.3213	16.25
377.3388	7.75	377.3538	19
377.3725	8	377.3925	15
377.4025	9.75	377.4238	13
377.435	9.75	377.455	13.5
377.47	8.5	377.485	14.25
377.505	8.25	377.5188	13.25
377.54	7	377.55	19.5
377.5713	8	377.585	14.75
377.6025	10.75	377.6238	14.25
377.6363	12.75	377.6575	12.5
377.6688	7.5	377.6875	11.5
377.7	9	377.7213	17.75
377.7375	9	377.7538	12
377.7675	10	377.785	14.25
377.8063	8.25	377.8213	16
377.8388	6	377.8538	13
377.8725	8	377.8925	11.25
377.9025	7.25	377.9238	14
377.935	9.25	377.955	17
377.9675	12.5	377.985	13
378.0063	8.5	378.0188	14
378.0375	8	378.05	13
378.0713	11.75	378.085	16.75
378.1025	12.25	378.1238	15
378.1363	9.5	378.1575	16.25
378.1688	11	378.1875	15
378.2	12	378.2213	15
378.2375	7	378.2538	11.5
378.2675	7.25	378.285	13.25
378.3063	8.5	378.3213	17.5
378.3388	8.75	378.3538	14.5
378.3725	8.75	378.3925	15.5

378.4025	9.25	378.4238	14.5
378.435	8.25	378.455	13
378.47	10.5	378.4863	16.25
378.505	9.75	378.5213	13
378.5375	9	378.555	15.75
378.5713	12.25	378.5863	17.25
378.6025	13	378.625	17.75
378.6363	9.75	378.66	13.25
378.6688	10	378.69	14.75
378.7	9.75	378.7238	18.25
378.74	12.25	378.7563	19.75
378.7675	10	378.7863	19.75
378.8063	11	378.8238	20.25
378.8388	15.75	378.8563	20.75
378.8725	9.75	378.895	18
378.9038	10	378.925	16.25
378.935	11.75	378.9563	15
378.9675	11.25	378.9863	17
379.005	12	379.0213	17.5
379.0388	12	379.0538	15.5
379.0713	12.5	379.0863	17.75
379.1025	9.5	379.125	12
379.1363	12.5	379.16	13.25
379.1688	11.75	379.19	14.25
379.2	12	379.2238	14.25
379.2375	13.75	379.2563	15
379.2675	12.5	379.2863	16.75
379.3063	13.25	379.3238	15.25
379.3388	10.75	379.3588	15
379.3725	11.5	379.395	14.25
379.4038	10.75	379.425	19.5
379.435	8.25	379.4563	17.25
379.4675	10.75	379.4863	17.5
379.505	11.25	379.5213	21.25
379.5388	9.75	379.5538	20.5
379.5713	9.25	379.5863	22.5
379.6025	9	379.625	19.25
379.6363	9.75	379.66	20
379.6688	8.25	379.69	15.75
379.7	7.75	379.7238	14
379.7363	10.5	379.7563	17
379.7675	8.5	379.7863	14.75
379.8075	9.25	379.8238	17.5
379.8388	10	379.8588	14.25
379.8725	12.5	379.895	17.5
379.9038	9.25	379.925	13.75
379.935	10.5	379.9563	15
379.9675	8.5	379.9863	14.75
380.005	9.5	380.0213	14.75
380.0388	10.25	380.0525	13.25

380.0713	11	380.0863	20.25
380.1025	10	380.125	18
380.1363	9	380.16	13.75
380.1688	8.25	380.19	15.75
380.2	12.5	380.2238	17.75
380.2363	12.25	380.2563	13
380.2675	11.75	380.2863	12.75
380.3063	11.75	380.3213	18
380.3388	9.5	380.3613	19
380.3725	11	380.395	15.5
380.4038	13.25	380.425	15
380.435	10.5	380.4563	18.75
380.47	7.25	380.4863	15.25
380.505	10.25	380.5213	13.5
380.5388	10.25	380.5525	16.25
380.5713	10.25	380.5863	16.25
380.6025	9.25	380.625	16.75
380.6363	8.25	380.66	15
380.6688	10	380.69	13.5
380.7	10.5	380.7238	12.5
380.7363	11	380.7563	12.5
380.77	10.5	380.7863	13
380.8063	10	380.8213	13.75
380.8388	10.25	380.8613	18
380.8725	9.25	380.895	19
380.9038	9.25	380.925	15.5
380.935	10.5	380.9563	12
380.97	12	380.9863	13.75
381.005	14	381.0213	17.25
381.0388	10.75	381.0525	17
381.0713	8.25	381.0863	17.5
381.1025	9.5	381.125	22.75
381.1388	5.75	381.16	19
381.1688	8	381.19	17
381.2	7.5	381.2238	14.5
381.2363	9.5	381.2563	13.75
381.27	8.5	381.2863	16
381.3063	7.5	381.3213	17.5
381.3388	7	381.36	13.5
381.3713	10.75	381.395	11.75
381.4038	14.25	381.425	14.25
381.4375	9.75	381.4563	16
381.47	10	381.4863	11
381.505	13	381.5225	10.25
381.5388	9	381.5525	13
381.5713	10	381.5863	16
381.6025	11.5	381.625	11.25
381.6388	6.75	381.66	9.25
381.6688	8.25	381.69	8
381.7	8.5	381.7238	12.25

381.7363	9	381.7563	11
381.77	9.5	381.7863	8.75
381.8063	10.25	381.8213	10.25
381.8388	9.75	381.86	11.5
381.8713	8.25	381.895	10.75
381.9038	11	381.925	11.25
381.935	7.25	381.9563	8.5
381.97	10.25	381.9888	8.5
382.0025	11.25	382.0213	8
382.0388	11.5	382.0525	11.25
382.0713	11	382.0863	10.5
382.1025	12	382.125	10
382.1388	8.5	382.16	11.5
382.1688	8.5	382.19	11.5
382.2	8.25	382.2238	14.5
382.2363	9	382.2563	13.25
382.27	9.75	382.2863	10
382.305	8	382.3213	16.25
382.3388	11.75	382.36	8.5
382.3713	8	382.395	11.75
382.4038	9.5	382.425	13.5
382.435	11.25	382.4563	15.75
382.47	5.75	382.4888	11
382.5038	9.25	382.5213	11.75
382.5388	5.75	382.5525	10.25
382.5713	6	382.5875	12
382.6025	7.75	382.625	9.5
382.6388	8.5	382.66	11
382.6688	14	382.69	13
382.7	12.5	382.7238	9
382.7363	15.5	382.7563	17.75
382.77	12.75	382.7863	11.75
382.805	13.5	382.8213	15.75
382.8388	12.5	382.86	15
382.8725	12.25	382.895	14.75
382.9038	12.75	382.925	10.75
382.935	12.25	382.9563	14.75
382.97	12.75	382.9888	17
383.0038	9.25	383.0213	17
383.0388	12.75	383.0525	12.75
383.0713	12.25	383.0875	14
383.1025	12.25	383.125	15.75
383.1388	9.75	383.16	15.25
383.1688	12.5	383.19	12.75
383.2	14	383.2238	11
383.2363	12.75	383.2563	17
383.27	13	383.2863	13.25
383.305	15.75	383.3213	15.5
383.3388	17.5	383.36	18.75
383.3725	12	383.395	14

383.4038	15.5	383.425	13
383.435	12	383.4563	13.25
383.4725	15.25	383.4888	14
383.5063	12.25	383.5213	16.75
383.5413	12.25	383.5525	14.25
383.5738	11.75	383.5888	13.75
383.6038	11.25	383.625	14
383.64	12	383.66	14
383.67	10.75	383.69	13.5
383.7013	12	383.7238	17.75
383.74	11.25	383.7563	18.5
383.7713	10.75	383.7863	17.5
383.8063	13.25	383.8213	16
383.8413	9	383.86	17.25
383.875	14.5	383.895	20.25
383.9063	12.25	383.925	14
383.9375	14	383.9563	13
383.9725	12.75	383.99	16.5
384.0063	12.25	384.0213	20.25
384.0413	12.5	384.0525	19
384.0738	10	384.0888	18.25
384.1038	11.25	384.125	20.5
384.14	9.75	384.16	21.75
384.17	11.75	384.19	23.5
384.2013	13.75	384.2238	14.25
384.24	8	384.2563	17
384.2738	11.25	384.2863	15.75
384.3063	11.75	384.3188	15.25
384.3413	10	384.3575	17.5
384.375	12.5	384.395	14.75
384.4063	11.25	384.425	17.25
384.4375	12.5	384.4563	16.5
384.475	10.5	384.4888	15.75
384.5075	11.75	384.5213	16.75
384.5413	8.25	384.5525	14.5
384.5738	10.75	384.5888	16.5
384.6063	7.5	384.625	14.25
384.64	9	384.66	17.5
384.67	13.25	384.69	15.5
384.7013	9.5	384.7238	14.5
384.74	9	384.7563	19.75
384.7738	13	384.7863	13.5
384.8063	11	384.8188	13.5
384.8413	9.25	384.8575	15
384.8763	11.75	384.895	16
384.9063	11.5	384.925	15
384.9375	13	384.9563	20.5
384.975	13.25	384.9888	14.5
385.0063	12.5	385.0213	13.5
385.0413	13	385.0525	16.75

385.0738	12	385.0888	16.75
385.1063	10.25	385.125	16
385.14	12.25	385.16	18
385.17	10	385.19	17.5
385.2013	8.75	385.2238	20.25
385.24	10.5	385.2563	19.75
385.2725	10	385.2863	17.5
385.3063	9.75	385.3188	16.75
385.3413	10.5	385.3575	17.75
385.3763	9.5	385.395	15.75
385.4063	8.5	385.425	17.5
385.4375	11.5	385.4563	18.5
385.475	12.5	385.4888	15.5
385.5063	10.75	385.5213	14.75
385.5413	8	385.5525	11.5
385.5738	8.75	385.5888	17
385.6063	13.5	385.6225	15
385.64	10.75	385.66	14
385.67	10.5	385.69	16
385.7063	13	385.7238	15.5
385.74	9.25	385.7563	14.75
385.7725	8.25	385.7863	16.5
385.8063	12.25	385.8213	15.5
385.8413	12	385.8575	12.5
385.8763	10.75	385.895	10.5
385.9063	12.75	385.925	15
385.9375	11	385.9563	14
385.975	13.75	385.9888	11.25
386.0063	12	386.0213	13.5
386.0413	11	386.0525	14.5
386.0738	12	386.0888	14.75
386.1063	11.75	386.1225	16.5
386.14	13.75	386.16	15
386.17	13.25	386.19	13.25
386.2063	10.75	386.2238	16.25
386.24	11.25	386.2563	16
386.2725	12	386.2863	14.25
386.3063	11.5	386.3225	18
386.3425	11.75	386.3575	15.5
386.3763	9.5	386.395	18
386.4088	9.25	386.425	18.25
386.4375	8	386.4563	18
386.475	6.25	386.4888	19.75
386.5063	12	386.5213	18
386.5413	9	386.5525	17.5
386.5738	8.75	386.5888	18.5
386.6063	14.75	386.6213	15.75
386.64	12	386.66	18
386.67	13.25	386.69	17
386.7063	10	386.7238	17.75

386.74	10	386.7563	16.25
386.7725	8.5	386.7863	16
386.8063	9	386.8225	18
386.8425	10.75	386.8575	14.25
386.8763	9.75	386.895	13.5
386.9063	8.5	386.925	16.25
386.9375	8	386.9563	18.5
386.975	7.25	386.9888	16.75
387.0063	11.25	387.0213	19
387.0425	12	387.0525	16
387.0738	10.5	387.0888	18.25
387.1063	11	387.1213	19.25
387.14	12	387.16	21.75
387.17	13.75	387.19	18.5
387.2063	11	387.2238	18.5
387.24	12.5	387.2563	20
387.2725	10.25	387.2888	25.75
387.3063	11.25	387.3238	22.5
387.3425	12.5	387.3613	21
387.3763	11	387.3963	23.25
387.4063	10	387.4263	25.25
387.4388	12.75	387.4588	21.5
387.475	16	387.49	19.5
387.5063	13.5	387.5225	21.5
387.5413	10.5	387.5575	24.75
387.5738	14.25	387.5913	18.75
387.6063	8.75	387.6238	19.25
387.64	10.75	387.6625	16.25
387.67	12	387.6925	17.5
387.7063	9.5	387.7275	16.5
387.74	14.25	387.7588	18
387.7725	11.25	387.7888	17
387.8063	13.25	387.8238	17
387.8425	15.25	387.8588	17.25
387.8763	11.75	387.8963	15.5
387.9063	12.25	387.9263	16.75
387.9388	11.5	387.9588	18.75
387.975	14.25	387.99	17.75
388.0063	11.25	388.0225	20.25
388.0413	14	388.0575	22.75
388.0763	13.25	388.0913	16.75
388.1063	10.25	388.1238	20
388.14	13.5	388.1625	19
388.17	7.25	388.1925	13.25
388.2063	9.25	388.2275	19.25
388.24	11.25	388.2588	17.5
388.2725	9.75	388.2888	18.25
388.3063	9.25	388.3238	18.5
388.3425	9.75	388.3588	19.25
388.3763	8	388.3938	16

388.4063	8
388.4388	7.75
388.475	10
388.5063	9.25
388.5413	7.75
388.5763	12.75
388.6063	9.25
388.64	13.75
388.67	9.5
388.7063	13.25
388.74	10
388.7725	10.5
388.8063	11.5
388.8425	8.25
388.8763	10.75
388.9063	13.75
388.9388	8.75
388.975	12
389.0063	9.5
389.0413	17
389.0763	9.75
389.1063	13
389.1425	15.25
389.1725	10.75
389.2088	11.25
389.24	11
389.2725	10.25
389.3063	9
389.3425	9.5
389.3763	14
389.4063	9.5
389.4388	12.5
389.475	13.5
389.5088	14.25
389.5388	10.5
389.5763	14.75
389.6063	10
389.64	10.25
389.6725	7.75
389.7063	12
389.7413	11
389.7725	10
389.8075	11.75
389.8425	9
389.8763	11.5
389.9063	12
389.9388	11.5
389.975	10.5
390.0088	9.25
390.0388	10

388.4263	21.5
388.4588	18.25
388.49	18
388.5225	17.25
388.5575	15.5
388.5913	14.75
388.6238	15.25
388.6625	16.5
388.6925	18.5
388.7275	20.25
388.7588	15.5
388.7888	19.25
388.8263	16.5
388.8575	18
388.8925	15.75
388.9263	14
388.9588	18.25
388.99	16
389.0225	17.5
389.0575	18.75
389.0913	18.5
389.1238	17.25
389.1625	17.25
389.1925	17
389.2275	19
389.2588	16.75
389.29	12.5
389.3238	18.25
389.3575	15
389.3938	19.5
389.4263	15
389.46	12.75
389.49	13.5
389.5225	20.5
389.56	14.5
389.5913	19
389.6238	16.25
389.6625	17.25
389.6925	16.75
389.7275	18
389.76	19.75
389.79	15
389.8238	15
389.8575	18.75
389.8913	15.5
389.9263	15.75
389.96	18.75
389.99	20.25
390.0225	16.75
390.06	16.75

390.0763	10.75	390.0913	15
390.1063	8.25	390.1238	16
390.14	8.25	390.16	13.75
390.1725	11	390.1925	16.5
390.2063	12.5	390.2275	12.25
390.24	11.75	390.2588	12.5
390.2725	8.75	390.29	13.5
390.3075	8.75	390.3238	12.75
390.3425	10.5	390.3575	15.5
390.3763	12.75	390.3938	16.5
390.4063	8.25	390.4263	16
390.4388	11.25	390.46	10.75
390.4725	8.75	390.49	14.25
390.5088	10	390.5225	13.5
390.54	15.5	390.56	16
390.5775	8.5	390.5913	16.5
390.61	10.75	390.6238	12.5
390.6413	13	390.66	14.75
390.6738	8.5	390.6925	13.75
390.7075	11.75	390.7275	17.5
390.7425	10.75	390.7588	13.5
390.775	13.25	390.79	17
390.81	9.75	390.8238	13.75
390.845	9.75	390.8575	15.75
390.8788	10.5	390.8913	18.25
390.91	13	390.9263	15.75
390.9413	9	390.96	13.25
390.975	9.75	390.99	16.75
391.01	13	391.025	18.75
391.04	11.75	391.06	16.75
391.0775	8.5	391.0913	18
391.11	8.75	391.1238	15.25
391.1413	10.25	391.16	19.5
391.1738	7.75	391.1925	19
391.2075	7.5	391.2275	17.5
391.2425	11	391.2588	19.75
391.2775	10	391.29	19.25
391.3125	6	391.3238	20.75
391.3488	11	391.3575	19.75
391.38	6.25	391.3925	17.75
391.4125	8.25	391.4263	16.5
391.4438	6.25	391.46	18
391.4763	9.25	391.49	18
391.5113	7	391.525	14
391.5413	10.25	391.56	15.75
391.58	7.75	391.5938	17.25
391.6125	10.75	391.6263	15.75
391.645	10.25	391.6613	14
391.675	13	391.695	12.5
391.71	9.5	391.73	14.5

391.7488	11.5
391.7775	12.25
391.8125	15.5
391.8463	10.5
391.88	16
391.9125	10.25
391.9438	12.5
391.9763	13
392.0113	11
392.0438	10
392.08	12.75
392.1125	11.75
392.145	11.25
392.175	9.5
392.21	9.5
392.245	10.5
392.2775	12.25
392.3125	12.75
392.3463	11.75
392.38	10.5
392.4125	11.25
392.4438	11.75
392.4763	9.75
392.5113	10.25
392.5438	12.75
392.5775	11.75
392.6125	15.25
392.645	12.25
392.6788	17.75
392.7125	13.25
392.7475	10.75
392.78	12.25
392.815	9.25
392.8513	8.75
392.8838	10
392.9175	8.5
392.95	10.5
392.9838	9.25
393.0163	10.75
393.0488	6.75
393.085	8.25
393.1188	9.5
393.1513	8.25
393.1825	10.25
393.2175	9.5
393.2513	10
393.2875	11.5
393.3188	11
393.3525	14.75
393.3875	12

391.7613	18.75
391.7925	18
391.825	18
391.8588	16
391.8963	17.25
391.9275	20.25
391.9613	14.75
391.9913	17.5
392.0263	16
392.0625	15.25
392.0938	16.5
392.1263	18
392.1613	21.25
392.195	16
392.23	15.75
392.2613	21.25
392.2925	16.5
392.325	15.75
392.3588	14.75
392.3963	21
392.4275	16
392.4613	20
392.4913	22.25
392.5263	16.75
392.5625	19
392.5938	19.75
392.6263	14.25
392.6613	16.5
392.6975	18
392.73	22
392.7625	18.5
392.7925	17.25
392.825	16.75
392.8613	15.25
392.8988	16.25
392.93	14.25
392.9638	13.75
392.9938	15
393.0275	14.5
393.0638	17.5
393.095	18.25
393.13	18.75
393.1625	20.25
393.1963	17.75
393.2313	17.25
393.265	17.5
393.2975	17.75
393.3275	15.75
393.3613	15.5
393.3988	18.5

393.4175	13	393.43	17.75
393.4475	10.25	393.4638	16.25
393.4838	11.75	393.4938	14.5
393.5188	12.75	393.5275	17
393.5488	10.75	393.5638	15.5
393.585	13.25	393.595	16.75
393.6188	12.75	393.63	17.5
393.6513	13.25	393.6625	16.25
393.6825	10.75	393.6938	14
393.72	10	393.7313	16.25
393.7513	12	393.765	14.25
393.7863	14.5	393.7963	17.5
393.8188	12.75	393.8275	16
393.8525	9.75	393.8613	16
393.8875	13.5	393.8963	14.75
393.9175	11	393.93	18.25
393.9475	12.5	393.9638	19.25
393.9838	12.75	393.9938	15.75
394.0188	11	394.0275	17.75
394.0488	10	394.0638	17.75
394.085	14	394.095	15.25
394.1188	8.5	394.1325	13.5
394.1513	12.75	394.1638	14.25
394.1825	12	394.195	15
394.22	13.5	394.2325	13.75
394.2513	9.5	394.2675	17.5
394.2863	10.5	394.2988	12.75
394.3188	12.5	394.3313	14.75
394.355	11.25	394.3625	16.5
394.3875	10.75	394.3988	13
394.4175	10.75	394.4325	12.25
394.4475	11.5	394.4663	14
394.4838	10.5	394.4988	17
394.5188	10.5	394.5288	15.25
394.5488	11.5	394.565	16
394.585	13.25	394.5975	15
394.6188	14.25	394.6325	11.75
394.6513	13	394.6638	12.75
394.6825	11.75	394.695	16
394.72	13	394.7325	14.75
394.7525	8.75	394.7675	13.75
394.7863	9.75	394.7988	17
394.8163	14	394.8313	15.5
394.8525	12.25	394.8625	14
394.8875	12.75	394.8988	15
394.9175	14.5	394.9325	18.25
394.9475	11.5	394.9663	11.25
394.9838	9.25	394.9988	18
395.0188	13	395.0288	13.25
395.0488	10.5	395.065	11.5

395.085	9.75	395.0975	14.5
395.1188	14	395.1325	14.25
395.1513	11.25	395.1638	15.75
395.1825	10	395.195	19.25
395.22	12.75	395.2325	10.5
395.2538	14	395.2675	13.75
395.2863	11.25	395.2988	11.75
395.3163	10.5	395.3313	15.5
395.3525	10.5	395.3625	13.25
395.3875	12.25	395.3988	16.5
395.4175	10.25	395.4325	15.25
395.4475	13.25	395.465	15.25
395.4838	14.25	395.4988	16.25
395.5188	9	395.5288	21.25
395.5488	12	395.565	17.75
395.585	11.5	395.5975	14
395.6188	10.5	395.6325	18
395.6513	9.25	395.6638	18.25
395.6825	10.5	395.695	13.75
395.72	11.75	395.7338	15.5
395.7538	13.25	395.7675	18.75
395.7863	14.25	395.7988	15.25
395.8163	12.25	395.8313	14.5
395.8525	15	395.8625	18.5
395.8875	9.75	395.8988	12.5
395.9175	11.5	395.9325	14.75
395.9475	15	395.965	19
395.9838	12.5	395.9988	18
396.0188	16.5	396.0288	15.25
396.0488	13.25	396.065	15
396.085	10.75	396.0975	13
396.12	12.5	396.1325	15.5
396.1525	11.75	396.1625	15
396.1825	16.75	396.195	11.5
396.22	11.75	396.2338	9.75
396.2538	12.5	396.2675	13
396.2875	10.25	396.2988	11.5
396.3163	11.5	396.3313	8
396.3538	14.5	396.3625	15.5
396.3875	12.5	396.3988	14.5
396.4175	11.25	396.4325	12
396.4475	11	396.465	17.25
396.4838	16.25	396.4988	13.75
396.5188	10.75	396.5288	14.75
396.5488	13.5	396.565	16.25
396.585	13.75	396.5975	15.5
396.62	11.75	396.6325	16
396.6513	14.75	396.6625	13.75
396.6825	14.25	396.695	17.5
396.72	13.5	396.7338	17.25

396.7538	14.5	396.7675	14.5
396.7863	14.75	396.7988	15.5
396.8188	15.5	396.8313	16
396.8525	11.75	396.8625	18.25
396.8875	14	396.8988	17
396.9175	13.25	396.9325	14
396.95	16.5	396.9675	17.25
396.9838	12.75	396.9988	16.5
397.0188	13	397.0288	15
397.0488	12.5	397.065	19
397.085	12.5	397.0975	16.25
397.12	12.25	397.1325	14
397.1513	14	397.1625	18.25
397.1825	12.5	397.195	15.5
397.22	10.5	397.2338	16.25
397.2538	12.75	397.2675	17.5
397.2863	13.25	397.2988	15.5
397.3188	14.25	397.3313	13.75
397.3525	15.75	397.3625	20.75
397.3875	13.25	397.3988	15.75
397.4175	13.75	397.4325	18.25
397.4475	13	397.4675	18.25
397.4863	14.5	397.4988	15.75
397.5188	14	397.5313	17.5
397.5488	11	397.565	15
397.585	11.25	397.5975	15.75
397.62	12.75	397.6325	18.75
397.6513	11.75	397.6625	19
397.6825	10.25	397.695	16.25
397.72	9.75	397.7313	19
397.7538	10.75	397.7675	17
397.7863	12.5	397.7988	17.25
397.8188	7	397.8313	17.75
397.8525	11.75	397.8625	16.75
397.8875	10.75	397.8988	15.5
397.9175	11	397.9325	13.5
397.9475	13.25	397.9675	13.75
397.9863	11.25	397.9988	13.75
398.0188	13.25	398.0313	18.25
398.0488	13.75	398.0675	13.75
398.085	12.75	398.0988	22
398.12	15.25	398.1338	15
398.1513	10.25	398.165	14.5
398.1825	10.25	398.1975	15.25
398.22	13.75	398.235	17.75
398.2538	13	398.2688	18.5
398.2863	15	398.3013	19
398.3188	12.5	398.3338	14.25
398.3525	13.75	398.365	15.75
398.3875	14.25	398.4013	11.25

398.4175	14.25	398.4313	16.25
398.45	13	398.4688	14.25
398.4863	15.25	398.5013	13.5
398.5188	13.25	398.5338	16
398.5488	16.25	398.5688	11.5
398.5838	14.75	398.5988	14
398.62	10	398.6338	15.5
398.6513	13.75	398.665	13
398.6825	16.25	398.7	13.25
398.7175	16.5	398.735	15.25
398.7538	18	398.7688	15.25
398.7863	14.25	398.8013	13.75
398.8188	16.25	398.8338	16
398.8525	15	398.865	15.75
398.8888	14.75	398.9013	13.5
398.9175	15.25	398.9313	12
398.95	14.5	398.9688	12.5
398.9863	12.25	399.0013	12
399.0188	11.5	399.0338	11.75
399.0488	8.25	399.0688	10.5
399.0838	14.5	399.0988	16.75
399.12	14.25	399.1338	14.75
399.1513	10.25	399.165	16.25
399.1825	12.25	399.2	15.5
399.2213	12.25	399.2338	16.75
399.2538	9.75	399.2688	18.25
399.2863	11	399.3013	20.75
399.3213	9.5	399.3338	18
399.3525	11.25	399.365	19.5
399.3875	9.5	399.4013	15.25
399.4175	9.25	399.4313	18.75
399.45	12	399.4688	19.25
399.4888	10.25	399.5013	19.5
399.5188	9	399.5338	18.25
399.5488	10	399.5688	18.25
399.5838	7.75	399.5988	20
399.62	8.75	399.6338	23.25
399.6513	7	399.665	16.25
399.6825	7.75	399.7	17.75
399.7213	7.25	399.7363	15
399.7538	5.75	399.7688	18.75
399.7863	8.5	399.8013	16.75
399.8188	7.5	399.8338	17.25
399.8525	8.5	399.865	16.5
399.8875	6.25	399.9013	20
399.9175	10.75	399.9313	19.5
399.95	11.25	399.9675	16.25
399.9888	12.25	400.0038	20.25
400.0188	9.75	400.0338	20
400.0488	11.75	400.0688	21.5

400.0838	8.5	400.0988	23
400.12	13.75	400.1338	21
400.1513	11.25	400.165	20.25
400.1825	10.25	400.2	22.5
400.2188	8.75	400.2338	18.75
400.2538	9	400.27	16.25
400.2863	7.75	400.3013	16.25
400.3188	10	400.3338	21.25
400.3525	9.75	400.365	13.75
400.3875	10	400.4013	18.75
400.4175	6.25	400.4313	14.25
400.45	6	400.4675	17.5
400.4913	6.75	400.5013	17.75
400.5213	10.5	400.5338	21.5
400.5513	8.5	400.5688	18.25
400.5863	7.5	400.5988	19.25
400.6213	7.25	400.6338	22
400.6525	11.5	400.665	18
400.6863	10	400.7	17.75
400.7225	9.75	400.7338	16
400.755	9.5	400.77	19
400.7875	8.5	400.8013	14.25
400.82	9.25	400.8338	17.25
400.8575	9.25	400.8688	17.25
400.89	12.75	400.9013	18.5
400.9213	12.75	400.9325	16
400.9525	12.25	400.9675	22
400.9913	9.5	401.0013	20.75
401.0213	11.75	401.0338	19.5
401.0513	14	401.0688	17.5
401.0863	12.75	401.1013	22.75
401.1213	13.5	401.1338	22.25
401.1525	11.5	401.165	25.75
401.1863	11.25	401.1975	22.25
401.2225	12.5	401.2338	21
401.255	11.25	401.27	19
401.2875	13	401.3013	18.5
401.32	16.25	401.3338	25.25
401.355	14.75	401.3688	25.25
401.39	14	401.4013	19
401.4213	13.25	401.4325	21
401.455	13.25	401.4675	21.5
401.4913	15	401.4988	23.5
401.5213	17	401.5338	22.25
401.5525	15.25	401.5688	19.25
401.5863	14.75	401.5988	20
401.6213	9	401.6338	17.75
401.6525	9.75	401.665	23.75
401.6863	13.25	401.6975	19.75
401.7238	11	401.7338	18.75

401.755	11.25	401.77	18.25
401.7875	12.75	401.8013	20.25
401.82	10.25	401.8338	19
401.855	9.75	401.8688	19.75
401.89	12.75	401.9013	16.25
401.9213	14.25	401.9325	20.75
401.955	9.25	401.9675	20.25
401.9913	14.75	401.9988	18.5
402.0213	10.5	402.0338	19
402.0525	8.75	402.0688	21.25
402.0863	12.75	402.0988	22
402.1213	7.5	402.1338	19.75
402.1525	9.5	402.165	16.75
402.1863	11	402.1975	16.5
402.2225	9	402.2338	18.25
402.255	10.5	402.27	22.5
402.2875	12.25	402.3013	21.25
402.32	11	402.3338	16.5
402.355	11.5	402.3688	21.5
402.39	10.25	402.4013	18.75
402.4213	11.75	402.4325	17
402.455	11	402.4675	17.75
402.4913	8.25	402.4988	19
402.5213	8	402.5338	16.25
402.5525	11.25	402.5713	15.5
402.5863	13.25	402.6025	19.25
402.6213	10.5	402.6363	17.25
402.6525	8.75	402.6675	17.75
402.6863	8	402.7	17
402.7225	10.75	402.7363	20.5
402.7575	11	402.7713	17.75
402.7875	10.25	402.8025	17.25
402.82	11	402.835	15.75
402.855	10.75	402.8713	14.75
402.89	12	402.9025	11.75
402.9225	11.25	402.9338	14
402.955	11.25	402.9675	14.25
402.9913	11.5	403.0013	14.5
403.0213	12.25	403.0363	17
403.0525	11.5	403.0713	15.75
403.0863	15	403.1025	20.5
403.1213	14.25	403.1363	16.5
403.1525	12.25	403.1675	19.75
403.1863	18.5	403.2	23.25
403.2225	14.25	403.2338	15.5
403.255	13.25	403.2713	16.25
403.2875	14.25	403.3025	16.5
403.32	17.5	403.335	18.75
403.355	13.75	403.3713	14.5
403.39	15	403.4025	19.75

403.4213	12.5	403.4338	18
403.455	11.25	403.4675	14
403.4913	10.25	403.5013	17.5
403.5238	10.5	403.5363	18
403.5525	14	403.5713	21.5
403.5863	12.5	403.6025	17
403.62	13.5	403.6363	15.25
403.655	11.75	403.6688	18.25
403.6875	16.25	403.7	18.5
403.7225	12.75	403.7338	16.5
403.755	10.5	403.7713	14
403.7875	10.75	403.8025	22.75
403.8225	10.25	403.835	18
403.855	7.5	403.8713	18.75
403.89	8	403.9025	17.75
403.9213	11.5	403.9338	19.75
403.955	13.75	403.9675	16.5
403.9913	13.75	404.0038	16.5
404.0213	9	404.0375	20.5
404.0525	12.75	404.0713	20.5
404.0863	11	404.105	21.25
404.1238	15.25	404.1363	22
404.155	15	404.1675	16.25
404.1875	16.5	404.2	19.75
404.2225	17.25	404.2338	20.5
404.255	13.75	404.2713	18.5
404.2875	12.5	404.3025	19.75
404.32	18.25	404.335	18.75
404.355	13.5	404.3713	19
404.39	13.25	404.4025	16.5
404.4213	16	404.4338	20.75
404.455	15.75	404.4675	21.25
404.4913	16.5	404.5038	19.75
404.5213	17.25	404.5363	16.75
404.5525	13.25	404.5713	20
404.5863	12.5	404.605	23.25
404.62	12.75	404.6363	18.25
404.655	14	404.6675	18.5
404.6875	10	404.7	19.25
404.72	12.75	404.7338	17.75
404.755	9.5	404.77	21
404.79	11.75	404.8025	17.75
404.8213	11.25	404.835	20.5
404.855	10	404.8713	22.5
404.89	10.5	404.9025	17.75
404.9213	11.5	404.9338	18.25
404.955	12.75	404.9675	20.5
404.9913	13.5	405.0038	16
405.0213	16.25	405.0363	18
405.0525	16.75	405.0713	18

405.0875	17.75	405.105	16.75
405.1225	13	405.1363	18.75
405.155	18.25	405.1675	16
405.1875	14.25	405.2	18.25
405.22	15.25	405.2313	14
405.255	13.75	405.27	15.75
405.2913	13	405.3025	16.25
405.32	13.25	405.3375	18.75
405.355	17.75	405.3713	17.5
405.39	14.25	405.4025	17.25
405.4238	19.25	405.4338	15.25
405.455	14.25	405.4675	18.75
405.4913	13.5	405.5025	20.5
405.5213	12	405.5363	19.5
405.5525	15.25	405.5713	18.25
405.5875	13	405.605	22
405.62	13	405.6363	22.75
405.655	13.5	405.6675	22
405.6875	14.75	405.7	20
405.7213	15	405.7313	17.5
405.755	14	405.77	20.25
405.79	15.25	405.8025	21.5
405.82	14.25	405.8375	19.5
405.8575	14.25	405.8713	18.25
405.8888	12.75	405.9025	21.25
405.9238	15.5	405.9338	18.5
405.955	15.75	405.9675	17.5
405.9913	15	406.0025	17
406.0213	15.75	406.0363	20
406.0525	18.75	406.0713	16.5
406.0875	12.5	406.1075	21.25
406.12	16.75	406.1363	16.25
406.1575	17.25	406.1675	19.75
406.19	17.75	406.2025	20.25
406.2238	13.25	406.2313	17.75
406.2563	12	406.27	20.5
406.2913	14.5	406.3025	16.75
406.3213	10	406.3375	18.5
406.3588	13.75	406.3713	19.5
406.39	15.5	406.4025	17.5
406.425	12.75	406.4338	19
406.4588	16.5	406.4675	18
406.4938	13.25	406.5025	16.5
406.5238	11.5	406.5363	19.5
406.555	11.75	406.5713	15.75
406.59	12.75	406.605	16.75
406.6225	14.5	406.6363	18.5
406.6575	14	406.6675	18.25
406.69	13.25	406.7	18.25
406.7238	10.5	406.7313	19.75

406.7563	16.25	406.77	22
406.7913	15.25	406.8038	19.5
406.8225	13.25	406.8388	14.75
406.8588	14.25	406.8713	19
406.89	15	406.9025	20.75
406.925	12.75	406.9338	17.25
406.9563	16.25	406.9675	20.5
406.9938	15.75	407	16.25
407.025	13.5	407.0363	18.25
407.0575	13.5	407.0713	15.25
407.09	15	407.105	21.5
407.1225	12	407.1363	19.75
407.16	10.5	407.1675	18.25
407.19	12	407.2025	20
407.2238	10.25	407.235	22.5
407.2563	7.5	407.2713	18.25
407.2913	10	407.305	20.25
407.3213	10	407.3413	16
407.3588	12	407.3738	17.5
407.39	11.5	407.405	23.25
407.425	12.25	407.4363	17.25
407.4563	13.5	407.4688	17.75
407.4938	12.75	407.5025	19.5
407.525	14	407.5388	16.5
407.555	13.75	407.5725	22.75
407.59	12.75	407.6063	19.5
407.625	14.75	407.6375	18
407.66	16.75	407.6688	19.5
407.69	16	407.7025	19.5
407.7238	18	407.735	18
407.7563	15.5	407.7713	19
407.7913	16.25	407.805	16.5
407.8213	16.25	407.8413	15.5
407.8588	17.75	407.8738	19.25
407.89	15.5	407.905	18.5
407.925	14.25	407.9363	18.5
407.96	16	407.9688	17
407.9938	14.75	408.0025	17.5
408.025	14	408.0388	16.75
408.055	15.5	408.0725	18.25
408.09	11.5	408.1063	18.5
408.125	15	408.1375	18
408.1588	14.75	408.1688	17
408.19	14	408.2025	20.5
408.2238	15.25	408.2338	18
408.2575	13.25	408.2688	21.25
408.2913	12.75	408.305	19.75
408.3213	15	408.3413	22
408.3588	16	408.375	21.25
408.39	12.75	408.405	23.25

408.425	12.75	408.4363	22.25
408.4563	14.25	408.4688	18.5
408.4938	11.75	408.5025	22.75
408.525	14.75	408.5388	17.75
408.555	15.25	408.5725	18
408.59	13.5	408.6063	16
408.625	16	408.6375	22
408.6588	19	408.6688	18
408.69	16	408.7025	19.75
408.7238	15.25	408.7338	21.25
408.7563	19.25	408.7688	19.75
408.7913	19.5	408.805	19.5
408.8213	20.5	408.8413	15.75
408.8613	18	408.8738	23.25
408.8913	17.75	408.905	21
408.9225	21.75	408.9363	21.5
408.9563	19.5	408.9688	23.25
408.9938	15.25	409.0025	20.5
409.025	19.25	409.0388	23.5
409.055	20.25	409.075	26.25
409.09	12.25	409.1063	23.25
409.125	13.25	409.1375	19.75
409.1588	11.5	409.1688	27.5
409.19	14.5	409.2025	22.25
409.2238	11.75	409.2338	24.75
409.2588	9.75	409.2688	23.75
409.2913	12	409.305	28.25
409.3238	12	409.3413	27.25
409.36	13.75	409.3738	26.25
409.3913	11	409.405	23
409.4225	11.5	409.4363	20.5
409.4588	12.5	409.4688	27
409.4938	13.75	409.5025	25
409.525	16.75	409.5388	21.75
409.555	21	409.5738	22.5
409.59	19	409.6063	22
409.625	19.25	409.64	22
409.6588	14.75	409.6688	20.5
409.69	17	409.7025	22.75
409.7238	16	409.7363	22.75
409.7588	17	409.7688	24.5
409.7913	14.5	409.8075	28.25
409.8238	15	409.8413	23
409.86	15.75	409.8738	22.25
409.8913	17.25	409.905	25.25
409.9225	18.5	409.9363	21
409.9563	19.25	409.9688	24.75
409.9938	18.5	410.0025	26.25
410.025	14.25	410.0388	25.25
410.0575	19.25	410.0738	28

410.09	17	410.1063	23.75
410.1275	15.5	410.1375	29.75
410.1588	18.25	410.1688	22.5
410.19	16.5	410.2025	27
410.2238	14.75	410.2338	28
410.2588	17.5	410.2688	25.25
410.2913	16	410.305	25
410.3238	14.75	410.3413	22.75
410.36	17.5	410.3738	25
410.3913	17.25	410.405	22.75
410.4225	16.25	410.4363	23
410.4563	20.25	410.4688	23
410.4938	15.5	410.5025	21.5
410.525	20	410.5388	19.5
410.555	18.25	410.5738	23
410.59	18.25	410.6063	22.25
410.6275	19.75	410.6375	19.25
410.66	20.5	410.6713	21.75
410.6888	16.75	410.7025	24.5
410.7238	16.25	410.7338	21
410.7588	15.75	410.7688	18.75
410.7913	19.25	410.805	16.5
410.8263	16	410.8413	19.25
410.86	15.5	410.8775	19.75
410.8938	13.75	410.905	17.5
410.9225	13	410.935	20.75
410.9563	12.75	410.9688	21.5
410.9938	12.25	411.0025	24.25
411.025	16.75	411.0388	19.75
411.055	11.75	411.0738	22.5
411.0925	10.5	411.1063	23.25
411.1275	12.25	411.1375	19.75
411.1588	11.5	411.1688	22.75
411.1888	11.5	411.2025	21.75
411.2238	10	411.2338	22.75
411.26	13	411.2675	23.75
411.2913	14	411.305	21
411.3238	11.25	411.34	24
411.36	10.75	411.3738	25.25
411.3913	10.25	411.405	27.75
411.425	13.25	411.435	26.25
411.4563	10.25	411.4688	20
411.495	10.25	411.5025	24.5
411.525	12.25	411.5388	21.5
411.555	11.25	411.5738	26.5
411.5925	12.75	411.6063	27.25
411.6275	10.75	411.64	28.25
411.6588	12	411.6713	24.75
411.6888	10	411.705	24.5
411.7238	9	411.735	26.5

411.7588	11.25	411.7713	23.25
411.7913	14.5	411.8088	21.75
411.8238	11	411.8425	28.75
411.8613	10.25	411.875	25.5
411.8913	11.5	411.9063	23
411.9225	11.5	411.9363	23
411.9563	9.75	411.9713	30
411.995	14	412.005	18
412.025	13.5	412.04	19.5
412.055	11.75	412.075	18
412.0925	12	412.1088	21.5
412.1275	12.75	412.14	21.5
412.1588	13	412.1713	17.5
412.1888	12.25	412.205	23.75
412.2238	13	412.235	21.75
412.2588	16.75	412.2738	23
412.2913	12.5	412.3088	22.5
412.3238	12	412.3425	15
412.3613	10.75	412.375	22
412.3913	9	412.4063	18.25
412.4225	14.75	412.4363	20.75
412.4613	11.25	412.4713	21.75
412.495	10.5	412.505	20
412.525	11.25	412.54	20
412.555	14	412.575	23.5
412.5925	11.75	412.6088	23.5
412.6275	9.5	412.64	21.5
412.6588	11	412.6713	21.5
412.6888	10.75	412.705	22.5
412.7238	11.75	412.735	23.75
412.7588	13.75	412.7713	21
412.7913	12	412.8088	19
412.8263	10.5	412.8425	19.5
412.8613	11.25	412.875	18.25
412.8913	13.75	412.9063	22.75
412.9225	11.75	412.9363	18
412.9563	13.75	412.9713	19.25
412.995	10.25	413.005	22.25
413.025	11.5	413.0388	23.75
413.055	11.75	413.075	22.75
413.0925	15.75	413.1063	21.25
413.1275	12	413.14	23
413.1588	10.75	413.1713	19.75
413.1888	11.5	413.205	19.75
413.2238	9.5	413.235	18.75
413.2588	11	413.2688	19
413.2913	11.75	413.3088	18
413.3263	10	413.3425	21.5
413.3613	10.25	413.375	21.25
413.3913	12.75	413.4063	20

413.4225	13	413.4363	23
413.4563	10.75	413.4713	20.5
413.495	10.75	413.505	19
413.525	11	413.5388	18.25
413.555	10.75	413.575	18.75
413.5925	13.5	413.6063	20
413.6275	11.5	413.64	21.75
413.6625	11.25	413.6713	21.75
413.6888	11.25	413.705	18.5
413.7238	13.25	413.735	21.25
413.7588	14.25	413.7688	18.5
413.7913	13.25	413.8088	19
413.8263	16.25	413.8425	18.25
413.8613	10	413.875	17.75
413.8913	13.75	413.9063	22
413.9225	10.75	413.9363	22.25
413.9563	12.25	413.9713	22.75
413.995	10.75	414.005	28
414.025	13.5	414.0388	16.5
414.0563	12	414.075	20.75
414.095	12.5	414.1063	23.75
414.1275	13	414.14	16.75
414.1588	9.5	414.1713	23
414.1888	14	414.2025	23.5
414.2238	12.5	414.235	24.25
414.2588	13.25	414.2688	22.75
414.2913	12.25	414.3113	20.25
414.3263	12.25	414.3425	23.75
414.3613	14.25	414.3738	20
414.3913	12.5	414.4063	21
414.4225	13	414.4363	26.25
414.4563	12.5	414.4713	24
414.495	13.25	414.505	20.5
414.525	11.75	414.5388	22
414.5563	13	414.575	22
414.595	16.75	414.6063	21.75
414.6275	13	414.6425	25
414.6588	14	414.6713	24.75
414.6888	11.75	414.7025	26
414.7263	13	414.735	21.5
414.7588	11.75	414.7688	23.75
414.7913	13.5	414.8113	24.5
414.8263	10.75	414.8425	21.75
414.8613	8.75	414.8738	19.25
414.8913	12.75	414.9063	25.75
414.9225	14.5	414.9363	21
414.9588	13.5	414.9713	16.75
414.9938	13	415.005	22
415.025	11.25	415.0413	21
415.0563	14.75	415.075	20.25

415.0975	8.75	415.1063	15.5
415.1275	13.25	415.14	21
415.1588	11.5	415.1713	15.25
415.1888	11.25	415.2025	18
415.2238	11.25	415.235	22.5
415.2588	13.25	415.2688	19.5
415.2938	14.5	415.3113	21.25
415.3288	12.5	415.3425	20.5
415.3613	13.75	415.3738	22.25
415.3913	14.25	415.4063	18.25
415.4225	19.5	415.4363	24.5
415.4588	13.75	415.4713	22.25
415.495	14	415.505	19
415.525	13.5	415.54	22.75
415.5563	14.25	415.575	23.75
415.595	15.25	415.6088	25.5
415.6275	11.5	415.64	21.25
415.6588	12	415.6713	21.5
415.69	8.75	415.7025	23.5
415.7238	12.75	415.735	19.25
415.7588	12.5	415.7688	18.5
415.7938	12.5	415.8113	27.25
415.8263	12.5	415.8425	21.25
415.8613	17.25	415.8738	26.25
415.8913	14	415.9063	16.75
415.9263	12	415.9363	19.5
415.9588	14.5	415.9688	24
415.9938	13.75	416.005	20.5
416.025	14	416.04	19.25
416.0563	13.25	416.075	21.5
416.0975	17.5	416.1063	20
416.1275	13.75	416.1375	25.25
416.1588	18	416.1713	20
416.1913	10.75	416.2025	20.25
416.2238	15.25	416.235	21
416.2588	16.25	416.2688	27.25
416.2938	17.25	416.3113	17
416.3288	12	416.345	20
416.3613	15.5	416.3738	21.25
416.3913	17	416.4063	21.5
416.4225	18	416.4363	19.5
416.4588	19	416.4688	18.75
416.4938	15.75	416.505	22.5
416.525	16.75	416.54	19
416.5563	13.75	416.575	24
416.5975	11.25	416.6063	19.5
416.6275	12.5	416.6375	18.75
416.6588	17	416.6713	23.25
416.6913	15.25	416.7025	22.75
416.7238	12	416.7375	23.75

416.7563	19	416.7688	23.25
416.7938	14.5	416.8113	21.75
416.8275	14.75	416.8425	21.25
416.8613	12.5	416.8738	22
416.8913	18	416.9063	19.25
416.925	13	416.9363	20.25
416.9588	12	416.9688	20.75
416.9913	14	417.005	22
417.0263	14.25	417.04	20.25
417.0563	16.25	417.075	23
417.0975	15.5	417.1063	23.5
417.1275	14.75	417.1375	22.75
417.1588	13.75	417.1713	21
417.1913	16.25	417.2025	23
417.2238	16.25	417.235	20.75
417.2563	15.75	417.2688	23
417.2938	15.5	417.3113	23.5
417.3275	15	417.3425	22
417.3613	14	417.3738	24.25
417.3913	12.5	417.4038	23.25
417.4225	14.5	417.4363	20.75
417.4588	16	417.4688	22.25
417.4913	14.75	417.505	23.75
417.5288	14.75	417.54	21.25
417.5563	16.5	417.575	19
417.5975	16.5	417.6063	24.75
417.63	16.75	417.6375	20.25
417.6588	20	417.6713	21.5
417.6938	21	417.7025	24.5
417.7238	19.25	417.735	21.5
417.7563	16.75	417.77	24.5
417.7938	18.5	417.8125	22.25
417.8275	16	417.8425	24
417.8613	18	417.8738	19.75
417.8913	17.25	417.9038	22.5
417.9225	14	417.9363	23.75
417.9588	14.75	417.9688	21.75
417.9913	17.75	418.005	25.5
418.0263	13.75	418.04	27.75
418.0563	15.25	418.075	28
418.0975	14.5	418.1063	27.75
418.1275	17.5	418.1375	30.5
418.1588	17	418.1713	21.5
418.1913	12.25	418.2025	19.5
418.2238	16.75	418.2338	27.25
418.2588	17.75	418.2688	27.25
418.2938	17	418.3088	24.5
418.325	18.75	418.3425	24
418.3613	16	418.3738	27.75
418.3913	16	418.4038	29

418.425	18.5	418.4363	28.25
418.4588	15	418.4713	26.75
418.4913	19.75	418.505	25
418.525	15.25	418.54	26.5
418.5563	17.25	418.575	23.25
418.5975	20.25	418.6063	26.25
418.6275	18.5	418.6375	22
418.6588	18.25	418.67	24
418.6913	19.75	418.7025	27
418.7263	18.25	418.735	26
418.7588	18	418.7688	21
418.7938	15.75	418.8088	23.25
418.825	18	418.8425	24.5
418.8613	16.25	418.8738	20.5
418.8913	15.5	418.9038	28.5
418.9275	15.5	418.9363	26.25
418.9588	15	418.9688	22.25
418.9913	17.25	419.005	23.75
419.025	14.5	419.0413	20.5
419.0563	14	419.075	21.25
419.0975	16.5	419.1063	26.75
419.1275	15	419.1375	21.25
419.1588	17	419.17	19.75
419.1938	18.25	419.2025	23.25
419.2263	13.25	419.235	18
419.2613	15	419.2688	21.25
419.295	15	419.3063	21.25
419.3263	16.5	419.3425	17
419.3625	19	419.3738	18.25
419.3938	14.25	419.4038	18.25
419.4288	12.5	419.4363	16.25
419.46	15.5	419.4688	18.25
419.4925	14.25	419.505	19.5
419.5275	15.75	419.5388	19.25
419.5588	14.25	419.575	19.25
419.6	17.5	419.6063	19.5
419.63	13.75	419.6375	25.25
419.6625	15	419.67	21
419.6938	10	419.7025	19.5
419.7263	12.25	419.735	18
419.7613	13.5	419.7688	25.25
419.795	12	419.8063	20
419.8263	10.75	419.8425	19
419.8625	16	419.8738	19.75
419.8938	12	419.9038	21.5
419.9288	15	419.9338	17.5
419.96	13.5	419.9688	19.75
419.9925	13.75	420.005	23
420.0275	12.25	420.0388	22.25
420.0588	15.25	420.075	21.75

420.1	12.5	420.1063	20
420.13	11.75	420.1375	24.75
420.1638	12	420.17	20.25
420.1938	13.5	420.2025	18.25
420.2263	13.5	420.235	21.5
420.2613	14.5	420.2688	20.5
420.295	11.75	420.3063	22.5
420.3263	11.25	420.3425	22.25
420.3625	13.75	420.3738	19.5
420.3938	14.75	420.4038	21
420.4288	11	420.4338	21.25
420.46	11.75	420.4688	18.5
420.4938	9.5	420.5025	17
420.5288	11	420.5388	18.5
420.5613	9.25	420.575	18.5
420.6	9.5	420.6063	15.25
420.63	8.75	420.6375	17
420.6638	10.25	420.67	17.25
420.6938	14.25	420.7025	14.75
420.7263	14.25	420.735	18
420.76	13.5	420.7688	17.25
420.795	13	420.8063	18.5
420.8263	8	420.8425	14.75
420.8625	8	420.8738	18
420.8938	9.25	420.9038	19
420.9288	13	420.9338	15.75
420.96	13.5	420.9688	15.5
420.9938	13	421.0025	14.5
421.0275	12.25	421.0388	20
421.0613	14.5	421.075	21
421.0988	13.25	421.1063	16
421.13	11	421.1375	21
421.1663	15.75	421.17	18.75
421.1938	15	421.2013	19.5
421.2263	17.75	421.235	19.75
421.26	18.25	421.2688	18.75
421.295	14.5	421.3063	21.25
421.3263	16.25	421.3425	20.25
421.3625	19.25	421.3738	19.5
421.3938	17	421.4038	17.75
421.4288	21.5	421.4338	20.25
421.46	18.25	421.4713	19.25
421.4938	21.25	421.5025	20
421.5275	17.5	421.5388	19.25
421.5613	18.25	421.575	20.25
421.5988	15.25	421.6063	15.25
421.63	18.5	421.6375	19.5
421.6638	12.5	421.67	18
421.6938	14.25	421.7013	19.5
421.7288	15.25	421.735	17.25

421.76	17.5	421.7688	19
421.7925	13.5	421.8063	17.5
421.8263	14.5	421.8425	16.75
421.8625	14	421.8738	18
421.8963	13.75	421.9038	15
421.9288	13	421.9338	16
421.96	15.75	421.9713	20.75
421.9938	14.75	422.0025	21.75
422.0275	12.75	422.0388	12.75
422.0613	17	422.075	15.75
422.1	18	422.1063	17.75
422.13	15.25	422.1375	18.75
422.1638	13	422.17	20
422.1938	8.25	422.2013	20.5
422.2263	14	422.235	18.75
422.26	14	422.2688	17.5
422.2925	15.5	422.3063	16.75
422.3288	13.5	422.3425	19
422.3625	14.75	422.3738	15.75
422.3963	14.25	422.4038	14.25
422.4288	16	422.4338	13
422.46	14	422.4713	17.25
422.4938	16	422.5025	15.75
422.5275	14	422.5388	15
422.5638	16.5	422.575	15.25
422.6025	13.25	422.6063	16.5
422.6325	15.5	422.6375	15
422.6663	14.75	422.67	15.5
422.6963	14.75	422.7013	19
422.73	15	422.735	18
422.7613	14.75	422.77	16.75
422.7938	11	422.8038	17
422.8288	16.25	422.8425	14.5
422.8638	15	422.8738	18.25
422.8975	11.25	422.9038	20.25
422.93	18.5	422.9338	19.75
422.9625	14.25	422.9713	14.5
422.9963	14	423.0025	16.25
423.0275	15.75	423.0388	20.5
423.0638	15.75	423.075	18.75
423.1	15.5	423.1063	15
423.1325	16.25	423.1375	15.75
423.1663	16.5	423.17	18.25
423.1963	14.25	423.2013	17.25
423.23	15	423.235	18
423.2613	13.5	423.27	15.5
423.2938	14.25	423.3038	20
423.3288	15.25	423.3425	17.75
423.3638	9.75	423.3738	17.5
423.3975	14.5	423.4038	14.75

423.43	12.75	423.4338	13.5
423.4625	14.25	423.4688	17.75
423.4963	16	423.5025	19.5
423.5288	12.25	423.5388	16
423.5638	13.25	423.575	20.5
423.6	15.75	423.6063	19.25
423.6325	10.75	423.6375	18.25
423.6663	13.25	423.67	16.5
423.6963	12	423.7013	13.5
423.73	14.25	423.735	17.75
423.7613	12.75	423.7713	21.25
423.7938	14.75	423.8038	17.25
423.8288	14.5	423.8425	17.25
423.8638	13.75	423.8763	16.75
423.8975	15.25	423.9038	20.75
423.93	16.5	423.9338	16.75
423.9625	17.25	423.9688	18.5
423.9963	14.75	424.0025	18.75
424.0275	14.75	424.0388	16.5
424.0638	14.75	424.075	20.25
424.1	19	424.1063	15
424.1338	11.75	424.1375	18.25
424.1688	20	424.17	15.25
424.1963	14.5	424.2025	17.5
424.23	13.75	424.235	11.5
424.2613	18.25	424.27	17.25
424.2938	15.25	424.3038	17.75
424.3288	12	424.3425	17
424.3638	21.5	424.3738	18.25
424.3975	14.25	424.4038	17
424.43	15.75	424.4338	19.5
424.4625	14.25	424.4688	18.25
424.4963	13.5	424.5025	16.25
424.5275	17	424.5388	19.75
424.5638	17.25	424.575	20.25
424.6	17	424.6063	19.75
424.6338	16.75	424.6375	20.25
424.6663	14.75	424.67	18.5
424.6963	14	424.705	20.5
424.73	13.25	424.7363	18.5
424.7613	13.5	424.7738	17.5
424.7938	14	424.8063	16
424.8288	16.75	424.8438	18.75
424.8638	15	424.875	14.75
424.8975	19	424.905	18.75
424.93	14.25	424.935	18
424.9638	16.5	424.9713	16.75
424.9963	18.75	425.005	14
425.0275	15	425.04	14.5
425.0638	18.5	425.0775	20.25

425.0975	18.75	425.1088	16.75
425.1338	17.25	425.14	19.5
425.1663	16.25	425.1725	17.25
425.1963	15	425.205	19.25
425.23	13.75	425.2363	15.5
425.2613	15	425.2738	13.25
425.2938	15.25	425.3063	16
425.3288	13.5	425.3438	19.5
425.3638	14.25	425.375	19.25
425.3975	11	425.405	16.5
425.43	11.25	425.435	16.5
425.4638	16.5	425.4713	17.5
425.4963	16	425.505	18.75
425.5275	15	425.54	17
425.5638	14	425.5775	18
425.5975	18	425.6088	17.75
425.6338	15.75	425.64	17
425.6663	17.25	425.6725	13.5
425.6963	17.5	425.705	22
425.73	14.5	425.7363	16
425.7613	13.75	425.7713	21.25
425.7938	18	425.8063	17.25
425.8288	18.5	425.8438	14.75
425.8625	18.25	425.875	15.75
425.8975	13.75	425.905	15
425.93	16.75	425.935	19
425.9638	12.25	425.9713	19.25
425.9963	15.75	426.005	16.75
426.03	14.5	426.04	17
426.0638	17	426.0763	14
426.0975	17.5	426.1088	15.5
426.1338	19.5	426.14	15.75
426.1663	16.75	426.1725	13.75
426.1963	20	426.205	19.25
426.23	16	426.2363	18
426.2613	16.5	426.2713	18
426.295	14	426.3063	13.75
426.3288	12	426.3438	16.75
426.3625	13	426.375	17
426.4	18.75	426.405	17
426.43	15.75	426.435	17.5
426.4638	14.5	426.4713	15
426.4963	12.75	426.5063	19
426.5275	15.25	426.54	20.25
426.5638	13.25	426.5763	14.5
426.5975	12.25	426.6088	17
426.6338	14.5	426.64	17.25
426.6663	14.75	426.6725	18
426.6963	17.25	426.705	18.25
426.73	19.5	426.7363	20.25

426.7613	17.75	426.7713	18
426.7938	17.25	426.8063	18
426.8288	24	426.8438	17.5
426.8625	17.5	426.875	19
426.8988	17.75	426.905	22
426.93	14.75	426.9375	17.75
426.9638	18	426.9713	17.25
426.9975	19.5	427.0063	17.25
427.0275	22	427.0425	17
427.06	18.75	427.0763	19.25
427.0975	20.25	427.1088	17.75
427.1338	19.5	427.14	18
427.1663	18.25	427.1725	18.5
427.1988	19	427.205	17
427.23	20.25	427.2388	19
427.2613	16.25	427.2738	16.75
427.2938	20	427.31	21.75
427.3288	18.25	427.3463	19.25
427.365	16.75	427.3775	17.5
427.3975	15.5	427.4075	19.5
427.43	19	427.44	15.5
427.4638	20	427.4725	16.5
427.4963	17	427.5075	13.75
427.5275	16.5	427.5413	19
427.56	18.25	427.5788	14.5
427.5988	19.5	427.61	16.25
427.6338	15.25	427.6413	17.5
427.6663	19.75	427.6738	18.5
427.6988	17.75	427.7075	17.25
427.73	13.75	427.7388	16.75
427.7613	16.5	427.7738	18
427.7938	21.25	427.81	16.25
427.8288	15.25	427.8463	12.75
427.865	16.75	427.8775	19.5
427.8975	18	427.9075	19.25
427.93	16.25	427.94	13
427.9638	16.75	427.9725	14.75
427.9963	15.25	428.0075	16
428.0275	16.75	428.0413	15.5
428.06	14.75	428.0788	15.25
428.0988	12.25	428.11	15.25
428.1338	18	428.1413	16.25
428.1663	13	428.1738	14.25
428.2	16.5	428.2075	19.5
428.23	19.25	428.2388	15.75
428.2613	20.75	428.2738	14.75
428.2938	16.25	428.31	15
428.3275	15.75	428.3438	17.5
428.365	10	428.3775	19.25
428.3975	14.75	428.4075	15.25

428.43	17.75	428.44	16.5
428.4638	13.25	428.4725	18.75
428.4963	17.75	428.5075	19.5
428.5275	15.75	428.5413	15.75
428.56	20.75	428.5788	17
428.5975	17.75	428.61	16.5
428.6313	16	428.6413	17.25
428.6638	16.5	428.6738	14.5
428.6988	17.25	428.7075	14.75
428.73	17	428.7413	19.25
428.7613	16.5	428.7738	19.5
428.7975	17.25	428.81	18
428.8275	15.75	428.8438	18
428.865	13	428.8775	17.25
428.8975	13	428.9075	17.25
428.93	17.5	428.94	21
428.9638	17.25	428.9725	18.75
428.9963	14	429.0075	16
429.0275	16.25	429.0388	18.25
429.06	13.25	429.0788	18
429.0975	17.75	429.11	21
429.1313	17.25	429.1413	18
429.1638	16	429.1738	17.25
429.1988	13	429.2075	16.5
429.23	12	429.2413	16.75
429.2613	12.5	429.2738	22.5
429.2938	15.25	429.31	18.25
429.3275	16.75	429.3438	19.75
429.365	14.25	429.3775	16
429.4	13	429.4075	20
429.4325	17	429.4425	16.25
429.4638	14.5	429.475	18
429.4963	15.75	429.5075	18
429.53	16.75	429.5375	18.75
429.56	17.25	429.5788	17.25
429.595	18	429.61	13.25
429.6313	16.5	429.6413	18.5
429.6638	18.75	429.6738	16.5
429.6988	17.5	429.7075	16.25
429.73	15.25	429.7413	15.25
429.7613	12	429.7738	16.5
429.7938	14.5	429.81	16.5
429.8275	15	429.8438	15.25
429.865	17	429.8775	19.25
429.8975	16	429.9075	18.5
429.9325	16	429.94	17
429.9638	11.5	429.975	21
429.9975	12.25	430.0075	17.25
430.03	14.75	430.0375	11.75
430.06	12.25	430.0788	16.25

430.0963	14.75	430.11	16.75
430.1313	13	430.1413	16.5
430.1638	13.5	430.1763	18.75
430.1988	14.75	430.2075	15.75
430.23	13.75	430.2413	18.25
430.2613	12.75	430.2738	18.75
430.2938	12	430.31	18.5
430.3275	11.5	430.3438	20.25
430.365	12.5	430.3775	21
430.3975	14	430.4075	16
430.4325	13.25	430.44	17.75
430.4638	12.25	430.4775	18.25
430.4963	11.75	430.5075	19.75
430.53	13.5	430.5375	17
430.56	15.25	430.58	21.5
430.6	10	430.6088	16.75
430.6313	12	430.6413	18
430.6638	15.5	430.6763	20.25
430.6988	14.75	430.7075	20.25
430.73	11.75	430.7413	17.5
430.7613	14	430.7738	20.5
430.7938	10.75	430.81	16.5
430.8275	11.25	430.8438	17.25
430.865	9.25	430.8775	15.75
430.8963	14.75	430.9075	24
430.9325	11	430.94	17.5
430.9638	12.75	430.9775	22.5
430.9963	10.5	431.0075	16
431.03	10.5	431.04	18
431.06	10.75	431.0788	14.75
431.0963	10.75	431.1088	16.25
431.1313	11.5	431.1413	17
431.1663	14.25	431.1763	18
431.1988	13.5	431.2075	17.5
431.23	14.25	431.2413	16
431.2613	15.75	431.2738	18
431.2938	13	431.31	18.5
431.3275	14.75	431.3438	14.5
431.3625	13.75	431.3775	16
431.3963	13.5	431.4075	18.25
431.4325	12.25	431.44	13
431.4638	13	431.4775	17.5
431.4963	13	431.5075	16.5
431.53	17.75	431.54	18
431.56	14.5	431.5788	17.5
431.5963	18.25	431.6088	19
431.6313	19.25	431.6413	19
431.665	15	431.6763	17.25
431.6988	15.75	431.7075	19.5
431.73	18.5	431.7413	22.5

431.7613	18	431.7738	17
431.7938	17.75	431.81	16.75
431.8275	16.5	431.8438	21.25
431.8625	15.5	431.8775	20
431.8975	12.25	431.9075	21
431.9325	14.75	431.94	24.75
431.9638	14.75	431.9775	20.75
431.9963	17.25	432.0075	17
432.03	15.75	432.0425	30.5
432.06	12.75	432.0788	26.5
432.0963	13.25	432.1088	16.5
432.1313	13.5	432.1413	19.5
432.1675	12	432.1763	18
432.1988	12.25	432.2088	17.75
432.23	14	432.2413	25
432.2613	8.75	432.2738	21.25
432.2938	15	432.3125	22.75
432.3275	17.75	432.3438	16.5
432.3625	15	432.3775	18
432.3963	12.25	432.4075	17
432.4325	11.75	432.44	16.5
432.4638	15	432.4788	16
432.4963	14.25	432.5075	19.25
432.53	15.75	432.54	16.75
432.56	15	432.5788	18.5
432.5963	12.25	432.6088	14.75
432.63	13.75	432.6413	17
432.665	18.25	432.6763	15.75
432.6988	11.5	432.71	17.5
432.73	16.5	432.7413	20.75
432.7638	17.75	432.7738	17
432.7938	14.25	432.8125	18.5
432.83	15.75	432.8438	18.75
432.8625	15.5	432.875	18.25
432.8988	15.5	432.9075	18.5
432.9325	16.25	432.94	22
432.9663	18.25	432.9775	17.5
432.9963	15.75	433.0075	20.25
433.03	15.75	433.04	16.5
433.06	12.25	433.0788	16
433.0963	15.25	433.1088	17.5
433.13	15.75	433.1413	18.75
433.1625	13.75	433.1763	15.5
433.1988	11.5	433.21	13.25
433.23	14.25	433.2413	15.5
433.2638	12.75	433.2738	17.5
433.2938	13.5	433.3125	12.5
433.33	13	433.3438	17.75
433.3625	14.75	433.375	17.5
433.3988	13	433.4075	18

433.4325	13.5	433.44	18.25
433.4638	16.5	433.4775	17.75
433.4963	15.5	433.5075	16.75
433.53	13	433.54	18.75
433.56	12.5	433.5788	22.75
433.5963	14.5	433.6088	17.5
433.63	13	433.6413	16.5
433.6625	16	433.6763	23
433.6988	15.5	433.71	16
433.73	17.75	433.7413	20.25
433.7638	21.25	433.7738	21.75
433.7938	16	433.8125	23
433.83	15.75	433.8438	26.5
433.8625	19	433.875	27.75
433.8963	14.75	433.91	25
433.9325	14.75	433.94	19.25
433.9638	15.75	433.9775	23.25
433.9963	13.5	434.0075	27
434.03	16.75	434.04	25.75
434.06	17.75	434.0788	26.25
434.0963	15.25	434.1088	29
434.1313	16.5	434.1413	23
434.1625	16.5	434.1763	24
434.1988	16.25	434.21	21.75
434.23	15.25	434.2413	19
434.2638	13.75	434.2738	18.5
434.2938	17	434.31	19
434.33	14.75	434.3438	17.75
434.3625	15	434.375	18.75
434.3963	17.25	434.4075	20.25
434.4325	14	434.445	20.5
434.4638	12	434.4775	21
434.4963	13.25	434.5075	17
434.53	14.5	434.54	20
434.5613	13	434.5788	16
434.5963	13.75	434.6088	15.5
434.63	18.5	434.6413	18
434.6638	15.25	434.6763	21.75
434.6988	15.5	434.71	19.5
434.73	14.75	434.7413	18
434.7638	15.75	434.7738	17.75
434.7938	14.5	434.81	20
434.83	14.5	434.8438	18.25
434.8625	14.25	434.875	19.5
434.8963	17	434.9075	20
434.9325	16.75	434.945	20
434.9638	17	434.9775	25
434.9963	12.5	435.0075	24
435.03	13	435.04	23
435.06	14	435.0788	20.5

435.0963	13.75	435.1088	20.25
435.1313	13.75	435.14	20.25
435.1613	17	435.1763	22.5
435.1988	10.5	435.21	21.75
435.23	10	435.2413	26.5
435.2638	14.5	435.2763	24.25
435.2938	18.5	435.3075	20
435.3275	13	435.3438	23.5
435.3625	15.75	435.375	22.25
435.3963	17.25	435.4075	24.5
435.4313	16.25	435.445	24
435.4638	18.5	435.4775	20.75
435.4975	14.5	435.5075	23
435.53	16.75	435.54	21.5
435.56	17	435.5788	21.75
435.5963	17.5	435.6088	20.75
435.6313	16.75	435.64	20.5
435.6638	19	435.6775	20.25
435.6988	17.25	435.71	16.75
435.73	16.25	435.7413	20.5
435.7638	17	435.7738	20.75
435.7938	14.75	435.8075	16.75
435.8275	15.5	435.8438	17.5
435.8625	16.75	435.875	17.5
435.8963	15.25	435.9075	16
435.9313	14.25	435.945	19
435.9638	13.75	435.9775	16
435.9975	14	436.0075	16.75
436.03	13	436.04	16.5
436.0625	13	436.0788	15.75
436.0963	14.5	436.1088	18
436.1313	10.75	436.14	15.5
436.1638	11.25	436.1788	18
436.1988	11.5	436.21	22
436.23	10.5	436.2413	15.75
436.2638	15.25	436.275	17.75
436.2938	13.5	436.3075	17.25
436.3275	13	436.3438	22.75
436.3625	12	436.375	22.75
436.3963	11.5	436.4075	17.75
436.4288	15.5	436.445	17.5
436.4638	13.5	436.4775	19.25
436.4975	15.25	436.5075	21.25
436.53	16.75	436.54	17.75
436.5625	11.5	436.5788	18.25
436.5963	17.25	436.6088	16.75
436.6313	20	436.64	17.25
436.6638	20.5	436.6788	19.25
436.6988	23	436.71	17.75
436.73	19.5	436.7413	21.5

436.7638	17.25	436.775	20
436.7938	17.5	436.8075	19.25
436.8275	16.75	436.8438	20.5
436.8663	15	436.875	18.5
436.8963	16.25	436.91	22.75
436.9288	15	436.945	19.75
436.9638	14.25	436.9775	22.25
436.9975	21.75	437.0075	19.5
437.03	13.25	437.04	16.5
437.0625	19	437.0788	16.75
437.0963	16.25	437.1088	17.75
437.1313	13.25	437.14	20.5
437.1638	19.75	437.1788	16.25
437.1988	13.75	437.21	15.75
437.23	14	437.2413	21.5
437.2638	15.25	437.275	14.25
437.2938	13.5	437.3075	20
437.3275	15	437.3438	17
437.365	11.75	437.375	15.25
437.3963	17	437.41	16
437.4288	11.75	437.445	16.5
437.4638	13.25	437.4775	16.5
437.4975	13	437.5075	20.75
437.53	16.25	437.54	23
437.5625	15	437.5788	18.5
437.5963	16	437.6088	15.75
437.6313	13.25	437.64	20.75
437.6638	12.75	437.6788	20.75
437.6988	15.25	437.71	18.75
437.7325	16	437.7413	21.25
437.7663	13	437.775	19
437.7938	12.75	437.8075	16.25
437.8275	16.25	437.8438	18.75
437.865	13	437.875	21.5
437.8963	14	437.91	17
437.9288	14.25	437.945	17.25
437.9638	16	437.9775	15.25
437.9975	12.25	438.0075	21
438.03	12.5	438.0425	22.25
438.0625	14.75	438.0788	22.75
438.0963	14.25	438.1088	18.5
438.1313	11	438.1425	22.75
438.1663	13.75	438.1788	16.75
438.2	12.75	438.21	20.25
438.235	9	438.2413	19.75
438.2663	13.5	438.275	22.25
438.2963	11	438.3075	16.5
438.3288	12.75	438.3438	19.5
438.3663	11.25	438.375	18.75
438.3975	9.75	438.41	18.5

438.43	14.5	438.445	20.75
438.465	13.25	438.4775	21
438.4988	14	438.5075	20.75
438.5313	15.5	438.54	21.5
438.565	15.25	438.5788	21.25
438.5988	12.75	438.6088	21
438.6338	14.25	438.6425	24.25
438.6675	11.75	438.6763	19.25
438.7	15.75	438.71	19
438.735	11.25	438.7413	18.25
438.7663	10	438.775	21
438.7963	11.75	438.8075	19.5
438.8288	12	438.8438	22
438.8663	11	438.875	22.5
438.8975	11	438.91	18
438.93	13.25	438.945	17.25
438.965	12	438.9775	16
438.9988	12	439.0075	18.75
439.0313	13.5	439.0425	15.75
439.065	11.75	439.0788	19.75
439.0988	11.25	439.1088	17.5
439.1338	8.25	439.1425	18
439.1638	10.75	439.1763	16
439.2	15.75	439.21	16.5
439.235	14.5	439.2413	16.75
439.2663	14.75	439.275	17.25
439.2963	12.25	439.3075	22.75
439.3288	12.25	439.3438	19
439.3663	12.5	439.3763	21
439.3975	10.25	439.41	17.25
439.43	14	439.445	21.75
439.4638	13	439.4775	18.25
439.4988	12.25	439.51	19.75
439.5313	14.5	439.54	18.75
439.5675	15.5	439.5763	17.5
439.5988	14.25	439.6088	23
439.6338	11	439.6438	18
439.6638	15.25	439.6763	22
439.7	12.5	439.71	21
439.735	14	439.7413	19.5
439.7688	15.5	439.775	20.5
439.7988	16	439.8075	19
439.8313	14.25	439.8463	15.5
439.8688	16.25	439.8763	13.5
439.9	15.75	439.91	18
439.9313	14	439.945	21.75
439.9663	15.25	439.9775	17.75
440.0013	13.25	440.01	20.75
440.0325	12.5	440.04	20.75
440.0663	13.75	440.0763	17.75

440.1	13	440.1088	19.5
440.135	11.75	440.1438	18.25
440.1663	13	440.1763	19
440.2038	11	440.21	22
440.2363	11.25	440.2413	14.5
440.2688	15.25	440.275	16.5
440.2988	15.5	440.31	20
440.3313	16.25	440.3463	22.5
440.3688	14.25	440.3788	16.25
440.4	13.5	440.4125	17
440.4313	12	440.4475	18
440.4663	12	440.4788	18
440.5013	14.5	440.5113	19.5
440.5325	16.75	440.5413	20.75
440.5663	13.5	440.58	18
440.6	13	440.6125	22.25
440.635	12.5	440.645	18.5
440.6663	11.25	440.6775	18
440.7038	10.5	440.7125	21
440.7363	11.25	440.7438	17.25
440.7688	13.75	440.7775	17.25
440.7988	12	440.81	15.5
440.8313	17	440.8463	19.5
440.8725	13	440.8788	16.25
440.9025	13.75	440.9125	18.25
440.9313	15.5	440.9475	23.75
440.9663	13.5	440.9788	18
441.0013	11.5	441.0113	17.75
441.0325	11	441.0413	17.5
441.0663	11.25	441.08	18.25
441.0975	12.75	441.1125	13.25
441.135	13	441.145	17.75
441.1663	13.5	441.1775	16
441.2038	11.25	441.2125	13
441.235	13.5	441.2438	16.75
441.2688	12.75	441.2775	15.5
441.2988	12.5	441.31	16.5
441.3313	16.25	441.3463	18.75
441.3688	14.5	441.3813	15.5
441.4	13	441.4125	13.75
441.4313	11.5	441.4475	16.75
441.4663	13.75	441.4788	16
441.5013	12.25	441.5113	13.75
441.5325	15.25	441.5413	19.5
441.5663	12.75	441.58	16.25
441.5975	12.75	441.6125	16.25
441.635	12.5	441.645	18
441.6663	12.5	441.6775	18.75
441.7013	16.5	441.7125	17.25
441.735	10.5	441.745	17.25

441.7688	12.25	441.7775	16.75
441.7988	14.75	441.81	15
441.8313	12.75	441.8475	16.5
441.8688	14.5	441.8813	16.25
441.9	17	441.9125	16.5
441.9338	16.5	441.945	17
441.9663	14.5	441.9788	14.25
442.0013	13.75	442.0113	13.75
442.0338	13.25	442.0413	17.75
442.0663	11.75	442.0775	15.25
442.0975	14	442.1125	16.25
442.135	8.25	442.145	15.5
442.1663	8.5	442.1775	15.5
442.2013	11.5	442.2125	13.25
442.235	12.5	442.245	16.25
442.2688	14	442.2775	17
442.2988	8.25	442.31	16.75
442.3338	11	442.3475	17.5
442.3688	11	442.3813	13.25
442.4	9.25	442.4125	15.5
442.4338	11	442.445	17.5
442.4663	11	442.4788	17
442.5013	8.5	442.5113	18.25
442.5325	7	442.5413	16.5
442.5663	11	442.5775	14.5
442.5975	9.75	442.6125	19.25
442.635	12.25	442.645	15.5
442.6663	9.75	442.6775	13.5
442.7013	13.25	442.7125	18.25
442.735	10.25	442.745	13
442.7688	10.75	442.7775	15.5
442.7988	10.75	442.81	17.75
442.8313	13	442.8475	14
442.8688	12	442.8813	14.75
442.9	8.75	442.9125	11.75
442.9338	12	442.945	15.75
442.9663	12	442.9788	15.25
443.0013	10.5	443.0113	14.5
443.0325	12	443.0413	16.5
443.0663	14.5	443.0775	14.25
443.0975	12	443.115	12.75
443.135	9.5	443.145	15.25
443.1663	11.25	443.1775	18.25
443.2013	10	443.2125	16
443.235	8.5	443.245	13.25
443.2688	13	443.2775	12.75
443.2988	12.75	443.31	13.5
443.3313	8.25	443.3475	16.75
443.3688	13	443.3813	14.75
443.4	11	443.4125	15.75

443.4338	9.75	443.4475	11.75
443.4663	11	443.4788	12.5
443.4988	10	443.5113	17.5
443.5325	10	443.5413	15.25
443.5638	9.25	443.5788	13.25
443.5975	10.75	443.615	15.5
443.6363	12.25	443.645	15.25
443.6663	11	443.6775	11.75
443.7013	9.75	443.7125	17.25
443.735	12	443.745	15.25
443.7688	9.75	443.7775	18.5
443.7988	9.75	443.81	12.75
443.8338	11.25	443.8475	13.75
443.8688	10.5	443.8813	14.75
443.9	10.5	443.9125	16.75
443.9325	10.5	443.945	15.25
443.9663	9	443.9813	17.75
443.9988	14	444.0113	16.25
444.0325	13.25	444.0413	15.25
444.0638	9	444.0788	20.25
444.0975	11.25	444.115	15.75
444.135	10.25	444.145	18.25
444.1675	6.75	444.1775	16.5
444.2013	11	444.2125	21
444.235	12.75	444.245	18.75
444.2688	9.25	444.2775	15.25
444.2988	12.25	444.31	16
444.3313	12.5	444.3475	15.25
444.3688	11.5	444.3813	15
444.4	10.5	444.4125	17.5
444.4325	10.75	444.445	11.75
444.4663	9.25	444.4813	16.5
444.4988	10	444.5113	12
444.5325	9	444.5413	13.75
444.5638	7.25	444.58	14
444.5975	12.75	444.615	12.5
444.635	9.25	444.645	15.25
444.6675	13.75	444.675	12.75
444.7013	9.5	444.7113	16
444.735	10.5	444.745	10.75
444.7688	12.25	444.7775	13.25
444.7988	6.75	444.81	16
444.8313	8.5	444.8475	14.5
444.8675	11.75	444.8813	12.25
444.9	10.75	444.915	11.5
444.9325	10.25	444.945	14.5
444.965	9	444.9813	15.5
444.9988	9.75	445.0113	15.75
445.0325	13	445.0413	17
445.0638	13.25	445.0788	15.75

445.1	11.75	445.115	17.25
445.135	12.75	445.145	18.5
445.1675	12.25	445.175	18.75
445.2013	12.25	445.2113	15
445.235	12.75	445.245	15.5
445.2663	14.5	445.2775	16.25
445.2988	13	445.31	17.5
445.3313	12.75	445.3488	14.25
445.3675	17.25	445.3813	19.5
445.4	13.5	445.4125	17.25
445.4325	15	445.445	17.75
445.465	12.5	445.4813	19.75
445.4988	11.25	445.5113	19
445.5325	14.75	445.5413	18.5
445.5638	16	445.5788	17.25
445.6	16.25	445.615	22.5
445.635	13.5	445.645	24.5
445.6675	11	445.675	20.5
445.7013	14.5	445.7125	21.25
445.735	11.75	445.745	19.25
445.7663	13	445.7775	20.5
445.7988	14.25	445.8113	17.25
445.8313	12.25	445.8488	19.5
445.8675	11	445.8813	14.5
445.9	11.5	445.9125	20
445.9325	11.5	445.945	21.75
445.965	15	445.9813	18.75
445.9988	13	446.0113	20
446.0325	13	446.0413	17.75
446.0638	12.5	446.0788	19.5
446.1	11	446.115	16.5
446.135	14.25	446.145	16.75
446.1688	9	446.175	14.5
446.2013	10.25	446.2125	15
446.235	11	446.245	20.5
446.2663	10	446.2775	16.25
446.2988	11.25	446.3113	18.75
446.3313	9.75	446.3488	17
446.3675	9.75	446.3813	16.25
446.4025	14.5	446.4125	10.75
446.4325	11	446.445	12
446.465	12.5	446.4813	18.75
446.4988	11.75	446.5113	15
446.5325	10.75	446.5413	20.5
446.5638	8.75	446.5788	17.5
446.6	10.25	446.615	20.75
446.6363	11.25	446.645	13.5
446.6675	11	446.675	19
446.7013	8.75	446.7125	19.75
446.735	12.75	446.745	18.75

446.7675	9.75	446.7775	21
446.7988	9.75	446.8113	19.25
446.8313	13.75	446.8488	16
446.8675	11.5	446.8813	16.25
446.9025	11	446.9125	13
446.9325	12.75	446.945	16.5
446.965	12.25	446.9813	15.25
446.9988	16.75	447.0113	16.75
447.0325	10.75	447.0413	18
447.0638	10	447.0813	21.5
447.1	14	447.115	22.5
447.135	11.25	447.145	20.75
447.1675	13.75	447.175	18.75
447.1988	12	447.2125	20
447.235	13.75	447.245	20
447.2663	14.75	447.2775	16.5
447.2988	13.5	447.31	17.25
447.3313	15.25	447.3488	23
447.3688	13.5	447.3813	19.5
447.4025	16.75	447.4125	20.75
447.4325	12.5	447.445	19.75
447.465	13.25	447.4788	15.75
447.4988	14.25	447.5113	16
447.5325	12.5	447.5413	16.75
447.5638	17.25	447.5813	18
447.6	19.5	447.615	15
447.635	17.25	447.645	15.75
447.67	19	447.675	15.5
447.7013	15.75	447.7125	18.5
447.7363	13	447.745	17.75
447.7675	14.25	447.7775	25.75
447.8	13.5	447.81	17
447.8325	11.25	447.8463	19
447.8725	11.75	447.8813	19.75
447.9063	12	447.9125	16.5
447.9338	14.75	447.9475	20.5
447.9675	14.5	447.9788	20
448.0013	11.25	448.0113	27.75
448.035	11.25	448.0413	21.25
448.0688	11	448.0813	16.5
448.1025	13.75	448.1175	17.75
448.135	14.25	448.145	17.75
448.17	13.5	448.175	17.75
448.2013	13.5	448.2125	15.5
448.2363	13.25	448.245	14.75
448.2675	14	448.2775	16.75
448.3	11	448.31	21
448.3325	12.75	448.3463	19.5
448.3688	12	448.3813	21.75
448.4038	15.5	448.4125	21

448.4338	14.25	448.4475	18
448.4725	14	448.4788	19.75
448.5013	13	448.5138	18.75
448.535	14.25	448.5438	16
448.5688	12.25	448.5813	20.5
448.6025	11.75	448.615	15.25
448.6375	14.25	448.645	20
448.67	11	448.675	18
448.7013	10.5	448.7125	16.5
448.7363	11	448.745	17
448.7675	11.75	448.7775	18.75
448.8	10.25	448.8113	17.5
448.8325	14.25	448.845	19.75
448.8688	10.5	448.8813	15.75
448.9038	14.5	448.9125	18.25
448.9338	16.5	448.9475	18
448.9675	13.25	448.9788	17
449.0013	10.75	449.0138	18.25
449.035	14.5	449.0438	18
449.07	14.25	449.0813	17.5
449.1025	10.75	449.115	20.5
449.1375	15.5	449.145	20
449.17	10.5	449.1775	17
449.2013	14.5	449.2125	17.75
449.2338	10.5	449.245	17.75
449.2675	13.5	449.2775	18.5
449.3	10.75	449.3113	18.75
449.3325	13	449.345	18
449.3688	14	449.3813	20.75
449.4038	14	449.4125	19.25
449.4338	15	449.4463	18.75
449.4675	18.75	449.4788	19.5
449.5013	14	449.5113	18
449.535	12	449.5438	20.75
449.5688	15.25	449.5813	17.75
449.6025	15	449.615	16.75
449.6375	14.75	449.645	20
449.6713	12.25	449.6775	19
449.7013	13.25	449.7125	20.25
449.7338	16.25	449.745	20
449.7675	10.5	449.7775	19
449.8	13.25	449.8113	18.75
449.835	15	449.8463	17.75
449.8688	14.25	449.8838	18
449.9038	12.5	449.915	17.75
449.9338	13.5	449.9488	15.75
449.9675	12.5	449.98	16
450.0013	13	450.0125	16
450.035	12.5	450.045	18.75
450.0663	11.75	450.0838	17.5

450.1025	9.5	450.1163	19
450.1375	11.25	450.1463	16.25
450.17	10.5	450.1788	16.5
450.2013	11.25	450.215	21.5
450.2338	13.5	450.2463	17.5
450.2675	11.25	450.28	16.25
450.3	12.75	450.315	19.5
450.335	9.25	450.3488	22.25
450.3688	9.75	450.3838	16.75
450.4025	10.75	450.415	16.25
450.435	10.75	450.4488	20
450.47	9	450.48	20
450.5038	10.75	450.5125	18.75
450.5363	9.75	450.5475	17.75
450.5675	11.75	450.5838	19.75
450.6038	13.5	450.6163	17.25
450.64	13	450.6463	19.5
450.6725	13.25	450.6788	16.5
450.705	11	450.715	18.25
450.735	12	450.7463	19.25
450.77	12.25	450.78	17
450.8025	7.5	450.8138	22.75
450.84	12.75	450.8488	16.75
450.8713	11.25	450.8838	18.25
450.9038	9.75	450.915	16.5
450.935	9.5	450.9488	18.25
450.97	11.75	450.98	18
451.0038	9	451.0125	14.25
451.0363	10	451.0475	15.25
451.0675	10.5	451.0838	17.5
451.105	10.75	451.1163	17.75
451.14	10.25	451.1463	16.5
451.1725	12.75	451.1788	12.25
451.205	13	451.2125	13.25
451.235	13.75	451.2463	17.25
451.27	12	451.2813	14.75
451.3025	16.25	451.3138	15.5
451.3375	16	451.3488	15.25
451.3713	13.5	451.3838	16
451.4038	12.25	451.415	13
451.435	13.25	451.4488	16
451.47	14.5	451.48	12
451.5038	11.25	451.5125	17
451.5363	11	451.5475	14
451.5675	12	451.5838	15.25
451.605	10.5	451.6163	13.75
451.64	9.75	451.6463	16
451.6725	12.25	451.6788	14.5
451.705	14.25	451.7125	15
451.735	11.25	451.7463	17

451.77	12.5	451.7813	18
451.8025	13.5	451.8138	15.5
451.8375	14.75	451.8513	19.75
451.8725	11	451.8838	15.25
451.905	15.25	451.9163	19.5
451.935	15	451.9488	21.5
451.97	11.75	451.98	17.5
452.0038	13.75	452.0125	16.5
452.0363	13.25	452.0475	19
452.0675	11	452.0838	24.5
452.105	14	452.1163	14.75
452.14	12.5	452.1463	15
452.1738	12.25	452.1788	11.25
452.205	11.75	452.2125	19.25
452.235	12.25	452.2463	12.75
452.27	11	452.2813	15.25
452.3025	12	452.3138	17.5
452.3375	10.75	452.3463	17.5
452.3725	8.75	452.3838	11.5
452.4038	10	452.4163	17.25
452.435	12.75	452.4488	16.25
452.4725	9.25	452.48	27.5
452.5025	11.75	452.5125	24.5
452.5363	9.75	452.5475	15.5
452.5675	11.25	452.5838	19.5
452.6063	11.5	452.6163	18.25
452.6425	16	452.6463	20.75
452.6725	11.5	452.6788	17.5
452.705	10.5	452.7125	15.5
452.735	12.25	452.7463	14.5
452.77	12	452.7813	15.25
452.8025	11.5	452.8138	21.5
452.84	14.25	452.8463	20
452.8725	12.5	452.8838	16.25
452.9038	13	452.9163	16.25
452.935	15	452.9488	19.75
452.97	12.75	452.9788	17.5
453.0025	13.25	453.01	17
453.0363	11.75	453.0475	15.5
453.0675	14	453.0838	17.5
453.105	10	453.1163	17
453.14	12	453.1463	18.75
453.1725	16.25	453.1788	21.5
453.205	13.5	453.2125	18.25
453.235	9.75	453.2463	17
453.27	8.75	453.2813	19.25
453.3025	10.25	453.3138	21
453.3375	8	453.3463	21.25
453.3725	9.75	453.3838	16.75
453.4038	10	453.4163	17.25

453.435	12	453.4488	15.75
453.47	10	453.4813	17.25
453.5025	11.25	453.51	22.25
453.5363	8.75	453.5475	18.75
453.5675	10.75	453.5838	22
453.605	13.5	453.615	18.25
453.64	8.75	453.6463	18.5
453.67	13.25	453.6788	18.25
453.7063	8	453.7125	24
453.735	12.25	453.7463	18.75
453.77	11.25	453.7825	27.25
453.8025	9	453.8138	21.25
453.8375	8.75	453.8463	19.5
453.8725	11	453.8838	14.5
453.9038	11	453.9163	23.25
453.935	10	453.9488	22.75
453.97	9.25	453.9788	20.75
454.0025	7.5	454.01	20
454.0363	7.25	454.0475	18
454.0675	8.75	454.0838	18.25
454.1075	12.75	454.115	20
454.14	11.5	454.1463	20.25
454.17	12	454.1788	18.5
454.205	14.5	454.2125	22
454.235	13.75	454.2438	19.25
454.27	13.5	454.2825	22.75
454.3025	15	454.315	21.25
454.3375	12.5	454.3463	20.25
454.3725	16.5	454.3838	18.75
454.4038	11.5	454.4163	25.5
454.435	13	454.4488	24.25
454.47	13.5	454.4788	17.75
454.5025	13.5	454.5125	20.75
454.5363	13.75	454.5475	18
454.5675	12.75	454.5838	17.25
454.6075	14.25	454.615	19.25
454.6425	13	454.6463	16
454.67	12.5	454.6788	20
454.705	11.75	454.7125	21.75
454.735	14.5	454.7438	19
454.7738	10.75	454.7838	20.75
454.8025	10.25	454.815	18.25
454.8375	10.5	454.8463	16.75
454.8725	12	454.8838	22
454.9038	9.75	454.9163	19
454.935	10	454.9488	14.25
454.97	16.25	454.9788	17.25
455.0025	10.5	455.0125	14.75
455.0363	12	455.0475	18.5
455.0675	11.25	455.0838	17

455.1075	12.25	455.1138	15.25
455.14	11.5	455.1488	15.25
455.1713	11	455.1788	16.25
455.205	12	455.2125	16.75
455.2363	15.75	455.2438	17.75
455.2738	12	455.2825	22
455.3038	14.5	455.315	16.75
455.3413	11	455.3463	18
455.3738	9	455.3838	17
455.405	11	455.4163	15.5
455.4375	14	455.4488	19.75
455.4725	10	455.48	15.5
455.505	13.5	455.5138	21.5
455.5388	12.5	455.5513	19.75
455.57	11.75	455.585	17.25
455.61	15	455.6163	17.75
455.645	16	455.6513	16.25
455.6725	13.5	455.6813	19
455.7063	13.5	455.715	19
455.7363	9.5	455.7463	18.5
455.7763	14	455.7825	21
455.8038	12	455.82	15.5
455.8425	11.5	455.8488	19.25
455.8738	11.5	455.885	19
455.905	14.5	455.9175	21
455.9375	12.5	455.95	24.75
455.9725	16	455.98	18.25
456.005	13	456.0138	24
456.0388	13.25	456.0513	20.75
456.0713	12	456.085	21
456.11	12.5	456.1163	24.75
456.1425	12.5	456.1513	27.5
456.1725	17.75	456.1813	21
456.2063	15	456.215	16
456.24	14.5	456.2463	20.25
456.2738	15.25	456.2825	24.75
456.3038	16	456.3175	17.5
456.3425	12.25	456.3488	21.75
456.3738	13.5	456.385	22
456.405	16	456.4175	17.25
456.4375	8.5	456.45	22.75
456.4725	14.5	456.48	19
456.505	12.75	456.5138	19.25
456.5388	13.75	456.5513	20.75
456.57	10.75	456.585	27.75
456.61	11.25	456.6163	23
456.6425	14.5	456.6513	20.5
456.6725	12.25	456.6813	19
456.7063	10.75	456.715	22
456.7363	13.25	456.7463	18.75

456.7738	14.75	456.7825	19
456.8038	13.75	456.8175	16
456.8475	12.5	456.8488	20.75
456.8738	12	456.885	18.5
456.905	12.5	456.9175	18.25
456.94	13.25	456.95	17.25
456.9725	12.25	456.98	22
457.005	13.25	457.0138	20
457.0388	15.25	457.0513	21.5
457.07	13.25	457.085	19.5
457.11	10.75	457.1163	15.75
457.1425	14.25	457.1513	22
457.1725	11.5	457.1813	21.25
457.2063	9.25	457.215	19.75
457.2363	12	457.2488	20
457.2713	7.5	457.28	16
457.3038	10.75	457.32	24
457.3475	8.5	457.3513	15
457.3738	10.5	457.3838	19.75
457.405	12.25	457.4188	18.75
457.4388	13.5	457.4513	17.5
457.4725	14	457.4825	14.25
457.505	12.75	457.5175	16.75
457.5388	14	457.55	15.25
457.575	13.75	457.5863	16
457.61	10.5	457.6188	14.75
457.6425	16	457.6538	13.75
457.6725	13	457.6838	17
457.7063	14.5	457.7175	15.5
457.7363	16.75	457.75	17
457.7713	18	457.7825	14.5
457.8038	15.75	457.82	15.75
457.8425	11.5	457.8513	13.25
457.8738	14	457.8838	15.5
457.905	13.5	457.9188	13.75
457.9425	17.25	457.9513	15.5
457.9725	12	457.9825	18.75
458.005	15.25	458.0175	16.25
458.0388	15	458.05	14.75
458.075	12.5	458.0863	16.25
458.11	15	458.1188	14.25
458.1425	14	458.1538	18.75
458.1725	15.5	458.1838	15.5
458.2063	13	458.2175	15
458.2363	16.5	458.25	15.5
458.2713	17.5	458.2825	15.75
458.3038	14.75	458.32	17.75
458.3425	13.25	458.3513	17.75
458.3738	17	458.385	16.25
458.405	14.75	458.4188	16.5

458.4388	16	458.4513	15.25
458.4725	15.5	458.4825	15.75
458.5075	18.25	458.5175	17
458.54	14.5	458.5488	17.75
458.575	13.25	458.5863	15.5
458.61	14.75	458.6188	20.25
458.6425	19.5	458.6538	18.5
458.6725	10.25	458.6838	16.25
458.7063	12.75	458.7175	17.75
458.7363	14.25	458.75	18.25
458.7713	12	458.785	18.25
458.8038	12.25	458.82	14.75
458.8425	11.5	458.8513	14.25
458.875	13	458.885	15.25
458.9075	14.5	458.9188	14.5
458.9425	17	458.9513	11.5
458.975	12	458.9825	14.25
459.0088	12.25	459.0175	12
459.04	11	459.0488	13
459.0763	16	459.0863	16
459.1125	13.25	459.1188	14.75
459.1463	13.25	459.1538	12.25
459.1763	16.75	459.1838	18.25
459.2075	15.75	459.2175	17.75
459.2388	18.75	459.25	16.5
459.2738	16.75	459.285	19
459.3075	17.75	459.32	14.75
459.345	13.5	459.3513	17.5
459.375	12.75	459.385	15.5
459.4063	17	459.4188	14.5
459.4425	13.5	459.4513	15.25
459.475	18.75	459.4825	18.25
459.5088	16.25	459.5175	16.75
459.54	16	459.5488	14.75
459.5763	15.75	459.5863	14.75
459.6125	19.5	459.6188	15.75
459.645	15.75	459.6525	19.5
459.6763	20.5	459.6838	16.5
459.7125	20.25	459.7175	13.25
459.7388	15.75	459.75	16.25
459.7738	17.25	459.785	19.5
459.8075	15	459.8175	14.5
459.8463	16	459.8513	17
459.875	17	459.885	19.5
459.9063	14.5	459.9213	15
459.9425	21	459.9513	19.5
459.975	16.75	459.9825	13.75
460.0088	10	460.0175	15
460.04	16.75	460.0488	17.25
460.0763	15	460.0863	14.75

460.1125	14.75	460.1188	15.5
460.145	13.25	460.1525	14.75
460.1763	17.5	460.1838	17.75
460.2075	14.25	460.2175	17.75
460.2388	21.5	460.25	19.75
460.2738	18.25	460.285	18.75
460.3075	16	460.315	14.5
460.345	15.75	460.3513	15.25
460.375	15	460.385	16.5
460.4063	16.75	460.4188	14.25
460.4425	11.5	460.4513	13.25
460.475	15	460.4838	11.25
460.5088	15.75	460.5175	17.25
460.54	15.75	460.5488	11.25
460.5763	14.5	460.5863	16
460.6125	15.75	460.6188	14.25
460.645	18.5	460.6538	13.5
460.6763	16.25	460.6838	13.25
460.7075	14.25	460.7175	14
460.7388	18.25	460.75	13.5
460.7738	18.75	460.785	14
460.8088	15	460.815	14.75
460.845	16.25	460.8513	17
460.875	16.25	460.885	16.75
460.9063	15.75	460.9188	14.75
460.94	15	460.9513	18.75
460.975	13.5	460.9838	15.5
461.0088	10.25	461.0175	19.25
461.0425	13.75	461.0488	19
461.0788	14	461.0863	17.75
461.1125	13	461.1188	17.25
461.145	12.75	461.1525	16.5
461.1763	11.5	461.1838	16.25
461.2075	9.75	461.2175	16.25
461.2388	13.25	461.25	16.5
461.2738	12.5	461.285	18.75
461.3075	12.25	461.315	15.25
461.345	15.25	461.3513	15
461.375	13.75	461.385	16.25
461.4088	11.5	461.4188	17.25
461.44	12.5	461.4513	14
461.475	14.25	461.4838	16
461.5088	10.5	461.5188	15
461.5425	11.75	461.5488	15
461.5788	11.75	461.5863	15.25
461.6125	12.5	461.6188	15.25
461.645	15	461.6525	16.5
461.6763	12.75	461.6838	15.75
461.7075	11.25	461.7175	18
461.7388	12	461.75	17

461.7738	10.5	461.785	13
461.8075	11.75	461.815	14.25
461.845	11.25	461.8513	17
461.875	11.25	461.885	19
461.9088	11.5	461.9163	17.5
461.94	9.5	461.9513	14.75
461.975	10	461.9838	16.75
462.0088	10	462.0188	15.75
462.0425	15.75	462.0488	11.75
462.0788	13	462.0863	13.75
462.1125	12.5	462.1213	14
462.145	14	462.1525	14.5
462.1763	13	462.1838	17
462.2075	11.75	462.2175	14.5
462.2388	12.75	462.25	14.5
462.275	13.75	462.285	14.75
462.3075	14.25	462.315	15.25
462.345	13	462.3513	18
462.3775	15.25	462.385	15.75
462.4088	11.75	462.4163	14
462.44	21.5	462.4538	18.75
462.475	13.75	462.4838	15.25
462.5088	12.5	462.5188	14.25
462.5425	16.25	462.5488	14
462.5788	14.5	462.5825	14
462.6163	14.5	462.6213	11
462.645	15.5	462.6525	15.25
462.6763	13.75	462.6838	15.75
462.7075	13.5	462.7175	13.25
462.7388	11.5	462.75	13
462.775	10.25	462.785	19
462.8075	13	462.815	21
462.845	12.5	462.8513	13.25
462.875	12.75	462.885	21.75
462.9088	14.5	462.9163	18.75
462.94	12	462.9513	20.5
462.975	14	462.9838	19.25
463.0088	14	463.0188	16.5
463.0425	12.25	463.0488	17.25
463.0788	14.25	463.0825	16.75
463.1125	12.5	463.1213	19.5
463.1463	10.75	463.1525	19.75
463.1763	12.5	463.1838	19.5
463.21	12.25	463.2175	17.5
463.2388	12.25	463.2525	14.75
463.275	11.5	463.285	23.5
463.3075	12	463.315	17.75
463.3425	11	463.3513	16.25
463.375	15.5	463.385	16.75
463.4088	14.5	463.4163	10.25

463.44	13.75	463.4513	18.25
463.475	14.25	463.4838	14.5
463.5088	15	463.5188	20.25
463.5425	13.75	463.5488	15
463.5788	18.25	463.5825	20
463.6125	15.5	463.6213	17.5
463.6463	15.25	463.6525	14
463.6763	14.5	463.6838	17.75
463.7075	15.75	463.7175	18
463.7388	16.25	463.7525	16.75
463.775	15.5	463.785	17.25
463.81	14	463.815	18.75
463.8425	12	463.8513	19
463.875	15.5	463.885	13.25
463.9088	14.75	463.9163	11.75
463.94	12.25	463.9513	14.75
463.975	15.25	463.9838	17.25
464.0113	13.5	464.0188	15.5
464.0425	11.75	464.0488	16.5
464.0813	12.25	464.0825	19
464.1125	14.75	464.1213	20
464.1463	13	464.1525	19.5
464.1763	14.5	464.1825	17.5
464.2063	16.25	464.2175	21.75
464.2413	15.75	464.2525	18.75
464.2763	16	464.285	12.25
464.31	13.5	464.315	13.25
464.3425	12.25	464.3513	19.25
464.375	15	464.385	11.5
464.4088	12.25	464.4163	12.5
464.44	16	464.4513	16
464.475	15.75	464.4838	14
464.5113	15.5	464.5188	13.75
464.5425	11	464.5488	17.5
464.5788	13	464.5825	13
464.6125	12.75	464.6213	11.5
464.6463	14.5	464.6525	13.5
464.6763	13.75	464.6825	14.75
464.7063	13.75	464.72	14.75
464.7388	17.25	464.7525	14.25
464.775	12.75	464.785	15
464.81	15.75	464.815	16
464.8425	17	464.85	12.25
464.8788	16	464.885	15.5
464.9088	15	464.9163	16.75
464.94	14.5	464.9513	14
464.975	14.5	464.9838	13.5
465.0113	13.5	465.0188	13
465.0425	17.25	465.0488	19
465.0788	15.5	465.0825	16.25

465.1125	16.25	465.1213	20
465.1463	13.25	465.1525	19.5
465.1763	15.25	465.1825	22
465.2063	14.25	465.22	18.5
465.2413	16.5	465.2525	19.25
465.275	18.75	465.285	19.25
465.31	17.25	465.315	19.25
465.3425	17.5	465.3513	20
465.3775	15.75	465.385	20
465.4088	17.75	465.4163	20.75
465.4425	13	465.4513	19
465.475	17.5	465.4863	17.5
465.5113	15.25	465.5188	19.25
465.5425	16.25	465.5488	24.75
465.5788	15.25	465.5825	18.25
465.6125	14	465.6213	20.5
465.6463	12	465.6525	19.5
465.6763	13.25	465.6825	21.5
465.7063	13.75	465.72	17.75
465.7413	15.5	465.7525	17.5
465.775	17.25	465.785	15
465.81	17.75	465.815	22.5
465.8425	17	465.8513	22
465.8775	12.25	465.885	17.75
465.9088	18.25	465.9163	20.5
465.94	17.25	465.9488	15.75
465.975	16.25	465.9863	24.5
466.0113	17	466.0188	21.25
466.0425	17	466.0488	17.25
466.0788	12.75	466.0825	18.75
466.1125	17.25	466.1225	19.75
466.1463	16.75	466.1525	16.75
466.1763	15.5	466.1825	17.25
466.2063	18	466.22	15
466.2413	15	466.2525	16
466.275	16	466.285	19.75
466.31	16.5	466.315	19
466.3425	17.75	466.3513	14.5
466.3775	13.5	466.385	17.5
466.4088	12.25	466.4163	17.75
466.44	16.75	466.4488	21.75
466.4725	18.75	466.4863	20.75
466.5113	13.5	466.5188	19.5
466.5425	14	466.5488	21.75
466.5788	15.75	466.5825	13.75
466.6125	15.25	466.6225	18.5
466.6463	17.25	466.6525	16
466.6763	19.75	466.6825	19.75
466.7063	18.25	466.72	20.5
466.7413	20.25	466.7525	19.25

466.775	17	466.785	17.5
466.81	14.5	466.815	21
466.8425	15.75	466.8513	21
466.8775	17.25	466.885	18.75
466.9088	19.5	466.9163	18.75
466.94	15.5	466.9488	17.25
466.9738	14.75	466.9863	18.75
467.0088	16	467.0188	19.5
467.0438	13	467.0488	18.75
467.0788	15.75	467.0825	22.5
467.11	18.75	467.1225	21.25
467.1463	15.5	467.1525	22.25
467.1763	15.75	467.1825	17.25
467.2063	12.5	467.22	16.25
467.2413	16.5	467.2525	19.5
467.2775	19.5	467.285	18.75
467.31	20.75	467.315	21.5
467.3425	17.5	467.3513	20.75
467.3775	17.5	467.385	19.75
467.4088	19	467.4163	18.25
467.44	15.75	467.4488	19
467.4738	21.25	467.4863	16.75
467.5088	17.75	467.5188	17.5
467.5438	14.75	467.5488	19.25
467.5788	13	467.5825	20.75
467.61	17.5	467.6225	17
467.6463	18.5	467.6525	17.75
467.6763	21.25	467.6825	18
467.7063	16.25	467.7188	15.25
467.7413	11.25	467.7525	19.25
467.7775	14.25	467.785	19.75
467.81	18	467.815	21.25
467.8425	17.5	467.8513	11
467.8775	14.5	467.885	14.25
467.9088	13.75	467.9163	17.75
467.94	21.75	467.95	17.25
467.9738	16.25	467.9863	16
468.0088	12.75	468.0188	16
468.0438	15	468.0488	18
468.0763	13.5	468.0825	18.25
468.1125	15.25	468.12	16
468.1463	14.25	468.155	16.5
468.1763	13.25	468.1825	19
468.2063	15.75	468.2188	18.75
468.2413	13.75	468.2525	19.75
468.2775	17.75	468.285	20.75
468.31	19	468.315	17.25
468.3425	15.25	468.3513	16
468.3775	15.25	468.385	21.5
468.4088	15.5	468.4163	18.75

468.44	14.75	468.45	20
468.4738	14.5	468.4863	18
468.5088	14.75	468.5188	21.75
468.5438	12.5	468.5488	16.25
468.5763	18	468.585	19.5
468.6125	19	468.62	21.75
468.6463	12.75	468.6525	22.25
468.6763	16	468.6825	20
468.7088	18.75	468.7188	19.25
468.7413	13.75	468.7525	17.5
468.7775	14.75	468.785	23.75
468.81	13	468.815	17.5
468.8425	14.5	468.8513	18
468.8775	10.5	468.885	16.25
468.9088	13	468.9163	18.5
468.94	15.75	468.95	19.25
468.9738	12.75	468.9863	18.25
469.0088	14.75	469.0188	24.25
469.0438	13.25	469.0488	18.5
469.0763	16.25	469.0863	15.75
469.1125	12.5	469.12	16
469.1463	16.75	469.1525	19
469.1763	15.25	469.1825	15.5
469.2088	15	469.2188	16.25
469.2388	16	469.2525	17
469.28	14.25	469.285	19
469.31	11.75	469.315	14
469.3425	14	469.3513	14.5
469.38	16.75	469.385	19.25
469.4088	16	469.4163	15
469.44	18.75	469.45	16
469.4738	16.75	469.4863	15.25
469.5088	15.5	469.5188	20
469.5438	15.25	469.5488	13.25
469.5763	18.75	469.585	17.25
469.6125	15.75	469.62	14
469.6463	14.75	469.6525	18.25
469.6763	17.25	469.6825	17
469.7088	15.5	469.7188	19.5
469.7388	18.5	469.7525	16
469.78	13.25	469.785	15.25
469.81	14.75	469.815	18.75
469.8425	15.25	469.8513	19
469.8775	13.75	469.885	16
469.9088	12.75	469.9163	18.75
469.94	15	469.9525	21.25
469.9738	14.5	469.985	19.25
470.0088	18.5	470.0188	18.25
470.0438	11.75	470.0488	18.25
470.0763	14.25	470.085	18.25

470.1125	13.25	470.12	18.25
470.1475	13.75	470.1525	17.5
470.1763	14	470.1825	18.5
470.2088	12.75	470.2188	22.75
470.2388	12	470.2525	19
470.2775	11.75	470.285	19.25
470.31	14.5	470.315	22.5
470.3425	11.5	470.3513	18.25
470.3775	11.75	470.385	24.25
470.4088	16.75	470.4163	24
470.4413	15.25	470.4525	23
470.4738	15	470.485	21.75
470.5088	14.5	470.5188	21.25
470.5438	9	470.5488	19.5
470.5763	12	470.585	21
470.6125	12.25	470.62	20
470.6463	10.25	470.6525	21.25
470.6763	10.5	470.6825	19
470.7088	16	470.7188	21
470.7413	12.5	470.7525	18
470.7775	18	470.785	19.75
470.81	14.5	470.815	19.75
470.8425	11.25	470.8513	19
470.8775	11.75	470.885	20.25
470.9088	12.75	470.9163	20
470.9413	13	470.9525	21.75
470.9738	13.5	470.985	14
471.01	13.25	471.0188	14.5
471.0438	12	471.0488	18
471.0763	13	471.085	16.75
471.1125	14.5	471.12	20.25
471.1463	13.5	471.1525	17.25
471.1763	17.25	471.1825	17
471.2088	13	471.2188	22
471.2388	14.75	471.2525	18.5
471.2775	16.25	471.285	22.75
471.31	13.5	471.315	20
471.345	10.75	471.3513	19.25
471.3775	9	471.385	21.25
471.4088	10.25	471.4163	21.75
471.4413	10	471.4525	23.75
471.4738	9.75	471.485	20
471.51	8	471.5188	21
471.5438	17.25	471.5513	17
471.5763	8.75	471.585	19.75
471.6125	10.25	471.62	21.25
471.6463	10.75	471.6525	20.75
471.6763	9.25	471.685	22.5
471.7088	9.5	471.7188	20.5
471.7388	13.5	471.7525	16.25

471.7775	12	471.7863	17.25
471.81	11.75	471.8175	16.5
471.8425	9	471.8513	20.5
471.8775	8.75	471.885	21.5
471.9088	12.25	471.9163	18
471.9438	11.75	471.9525	15
471.9738	11	471.985	24
472.01	9.25	472.0188	20.25
472.0438	11.25	472.0488	16.25
472.0763	8.25	472.085	16.25
472.1125	9.75	472.12	18.25
472.1463	11.5	472.1525	18.75
472.1788	9.75	472.1863	18.25
472.2088	10.75	472.2188	15
472.2413	12.25	472.25	16.5
472.2775	8	472.285	18.25
472.31	10.5	472.3175	19
472.3413	7.75	472.3513	18.25
472.3775	10.75	472.3838	13.75
472.4088	11.5	472.4163	18.5
472.4413	9.25	472.4525	19
472.4738	8.5	472.485	17.5
472.5088	9.75	472.5188	19
472.5425	12.25	472.5488	19
472.5763	11	472.585	16.25
472.6125	12	472.62	16.5
472.6463	12.75	472.6525	17.75
472.6788	12.25	472.6863	20.5
472.7088	12.5	472.7188	14
472.7388	9.5	472.75	16.25
472.7775	13	472.785	23
472.81	10.5	472.8175	26.5
472.8413	12.75	472.8513	23.25
472.8775	10.5	472.8838	19.25
472.9088	11.25	472.9163	17
472.9413	8.25	472.9525	19.5
472.9738	10.25	472.985	16.75
473.0088	12.5	473.0188	16.5
473.045	9.75	473.0488	20.25
473.0788	13.25	473.085	15.75
473.1125	10.5	473.12	15.5
473.1463	10.75	473.1525	21.75
473.1788	9	473.1863	18
473.2088	13.75	473.2188	13.25
473.2413	13	473.25	17
473.2775	11.75	473.285	18
473.31	13.25	473.3175	17.75
473.3413	12	473.3538	17.25
473.3775	13.5	473.3838	16.25
473.4088	9.5	473.4163	12.25

473.4413	16.25	473.4525	15
473.4738	13.25	473.485	16
473.5088	10.5	473.5188	14.5
473.5425	11.75	473.55	13.75
473.5763	11	473.585	13
473.6125	12.25	473.62	14.5
473.6463	14.5	473.6525	17.75
473.6788	13.25	473.6863	16.25
473.7088	12.25	473.7188	11.75
473.7413	13.5	473.75	16.75
473.7775	9.75	473.785	13.5
473.81	12.5	473.8175	15.5
473.8413	13.75	473.8538	16.75
473.8775	15	473.8838	15.5
473.91	12.25	473.9163	14.75
473.9413	10.25	473.9525	22.75
473.9738	9	473.985	16.25
474.01	13	474.0175	19.5
474.0425	12.75	474.05	17
474.0763	11.25	474.085	15
474.1125	13.25	474.12	18.75
474.1463	16	474.1525	15.75
474.1788	11.25	474.1863	16.75
474.2088	10	474.2188	18.5
474.2413	12.5	474.25	15
474.2775	14.25	474.285	13.75
474.31	12.5	474.3175	14.75
474.3425	12	474.3538	16
474.38	13.75	474.3838	14.25
474.4125	15	474.4163	11.5
474.4438	10.25	474.4525	11.25
474.4788	14	474.485	11
474.5163	19.75	474.5175	15
474.5463	14.75	474.55	14
474.5813	15	474.585	16.5
474.6138	13.75	474.62	12.25
474.6488	15.25	474.6525	13
474.6813	15	474.6863	16.75
474.7113	16.25	474.7188	19.25
474.745	18.25	474.75	13
474.7813	17	474.785	18.5
474.8125	15.5	474.8175	16.25
474.845	14.5	474.8538	21.75
474.8825	16.25	474.8838	15.75
474.915	14.25	474.9188	16.5
474.9463	16.25	474.9525	14.5
474.98	14.75	474.985	20.25
475.0163	15.5	475.0175	16
475.0463	13.75	475.05	15.75
475.0813	15.25	475.085	16.25

475.1138	11.5	475.12	20.5
475.1488	10.5	475.1525	15
475.1813	13.25	475.1863	14.25
475.2113	9.25	475.2188	16.5
475.245	10.5	475.25	17
475.2813	10.5	475.2875	16.25
475.3125	8.75	475.3175	16.75
475.345	7.75	475.3538	19
475.3825	12.25	475.3838	17.5
475.415	12	475.4188	16.75
475.4463	11.75	475.4525	18.25
475.48	10	475.485	11.5
475.5138	11.75	475.5175	14
475.5463	8.5	475.55	15.5
475.5813	12.25	475.585	17.5
475.6113	11.75	475.62	16.25
475.6513	11.25	475.6525	20
475.6813	11.5	475.6863	21.25
475.7113	11.25	475.7188	18.75
475.745	10.75	475.75	17.75
475.7813	8.5	475.7875	17
475.8113	12.25	475.8175	14
475.845	14	475.8538	14
475.8825	13.75	475.8838	16.5
475.915	12.25	475.9188	16
475.9463	10.5	475.9525	12.25
475.98	8.25	475.985	14.5
476.0138	6.75	476.0175	15.75
476.0463	11	476.05	19
476.0813	8.25	476.085	13
476.1113	10.75	476.12	16.75
476.1513	11.25	476.1525	19.75
476.1813	10.75	476.1863	20.25
476.2113	11.75	476.2188	14.75
476.245	9.75	476.25	16.5
476.2813	11.25	476.285	16.5
476.3113	11.5	476.3175	16.75
476.345	12.25	476.355	17.5
476.3825	9.5	476.3875	16.75
476.415	14.25	476.4213	18.25
476.4463	12	476.4538	17.5
476.4813	12	476.4863	15
476.5138	9.75	476.52	18.75
476.5463	9.5	476.5513	17.75
476.5813	13.5	476.5875	19.5
476.615	14.25	476.6225	14.25
476.655	11.25	476.6538	13.75
476.6825	11.75	476.6888	15.25
476.715	11.75	476.7213	11.5
476.7475	11.25	476.7525	13.25

476.7838	6	476.7875	15.75
476.8138	10	476.82	12.25
476.8475	9	476.855	11.75
476.885	6.75	476.8875	13.5
476.9175	9.75	476.9213	15.25
476.9488	7.25	476.9538	12.5
476.9825	9.5	476.9863	13.5
477.015	8.75	477.02	13.75
477.0475	11.5	477.0513	11.75
477.08	10.25	477.09	14.25
477.115	12.5	477.1225	15.25
477.1525	10.75	477.155	12.25
477.1838	14.75	477.1888	12
477.215	9.5	477.2213	12
477.25	11.75	477.2525	12.5
477.2838	9.75	477.2875	16.5
477.3138	13.75	477.32	15.5
477.3475	12.75	477.355	14.25
477.385	11.75	477.3875	16.25
477.4175	10.5	477.4213	18.25
477.4488	14	477.4538	18.5
477.4825	12.75	477.4863	21.25
477.515	12	477.52	21
477.5475	14.5	477.5513	18
477.58	9.5	477.59	19.5
477.615	9.75	477.6225	21.5
477.6525	13.5	477.655	18.75
477.6825	11	477.6888	22
477.715	8.25	477.7238	17.75
477.75	11	477.7525	19.5
477.7838	7	477.7875	16.75
477.8138	11	477.82	20.5
477.8475	9	477.855	18
477.885	11	477.8875	17.5
477.9175	11.5	477.9213	21
477.9488	10.25	477.9538	19.25
477.9825	9.75	477.9863	19.75
478.015	10	478.02	19.25
478.0475	15.25	478.0513	19.5
478.08	12	478.09	19
478.115	11.75	478.1225	18.25
478.1525	9.5	478.155	20.75
478.1825	10.75	478.1888	14
478.215	12.25	478.2213	15.5
478.25	11.5	478.255	14.75
478.2838	10.75	478.2875	17.75
478.3138	7.5	478.32	13.5
478.35	8	478.355	16
478.3863	10.75	478.3875	15.25
478.4175	10	478.4213	15.5

478.45	11.75	478.4538	19.75
478.4825	9.5	478.4863	14.5
478.515	9.5	478.52	19.5
478.5475	10.75	478.5513	19
478.58	12.75	478.59	13
478.615	10.25	478.6225	11.5
478.6525	10	478.655	16.5
478.6825	9	478.6888	12.75
478.715	10.5	478.7213	15
478.75	6.75	478.755	14.5
478.7838	9.5	478.7875	14.25
478.8138	7.25	478.82	16
478.8488	9.5	478.855	18.25
478.8863	6.25	478.8875	15.25
478.9175	7	478.9213	16.75
478.95	7.5	478.9563	14.5
478.9825	10	478.9888	19.5
479.015	10.75	479.02	15.5
479.0475	9.75	479.05	14
479.08	10	479.09	14.75
479.115	10.75	479.1225	17.25
479.1525	10	479.155	19.5
479.1825	8.75	479.1888	16.5
479.215	10	479.2213	19.5
479.25	12	479.255	18
479.2825	10	479.2875	17.5
479.3138	9.5	479.3225	16
479.3488	12	479.355	14.25
479.3838	10.5	479.3875	13.75
479.4175	12	479.4213	17.25
479.45	13.75	479.4563	18.5
479.4838	13.25	479.49	16.75
479.515	9.5	479.5225	14.75
479.5475	11.75	479.555	20.75
479.58	12	479.5925	17.5
479.615	8	479.625	19.5
479.6525	14.25	479.6575	13
479.6825	11.25	479.6913	17.75
479.715	10.25	479.7225	15
479.75	13.25	479.7563	21.5
479.7825	12	479.7888	19
479.8138	15	479.8238	20
479.8488	12	479.8563	19.25
479.8838	12.75	479.8888	22.75
479.9175	11.25	479.9225	15.75
479.95	11.25	479.9563	22.75
479.9838	13.5	479.99	21.25
480.015	14	480.0225	19.75
480.0475	11.75	480.055	20
480.08	14.75	480.0925	19

480.1125	10	480.125	23
480.1525	7.75	480.1575	20.25
480.1875	11.5	480.1913	19.75
480.215	8.5	480.2225	20.25
480.25	11.5	480.2563	18
480.2825	11	480.2888	24
480.3138	11.75	480.3238	16.5
480.3488	12.75	480.3563	20.5
480.3838	10	480.3913	18.5
480.4175	14.5	480.4225	21.5
480.45	10.75	480.4563	18.5
480.4838	11.75	480.49	19
480.515	10	480.5225	19.75
480.5475	13	480.555	19.5
480.58	10.75	480.5925	15.75
480.6138	8.75	480.625	20
480.6525	7	480.6575	16.75
480.685	10	480.6913	18.75
480.7175	10.5	480.725	18.75
480.75	10.5	480.7563	18.25
480.7825	9	480.7888	16
480.8138	9.5	480.8238	19.75
480.8488	9.25	480.8563	21.75
480.8838	10	480.8913	18
480.9175	6.25	480.9225	15.25
480.95	7.5	480.9563	15
480.9838	7.5	480.99	17.75
481.015	9.5	481.0225	17
481.0475	7.75	481.055	17.5
481.08	9.25	481.0925	13.25
481.1138	9.5	481.125	15
481.15	9.75	481.1575	17.5
481.185	9.75	481.1913	16.5
481.215	10.5	481.225	19.25
481.2513	10.75	481.2563	17.25
481.2825	10.25	481.2913	16.75
481.3138	9.75	481.3238	15.75
481.3488	5	481.3563	16.5
481.3813	9.25	481.3913	18.25
481.4175	9	481.4225	18
481.45	7.75	481.4563	19
481.4838	10.25	481.49	15.5
481.515	13	481.5225	18.75
481.5475	10.5	481.555	16.5
481.5825	11.25	481.5925	17.75
481.6138	13.75	481.625	15.5
481.65	10	481.6575	12.75
481.685	11.5	481.6913	16
481.715	11.25	481.725	13.75
481.7538	11.5	481.7563	14

481.7838	11	481.7913	18.25
481.815	13.5	481.8238	16.5
481.85	9	481.8563	15.75
481.885	11	481.8913	12.75
481.9213	12.5	481.9225	16.75
481.9513	11.5	481.9588	13
481.985	10.75	481.99	17.5
482.0175	10	482.0225	14
482.05	11.75	482.055	12.25
482.085	13	482.0925	14
482.1163	13.75	482.125	14
482.1525	10.75	482.1575	12.5
482.1888	12	482.1913	15.5
482.22	10.75	482.225	13.5
482.2525	11	482.2563	13.5
482.2838	11	482.2913	13.5
482.315	8.25	482.3238	11.75
482.3513	12	482.3563	20.5
482.385	11.75	482.3913	16
482.4213	10.25	482.4225	15
482.4513	9	482.4575	17.5
482.485	9.5	482.49	20.25
482.5175	9	482.5225	17.75
482.5475	7	482.555	18
482.585	10.25	482.5925	13.25
482.6163	11	482.625	20
482.6525	12	482.6575	15.25
482.6875	10	482.6888	17.25
482.72	10.5	482.725	15.5
482.7525	9.25	482.7563	17.25
482.7838	10.75	482.7913	14.75
482.815	11.5	482.8238	17.75
482.8525	13.25	482.8563	17.5
482.885	9.75	482.8913	23
482.9213	12.25	482.9225	20.75
482.9513	10	482.9575	17.75
482.985	11.25	482.99	15
483.0175	9.75	483.0225	18.25
483.0475	10.5	483.055	18
483.085	10.75	483.0925	16.25
483.1163	12	483.125	25.5
483.1513	8.75	483.1575	20.75
483.1875	8	483.1888	21.75
483.22	7.5	483.225	16
483.2525	11.25	483.2588	17.25
483.2838	9.5	483.2913	17.75
483.315	6.5	483.3238	16.75
483.3525	5.75	483.3563	17.75
483.385	6.5	483.3913	17
483.4213	6.25	483.4225	19.75

483.4513	7
483.485	9
483.5175	8
483.5475	9.25
483.585	8.75
483.6163	9.5
483.6513	12
483.6875	7.25
483.72	8.5
483.7525	10.5
483.7838	9.75
483.8163	9.5
483.8525	10
483.885	12
483.9213	12.25
483.9513	12.25
483.985	12.25
484.0175	11.25
484.0475	10
484.085	10.5
484.1163	11.25
484.1525	9.75
484.1875	8.5
484.22	12.75
484.2525	10
484.2838	11.75
484.3163	11.5
484.3525	14.25
484.385	10.5
484.4238	11.5
484.455	11.25
484.4875	10
484.52	13
484.5513	12.25
484.5888	11.25
484.6188	12.25
484.655	9.25
484.69	12.25
484.7225	11.75
484.755	14
484.785	12.75
484.8188	13.5
484.8538	12.5
484.8863	12.75
484.9213	10.75
484.955	14.5
484.9875	16.5
485.02	13.75
485.0513	11.75
485.0888	10.75

483.4575	16
483.49	15
483.5225	16.25
483.555	16.75
483.5925	16.25
483.6275	13.75
483.6575	17
483.6913	14
483.725	18.25
483.7588	17.25
483.7913	14
483.8238	16
483.8563	13.75
483.8913	13.5
483.9225	17.25
483.9575	18
483.99	16
484.0238	19
484.055	22
484.0913	20
484.1275	17
484.16	14.5
484.1913	14.5
484.225	13.25
484.2588	18.75
484.2913	15.75
484.3238	15
484.3563	15
484.3913	19.5
484.4225	16.5
484.4575	15.5
484.49	18.25
484.5238	19.25
484.555	20.75
484.5913	18.25
484.6275	20.5
484.6575	17.75
484.6913	16.5
484.725	20.5
484.7588	17.25
484.7913	18.75
484.8238	18.5
484.8563	14.75
484.8913	20.75
484.925	18.5
484.9575	17.75
484.99	17.5
485.0238	18.25
485.055	17.5
485.0913	26.5

485.1188	9.75	485.1275	24.25
485.155	9.75	485.1575	17.25
485.19	8.75	485.1913	17.75
485.2225	9.25	485.225	18.75
485.2538	8.75	485.2588	17.5
485.285	9	485.2913	18.25
485.3188	9.75	485.3238	19.5
485.3538	9.5	485.3563	17
485.3863	9.25	485.3913	19.75
485.4188	10.75	485.425	15
485.455	8.75	485.4575	19
485.4875	10.5	485.49	18
485.52	9	485.5238	14.5
485.5513	7.75	485.555	17.25
485.5888	8.5	485.5913	18.5
485.6188	6.5	485.6275	19
485.655	9.25	485.6575	19.75
485.69	11	485.6913	16
485.7225	9.25	485.725	20
485.7538	12.5	485.7588	14.5
485.785	13.75	485.7913	16.25
485.8188	10.5	485.8238	14.75
485.8538	9	485.8563	15.25
485.885	11.5	485.8913	16.5
485.9188	13.25	485.925	21
485.955	10.75	485.96	19.5
485.9875	12.5	485.99	19.25
486.02	11	486.0238	19
486.0513	10.25	486.055	19.75
486.0888	13.5	486.0913	19
486.1188	11	486.1275	20.25
486.155	10.5	486.1588	19.75
486.19	14	486.1913	17.75
486.2225	10.25	486.225	18.75
486.2538	11.25	486.2588	19
486.2875	10	486.2913	15.75
486.3175	11	486.3238	17.5
486.3538	10.75	486.3563	17.75
486.3875	12	486.3913	18.75
486.4188	10.25	486.425	18.5
486.455	9.5	486.4575	19.75
486.4875	10	486.4913	17.75
486.52	9.5	486.5238	20.5
486.5513	8.5	486.555	19
486.5888	11.25	486.5913	23.25
486.6188	13.25	486.6275	24.25
486.655	11.25	486.6588	18.5
486.6913	7.5	486.6913	16
486.7225	8.5	486.725	20.5
486.7538	7.5	486.76	20.75

486.7875	11	486.7925	18.5
486.8175	8.25	486.825	16.25
486.8538	10.5	486.86	18.25
486.8875	10.75	486.8925	22
486.9188	9.75	486.9263	21
486.955	7.25	486.9588	24.5
486.9875	9.25	486.9938	18
487.02	9.5	487.0263	21.25
487.0513	9	487.0575	18.75
487.0888	8.75	487.0963	19
487.1188	8.25	487.13	18
487.155	9.75	487.1613	19.25
487.1925	8	487.1938	17.5
487.2225	10.5	487.2275	19.5
487.2538	11.25	487.26	22.75
487.2875	9	487.2925	16.75
487.3175	5.5	487.325	23.75
487.3538	8	487.3625	21.5
487.3888	7.5	487.395	20
487.4188	11	487.4263	19.75
487.455	6.5	487.4588	18.75
487.4875	12.25	487.4938	20.25
487.52	9.25	487.5263	16.5
487.5513	7.75	487.5588	21
487.5888	9.5	487.5963	15.5
487.6188	9.25	487.63	19
487.655	6.5	487.6613	18.25
487.6913	8.25	487.6938	16.25
487.7225	6.25	487.7275	22.25
487.7538	5.5	487.76	20.25
487.7888	9	487.7925	21.75
487.82	7.5	487.825	17.25
487.8538	11.25	487.8625	20
487.8888	6.5	487.895	16.75
487.9188	7.75	487.9263	17.25
487.955	7.5	487.9588	16.25
487.9875	6.5	487.9938	20.5
488.02	7.75	488.0263	17.25
488.0513	7.75	488.0588	12
488.0888	6.25	488.0963	16.25
488.1188	9.75	488.13	18
488.155	11	488.1613	21.25
488.1888	11.75	488.1938	19.25
488.2225	9.25	488.2275	19.25
488.2538	8.5	488.26	22.5
488.2875	9.75	488.2925	18.25
488.32	12.5	488.325	18.75
488.3538	8	488.36	20
488.3888	9.75	488.3975	19.5
488.4213	10.25	488.4263	21.25

488.455	5.75	488.4575	17.5
488.4875	9.75	488.4938	20.75
488.52	8.5	488.5263	21.25
488.5513	10	488.5588	15
488.5888	11.25	488.5963	18.75
488.6188	11.25	488.6313	17.75
488.655	10.5	488.6613	16.5
488.6888	11	488.6938	19.5
488.7225	10	488.7275	18.75
488.7538	10	488.76	19
488.7875	12.75	488.7925	19.75
488.8213	9	488.825	18
488.8538	8	488.86	17.25
488.8888	10.75	488.895	16.25
488.9213	9.5	488.9263	18.5
488.955	8	488.9575	20.75
488.9875	8.5	488.9938	14.5
489.02	10.75	489.0263	14.75
489.0513	11.75	489.0588	14
489.0888	7	489.0963	16
489.1188	10.5	489.1313	18.25
489.155	8.25	489.1613	16.75
489.1875	9.5	489.1938	15
489.2225	9.75	489.23	13.5
489.255	10	489.26	16
489.2875	8.75	489.2925	14.75
489.32	7.5	489.325	13.5
489.3538	8	489.36	15.25
489.3888	7.5	489.395	11
489.4225	9.75	489.4263	16.25
489.455	10.75	489.4575	14.75
489.4875	5.25	489.4938	14.5
489.5213	9.75	489.5263	11.75
489.5513	7.75	489.5588	13.75
489.5888	9.5	489.5963	14.25
489.6188	8.25	489.6313	11.5
489.655	10.5	489.6613	18
489.6875	7	489.6938	15.5
489.7225	12.25	489.73	22
489.7538	11.5	489.76	14.75
489.7875	6.75	489.7925	18.25
489.82	9.75	489.825	14
489.8538	10.25	489.8625	13.5
489.8913	9.25	489.895	14.75
489.9213	11.75	489.9263	14
489.955	11	489.9575	10
489.9875	12.75	489.9938	18.25
490.0213	13	490.0263	16.75
490.0525	11.75	490.0588	16.25
490.0888	11.75	490.0963	15.5

490.12	9.75	490.1313	13.5
490.1588	9.25	490.1613	14
490.19	11.25	490.1938	19
490.225	10.75	490.23	19.75
490.255	8.5	490.26	20.25
490.2888	10	490.2925	16.5
490.3238	9.5	490.325	15.5
490.355	6	490.3625	21.25
490.39	7	490.395	17.25
490.4225	8	490.4263	12.5
490.4563	6.75	490.4575	17.25
490.49	7.5	490.4938	16.75
490.5238	7.25	490.5263	17.5
490.555	9.25	490.5588	19.75
490.5913	7.5	490.5963	19
490.6225	8.5	490.6313	19.75
490.6588	10.75	490.6613	13.75
490.69	9.75	490.6938	15
490.725	9.5	490.7325	18
490.755	8.75	490.76	15.5
490.7888	6	490.7925	15.5
490.8238	11.5	490.8275	14.5
490.855	8.25	490.8625	15
490.89	6.5	490.895	14.25
490.9225	7.5	490.9263	14
490.9563	9.25	490.9575	15.5
490.99	6.75	490.9938	16.75
491.0238	8.75	491.0263	13.25
491.055	8.5	491.0588	14.5
491.0913	7.75	491.0963	15.5
491.1225	8.75	491.1313	16.75
491.1588	8.5	491.1613	14.25
491.19	5	491.1938	13
491.225	8.75	491.23	13.25
491.255	9.25	491.26	15
491.2888	8.5	491.295	16
491.3213	6.75	491.3275	15.75
491.355	8.5	491.3625	15.5
491.39	7.5	491.395	17.5
491.4225	6.5	491.4263	12.75
491.4563	10.25	491.46	13.5
491.49	8	491.4938	14.75
491.5238	10.25	491.5263	14.25
491.555	7	491.5588	13.25
491.5913	8.75	491.5988	13
491.6225	9.75	491.63	13.75
491.6588	8	491.6613	12.25
491.69	10.25	491.6938	15.5
491.725	10.25	491.73	12.5
491.7563	8	491.76	13.25

491.7888	7.75	491.795	14.25
491.8213	7.25	491.8275	15.5
491.855	9.5	491.8625	18.25
491.89	5.75	491.895	17.5
491.9225	7.5	491.9263	12
491.9563	7.75	491.96	12.75
491.99	7	491.9938	15
492.0238	6.75	492.0263	15.5
492.055	5.75	492.0588	15.25
492.0913	7.5	492.0988	19.25
492.1225	5.25	492.13	23.75
492.1588	8.5	492.1613	15.5
492.19	5.25	492.1938	15.25
492.225	5.25	492.2275	18
492.255	7.25	492.26	15.5
492.2888	6.25	492.295	17.5
492.3213	6.75	492.3275	13.75
492.355	6.5	492.3625	18.75
492.3925	5.5	492.395	16.25
492.4225	5.5	492.4263	16
492.4563	7	492.46	18
492.49	6.75	492.4938	20
492.5238	8	492.5263	14.5
492.555	7.75	492.5588	16.25
492.5913	8.5	492.5988	17.25
492.6225	11.75	492.63	18.25
492.6588	7.5	492.6613	13
492.69	10	492.6938	15.25
492.725	11.5	492.7275	18.75
492.755	7.25	492.76	17
492.7888	6	492.795	17.25
492.8213	7.5	492.8275	15.75
492.855	7.75	492.8625	33.75
492.8925	8	492.895	27.75
492.9225	7.5	492.9263	22.5
492.955	7.5	492.96	33.75
492.99	9.25	492.9938	42.25
493.0238	8.75	493.0263	23.25
493.055	9.5	493.0588	21
493.0913	7.75	493.0988	26.5
493.1225	7.75	493.13	19.75
493.1588	8.5	493.1613	18.25
493.19	9.75	493.1938	20
493.225	5	493.2275	28.25
493.255	8.5	493.26	20.75
493.2888	9	493.295	36
493.3213	9	493.33	22.25
493.355	9.25	493.3625	42.5
493.3925	8.75	493.395	54
493.4225	10.5	493.4263	19.75

493.4563	8.25	493.46	45.75
493.49	10	493.4938	44.25
493.5238	10.25	493.5263	19.5
493.555	10.25	493.5588	18.25
493.5913	10.5	493.5988	18
493.6225	10	493.63	16.25
493.6588	10.5	493.6613	22.25
493.69	8.5	493.695	19
493.725	10.5	493.7275	17.5
493.755	10.75	493.76	20.25
493.7913	10	493.795	19.75
493.8213	8.75	493.83	16.75
493.855	11	493.8625	15.75
493.8925	7.5	493.895	19
493.9225	10.5	493.9263	19.25
493.955	10.25	493.96	24.5
493.99	10.25	493.9938	24.25
494.0238	8.25	494.0263	24.5
494.055	8.75	494.0588	18.5
494.0913	11.25	494.0988	20.5
494.1225	10.75	494.13	25.75
494.1588	14	494.1613	21.5
494.19	14.25	494.195	20.5
494.225	11.75	494.2275	17.25
494.2575	9.75	494.26	23.25
494.2913	15.5	494.295	21.25
494.3213	11.75	494.33	21.25
494.355	12.75	494.3625	35
494.3925	10	494.3925	24.5
494.4225	12.25	494.4263	18.5
494.455	12	494.46	20.75
494.49	13.75	494.4938	17.5
494.5238	12.25	494.5263	17.5
494.555	11.75	494.5588	16
494.5913	9.75	494.5988	15.75
494.6263	11.75	494.63	20
494.6613	10.5	494.6613	20.25
494.6925	12.5	494.695	19.25
494.7275	11.5	494.7275	17.25
494.7613	10.25	494.76	16.75
494.7938	9.5	494.795	21.25
494.8238	10	494.83	15.25
494.8563	12.25	494.8625	14.5
494.8938	8.25	494.8925	15.75
494.9238	11.5	494.9263	19.75
494.9563	8.75	494.96	21
494.9913	12.5	494.9938	19
495.025	10.75	495.0263	17
495.0563	10.5	495.0588	16.75
495.0938	12.5	495.0988	13.75

495.1263	10	495.13	16
495.1613	9.5	495.1613	18.75
495.1925	11	495.195	18.5
495.2275	8.75	495.2275	18
495.2575	11.5	495.26	18.25
495.2938	10.75	495.295	20
495.3238	9.5	495.33	16.75
495.3588	12.25	495.3625	18.5
495.3938	9.25	495.3925	19
495.4238	12.25	495.4263	18
495.4588	12.75	495.46	18
495.4913	10.5	495.4938	23.75
495.525	12.5	495.5275	19.75
495.5563	10.25	495.5588	19.5
495.5938	11.5	495.5988	21.5
495.6263	10.75	495.63	19
495.6613	10.25	495.6613	23
495.6925	9.75	495.695	22
495.7275	8	495.7275	22
495.7575	6.75	495.76	16.25
495.7938	8.75	495.795	24.75
495.8238	8.25	495.83	25.75
495.8563	8.75	495.8625	22
495.8938	11.75	495.8925	18.5
495.9238	6	495.9288	18.5
495.9563	10.25	495.9625	17.25
495.9913	11.25	495.9938	20.75
496.025	13.5	496.0275	20.75
496.0563	14.25	496.0588	20
496.0938	11	496.0988	22
496.1263	11.5	496.13	27.75
496.1613	10.75	496.1613	28.25
496.1925	11.5	496.195	26.5
496.2275	12.75	496.2275	34
496.2575	10.5	496.26	22.25
496.2938	10.5	496.295	21.5
496.3238	10.5	496.33	22.5
496.3563	11.25	496.3625	24.75
496.3938	16.25	496.3925	21.75
496.4238	12.5	496.4288	23.25
496.4588	12.25	496.46	22.25
496.4913	12	496.4938	15.75
496.525	11.25		
496.5563	11.75	496.5588	45.25
496.5938	13.25	496.5988	23.75
496.6263	10.75	496.63	31.75
496.6613	11.75		
496.6925	12	496.695	23
496.7275	15.25	496.7275	52
496.7575	11.5	496.76	34.75

496.7938	12.5	496.795	34.75
496.8238	12.5	496.8275	48
496.8563	11.5	496.8625	17
496.8938	9.75		
496.9238	11	496.9288	30.75
496.9563	12	496.96	19.75
496.9913	10.75	496.9938	19.75
497.0263	12.25	497.0275	17
497.0575	11	497.0613	27.75
497.0938	12.75	497.0988	18.25
497.1263	14	497.1313	17.25
497.1613	15	497.1613	16
497.1925	16	497.195	17.5
497.2275	15.75	497.2275	17.25
497.2575	12.25	497.26	22
497.2938	10.75	497.295	33.25
497.3238	12.75	497.3275	18.75
497.3563	11.75	497.3625	31.75
497.3938	14	497.3925	40.25
497.4238	14	497.4288	19.5
497.4563	13.5	497.46	30.25
497.4913	14.75	497.4938	17.5
497.5263	16.5	497.5263	23.5
497.5575	15.5	497.5613	19.5
497.5913	9.5	497.5988	21
497.6263	11	497.63	18.5
497.6613	10	497.6625	28.5
497.6925	11.25	497.6963	22.5
497.7275	12.25	497.7288	17.75
497.7575	12.25	497.7613	33.25
497.7938	12.75	497.7988	17.75
497.8238	10.75	497.8288	24.75
497.8613	8.25	497.8638	24.25
497.8938	13.5	497.8938	17.5
497.9238	9.5	497.9313	19.5
497.9563	9	497.965	22.25
497.9913	10.25	497.9963	30.75
498.0263	12.25	498.03	22.25
498.0563	14.5	498.0638	21.25
498.0913	12.75	498.1013	23.75
498.1263	14.5	498.1325	17
498.1613	14.5	498.1638	23.75
498.1925	9.5	498.1963	21.75
498.2275	13.75	498.2288	18.25
498.2575	15.75	498.2613	16.75
498.2938	14	498.2988	29
498.3238	9.75	498.3288	18.25
498.3588	10.75	498.3638	16.75
498.3938	10.25	498.3938	18
498.4238	8.25	498.4313	16.75

498.4563	12	498.4625	20.25
498.4913	11.5	498.4963	22.5
498.5263	7.25	498.53	17.75
498.5563	8	498.5638	15.5
498.5913	8.5	498.6013	20.5
498.6263	8.25	498.6325	15.75
498.6613	9.5	498.6638	19.75
498.6925	9.5	498.6963	18
498.7275	10.25	498.7288	13.25
498.76	11.5	498.7613	16
498.7938	10.25	498.7988	20.25
498.8238	10.25	498.8288	22.5
498.8588	8.25	498.8638	14.5
498.8938	12.5	498.8938	19.25
498.9238	11	498.9313	16.25
498.9563	10	498.9625	17.25
498.9913	10	498.9963	18.5
499.0263	8.75	499.0288	18.5
499.0563	11.25	499.0638	13.5
499.0913	11	499.1	18.75
499.1288	11.75	499.1325	21.5
499.1613	8.25	499.1638	18.75
499.1925	12	499.1963	19.75
499.2275	10	499.2288	22.5
499.26	13.75	499.2638	21.5
499.2938	12	499.2988	21
499.3238	11	499.3288	21.25
499.3588	12.25	499.3638	23.25
499.3938	12.25	499.3938	19.75
499.4238	12.25	499.4313	21.5
499.4563	10.75	499.4625	24.25
499.4913	11	499.4963	17.5
499.5263	13.25	499.5288	16
499.5563	14.75	499.5638	16.5
499.5913	9.5	499.6	22
499.6263	9.75	499.6325	18.25
499.6613	8.25	499.6638	21
499.6925	7.5	499.6988	21
499.7275	7.25	499.7288	35.5
499.76	6	499.765	16.25
499.7938	8.5	499.7988	46
499.8238	12	499.8288	32.25
499.8588	7	499.8638	45.25
499.8938	11.25	499.8938	47.75
499.9238	12.25	499.9313	20.25
499.9563	8	499.9625	35
499.9913	10.25	499.9963	27.5
500.0263	11.25	500.0288	17.5
500.0563	8.75	500.0638	21.25
500.0913	10.75	500.1	25.5

500.1263	7.5	500.1325	20.5
500.1613	13.75	500.1638	22
500.1925	9.5	500.1963	20.25
500.2275	12	500.2288	17.5
500.26	8.75	500.2638	18.25
500.2938	8.25	500.2988	17.75
500.3238	11	500.3288	18.75
500.3588	8.5	500.3638	20.5
500.3938	9	500.3963	18.25
500.4238	7.75	500.4313	15.5
500.4588	8.5	500.4625	18
500.4913	11	500.4963	19.25
500.5263	11.75	500.5288	25.25
500.5563	11.25	500.5638	16.75
500.5913	11	500.6	16.25
500.6263	12	500.6325	14.5
500.6613	9.25	500.6638	18
500.6925	8.25	500.6963	16
500.7275	8	500.7288	12.5
500.76	8.75	500.7638	19.25
500.7938	10.25	500.7988	15.5
500.8238	10	500.8288	17
500.8588	8	500.8638	16
500.8938	11.5	500.8963	15.75
500.9238	10.25	500.9288	19.5
500.9588	11.75	500.9625	13.75
500.9938	11.75	500.9963	21.75
501.0263	11.75	501.0288	16
501.0563	11.25	501.0638	25.75
501.0913	7.5	501.1	17
501.1263	13.5	501.1325	18
501.1613	10.25	501.1638	30.5
501.1925	8.75	501.1963	22.75
501.2275	11.75	501.2288	21.75
501.26	13	501.2638	18.25
501.2938	9.25	501.2963	23.75
501.3238	11.25	501.3288	19.75
501.3588	10.75	501.3638	27.75
501.3938	8.5	501.3963	18.5
501.4238	12.25	501.4288	17
501.4588	9	501.4625	16.25
501.4938	11	501.4963	19
501.5263	7.5	501.5288	18.5
501.5563	7.5	501.5638	13.5
501.5913	11.5	501.6013	14.75
501.6263	12.75	501.6325	15.5
501.6613	14.25	501.6638	15.25
501.6925	11	501.6963	15.75
501.7275	10.25	501.7313	13.75
501.76	10	501.7638	17.25

501.7938	10.75	501.7963	14
501.8238	8.5	501.8288	14
501.8575	11	501.8638	12.25
501.8938	12	501.8963	17.25
501.9238	10.5	501.9288	13
501.9588	15	501.9625	21.25
501.9938	11	501.9963	12.25
502.0263	12	502.0288	18
502.0563	14	502.0638	23.25
502.0925	13	502.1	20
502.1263	14.5	502.1325	15.5
502.1613	13	502.1638	15
502.1925	13.5	502.1963	21
502.2275	12.25	502.2313	14.75
502.26	13	502.2638	15.5
502.2938	15.75	502.2963	14.25
502.325	16.5	502.3288	13.5
502.3588	11.25	502.3638	13
502.3963	16.25	502.3963	13.5
502.4263	13	502.4288	15.25
502.4613	14	502.4625	15.25
502.4963	12	502.4963	16
502.5288	13.25	502.5288	16.25
502.5588	11.25	502.5638	17.5
502.595	12.5	502.6	17.75
502.6313	12.75	502.635	16.25
502.6625	10.25	502.6638	17.25
502.6938	9.5	502.6963	18
502.7313	12.75	502.7313	20
502.7613	11.5	502.7638	16.25
502.795	8.75	502.7963	17.25
502.825	10.5	502.8288	16
502.86	11.75	502.8638	27
502.8963	11	502.8963	24
502.9263	10.75	502.9288	15.5
502.9625	10	502.9625	16
502.9963	9	502.9963	20.25
503.0288	9	503.0288	14.5
503.0588	6.5	503.0638	13.5
503.095	8.25	503.0975	14
503.1313	9.75	503.1338	13
503.1625	8.75	503.1638	17.25
503.1938	12.5	503.1963	13.75
503.2313	9.75	503.2313	13
503.2613	10	503.2638	13.5
503.295	7.75	503.2963	16
503.325	7.75	503.3288	15.5
503.3588	11	503.3638	13
503.3963	8.5	503.3963	18
503.4263	9	503.4288	17

503.4625	9.75	503.4625	16.5
503.4963	7.75	503.4963	15.5
503.5288	11.5	503.5288	15.25
503.5588	10.5	503.5638	19.75
503.595	8.25	503.5975	16
503.6313	8.25	503.6338	16.25
503.6625	8.25	503.6663	20.75
503.6938	10.75	503.6988	13.75
503.7313	7	503.7325	13.75
503.7613	9.75	503.7663	14.5
503.795	10.25	503.7988	17.75
503.825	9.5	503.8313	24.75
503.8588	7.25	503.8663	16.75
503.8963	8.5	503.8988	15.75
503.9263	10.5	503.93	13.5
503.965	9	503.9663	13.75
503.9963	6.75	503.9988	14.25
504.0288	8.25	504.03	17.25
504.0588	7.5	504.065	13
504.095	7	504.0988	13.25
504.1313	5.25	504.135	14.5
504.1625	7	504.1663	15.25
504.1938	10	504.1988	16.25
504.2313	9.25	504.2325	15.25
504.2613	7	504.2663	14.75
504.295	10.5	504.2988	17
504.325	7.75	504.3313	21.25
504.3613	7	504.3663	16.25
504.3963	6.75	504.3988	14.25
504.4263	6.75	504.43	19.5
504.4625	7	504.4663	16.25
504.4963	8.5	504.4988	12.5
504.5288	6.75	504.53	17
504.5588	8.25	504.565	20
504.595	7.75	504.5988	18
504.6288	8.75	504.635	16
504.6625	9.5	504.6663	19.75
504.6938	9	504.6988	13.5
504.7313	9	504.7325	18.5
504.7613	8	504.7663	15.25
504.795	8.5	504.7988	13
504.825	7.75	504.8313	15.5
504.8613	9.75	504.8663	18.75
504.8963	6.5	504.8988	15.5
504.9263	11	504.93	17.25
504.9625	8.25	504.9663	19.25
504.9963	8.75	504.9988	19.75
505.0288	11.5	505.03	14.5
505.0588	10.25	505.0625	17.75
505.095	7.75	505.0988	19

505.1313	8.25	505.1363	20.25
505.1625	8	505.1663	22
505.1975	7	505.2	20.25
505.2313	4.5	505.2325	18
505.2613	8.5	505.2663	19.75
505.295	9	505.2988	17.75
505.325	8	505.3313	18
505.3613	7	505.3688	17
505.3963	8	505.3988	17.75
505.4263	7	505.43	19.5
505.4625	8.5	505.4663	19.75
505.4963	7.5	505.4988	18.25
505.5288	6.75	505.53	16
505.5588	8.5	505.5625	15.5
505.595	8.5	505.5988	13.5
505.6288	9.5	505.635	19.5
505.6625	11.25	505.6663	12.75
505.6988	8.75	505.7	13
505.7313	6.5	505.7325	22
505.7613	9.25	505.7663	18.75
505.795	7.75	505.7988	14
505.825	6.75	505.8313	15
505.8613	8.5	505.8688	13.75
505.8963	9.5	505.8988	15.25
505.9263	8	505.93	15
505.9625	8.75	505.9663	13
505.9963	10.25	505.9988	14.75
506.0275	8.5	506.03	14.75
506.0588	12	506.0625	12.25
506.095	7	506.0988	16.5
506.1288	10	506.135	15.25
506.1625	10.75	506.1663	14.75
506.1975	12	506.2	15.5
506.2313	8.5	506.2325	12.75
506.2613	13.75	506.2663	12.25
506.2975	11.75	506.2988	13
506.325	10.25	506.3313	11.75
506.3638	11.25	506.3688	14.25
506.3963	11.75	506.3988	14
506.4263	10.5	506.43	10.5
506.465	10	506.4688	12.75
506.4963	12.5	506.4988	13.75
506.5275	11	506.5313	24
506.5588	14.25	506.5625	13
506.595	10.25	506.5988	16
506.6288	12	506.635	12.5
506.6625	13	506.6663	11.75
506.6975	13.5	506.7	11.75
506.7313	10.75	506.7325	13
506.7613	11.75	506.7663	9.5

506.7975	11.25	506.7988	16.75
506.825	13.5	506.8313	16.75
506.8638	13	506.8688	16
506.8963	10.25	506.8988	16.5
506.9275	11.25	506.93	13.25
506.9625	10.75	506.9663	16.25
506.9963	11.5	506.9988	15.5
507.0275	16	507.0313	13.75
507.0588	14.75	507.0625	13
507.095	14	507.0988	13.75
507.1288	14	507.135	15.5
507.1625	12.25	507.1663	11.75
507.1975	11.5	507.2	13
507.2313	10.75	507.2325	13.25
507.2613	10.5	507.2663	12.75
507.295	13.5	507.2988	11
507.325	11	507.33	11.5
507.3638	11.5	507.3688	14.5
507.3963	8.75	507.3988	6.25
507.4275	9.25	507.43	12.25
507.4625	10.25	507.4663	15.5
507.4963	10.5	507.4988	14.25
507.5275	8.75	507.5313	12
507.5588	6.75	507.5625	13.25
507.595	9.25	507.6	14.75
507.6288	6.75	507.6388	21.25
507.6625	8.75	507.6713	14
507.6988	10	507.7013	16.25
507.7313	7.75	507.7338	14.5
507.7613	8.5	507.7675	13
507.795	8.5	507.8	15.5
507.825	8.5	507.8325	16
507.8638	11	507.8688	12.25
507.895	8.5	507.9	12.75
507.9275	8.25	507.935	15
507.9625	10.25	507.9688	15.75
507.9975	8.5	508.0013	17.5
508.0275	7.5	508.0338	16.25
508.0588	10	508.065	13.5
508.095	10.5	508.1025	16.25
508.1288	9.5	508.1388	15.75
508.1625	10.25	508.1688	13
508.1988	10.75	508.2013	14.5
508.2313	9	508.2338	13.25
508.2613	13	508.2675	17.5
508.295	9	508.3	14.75
508.3275	10.75	508.3325	11.25
508.365	8.75	508.3688	17
508.3963	9	508.4	15
508.4275	8.5	508.435	12

508.4625	13	508.4688	13.25
508.4963	9	508.5013	16.75
508.5275	10.5	508.5338	12.75
508.5588	11.5	508.565	13
508.595	10.25	508.6025	14.5
508.6288	7.5	508.6375	12.5
508.665	11	508.6688	14
508.6988	11	508.7013	15.5
508.7313	9.25	508.7338	14.75
508.7613	10	508.7675	14.25
508.795	9.25	508.8	20.5
508.8275	8.5	508.8325	15.25
508.8663	9	508.87	13.75
508.895	8.25	508.9	15.75
508.93	9.5	508.935	12.5
508.9625	9	508.9688	15
508.9963	8.75	509.0013	16
509.0275	8.5	509.0338	13.75
509.0588	9.5	509.065	13.75
509.095	8	509.1	14
509.1288	9.75	509.1375	16.5
509.165	9.25	509.1688	15.75
509.2	7.25	509.2013	12.25
509.2313	9	509.2338	15.25
509.2613	9.5	509.2675	13.75
509.295	12	509.3	14.5
509.3275	11.75	509.3325	11
509.3638	9	509.3688	14
509.395	7.5	509.4	11
509.43	8	509.435	15.25
509.4625	10.25	509.4688	15
509.4963	7.5	509.5013	15
509.5275	8.5	509.5338	12.5
509.5588	10	509.565	13.75
509.5963	6.75	509.6	16
509.6288	9.5	509.6375	14
509.665	10	509.6688	15
509.6988	6	509.7013	14.5
509.7313	9.5	509.7338	15.75
509.7613	10.25	509.7675	12.75
509.795	7.25	509.8	16
509.8275	13.5	509.8325	14.5
509.8638	12.75	509.8688	17
509.895	7.75	509.9	13
509.93	9.25	509.935	18.5
509.9625	9.75	509.9688	17.75
509.9988	12.5	510.0013	19.25
510.03	13.25	510.0338	19.75
510.0613	9.75	510.065	14.5
510.1	9.5	510.1	19.25

510.1313	8.25	510.1375	18.75
510.165	11.25	510.1688	18.5
510.2	11.75	510.2013	21.5
510.2325	9.25	510.2338	16.5
510.2663	9.75	510.2675	22.5
510.2963	11.5	510.3	18
510.33	9.75	510.3325	18
510.365	8	510.3675	15
510.3975	9.75	510.4	19.75
510.4325	8.25	510.435	21
510.465	9	510.4688	20.25
510.4988	6.75	510.5013	19.25
510.53	8.25	510.5338	19.25
510.5613	12	510.565	18.25
510.6	8.5	510.6	23.5
510.6313	10.75	510.6375	16.75
510.6663	9	510.6688	14
510.7	10.75	510.7013	18.25
510.7325	8.5	510.7338	15
510.7625	8	510.7675	12.25
510.7963	8.5	510.8	16.75
510.8313	7.75	510.8325	17.25
510.865	10	510.8675	18
510.8975	9	510.9	19
510.9325	10.25	510.935	15.5
510.965	9	510.9688	15.5
510.9988	10.25	511.0013	16
511.03	9.75	511.0338	15
511.0613	9.25	511.065	15.5
511.1	10.5	511.1	17.75
511.1313	8.5	511.1388	18
511.165	11.5	511.1688	14.25
511.2	12	511.2013	12.75
511.2325	10.25	511.2338	20.25
511.2625	10	511.2675	18.5
511.2963	13	511.3	15.25
511.3313	13.5	511.3325	10.75
511.365	13.25	511.3675	16.5
511.3975	10.25	511.4	16.5
511.435	9.5	511.435	15.5
511.465	13	511.4688	18.5
511.4988	11.5	511.5013	15.25
511.53	12.25	511.5338	21.25
511.5613	9.75	511.5675	19
511.6	13.5	511.6	19.5
511.6338	11.25	511.6375	20.5
511.6663	12	511.6688	24
511.7	11	511.7013	16.5
511.7325	9.25	511.7363	17
511.7625	13	511.7675	15.75

511.7975	11
511.8313	11.5
511.865	10.25
511.8975	9.75
511.9325	9.75
511.965	10.25
511.9988	10.5
512.035	13
512.065	8.5
512.1038	8
512.1375	8.25
512.1725	6.75
512.2038	9.75
512.2363	6.75
512.2663	5.25
512.3	7.25
512.3363	6.75
512.3688	8.25
512.4013	9.5
512.4363	10.5
512.47	8
512.5025	7
512.5338	9.5
512.565	7
512.6038	9
512.64	8.75
512.6725	8.75
512.7038	8
512.7363	9.75
512.7663	8
512.8	7.25
512.8363	6.75
512.8713	8.5
512.9013	9.25
512.9363	11.25
512.97	9.5
513.0025	10.75
513.0338	8
513.065	8.25
513.1038	11.5
513.1375	9.75
513.1725	9
513.2063	11.25
513.2363	9.75
513.2663	8.5
513.3	9.5
513.3388	10.5
513.3713	10
513.4013	10.75
513.4363	11.25

511.8	20.75
511.8325	20
511.8675	17.5
511.9	16.5
511.935	22.75
511.9688	15.25
512.0013	17.5
512.0338	18.25
512.065	18
512.0975	18
512.135	17.75
512.1688	15.25
512.2013	16.5
512.2363	18.75
512.265	15.75
512.3	19.5
512.3325	13.75
512.3675	22.5
512.4025	25
512.435	20.25
512.4688	19.75
512.5013	21.5
512.5338	26.75
512.565	16.5
512.5975	17
512.635	14.75
512.6688	19
512.7013	18
512.7338	15.75
512.765	18
512.8	20.5
512.8325	18
512.8675	18
512.9	19.25
512.935	22.75
512.9688	16.75
513.0013	17
513.0338	17.5
513.065	20.25
513.0975	16.25
513.135	19.25
513.1688	21.25
513.2013	18.25
513.2338	19.5
513.2675	17.75
513.3	23.75
513.3325	21.25
513.3675	22
513.4	19.25
513.4375	21.25

513.47	9.5	513.4688	18.5
513.5025	11.75	513.5013	16
513.5338	7.75	513.5338	21
513.565	8.5	513.565	20.25
513.6038	10.75	513.5975	17
513.6375	9.75	513.6325	23.25
513.6725	8.5	513.6688	22.25
513.7038	8	513.7013	19.5
513.7363	11.25	513.7338	22
513.7663	11.25	513.7675	20
513.8038	13.5	513.8	18.5
513.8388	11.75	513.8325	19.75
513.8713	8	513.8675	22.25
513.9013	10.5	513.8988	24.25
513.9363	10.5	513.935	22.75
513.97	10.75	513.9688	23
514.0025	11.75	514.0013	22.5
514.0338	9.5	514.0338	25.25
514.065	12.25	514.065	24
514.105	11.5	514.0975	24.5
514.1375	10.5	514.1325	19.25
514.1725	10.25	514.1688	25.5
514.2038	12.25	514.2013	27.75
514.2363	13	514.2338	21.75
514.2663	15.75	514.2675	22.75
514.2975	12.75	514.3	22
514.3388	11.5	514.3325	21.75
514.3725	11.5	514.3675	20.5
514.4038	11.25	514.3988	19.25
514.4363	8.75	514.4375	21
514.47	11.25	514.4688	21
514.5025	10.25	514.5013	16.75
514.5338	12.75	514.5338	17.25
514.565	8.5	514.565	18.25
514.605	10	514.5975	19.75
514.6375	12	514.6325	19.25
514.6725	8.5	514.6688	18.5
514.7038	8	514.7013	18.5
514.7363	8.75	514.7338	23.5
514.7663	9.5	514.7675	17
514.7975	7.25	514.8	21.25
514.8388	8	514.8325	17.25
514.8713	9	514.8675	17.5
514.9038	9.25	514.8988	17.75
514.9388	9.25	514.9375	25.25
514.97	11	514.9688	19.5
515.0025	10.5	515.0013	19
515.0338	9.75	515.0338	19.5
515.065	9	515.065	24
515.105	11.75	515.0975	21.75

515.1375	10.25	515.1325	22
515.17	9.25	515.1688	23.25
515.2038	9.5	515.2013	22.5
515.2363	8.5	515.2338	23.75
515.2688	5.75	515.2675	21.25
515.2975	11.25	515.3	20.5
515.3413	11	515.3325	22
515.3713	8.25	515.365	19.75
515.4038	8	515.4013	17.25
515.4363	8.75	515.4375	22
515.47	7.25	515.4688	23
515.5025	9.5	515.5013	15
515.535	11	515.5338	21.5
515.565	10.25	515.565	20
515.605	9.5	515.5975	16.75
515.6375	12.75	515.6325	19.75
515.67	12.5	515.6688	22
515.7038	10.25	515.7013	21
515.7363	8.5	515.7338	18.5
515.7663	10	515.7675	17.75
515.7975	12.25	515.8	23
515.8413	9.25	515.8325	19
515.8713	11	515.865	18.25
515.9038	10.75	515.8988	21.25
515.9363	7.25	515.935	18
515.97	11.25	515.9688	22.75
516.0025	8	516.0013	20.5
516.0338	9.25	516.0338	19.25
516.0675	9.25	516.065	16.25
516.105	10.5	516.0975	20.75
516.1388	9.5	516.1363	23.5
516.17	12.75	516.17	19.25
516.2038	12.25	516.205	18
516.2363	10.5	516.24	21
516.2663	10	516.2713	19.5
516.2975	12.75	516.3038	19.25
516.3413	12	516.3363	21.5
516.3713	9	516.3688	20.25
516.4038	11	516.4025	25.25
516.4363	9.75	516.44	28.75
516.47	11	516.4738	25
516.5025	12.5	516.5063	30.75
516.5338	8.5	516.5388	26
516.5688	10.75	516.5688	22.5
516.605	12.5	516.6013	24.75
516.6388	12.25	516.6363	23.75
516.67	15	516.67	23.75
516.7025	15	516.7075	19.25
516.7388	10	516.74	22.75
516.7663	14.25	516.7713	21.25

516.7975	11.75	516.8038	20.5
516.8413	11.25	516.8363	21
516.8713	14	516.8688	28.5
516.9038	12.5	516.9025	23.5
516.9363	12.25	516.94	23.25
516.97	7.5	516.9738	23.25
517.0025	10.75	517.0063	23.75
517.0338	11.75	517.0388	22
517.0688	13.5	517.0688	26.75
517.105	11.75	517.1013	23.25
517.1388	11.75	517.1363	26.5
517.17	10	517.17	22.75
517.2025	13	517.205	26.25
517.2363	10.75	517.24	21.75
517.2663	9.25	517.2713	21.5
517.2975	10	517.3038	24.75
517.3425	9.5	517.3363	23.25
517.3713	10.5	517.3688	20
517.4038	13	517.4025	17.25
517.4363	10.25	517.4375	19.25
517.47	14.75	517.4738	25.25
517.5025	12	517.5063	27
517.5338	11.5	517.5388	22.25
517.5688	12.75	517.5688	23.5
517.605	11.25	517.6013	30
517.6388	9.75	517.6363	25.25
517.67	9.25	517.67	22.5
517.7025	9	517.705	24
517.7363	11	517.74	17.5
517.7663	10.25	517.7713	18.25
517.8	7.75	517.8038	21.75
517.8413	10	517.8363	23.75
517.8713	9.75	517.8688	18.5
517.9038	13.25	517.9025	16
517.9388	7.5	517.9375	19
517.97	7.5	517.9738	17.5
518.0025	13.25	518.0063	21
518.0338	12	518.0388	26.5
518.0688	10	518.0688	15.75
518.105	7.75	518.1013	16
518.1388	11.25	518.1363	17.25
518.17	12.75	518.17	18.5
518.2025	10.5	518.205	18.25
518.2363	9.25	518.24	16.25
518.2663	11.25	518.2713	15.5
518.3	12.25	518.3038	20.25
518.3413	10.75	518.3363	15.75
518.3738	11.75	518.3688	22.25
518.4038	12.5	518.4025	16
518.4388	9.75	518.4375	19.25

518.47	13.5	518.4738	16.75
518.5025	13.25	518.5063	14.25
518.5338	11.25	518.54	19
518.5688	13	518.57	17
518.605	12	518.6025	14.25
518.6388	11.75	518.6375	18.5
518.67	15	518.6725	16.25
518.7025	12.5	518.7088	15.25
518.7363	11.5	518.7413	14.5
518.7663	11.75	518.7738	13.75
518.8	10.75	518.8063	14.5
518.8413	9.5	518.8388	13.5
518.8738	9.5	518.87	10.5
518.9038	9.25	518.905	15.5
518.935	10.5	518.9388	15.25
518.97	9.75	518.9763	14.75
519.0025	10	519.0088	14.75
519.0363	14	519.04	17.25
519.0688	9.75	519.07	15
519.105	6.75	519.1025	15.5
519.1388	11.75	519.1375	16.25
519.17	10.25	519.1725	13.5
519.2025	11	519.2088	15
519.2363	10.25	519.2425	16
519.2688	13.5	519.2738	17.5
519.3	12.25	519.3063	12.5
519.3413	12.75	519.3388	13
519.3738	9.5	519.37	16
519.4038	13	519.405	13.5
519.435	10.75	519.4388	14
519.47	7.25	519.4763	12.25
519.5025	7.75	519.5088	12.75
519.535	13	519.54	14.75
519.57	9.5	519.57	12.75
519.6063	10	519.6025	16
519.6425	8	519.64	12
519.67	11.25	519.6725	14.5
519.7025	9.75	519.7075	14.25
519.7363	11.25	519.7413	16.25
519.7663	11.5	519.7738	15
519.8	14.25	519.8063	14.75
519.8413	9.25	519.8388	18.75
519.8738	8	519.8725	15
519.9038	13	519.9063	18
519.935	9	519.9425	16
519.9675	9.25	519.9788	13.5
520.0025	9.75	520.0113	15.75
520.035	12	520.0425	19
520.07	9.75	520.0725	19.75
520.1063	9.25	520.105	20.25

520.1388	12.25	520.1425	20.25
520.17	13.25	520.175	20.25
520.2025	13	520.21	17
520.2388	14.75	520.2425	19.25
520.2688	11	520.275	17.5
520.3	11.75	520.3075	17
520.3413	11.5	520.34	20.25
520.3738	12.5	520.3738	18.25
520.4038	12	520.4063	14.75
520.435	10.75	520.4425	18.25
520.4675	11.25	520.4788	19
520.5025	10.75	520.5113	21.5
520.535	10.25	520.5425	19.25
520.57	12	520.5725	19.75
520.6063	9.75	520.605	17.75
520.6388	11	520.6425	21.75
520.67	9.75	520.6763	16.5
520.7025	11	520.71	19.25
520.7363	7.25	520.7425	19.75
520.7688	9.5	520.775	20
520.8	9.25	520.8075	14.25
520.8413	12.25	520.84	18
520.8738	9.5	520.8738	13.5
520.9038	9.75	520.9063	13.5
520.935	11.75	520.9425	15.75
520.9675	12	520.9788	16.5
521.0025	10.5	521.0113	17.25
521.035	8	521.0425	14.5
521.07	9.25	521.0725	19
521.1063	5.75	521.105	19.5
521.1388	9.75	521.1425	16.5
521.17	10.5	521.175	16.75
521.2025	10.5	521.21	20
521.2363	9	521.2425	17.25
521.2688	10.25	521.275	22
521.3	7.5	521.3075	18.5
521.3413	10.25	521.34	17.5
521.3738	10.5	521.3738	21.5
521.4038	12.25	521.4063	19
521.4363	10.75	521.4425	19.5
521.4675	7.75	521.4788	19.75
521.5038	10.25	521.5113	19.5
521.535	9.75	521.5425	15
521.57	11.75	521.575	17
521.6063	11.5	521.605	26.5
521.6388	10.25	521.6425	25.75
521.67	10	521.675	20.5
521.7025	10	521.71	18.75
521.7363	9.25	521.7425	23.5
521.7688	7.75	521.775	21.25

521.8025	12.25	521.8075	18
521.8438	9.25	521.84	18.75
521.8738	8.25	521.8738	17.75
521.9038	7.75	521.9063	20.25
521.935	8.5	521.9425	20
521.9675	8.25	521.9788	18.75
522.0038	6	522.0113	17.75
522.0388	6.25	522.0425	17.75
522.07	9.5	522.0725	18.75
522.1063	8.5	522.105	23.25
522.1388	9	522.1425	20
522.17	11.25	522.175	20.5
522.2025	8.25	522.21	19.5
522.235	9	522.2425	16.25
522.2688	10.75	522.275	16
522.3025	11.75	522.3075	14.5
522.3438	10	522.34	19.75
522.3738	14.25	522.3725	24.5
522.4038	11.5	522.4063	21.25
522.4363	9.25	522.4425	19.25
522.4675	10	522.4763	19
522.5038	11.5	522.5113	21.25
522.535	9.5	522.545	23
522.57	11.25	522.5725	20.25
522.6063	9.75	522.6075	24.75
522.6388	9	522.6425	21.5
522.67	10	522.675	22
522.7025	10.75	522.7125	21.25
522.7375	12.75	522.7425	20
522.7688	13.75	522.775	23.25
522.8025	10.75	522.8075	21.25
522.8438	9.5	522.84	19.5
522.8738	7.25	522.8725	20
522.9038	11.75	522.9063	17.75
522.935	8.25	522.9425	24
522.9675	12.5	522.9763	22.75
523.0038	12.25	523.0113	20.25
523.035	10.75	523.0425	24
523.07	12.75	523.0725	21
523.1063	10.5	523.105	23
523.1388	14	523.145	16.25
523.17	14.25	523.175	20.75
523.205	12.25	523.21	16.75
523.2413	9.5	523.2425	22.25
523.2688	15.25	523.275	22.25
523.3025	12.75	523.3075	21.5
523.3438	10.5	523.34	16
523.3738	14.25	523.3738	16
523.4038	11.5	523.4063	18.5
523.435	12.75	523.4425	20

523.4675	13.75	523.4763	21.5
523.5038	12.5	523.5113	17.5
523.535	10.75	523.5425	22.5
523.5725	14.25	523.5725	18.5
523.6063	9.25	523.605	21.25
523.6388	10	523.6425	17.75
523.67	9.5	523.675	16.75
523.7025	11.25	523.71	17.5
523.7375	8.5	523.7425	21
523.7688	12	523.775	17
523.8025	12.25	523.8075	16.5
523.8438	12	523.84	21
523.8738	13	523.8738	21.75
523.9038	11	523.9063	23.25
523.935	10.75	523.9438	18.75
523.97	6.75	523.9763	18.25
524.0013	7.5	524.0113	21.5
524.035	9	524.0425	23
524.0725	9.25	524.0738	18.75
524.1063	11	524.105	16.75
524.1388	13	524.1425	22.75
524.17	9.5	524.1763	20
524.2	8.75	524.21	23.5
524.2375	9	524.2425	25.75
524.2688	11.75	524.275	21
524.305	10	524.3075	20.5
524.3475	9.75	524.34	21.5
524.3738	9.25	524.3738	19.25
524.4038	9.25	524.4063	18.75
524.435	8.75	524.4425	23.5
524.47	11.75	524.4763	22.5
524.5013	14.25	524.5113	20
524.535	11.75	524.5425	22.75
524.5725	12	524.5725	20.75
524.6063	13.25	524.605	21.5
524.6388	10.25	524.6425	21
524.67	12	524.6788	18.75
524.7	12	524.71	20.75
524.7375	10	524.7425	18.5
524.7688	15.5	524.775	22
524.805	12.5	524.8075	22.75
524.8438	11.75	524.84	23.25
524.8738	13	524.8738	23.75
524.9038	8	524.9063	19.25
524.935	13.5	524.9425	21
524.97	11.25	524.9763	18.75
525.0013	8.75	525.0113	22.25
525.035	9.25	525.0425	17
525.0725	11.5	525.0725	23.75
525.1063	10.5	525.105	16.5

525.14	11	525.1425	21.25
525.17	10.75	525.1788	21.5
525.2013	9.25	525.2088	19.75
525.2375	10.25	525.2425	20.25
525.2688	10.75	525.275	23
525.305	11.5	525.3075	24
525.3438	12.25	525.34	24
525.3738	15	525.3763	24
525.4038	9.75	525.4063	21.25
525.435	11.25	525.4425	19.5
525.47	13.75	525.4763	22.75
525.5013	13	525.5113	25.5
525.5363	15	525.5425	23.5
525.57	14.5	525.5725	23
525.6063	10	525.605	20.25
525.6388	12.5	525.6413	23.5
525.67	11	525.6788	20.5
525.7013	13.25	525.7088	19.75
525.7375	13	525.7413	24.5
525.7688	10.5	525.775	26.5
525.8063	17	525.8075	25
525.8438	14.25	525.84	21.75
525.8738	16	525.8763	20.75
525.905	19	525.9088	23.25
525.9363	9.5	525.945	20.25
525.9725	12.5	525.9788	28.25
526.0038	13.5	526.0125	25.5
526.04	10.75	526.0438	26.75
526.0725	12	526.0738	24
526.1088	14	526.1088	23.5
526.1413	11.25	526.1413	21
526.1725	12.25	526.18	19
526.2038	11.75	526.21	15
526.2388	12	526.2438	23.25
526.2688	12.75	526.2775	20.75
526.3088	11.25	526.31	21
526.3425	12	526.3425	22.75
526.375	11.75	526.3763	20.5
526.405	9.75	526.4088	20.5
526.4363	14.5	526.445	20.25
526.4725	15.25	526.4788	18.25
526.5038	13.25	526.5125	21.75
526.54	12.75	526.5438	19.5
526.5725	12.25	526.5738	20.25
526.6113	9.25	526.6088	22.25
526.6413	9.75	526.6413	13.75
526.6725	10	526.68	22.5
526.7038	13.75	526.71	18.25
526.74	11.75	526.7438	18.25
526.7688	11.75	526.7775	19.25

526.8088	10.25	526.81	19.75
526.8425	14	526.8425	16.25
526.875	12.25	526.8763	15.75
526.905	11.75	526.9088	15.75
526.9388	13.75	526.945	17.5
526.9725	11.5	526.9788	21.5
527.0038	10.5	527.0125	21
527.04	9.75	527.0438	18.75
527.0725	11.25	527.0738	14.5
527.1088	12.75	527.1088	16.25
527.1413	14.75	527.1413	16.75
527.1725	12.25	527.18	19.75
527.2063	15.25	527.21	16.75
527.2388	12.5	527.2438	25
527.2688	10.75	527.2775	20
527.3088	15.25	527.31	20.75
527.3438	13.5	527.3425	20
527.375	10	527.3763	21.25
527.405	10.25	527.41	16.75
527.4388	8.75	527.445	19.25
527.4725	11.5	527.4788	22.5
527.5038	10.75	527.5125	19.5
527.54	11.5	527.5438	17
527.5725	12.75	527.5738	14.75
527.6088	12.75	527.6088	16.5
527.6413	13.5	527.6413	17.25
527.6725	12.25	527.68	18.5
527.7038	10.5	527.71	19.75
527.7388	14	527.7438	22.5
527.7713	8.5	527.7775	20.5
527.8088	11	527.81	19.5
527.8425	12.75	527.845	17.75
527.8775	10	527.8775	17.75
527.905	8.75	527.9125	24.5
527.9388	9	527.945	20.75
527.9725	7	527.9788	23.5
528.0038	10.25	528.0125	21.25
528.0375	10	528.0425	21
528.0725	11.75	528.0738	22.25
528.1088	10.5	528.1088	19.75
528.1413	9.75	528.1413	18.75
528.1738	9.25	528.18	18.75
528.2038	12	528.21	20.25
528.2388	12.75	528.2438	20.75
528.2738	12.5	528.2775	19.25
528.3088	11.25	528.3113	21.5
528.3425	13	528.345	22.5
528.375	12	528.3775	20.75
528.4088	10	528.4125	18.25
528.4388	9	528.445	15.75

528.4725	12.75	528.4788	19.25
528.5038	13.75	528.5125	18.5
528.5375	10	528.5425	18.75
528.5725	10.75	528.5738	21.75
528.6088	10	528.6088	20.75
528.6413	13	528.6413	23.5
528.6763	9.25	528.68	19
528.7038	10.25	528.71	16.75
528.7388	10.75	528.7438	19.25
528.7738	12.75	528.7775	16.5
528.8088	14.75	528.81	17.75
528.8425	11.75	528.845	20.25
528.875	11.5	528.8775	20.75
528.905	10	528.9125	21.75
528.9388	10.25	528.945	16.25
528.9725	8.5	528.9788	20.25
529.0075	10.25	529.0138	16.25
529.0388	10	529.0425	17.75
529.0725	9.5	529.0738	21
529.1088	9.5	529.1113	17
529.1413	10.5	529.1413	21
529.1738	9	529.18	20.25
529.2038	9.5	529.21	18.25
529.2388	10	529.2438	20.75
529.2738	10.5	529.2775	20
529.3088	9.25	529.31	20
529.3425	7.25	529.345	16.25
529.375	9.75	529.3775	15.5
529.4075	9.5	529.4125	21.25
529.4388	9	529.445	15.75
529.4725	9.75	529.4788	11.75
529.5038	9	529.5125	16.5
529.5388	9.75	529.5425	14.75
529.5725	10	529.5738	20
529.6125	11.5	529.6088	14.75
529.6413	11	529.6438	15.75
529.6738	10.25	529.68	20
529.7038	8.5	529.71	20
529.7388	8.5	529.7438	19.75
529.7738	10.25	529.7775	18
529.8063	10.25	529.81	19.5
529.8413	8.25	529.845	16.75
529.875	8.5	529.8775	16.5
529.9075	7.75	529.9125	21.75
529.9388	11	529.945	21.25
529.9725	8	529.9763	18.5
530.005	10.5	530.0125	16.5
530.0388	10.5	530.0425	21.75
530.0725	12	530.0738	18.25
530.1125	11.25	530.1113	16.75

530.1413	11.75	530.1438	19.5
530.1738	14	530.18	13
530.2038	9.25	530.21	18.75
530.2388	12.25	530.2438	16.75
530.2738	12.25	530.2775	19
530.3063	12.25	530.31	23.75
530.3413	11	530.345	19.75
530.375	11.25	530.3775	21
530.4075	9.5	530.4125	19.5
530.4388	14	530.445	18.5
530.47	12	530.4763	19.25
530.5063	12	530.5125	19.25
530.5388	13	530.5425	22.5
530.5725	11.75	530.5738	23.25
530.6088	14.25	530.6088	18
530.6413	15.5	530.6438	22
530.6738	12.25	530.68	20.5
530.7063	11.75	530.71	21.5
530.7388	11.75	530.7438	20.25
530.7738	13.75	530.7775	20
530.8063	14.25	530.81	20.25
530.8413	13	530.845	21.25
530.875	8.5	530.8775	25
530.9075	12.25	530.9125	24
530.9388	11.25	530.945	25.75
530.97	11.5	530.9763	24.25
531.0063	12.5	531.0125	29.25
531.0388	14.25	531.0425	23.75
531.0725	13.25	531.0738	25.25
531.1088	14	531.1088	20.75
531.1425	16.5	531.1463	29
531.1738	12.25	531.18	22.75
531.2038	14	531.21	21.75
531.2388	10.75	531.2438	21.25
531.2738	13	531.2775	23.5
531.3063	13.25	531.31	19.75
531.3413	13	531.345	23.5
531.375	10.75	531.3775	18.75
531.4075	14	531.4113	21
531.4388	14	531.445	19.75
531.47	18	531.4763	20.25
531.5063	11.75	531.5125	25.75
531.5388	16.25	531.5425	18.25
531.5725	16	531.5738	19
531.6088	13.75	531.6088	23.25
531.6425	12.75	531.6463	26.25
531.6738	13.25	531.68	19.5
531.7038	12.5	531.71	22
531.7388	13.5	531.7438	20.75
531.7738	13.25	531.7775	17

531.8063	12.25	531.81	23.25
531.8413	10	531.845	27.5
531.875	9	531.8775	29.5
531.9075	12	531.9113	24
531.9388	7.25	531.945	20
531.97	12.25	531.9763	29
532.0063	7.75	532.0125	27.75
532.0388	10.25	532.0425	25.25
532.0738	16.5	532.0738	26
532.1113	9	532.1088	27
532.145	12.75	532.1463	25.25
532.1763	12.5	532.18	26.75
532.2063	9.75	532.21	23.25
532.2425	10	532.2438	24.75
532.2763	11.25	532.2775	22
532.3075	9.25	532.31	16.75
532.345	8.5	532.345	26.75
532.3788	13.25	532.3788	24
532.4088	9.5	532.4113	25.5
532.44	12.25	532.4475	21.75
532.4725	14.25	532.4813	23.25
532.5088	11.25	532.515	26.25
532.5413	12.75	532.545	22.25
532.5738	11.75	532.5763	24.5
532.6088	14.5	532.61	27.25
532.645	10.25	532.6475	26.75
532.6763	11.5	532.6813	24.75
532.7063	13.75	532.7113	23.25
532.7425	11.75	532.745	25
532.775	10.75	532.7788	24.25
532.8075	12.75	532.8088	21.5
532.845	14	532.8475	22.75
532.8788	11	532.8813	20.5
532.9088	11.25	532.9138	19
532.94	13.25	532.9475	21.25
532.9725	12	532.9813	22
533.0088	12.5	533.015	22.5
533.0413	16.25	533.045	26.25
533.0738	11.5	533.0763	23.75
533.1088	8.5	533.11	25.5
533.145	11.75	533.1475	23.75
533.1763	12	533.1813	19.5
533.2063	16.25	533.2113	23.5
533.245	12.75	533.245	19.75
533.275	12.25	533.2788	23.5
533.3075	12	533.3088	19.25
533.3425	14.5	533.3475	19
533.3788	12.5	533.3813	19.5
533.4088	11.25	533.4138	19.25
533.44	10.25	533.4475	20.75

533.4725	13	533.4788	21.75
533.5088	11	533.515	25.25
533.5413	11	533.5475	22.25
533.5738	10	533.5763	19.25
533.6088	10	533.61	23.5
533.645	13.75	533.6475	21
533.6763	9.75	533.6813	19.75
533.7088	9.5	533.7113	21.75
533.7463	15.5	533.745	17.25
533.7763	10.5	533.7788	21.5
533.81	13.75	533.8088	19
533.845	10.5	533.8475	23.5
533.8813	10	533.8813	19.5
533.9113	8.5	533.9138	23.25
533.9425	15.25	533.9475	21.75
533.9763	15	533.9788	23.75
534.0113	12.75	534.015	22.25
534.0438	12.25	534.045	23.75
534.075	12.5	534.0763	23.25
534.11	7.75	534.1125	22.25
534.1463	9.5	534.1475	20
534.1775	12	534.1825	23.5
534.2088	9.25	534.2113	19.75
534.2463	6.75	534.2463	20.75
534.2763	8	534.2788	20
534.31	9.75	534.3088	22.75
534.345	9.25	534.3475	23.5
534.3838	8.75	534.3813	20.75
534.4113	10.5	534.4138	18.25
534.4425	12.5	534.4475	18
534.4788	13.25	534.4788	19.25
534.5113	14.5	534.515	19.75
534.5438	15	534.545	18.25
534.575	12.75	534.5763	19.75
534.6125	11.75	534.615	23.5
534.6463	12.5	534.6475	18
534.6775	10.75	534.6813	26
534.7088	12	534.7113	20
534.7463	12.75	534.7463	19.25
534.7763	12.75	534.7788	15.75
534.81	12.25	534.8088	20.25
534.845	9.25	534.8475	17.5
534.8813	13.5	534.8813	14.75
534.9113	11.5	534.9138	17.5
534.9425	14.5	534.9475	17
534.9788	12.75	534.9788	17.75
535.0113	9	535.015	12
535.0438	11	535.045	19.25
535.075	10	535.0763	17
535.1113	11.25	535.115	16.75

535.1463	11	535.1475	21.25
535.1775	9.75	535.1788	22.25
535.2088	8.75	535.2113	14.75
535.2463	8.75	535.2463	20.5
535.2763	13	535.2788	23
535.31	9.25	535.3088	20.25
535.345	7.25	535.3488	17.5
535.3813	10	535.3825	23.25
535.4113	9.5	535.4138	18.25
535.4425	12.75	535.4475	19.5
535.4788	9.75	535.4788	23.5
535.5113	8.75	535.515	18
535.5438	10	535.545	15
535.575	9.5	535.575	19
535.6113	7.75	535.615	17.75
535.6463	10.25	535.6475	19.5
535.6775	10.25	535.6788	19.5
535.71	9.75	535.7113	16.5
535.7463	10	535.7463	18
535.7763	10.75	535.7788	17.5
535.81	8.5	535.8088	14.75
535.845	11	535.845	22.25
535.8813	7.5	535.8825	15.75
535.9113	10.5	535.9138	18.25
535.9425	11	535.9488	19
535.9788	10.75	535.9788	18.5
536.0113	9	536.015	20.25
536.0438	11.75	536.045	18.25
536.075	8.5	536.075	16.75
536.1113	9.75	536.115	15.25
536.1463	7.25	536.1475	19
536.1775	9.75	536.1788	18.75
536.21	10.5	536.2113	18
536.2463	8	536.2463	19.5
536.2763	9.5	536.2788	16.25
536.31	9.75	536.3088	17.5
536.3475	11.75	536.3463	15
536.3813	10.5	536.3825	16.75
536.4113	8	536.4138	16
536.4425	7.75	536.4475	14.75
536.4788	7.5	536.4788	16.5
536.5113	7	536.515	17
536.5438	8.5	536.545	19.5
536.575	9.25	536.575	17.25
536.6113	6.75	536.615	20.5
536.6463	8.5	536.6475	23.5
536.6775	12.5	536.6788	28
536.71	9	536.7113	36.75
536.7463	8.25	536.745	23.5
536.7763	9.75	536.7813	18.5

536.81	11.5	536.8113	23.5
536.8463	6.5	536.8488	23.5
536.8775	9.75	536.885	31
536.9113	7.25	536.9163	35
536.9425	6.5	536.9488	20.5
536.9788	6.5	536.9825	23.75
537.0113	9.25	537.0163	21.75
537.0425	10.75	537.0463	22.25
537.0775	11.25	537.0788	22.25
537.1138	6	537.1163	23
537.1488	7.5	537.1488	20
537.18	10.25	537.1813	20.25
537.2125	9.25	537.2138	25.5
537.2488	11.5	537.2475	20.5
537.2788	8.5	537.2813	19.25
537.3125	12	537.3113	16.25
537.3475	6.75	537.3488	18.5
537.3788	12	537.385	17.5
537.4125	8.75	537.4163	21.75
537.4438	10.25	537.4488	17.75
537.4825	10.25	537.4825	21.5
537.5125	7.5	537.5163	15.75
537.5438	13.25	537.5463	18.25
537.5788	13.75	537.5788	20.25
537.6138	9	537.6163	19.25
537.6488	14.75	537.6488	15.25
537.68	11.25	537.6813	19.75
537.7138	11.5	537.7138	17.5
537.7488	9.75	537.7475	15.25
537.7788	12.5	537.7813	13
537.8125	9.25	537.8113	17.5
537.8475	13	537.8488	17.5
537.8788	10.75	537.885	22.75
537.9125	13.75	537.9188	20
537.9438	11.75	537.9488	20.25
537.9825	10.25	537.9825	17.5
538.0125	12.25	538.0163	14.25
538.0438	12	538.0463	19.25
538.08	9.75	538.0788	20
538.1138	11.5	538.115	16.75
538.1488	14.25	538.1488	17.5
538.18	10.75	538.1813	19
538.2138	8.75	538.2138	18.75
538.2488	10.5	538.2475	18.5
538.2788	12.5	538.2813	17.25
538.3125	14.75	538.3113	22.25
538.3475	10.5	538.3463	19.75
538.3788	13	538.385	17.5
538.4125	13.5	538.4163	18.25
538.4438	10.75	538.4463	17

538.4825	11.5
538.5125	9.75
538.5438	12.25
538.58	10
538.6138	12.5
538.6475	10.75
538.68	10.5
538.7138	12.75
538.7488	10.5
538.7788	10.25
538.8125	12.5
538.8475	9.5
538.8788	9.25
538.9125	13.5
538.9463	9
538.9825	11.5
539.0125	11.5
539.0438	8
539.0813	11.5
539.1138	11
539.1475	13.5
539.18	14
539.2138	11.25
539.2488	10
539.2788	9.5
539.3125	12.5
539.3475	9.25
539.3788	10.75
539.4125	11.75
539.4463	12.75
539.4825	11.5
539.5125	12
539.5438	11
539.58	11
539.6138	11.5
539.6475	15
539.6838	8.75
539.7138	11.5
539.7488	15.75
539.7788	12.75
539.8138	10.5
539.8475	14
539.8788	11.75
539.91	10.75
539.9463	13.75
539.9825	15
540.0125	13.25
540.0438	13.5
540.08	11
540.1138	14.75

538.4825	16
538.5163	18.5
538.5463	18.5
538.5788	17.25
538.615	14.75
538.6488	14.75
538.6813	23.75
538.7138	19.75
538.7475	17
538.7813	19.5
538.8138	17.5
538.8463	25.75
538.885	18
538.9163	19.75
538.9463	15
538.9825	15.5
539.0163	17.5
539.0463	14.5
539.0788	16.5
539.1175	17
539.1488	17.5
539.1813	20
539.215	20.75
539.2475	18.5
539.2813	20.75
539.3138	19.75
539.3463	19
539.385	19.5
539.4163	20.5
539.4463	16
539.4825	15.25
539.5163	17
539.5463	17.25
539.5788	19
539.6175	16.75
539.6488	19.75
539.6813	20
539.715	22.75
539.7475	17.25
539.7813	17.75
539.8138	20.5
539.8463	17.25
539.885	15.75
539.9163	19.75
539.9463	20.25
539.9825	17.5
540.0163	21
540.0463	19.5
540.0788	21.25
540.1175	17.25

540.1475	12.25	540.1488	18
540.1838	13.75	540.1813	19.5
540.2138	11.5	540.215	16.25
540.2488	13.25	540.2475	19.75
540.2788	13.75	540.2813	17.5
540.3138	13.25	540.3138	17
540.3475	11.75	540.3463	15.75
540.3788	15.75	540.3825	15.75
540.41	13.5	540.4163	18.5
540.4463	11.75	540.4488	16.75
540.485	9.75	540.4825	17.25
540.5125	11.5	540.5163	16
540.5438	10.5	540.5488	16.25
540.58	9.5	540.5788	21
540.6138	13.5	540.6175	19
540.6475	10.5	540.6488	18.25
540.68	9.25	540.6813	16.5
540.7138	9.25	540.715	16.25
540.7488	11	540.7475	17.75
540.7788	10	540.7813	20.75
540.8138	9	540.8138	22
540.8475	9.75	540.8463	20
540.8788	11	540.8825	20.25
540.91	11.5	540.9163	25
540.9463	10.25	540.9488	20.5
540.9813	6.75	540.9825	19.75
541.0125	9.25	541.0138	22.25
541.0463	11.5	541.0488	19
541.08	8.75	541.0788	18
541.1138	9.5	541.115	17.75
541.1475	10.5	541.1488	19.5
541.18	9	541.1813	18.75
541.2138	11.25	541.215	17.75
541.2488	11.25	541.2475	18.25
541.2788	8.75	541.2813	18.25
541.3138	10.75	541.3138	15
541.3475	13.25	541.3463	21.25
541.3788	12.25	541.3825	16.75
541.41	11.75	541.4163	15.75
541.4463	13.75	541.4488	18.5
541.4813	9	541.4825	18.75
541.5125	8.75	541.5163	16
541.5463	11.75	541.5513	15.5
541.58	10	541.5813	18
541.6138	8	541.6163	20.75
541.6475	9.25	541.6513	17.75
541.6775	11	541.685	23
541.7138	9.5	541.7175	20.25
541.7488	12	541.7488	23.5
541.7788	11.25	541.785	17

541.8138	12.5	541.815	15
541.8475	10.75	541.85	24
541.8813	10.5	541.8863	19.25
541.91	12.75	541.9175	18
541.9463	12.25	541.95	22.25
541.9813	10	541.985	20.75
542.0125	13.25	542.0163	20.5
542.0463	12.25	542.0513	18
542.08	12.75	542.0813	20.75
542.1138	13.5	542.1163	21.25
542.1475	17.5	542.1513	20.75
542.1775	13.5	542.185	17.75
542.2138	13.25	542.2175	17.5
542.2488	10.25	542.2488	19.25
542.2788	10.75	542.2838	18.75
542.3113	14.5	542.315	16.5
542.3475	10.75	542.3488	18.25
542.3788	10	542.3888	19
542.41	10.25	542.4175	20.25
542.4463	10.5	542.45	16.75
542.4825	9	542.485	18.75
542.515	11.5	542.5163	21
542.5463	9.75	542.5513	22.75
542.58	11.5	542.5813	15.25
542.6138	8.25	542.6163	16.5
542.6475	10.75	542.6513	20.25
542.6775	8.25	542.685	20.5
542.7138	12	542.7175	18.75
542.7488	11	542.7488	15.5
542.78	9	542.7838	21.25
542.8113	10.75	542.815	17.75
542.8475	8.25	542.8513	16
542.88	10.5	542.885	17
542.91	9.25	542.92	18.25
542.9488	7.75	542.95	18.25
542.9813	9.75	542.985	18.25
543.0125	11.5	543.0163	18
543.0463	8.75	543.0513	19
543.0813	8.75	543.0813	17.5
543.1138	9	543.1163	19.75
543.1475	11	543.1513	17.5
543.1775	8.75	543.185	18.75
543.2138	15.25	543.2175	18.5
543.2488	10	543.2488	20
543.28	12.75	543.2838	16.25
543.3113	11	543.315	20
543.3475	11.75	543.3525	20.5
543.38	14.75	543.385	17.75
543.41	12.75	543.42	17.75
543.4488	12.25	543.45	15.25

543.4813	10.75	543.485	16.25
543.5125	10	543.5188	20.25
543.5463	13.25	543.5513	19.75
543.58	11	543.5813	19.5
543.6138	12.25	543.6163	17.75
543.6475	12.25	543.6513	16.5
543.6775	12	543.6875	20
543.7138	9	543.7175	19.75
543.75	9	543.7488	21.25
543.78	12	543.7838	21
543.8113	12.5	543.815	21.75
543.8475	8.5	543.8525	18.75
543.88	9.25	543.885	15.75
543.91	12.75	543.92	16.75
543.9475	11.75	543.95	23.25
543.9813	12.75	543.985	19.25
544.015	16.5	544.0188	17
544.0463	12.5	544.0513	16
544.08	12.25	544.0813	21.5
544.1138	12	544.1163	16.25
544.1488	12.75	544.1513	16.25
544.1775	9	544.185	17.5
544.2138	11	544.2175	20.5
544.2488	12	544.2488	17.75
544.28	10.25	544.2838	16.75
544.3113	9.5	544.315	16.25
544.3475	14.75	544.3525	19
544.38	13	544.3838	22.75
544.41	12.5	544.42	24.25
544.4475	11.25	544.45	16.25
544.4813	8.5	544.485	19
544.515	12.25	544.5188	19.25
544.5463	10.25	544.5513	19.5
544.58	9.75	544.5813	19
544.6138	10.25	544.6163	15.75
544.6475	12.75	544.6525	15.5
544.6775	9.5	544.685	17
544.7138	11.5	544.7175	19.75
544.75	13.25	544.7488	15.5
544.78	9	544.7838	15.5
544.8113	13.25	544.815	17.25
544.8475	11.75	544.8525	17.5
544.88	13.75	544.8838	18
544.91	9.75	544.92	16.5
544.9475	11.5	544.95	17.25
544.9813	12.75	544.985	16.25
545.015	10.25	545.0188	14.25
545.0463	9	545.0513	18.5
545.0788	9.75	545.0813	14.5
545.1138	9.25	545.1163	16.25

545.1475	9.75	545.1513	16
545.1775	9	545.185	14.75
545.2138	8.75	545.2175	15
545.25	9.75	545.2488	17.75
545.28	11.75	545.2838	14.75
545.3113	7.75	545.315	18
545.3513	14	545.3525	18.75
545.38	11.5	545.385	18.25
545.41	10.75	545.42	19
545.4475	11.5	545.45	21.5
545.4813	10	545.485	22.5
545.515	13.25	545.5188	25.5
545.5463	11.25	545.5513	18.5
545.5788	11.75	545.5813	23
545.6138	8.25	545.6163	21
545.6475	12.25	545.6513	26.25
545.6775	12	545.685	21
545.7138	12.5	545.7175	25.25
545.7475	13.5	545.75	18.25
545.78	11.25	545.7838	20.75
545.8113	12	545.815	18.25
545.8475	11.75	545.8525	21.25
545.88	13	545.885	22.25
545.91	9	545.92	24.75
545.9475	10.75	545.95	22
545.9813	9.75	545.985	25.75
546.015	10.75	546.0188	21
546.0463	9.5	546.0513	23.25
546.0788	12	546.0813	20.75
546.1138	6.75	546.1163	26
546.15	9	546.1513	20.25
546.18	11	546.185	23.25
546.2138	9	546.2175	18.5
546.2475	8.5	546.25	16
546.28	8.75	546.2838	21.5
546.3113	10	546.315	16.75
546.3475	9.75	546.3525	19.5
546.38	9.25	546.385	20.75
546.41	11.25	546.42	19.5
546.4475	12	546.45	16.5
546.4813	14.5	546.485	18.25
546.515	9.75	546.5225	14.75
546.5463	10.5	546.5513	15.25
546.5788	12	546.5813	17
546.6138	10.25	546.6188	16.75
546.6475	11	546.6513	24
546.68	10.5	546.685	20
546.7113	12.25	546.7175	19.25
546.7475	7.75	546.7525	23
546.78	12.5	546.7863	22.5

546.8113	12.25	546.8175	19.75
546.8475	9.75	546.8538	20.5
546.88	9.5	546.8863	22
546.91	14	546.9213	21
546.9475	12.75	546.9538	21.5
546.9838	10.25	546.9863	19.25
547.0175	12	547.0225	22.75
547.0488	13	547.0525	21
547.0813	10.75	547.0838	21.25
547.1175	13	547.1213	20
547.15	11.5	547.1538	17.75
547.1838	14.75	547.1875	18.5
547.2138	10.75	547.22	16.5
547.2488	12	547.2525	21
547.2813	7.25	547.2863	18.75
547.3125	11	547.3175	17.75
547.35	12	547.3525	19.5
547.3825	12.5	547.3863	15.75
547.4125	14.25	547.42	15
547.4488	8.75	547.4538	16.5
547.4863	11.25	547.4888	17
547.5175	13	547.5225	20
547.5488	10.25	547.555	14.5
547.5813	8.25	547.5838	18.25
547.6175	12	547.6213	14.25
547.6488	12.75	547.6513	14
547.6838	11.75	547.6875	17.75
547.7138	10.25	547.72	21.5
547.7513	9	547.7525	15.5
547.7813	11	547.7863	19.5
547.8125	8.75	547.8175	19.25
547.8475	7.75	547.8525	17.75
547.8825	10.25	547.8863	16.75
547.9125	10.25	547.92	15
547.9488	14.5	547.9538	17.5
547.9863	9.25	547.9888	20.75
548.0163	7.5	548.0225	20.5
548.0488	13.25	548.0525	22.25
548.0813	9.75	548.0863	25.75
548.1175	8.75	548.1238	18.5
548.1488	9.25	548.1538	22
548.1838	9.5	548.19	26.75
548.2163	11.25	548.2225	20.5
548.2488	9.75	548.2563	25.75
548.2813	7.5	548.2888	23.75
548.3125	9.5	548.32	29.25
548.3475	10.75	548.355	28.5
548.3825	8.5	548.3875	26.75
548.4125	8.5	548.4213	26.75
548.4488	9.75	548.455	25.75

548.4863	9	548.4913	23
548.5163	9	548.5238	21.25
548.5488	8.75	548.5538	21.5
548.5813	9	548.5863	21.25
548.6175	10.75	548.6238	27
548.6488	7.25	548.6538	23.75
548.6838	8.75	548.69	25
548.7163	7.75	548.7225	26.5
548.7538	9	548.7563	21.5
548.7813	10	548.7888	23.75
548.8125	10.75	548.82	25
548.8475	10	548.855	21.5
548.8825	12.25	548.8875	25.75
548.915	11.75	548.9238	25
548.9488	9	548.9538	24
548.9863	12.5	548.9913	25.75
549.0163	10	549.0238	26
549.0488	12.75	549.0538	19
549.0838	14.25	549.0863	19.75
549.1188	18	549.1238	24.25
549.15	13	549.1538	24.25
549.1863	15	549.19	21.75
549.2188	13.5	549.2225	24
549.2513	17	549.2563	21
549.2838	13.75	549.2888	22.75
549.315	14.5	549.32	23.5
549.3525	20	549.355	23.5
549.3863	15.25	549.3875	26.75
549.4188	11.5	549.4213	22.75
549.4525	16.25	549.4538	22.75
549.4888	14.75	549.4913	22.75
549.52	14.75	549.5238	26
549.5525	15.25	549.5538	22.25
549.5875	16.25	549.5888	28
549.6213	14.5	549.6238	24.25
549.6525	13.5	549.6538	23.5
549.6888	17.5	549.69	22.75
549.7213	12.75	549.7238	24.25
549.7538	12.75	549.7563	25.25
549.7863	11.25	549.7888	24.25
549.8163	12.25	549.82	23.75
549.8525	14.5	549.855	24.25
549.8863	12.25	549.8875	23.75
549.9188	11.75	549.9213	21.5
549.9525	14	549.9538	20.5
549.9888	11.75	549.9913	18
550.0188	15.25	550.0238	23
550.0525	10.25	550.0538	21.25
550.0875	10.5	550.0888	20.25
550.1213	11.25	550.1238	19.25

550.1525	11.75	550.1538	19.25
550.1888	11	550.1875	22.25
550.2213	12.25	550.2238	23.5
550.2538	11.75	550.2563	19.5
550.2838	13.75	550.2888	22.5
550.3163	12.75	550.32	20.5
550.355	13	550.355	20.25
550.3863	16.5	550.3875	21.75
550.4188	14.5	550.4213	25
550.4525	13	550.4538	24
550.4888	14.5	550.4925	23
550.5188	15	550.5238	26.25
550.5525	11	550.5538	22.25
550.5875	13.75	550.5888	19
550.6213	11	550.6238	23.25
550.6525	11.75	550.6563	21.75
550.6913	12.75	550.6875	24
550.7238	10.5	550.7238	22
550.7538	11.5	550.7563	23
550.7838	13.25	550.7888	21.75
550.8163	11.5	550.8213	20.25
550.8525	12.5	550.855	23.25
550.8863	12.25	550.8875	20.5
550.9188	10.5	550.92	25.5
550.9525	11.25	550.9538	21
550.9888	13.5	550.9925	22
551.0188	10.75	551.0238	21.25
551.0525	10	551.0538	24
551.0875	11.5	551.0888	20.25
551.12	9.75	551.1213	22.5
551.1525	13.75	551.1538	26.25
551.1888	16.5	551.1875	24.75
551.2213	10.75	551.2238	25.5
551.2538	10.75	551.2563	21.75
551.2838	11	551.2888	23.75
551.3188	12.75	551.3213	23
551.3513	10.5	551.355	21.5
551.3863	12.75	551.3875	19.5
551.4188	9.25	551.42	22
551.4525	11.5	551.4563	19.5
551.4888	10.5	551.4925	19.75
551.5188	9.25	551.5238	22.75
551.5525	10.25	551.5538	20
551.5875	9.5	551.59	21.75
551.62	13.75	551.6213	17.75
551.6525	9	551.6538	21
551.6888	13.75	551.6875	20.5
551.7213	12.25	551.7238	18
551.7538	9.5	551.7563	18.75
551.7838	11.25	551.7888	17

551.8163	11.25	551.8213	19
551.8513	10.5	551.855	18.5
551.8863	11.75	551.8875	19
551.9188	12.75	551.92	19.75
551.9525	10.25	551.9563	15.5
551.9888	11.75	551.9925	22.75
552.0188	16.25	552.0238	19.75
552.0513	11.5	552.0563	18.25
552.0875	10.75	552.09	21
552.12	12.25	552.1213	20.5
552.1525	7.75	552.1538	20.5
552.1888	11	552.1875	22.5
552.2213	12.25	552.2238	19.25
552.2538	10.75	552.2563	16.5
552.2838	9.5	552.2888	17.25
552.3188	11	552.3213	19.25
552.3513	11	552.355	15.75
552.3863	13.75	552.3875	19
552.42	11	552.42	15.5
552.4525	16.25	552.455	19.75
552.4888	12.25	552.4925	17.25
552.5188	10.25	552.5238	20.75
552.5513	11	552.5563	22
552.5875	12.75	552.59	18.5
552.62	13	552.6213	16
552.6525	12.25	552.6538	16.5
552.6888	13	552.6875	18.25
552.7213	14	552.7263	12.25
552.7538	11.75	552.7563	19
552.7838	15.5	552.7888	16.75
552.8213	9.75	552.8213	19.5
552.8513	16	552.855	16
552.8863	13.5	552.8875	15.75
552.92	15.75	552.92	16.75
552.9525	14.75	552.955	15
552.9888	16.5	552.9925	16.25
553.0188	13	553.0238	18
553.0513	14.5	553.0563	16.75
553.0875	10.25	553.09	17.5
553.12	9.75	553.1238	15.25
553.1525	13.25	553.1538	15.5
553.1888	11.75	553.1888	16.25
553.2213	11	553.2263	16
553.2538	9.5	553.2563	19.75
553.2838	14.75	553.2888	14.75
553.3163	12	553.3213	17
553.3513	11.5	553.355	16.75
553.3863	9.25	553.3863	18.5
553.42	9.25	553.42	16.5
553.4525	8.75	553.455	18

553.4888	7.25	553.4925	19
553.5188	8	553.5238	13.75
553.5513	10.75	553.5563	17.25
553.5875	13	553.59	17.75
553.62	9.75	553.6213	15.5
553.655	10.5	553.6538	15.75
553.6888	14.25	553.6888	17.5
553.7213	11.75	553.7263	19.25
553.7538	13	553.7563	17.75
553.7838	10.25	553.79	20.25
553.8163	13.5	553.8213	14.5
553.8513	11.75	553.855	18.75
553.8863	12.5	553.8863	21.5
553.92	10.5	553.92	15
553.9525	12.25	553.955	17.5
553.9888	12.5	553.9925	17
554.0188	13.75	554.0238	21.5
554.0513	13.25	554.0563	17.5
554.0875	12	554.09	23
554.12	15.75	554.1213	19.75
554.155	14.75	554.1538	20.5
554.1888	10.5	554.1888	24.25
554.2238	11.75	554.2263	25.5
554.2538	14.75	554.2563	22.75
554.2838	12.75	554.29	20.25
554.3163	12.75	554.3213	20.5
554.3513	13.5	554.355	18.25
554.3863	11.75	554.3863	20.25
554.42	13	554.42	18
554.4525	13.25	554.455	17.5
554.4888	13.75	554.4925	17
554.5188	16.5	554.5238	23.75
554.5538	10.5	554.5563	21.25
554.585	14.5	554.59	15.25
554.62	12	554.6213	15
554.655	9.75	554.6538	17.25
554.6888	11.75	554.6888	18
554.7238	11	554.7213	17.75
554.7538	10.25	554.7563	19.75
554.7838	13.25	554.79	17.75
554.8163	12.75	554.8213	16.25
554.8513	10.75	554.855	18
554.8863	11	554.8863	20.5
554.92	9.75	554.92	18.5
554.9525	9.25	554.9575	17
554.9888	12.25	554.9925	18.75
555.0188	12.5	555.0238	22.25
555.0538	10.75	555.0563	22.25
555.085	8.75	555.09	18
555.12	13	555.1213	24

555.155	9.5	555.1513	19.75
555.1888	10.75	555.1888	24
555.2213	10.75	555.2238	24.5
555.2538	14.75	555.2563	24
555.2838	8.75	555.29	19.25
555.3163	11	555.3238	21.75
555.3513	14.75	555.355	20
555.3863	10.75	555.3863	23.25
555.42	11	555.4225	20.25
555.4525	9.75	555.4575	20.75
555.4913	10.25	555.4925	22
555.5188	12	555.5238	20
555.5538	11.75	555.5563	21.75
555.585	12.25	555.59	22.75
555.62	11	555.6238	21
555.6563	13.5	555.6513	20
555.6888	14.5	555.6888	21.25
555.7213	14.5	555.7213	18
555.7538	11.25	555.7563	18.75
555.7838	9.5	555.79	19.25
555.8163	9	555.8238	21.25
555.85	12.25	555.855	18.75
555.8875	10.5	555.8888	16.75
555.9225	10.25	555.925	22.5
555.9525	10	555.96	22
555.9888	11.25	555.995	18.5
556.0188	10.5	556.0288	17.5
556.0538	9.25	556.0588	14.5
556.085	10.5	556.0913	17
556.12	10.75	556.1225	17.25
556.1563	9.5	556.1525	19.5
556.1888	13.25	556.1925	14.75
556.2213	13	556.2225	15.25
556.2538	12.75	556.2575	13.25
556.285	15.5	556.2913	17.75
556.3163	14	556.3263	15.5
556.3513	10.75	556.3575	15.5
556.3875	11.75	556.3888	15.75
556.42	15.75	556.425	17
556.4525	13.75	556.46	15
556.4888	10.5	556.4925	15
556.52	11.75	556.5288	14.5
556.5538	13.25	556.5588	17
556.585	10.25	556.5913	18.25
556.62	10	556.6225	15.5
556.6563	10.75	556.6525	14
556.6888	12	556.6925	19
556.7213	8.75	556.7225	19
556.7538	9.25	556.7575	16.5
556.785	9.25	556.7938	17

556.8163	10.25	556.8263	18.5
556.8513	8.75	556.8575	12.25
556.8875	10	556.8888	22.5
556.92	9.25	556.9238	21.5
556.9513	10	556.9613	20.5
556.9888	11	556.9925	15.5
557.0188	8	557.0288	18.5
557.0538	11	557.0588	17.25
557.085	10.5	557.0913	16.75
557.1175	14.5	557.1225	15.25
557.1563	12.75	557.1525	18.75
557.1888	12.5	557.1925	16.25
557.2213	9	557.2225	14.5
557.2538	10.25	557.2575	15.25
557.285	12.25	557.2913	17
557.3163	11.25	557.3263	19.75
557.35	10.25	557.3575	17.75
557.3875	10.25	557.3888	15.25
557.42	14.5	557.4238	16.75
557.4513	12.25	557.46	17.75
557.4888	11.75	557.4925	21.75
557.5188	6.5	557.5288	17.5
557.5538	11.25	557.5588	16
557.585	8.75	557.5913	18.75
557.62	9	557.625	18.25
557.6563	11	557.6575	19.5
557.6888	8.25	557.695	16.5
557.7238	9.75	557.725	20.75
557.7538	8.75	557.76	19.5
557.785	10.25	557.7925	22.25
557.8163	8.25	557.8275	16.5
557.85	10.25	557.8588	16.5
557.8875	7.75	557.89	18.5
557.92	10.25	557.9275	14.75
557.9513	10	557.9613	21.25
557.9888	9.75	557.9938	18.25
558.0188	8.5	558.0313	14.5
558.0538	11.75	558.0613	22
558.085	12	558.0938	17.25
558.12	11.5	558.125	22.75
558.1563	12.75	558.1575	22.75
558.1888	13.5	558.195	21.25
558.2238	9.5	558.225	20
558.2538	12.5	558.2588	23
558.285	10	558.2925	19.25
558.3163	11.25	558.3275	22.25
558.35	9	558.3588	18.75
558.3875	8.75	558.39	19.25
558.42	12	558.4275	18.25
558.4513	7.5	558.4613	23

558.4888	13.25	558.4938	19.25
558.5213	9.5	558.5313	17.75
558.5538	10.5	558.5613	19.25
558.585	12	558.5938	15.25
558.62	11.75	558.625	18
558.6563	13	558.6575	20.5
558.6888	11.75	558.695	22.75
558.7238	13	558.725	20.25
558.7538	11	558.7588	21
558.785	11	558.7925	21.5
558.8163	11	558.8275	17.25
558.85	10	558.8613	22.5
558.8863	9.25	558.89	18
558.92	8	558.9263	19
558.9513	8.25	558.9613	17.75
558.9888	8.5	558.9938	20.25
559.0213	7.5	559.0313	18.75
559.0538	10.25	559.0625	15.25
559.085	9.75	559.0938	20.5
559.12	8.5	559.125	15.5
559.1563	10.25	559.16	16.5
559.1888	10.25	559.195	15.75
559.2238	11.25	559.225	15
559.2538	11.75	559.2613	17
559.285	13.25	559.2925	17.25
559.3163	15	559.3275	15.25
559.3525	11.25	559.3588	18.5
559.3863	13	559.39	18.75
559.42	14.5	559.4263	16.25
559.4513	11	559.4613	24
559.4888	12.25	559.4925	17.5
559.5213	11.75	559.5313	17.5
559.5538	10.75	559.5625	18
559.585	11.75	559.5938	19.25
559.62	15	559.625	22
559.6563	13.75	559.66	17.75
559.6888	12.5	559.695	13.75
559.7238	13.25	559.725	20.5
559.7538	13.25	559.7613	15
559.785	12.75	559.7925	14
559.8163	17.5	559.8275	14.75
559.8538	14	559.8588	15
559.8863	12.5	559.8913	15.25
559.92	13.25	559.9263	15.75
559.9513	13	559.9613	15.5
559.9888	12.25	559.9925	13
560.0213	14.75	560.0288	15.25
560.0538	13.25	560.0625	15.5
560.085	13.5	560.0938	17.25
560.1175	12	560.125	15.25

560.1538	10.5	560.16	13.5
560.1888	14	560.195	14.75
560.2213	13.5	560.225	14.5
560.255	12.75	560.2613	15.25
560.285	12.75	560.2925	14.25
560.3163	11.5	560.3275	15.75
560.3525	14	560.3588	14.25
560.3888	12.5	560.3913	15.5
560.4263	14	560.4263	16.5
560.455	12.75	560.4613	14.25
560.495	13.75	560.4925	14.75
560.525	12.5	560.5288	13.25
560.5575	13.5	560.5625	15.25
560.5888	11.75	560.5938	17.25
560.6213	8.25	560.625	16.25
560.6588	10.25	560.66	17.75
560.6938	7.75	560.6925	17
560.7263	10.75	560.725	17.75
560.7588	9.25	560.7613	17.25
560.79	9.5	560.7925	20.5
560.8225	9.25	560.8275	16.5
560.8563	11.25	560.8588	16
560.8925	11.75	560.8925	15.25
560.9238	10	560.9263	16.25
560.9563	11	560.9613	20.5
560.995	10	560.9925	21.5
561.025	11.5	561.0288	17
561.0575	10.5	561.0625	18.75
561.09	9	561.0938	16.5
561.1213	14.25	561.125	18.25
561.1588	12	561.16	20
561.1925	13	561.1925	19
561.2263	12	561.225	15.5
561.2588	9.75	561.2613	16.5
561.29	13.25	561.2925	18.25
561.32	12.5	561.3275	14.75
561.3563	12.75	561.3588	15
561.3925	12.5	561.3925	18.25
561.4225	8.5	561.4263	21
561.4563	13.25	561.4613	20.25
561.495	10.75	561.4925	20.5
561.525	10	561.5288	23
561.5575	9.5	561.5625	24.5
561.59	7.5	561.5938	21
561.6213	11	561.625	28.75
561.6588	11	561.66	24.25
561.6925	10.75	561.6925	24
561.7288	9.25	561.725	23.25
561.7588	12	561.7613	22.25
561.79	11.75	561.7913	21.25

561.82	10.5	561.8275	20.25
561.8563	11	561.8588	21.25
561.8925	10.75	561.8925	21.5
561.9225	11.5	561.9263	24.75
561.9563	11.75	561.9613	19.75
561.995	10.25	561.9925	21.75
562.025	10	562.0288	25
562.0575	10	562.0625	22.75
562.09	13.75	562.0938	21
562.1213	11.5	562.125	18
562.1588	10.25	562.16	19.25
562.1925	13	562.1925	20.5
562.2263	10.5	562.225	20
562.2588	10.75	562.2613	23
562.29	11.5	562.2913	18.5
562.32	14.5	562.3275	21.5
562.3563	12.75	562.3588	18.75
562.3925	9.75	562.395	23.75
562.4225	13.25	562.4288	13.25
562.4588	12.25	562.4625	22.25
562.495	9.5	562.4963	15
562.525	12.5	562.5313	23.5
562.5575	9.75	562.565	21
562.59	11	562.595	18
562.6213	9.25	562.6263	17.75
562.6613	9	562.6613	17.5
562.6975	11.5	562.695	18.25
562.7313	9.75	562.7275	17.75
562.7613	12.5	562.7625	16
562.7925	8.25	562.7963	19.5
562.8225	9	562.83	16.25
562.8575	7.25	562.8613	16.75
562.8938	8.5	562.895	14.75
562.9238	8.25	562.9288	19.5
562.96	6.75	562.9613	17.75
562.9963	8.5	562.9963	19.25
563.0263	8	563.0313	16
563.0588	12	563.065	18.5
563.0925	10	563.095	16.5
563.1238	10.25	563.1288	19.75
563.1613	9.5	563.1613	19
563.195	8	563.195	18.25
563.2288	9.5	563.2275	21
563.2613	9.75	563.2625	19.5
563.2925	11.75	563.295	18.75
563.325	8.5	563.33	21.75
563.3575	10.25	563.3613	17
563.3925	10.75	563.395	19.75
563.4238	10	563.4288	17.25
563.46	8.5	563.4613	16.75

563.4963	11	563.4963	19.25
563.5263	8.75	563.5313	15
563.5588	10.25	563.5625	17.75
563.5925	9	563.595	19.5
563.6238	12.5	563.6313	19
563.6613	9.75	563.6613	17.25
563.695	10.75	563.695	17.25
563.7288	7	563.7275	16.25
563.7613	10.25	563.7625	18.5
563.7925	9	563.795	15.25
563.825	6	563.83	16
563.8575	8	563.8613	20
563.8925	11.75	563.895	17.5
563.9238	7.25	563.9288	19
563.96	9.75	563.9613	17.75
563.9963	10.5	563.9963	18.75
564.0288	10.5	564.0313	18
564.0588	13	564.0625	21.75
564.0925	10.75	564.095	20.75
564.1238	10.5	564.1313	18.75
564.1613	9	564.1613	19.5
564.195	10.25	564.195	15.75
564.2288	10.75	564.2275	18.5
564.2613	12	564.2625	19.25
564.2925	10.5	564.295	16.75
564.325	9.25	564.33	17.5
564.3575	6.5	564.3613	19.25
564.3925	6.75	564.395	18.5
564.4263	8.75	564.4288	18.5
564.46	7.5	564.4613	20.5
564.4963	11.5	564.4938	18.75
564.5263	8.5	564.5313	17
564.5613	8.75	564.5625	20.25
564.5925	7.75	564.595	14.5
564.6238	8.75	564.6313	20.75
564.6613	8.5	564.6613	20
564.6963	10	564.695	18.5
564.7288	8	564.7275	21.5
564.7613	8.75	564.7625	14.5
564.7925	7.5	564.795	21.25
564.825	8.75	564.83	16.25
564.8575	5.5	564.8613	20.25
564.8925	9.75	564.895	18
564.9263	8	564.9288	20
564.9588	10.25	564.9613	17.75
564.9963	10.75	564.9963	22.25
565.0263	9.75	565.0338	19.75
565.06	13.75	565.0663	27.5
565.0925	10.75	565.0975	20.25
565.125	10	565.1338	18.25

565.1613	8	565.1638	22.5
565.1963	9.75	565.1975	16.5
565.2288	12.75	565.23	19.75
565.2613	10.75	565.2613	18.75
565.2925	9.75	565.2963	16.75
565.325	9.25	565.3325	19.25
565.3575	10	565.3625	19.5
565.3925	10.5	565.3963	18.5
565.4263	10	565.43	22
565.4588	10.5	565.4625	19.25
565.495	10	565.4963	21.25
565.5263	7.75	565.5338	18.5
565.5613	10.5	565.5663	16.25
565.5925	8.25	565.5975	18.75
565.625	10.25	565.6338	16.75
565.6613	10	565.6638	17.75
565.6975	9.75	565.6975	20.5
565.7288	8.75	565.73	14
565.7613	8.75	565.7613	13.25
565.7925	7.75	565.7963	17.25
565.825	8	565.8325	16
565.8575	10.75	565.8638	16.25
565.8925	8.25	565.8963	18.75
565.9263	8.5	565.93	14.5
565.9588	10.5	565.9625	13.5
565.995	8.5	565.9963	16.75
566.0263	8.25	566.0338	15
566.06	10.5	566.0663	13.75
566.0925	9	566.0975	15.75
566.125	11	566.1338	22.75
566.16	10.75	566.1638	16.5
566.1963	11.25	566.1975	14.25
566.2313	11.75	566.2275	14.5
566.2613	9.75	566.2613	16.75
566.2925	10.5	566.2963	17
566.325	9	566.3325	16.5
566.3575	13	566.3638	19
566.3925	9	566.3963	17.25
566.4263	10.5	566.43	23.5
566.4588	8.5	566.4625	18
566.495	10.25	566.4963	16.25
566.5263	8.75	566.535	17
566.56	8.5	566.5663	17
566.5925	8.5	566.5975	17.25
566.6263	10.25	566.6338	17
566.66	10.5	566.6638	25.5
566.6963	9	566.6975	16.75
566.7263	12.5	566.7275	18.75
566.7613	10.75	566.7613	18
566.7925	10.25	566.7963	20.75

566.825	11.5	566.8325	17.75
566.8575	11	566.8638	19.5
566.8925	10	566.8963	15
566.9263	10	566.93	18.75
566.9588	8	566.9625	15
566.995	13	566.9963	19
567.0263	10	567.035	19.25
567.06	7	567.065	17.75
567.0925	8.5	567.0975	17.5
567.1263	11.75	567.1338	17
567.16	10.25	567.1638	16.25
567.1963	11.5	567.1975	15.25
567.2263	10.25	567.2275	16.75
567.2613	8.25	567.2638	19.75
567.2925	7.75	567.2988	13.75
567.325	8	567.335	9.75
567.3575	9.25	567.3663	16
567.3925	7.5	567.4	13.75
567.4288	11.75	567.4338	14.75
567.4588	10.75	567.465	16
567.495	13.75	567.4988	15.5
567.5263	12.75	567.5363	13.5
567.56	11.25	567.5663	13.5
567.5925	8	567.5988	14.75
567.6263	12.75	567.6338	10.75
567.66	13.75	567.6675	15.75
567.6963	12	567.6988	14.5
567.7288	12	567.7288	14
567.7588	14.5	567.7638	15.75
567.795	9.75	567.7988	14.25
567.825	8.5	567.835	15.5
567.8575	10.5	567.8663	15.75
567.8925	12	567.8975	15.5
567.9288	11.5	567.9338	18.5
567.9588	8.75	567.965	18
567.995	9.5	567.9988	16
568.0288	15	568.0363	17.75
568.06	11	568.0688	19
568.0925	14.25	568.1038	20
568.1263	12	568.1375	17.5
568.16	10	568.17	18.75
568.1963	12	568.2013	18.75
568.2263	10.5	568.2313	17
568.2588	12.5	568.2663	16.5
568.295	10.5	568.3013	17.5
568.325	16.75	568.3363	15.25
568.3575	9.75	568.3675	19
568.3975	11.5	568.4	19.5
568.4288	8.5	568.435	19
568.4588	8.75	568.4663	18.25

568.495	9.5	568.5	21.25
568.5263	10.5	568.535	21.5
568.56	8	568.5688	15.5
568.5925	8.75	568.6038	13.75
568.6263	8.5	568.6375	17.75
568.66	9.25	568.67	19
568.6963	9.5	568.7013	16.25
568.7263	8.5	568.7313	14.25
568.7588	9.25	568.7688	18.75
568.795	7.75	568.8013	17
568.825	11.25	568.8363	17
568.8575	9	568.8675	19.75
568.895	9.75	568.9	23
568.9288	11.25	568.9375	23.75
568.9588	9.25	568.9663	16.5
568.9925	11.75	569	19.75
569.0263	10	569.035	16.75
569.06	8.25	569.0688	20.75
569.0925	8.5	569.1038	16.25
569.1263	9.25	569.1375	16
569.16	8	569.17	20.75
569.1963	9.5	569.2013	15.75
569.2263	11.75	569.2313	18.25
569.2588	8	569.2688	17.75
569.295	10.75	569.3013	17.5
569.325	8	569.3363	20.75
569.3575	11.75	569.3675	18.25
569.395	10	569.4	19.25
569.4288	9	569.435	15
569.4588	9.25	569.4675	18.5
569.4925	11.75	569.5	19.5
569.5263	11	569.535	17.75
569.56	9.5	569.5688	15.25
569.5925	13.25	569.6038	16.5
569.6263	13	569.6375	22
569.66	10	569.67	16.5
569.6963	12.75	569.7013	19
569.7263	11.5	569.7313	17.25
569.7588	12.5	569.7688	18.5
569.795	9	569.8038	19
569.825	11.25	569.8363	22
569.8575	10.75	569.8675	20.75
569.895	16.25	569.9	16.25
569.9288	13.5	569.935	21.25
569.9588	13.5	569.9663	23.75
569.9925	12.5	570	21
570.025	11.25	570.035	23
570.06	13.25	570.0688	17.25
570.0925	11.25	570.1038	16
570.1263	9	570.1375	21.75

570.16	10.75	570.17	15.75
570.1963	10.75	570.2013	18.75
570.2263	10.75	570.2313	14.75
570.2588	10.5	570.2688	19.25
570.295	10	570.3013	17.75
570.325	13	570.3375	17
570.36	9.75	570.3675	17.75
570.395	10.25	570.4	15.5
570.4288	14.75	570.435	18.25
570.4588	12.5	570.4663	14.75
570.4925	10.25	570.4988	18.5
570.5263	12.75	570.535	19
570.56	14.25	570.5688	19
570.5925	13	570.6038	18.75
570.6275	12.5	570.6375	14.75
570.6613	10.75	570.67	16
570.6963	14	570.7013	14.25
570.7263	12.5	570.7313	14.75
570.7575	12.5	570.7688	14.75
570.795	11.75	570.8013	11.25
570.825	10	570.8375	12
570.86	10.5	570.8675	13.5
570.895	12	570.9	11.25
570.9288	9	570.935	14
570.9588	10.25	570.9663	15.75
570.9925	11	570.9988	16
571.0263	15.25	571.035	14.25
571.06	11.75	571.07	13.75
571.0925	12.5	571.1038	12
571.1275	12.25	571.1363	16
571.1613	12.25	571.17	16.5
571.1963	10.25	571.2013	19
571.2263	12.75	571.2313	16.75
571.2575	9	571.2675	15.5
571.295	12	571.3038	21.75
571.325	10.5	571.3375	19.25
571.36	11	571.3675	21.25
571.395	11.25	571.4	23
571.4275	10.75	571.435	19.75
571.4588	10.75	571.4663	16
571.4925	9.25	571.5	19.25
571.5263	12.5	571.535	19.75
571.56	8.5	571.57	18
571.5925	11.5	571.6038	16.75
571.6275	9.75	571.6363	14.25
571.6613	14.75	571.67	18.75
571.6963	13.5	571.7013	21.75
571.7263	10	571.7313	15.5
571.7575	8.5	571.7675	14.25
571.795	13.25	571.8013	18.5

571.825	12	571.8375	18.5
571.86	8.5	571.8675	15.25
571.895	10.75	571.9	15.75
571.9275	12.25	571.935	19.25
571.9588	11.5	571.9663	20.5
571.9925	13.5	572	19
572.0263	12.75	572.035	18
572.06	12.25	572.07	17
572.0925	11.25	572.1038	21
572.1263	15.25	572.1363	22
572.1613	11.75	572.17	22
572.1963	12.75	572.2013	23.25
572.2263	9.5	572.2313	19.75
572.2575	12	572.2675	21
572.2925	12	572.3013	20
572.325	11.75	572.3375	22.75
572.36	9.75	572.3675	20.75
572.395	12.5	572.4	18.75
572.4275	10.25	572.435	20
572.4588	12.5	572.4663	26
572.4925	11.25	572.5	22.5
572.5263	7.75	572.5363	21.25
572.56	13.75	572.5725	20
572.5925	11.5	572.6038	20.25
572.6263	11.25	572.6363	21.75
572.6613	12	572.67	19.25
572.6963	13	572.7013	21
572.7263	14.5	572.7313	18.75
572.7575	12.25	572.7675	20.75
572.795	10.25	572.8013	17.5
572.825	11.75	572.8375	18.25
572.86	14.5	572.8675	15
572.895	11.5	572.8975	17.75
572.9275	14.5	572.935	21
572.9588	11.75	572.9663	16.75
572.9925	10.25	573	21.5
573.0238	14.25	573.035	17.75
573.06	11	573.0725	15.5
573.0925	10	573.1038	17.5
573.1263	10	573.1363	18
573.1613	11.75	573.17	16.75
573.1963	15.25	573.2013	19.25
573.2263	10.75	573.2313	16.25
573.2575	10.25	573.2675	17
573.2925	11.5	573.3038	19.5
573.325	9.5	573.34	16.5
573.36	12.5	573.37	20
573.3975	13	573.4025	18.5
573.4275	14.5	573.4363	18.5
573.4588	10	573.4675	18

573.4925	13.25	573.5038	18
573.5238	14	573.5363	20.5
573.56	11	573.5738	20.5
573.5925	12.25	573.605	17.25
573.6263	11.5	573.6375	19.25
573.6613	9.5	573.6738	16
573.6963	9.25	573.7038	20.5
573.7263	10	573.7338	20.5
573.76	9	573.77	19
573.7925	9.5	573.8038	22
573.825	11.75	573.84	14.75
573.8625	9.25	573.87	18.75
573.8975	10.75	573.9	19.75
573.9275	10.25	573.9363	19.25
573.9588	11.5	573.9675	20.5
573.9925	9	574.0038	18.25
574.0238	7.75	574.0363	15
574.06	11	574.0738	14
574.0925	11.25	574.105	14.75
574.1263	12.75	574.1375	20.25
574.1613	12.25	574.1725	16.75
574.1963	10.5	574.2038	16.75
574.2263	12.25	574.2338	14
574.26	11.25	574.27	13.25
574.2925	11.5	574.3038	16
574.325	11.5	574.34	14.75
574.3625	12	574.37	15.25
574.3975	11	574.4025	15.5
574.4275	9.5	574.4363	20
574.4588	10.75	574.4675	23
574.4925	8.75	574.5038	16.5
574.5238	10.5	574.5363	15.5
574.56	12.5	574.5738	17
574.5925	12.25	574.6063	16
574.6263	11.5	574.6375	17.5
574.6613	9.5	574.6725	16.75
574.6963	12	574.7038	15.25
574.7263	12.75	574.7338	17
574.76	13	574.77	18
574.7925	12.75	574.8063	12.75
574.825	10.5	574.84	13.75
574.8625	11.75	574.87	17
574.8975	12.5	574.9025	17.75
574.9275	10	574.9388	20
574.9588	10.75	574.9675	15
574.9925	9.75	575.0038	14
575.0238	10.5	575.0363	18.25
575.0588	11	575.0738	18.5
575.0925	11.75	575.105	19.5
575.1263	12	575.1375	20.5

575.1613	12
575.1963	9.5
575.2263	11.75
575.26	10.5
575.2913	10.25
575.325	13.25
575.365	11.75
575.3975	10.5
575.4275	11
575.4588	12.25
575.4925	9.75
575.5238	11.25
575.5588	12.5
575.5938	9
575.63	12.25
575.6625	11
575.6975	10
575.7275	7.75
575.7613	12.25
575.7938	11
575.8275	11
575.865	9
575.9	7.25
575.93	10
575.9613	9.75
575.995	10
576.0263	8
576.06	10.75
576.095	8.5
576.1325	9
576.1625	7.5
576.1975	12
576.2275	7.5
576.2613	9
576.2938	10
576.3275	8.75
576.365	10
576.4	12.25
576.43	12.5
576.4613	14.75
576.4963	14.5
576.5263	12.5
576.56	15.25
576.595	12.25
576.6325	11.25
576.6625	9.75
576.6975	13.75
576.7275	14.25
576.7613	14
576.7938	10.25

575.1725	18
575.2038	21
575.2363	15.75
575.27	17.75
575.3063	21.75
575.34	16.5
575.37	17.5
575.4025	17.25
575.4363	21.25
575.4675	16.75
575.5013	22.25
575.5363	15.75
575.5738	19.75
575.605	19.25
575.6375	17.75
575.67	21.75
575.7038	20
575.7363	22.5
575.77	22
575.8088	22.5
575.84	24
575.87	20.75
575.9025	18.25
575.9363	19.25
575.9675	20.25
576.0013	22
576.0388	20.25
576.0738	16.5
576.105	18
576.1375	21.25
576.17	21.25
576.2038	20.5
576.2363	23
576.27	23.75
576.3088	23.5
576.34	22
576.37	24.5
576.4025	20.5
576.4363	18.5
576.4675	22
576.5013	22.75
576.5388	21.75
576.5738	23.75
576.605	19.5
576.6375	17.5
576.67	17.25
576.7038	23.5
576.7388	18.5
576.77	24.25
576.8088	25.5

576.8275	14.25	576.84	26.5
576.865	12.5	576.87	26.5
576.9	9.25	576.9025	24
576.93	10	576.9363	24.25
576.9613	8.75	576.9675	24.75
576.995	13.25	577.0013	25.5
577.0263	7.25	577.0388	26
577.06	8.75	577.0738	21.75
577.095	10.75	577.105	24.25
577.1325	8.25	577.1375	25.5
577.1625	10.5	577.17	22.75
577.1975	11	577.2038	21.25
577.2275	11.75	577.2363	20.5
577.2613	11.5	577.27	20.5
577.2938	9	577.3088	20
577.3275	12.5	577.34	19.25
577.365	10.75	577.37	15.5
577.4	11	577.4025	18.25
577.43	11.25	577.4363	18.25
577.4613	12.5	577.4675	16.5
577.4963	11.75	577.5013	17
577.5263	11.25	577.54	21
577.56	12.25	577.5738	17.25
577.595	15.5	577.605	21.5
577.6325	11	577.6375	16.5
577.6625	13.5	577.67	18
577.6975	11	577.7038	14.5
577.7275	10.5	577.7363	16
577.7613	10.25	577.77	18.5
577.7938	10.75	577.8088	15
577.825	10.5	577.84	17.5
577.865	12.25	577.87	16
577.9	9.75	577.9025	16.25
577.93	10.25	577.9363	16.75
577.9613	8	577.9675	18
577.9975	10.25	578.0013	19.25
578.0263	13.25	578.04	18.75
578.0575	12	578.0738	17.75
578.095	9.5	578.105	22
578.1325	9.5	578.1375	20.5
578.165	8.75	578.17	25.25
578.195	8	578.2038	24.5
578.2275	11	578.2363	20
578.2613	9.25	578.2725	18.75
578.2938	9.5	578.3088	22.25
578.325	11	578.34	27
578.365	11.75	578.37	18
578.4	11.75	578.4025	24.5
578.43	10.75	578.4363	21.25
578.4613	10.5	578.4675	22.75

578.4963	10.25	578.5013	24.5
578.5263	10.5	578.54	21.5
578.5575	9.75	578.5738	19
578.595	11	578.605	16.5
578.6325	13.5	578.6375	17.75
578.6625	11	578.67	18.5
578.695	11.75	578.7038	19.75
578.7275	10.75	578.7363	19
578.7613	11.75	578.7738	22.5
578.7938	12.25	578.8088	18.25
578.825	13.25	578.84	21
578.8663	12	578.8725	21.5
578.9	12.5	578.9025	18
578.93	11.5	578.9363	22.5
578.9613	13	578.9675	22.5
578.9963	14.5	579.0013	23.25
579.0263	14	579.0413	27
579.0575	14.5	579.0738	20.25
579.095	11.5	579.105	23.75
579.1325	14	579.1375	18
579.1625	11.5	579.17	18.75
579.195	10.75	579.2038	18.25
579.2275	11.5	579.2363	18.25
579.2613	14.5	579.2738	18.75
579.2938	11.5	579.3088	19.25
579.325	8.75	579.34	21.5
579.3663	10.25	579.37	18
579.4	13.25	579.4025	24.5
579.4313	8.5	579.4363	19.5
579.4613	11.5	579.47	21.5
579.4963	9.75	579.5013	19
579.5263	11.25	579.5413	22
579.5575	14.5	579.5738	24
579.595	12.25	579.605	23
579.6325	9	579.6375	25
579.6625	12.5	579.67	21.75
579.695	13.5	579.7038	21.75
579.7275	10.75	579.7363	22.5
579.7613	10.5	579.7763	24.25
579.7938	11.25	579.8088	23.75
579.825	10.25	579.84	25.75
579.8663	9.25	579.87	22.25
579.9	9.75	579.9025	17.25
579.93	9.75	579.9363	22
579.9613	9.25	579.9675	26.5
579.9963	10.25	580.0025	23
580.0263	10	580.0413	22
580.0575	9.25	580.0738	17.25
580.0925	11	580.105	19.25
580.1313	13.75	580.1375	16.75

580.1625	11.75	580.17	18.75
580.195	12	580.2038	18.75
580.2275	13.25	580.2363	19
580.2613	12	580.2763	16.75
580.2938	12.25	580.3088	24.5
580.3263	9.25	580.34	19.25
580.3688	12	580.37	20.25
580.4013	13.5	580.4025	21
580.4313	10	580.4363	23
580.4625	8	580.4675	19.5
580.4975	12	580.5025	22.5
580.5313	10	580.5413	20.25
580.5625	10.5	580.5738	27
580.5988	9.5	580.605	26
580.6338	8.75	580.6375	22.25
580.6663	9	580.67	23.5
580.6988	11	580.7038	21.5
580.7338	13.25	580.7363	26
580.765	10.5	580.7763	19
580.7963	12	580.8088	27
580.8275	10.75	580.84	25
580.8713	11.25	580.87	22
580.9038	12.75	580.9	28
580.9338	7	580.9363	25.5
580.9675	9.25	580.9675	22.75
581	11	581.0025	22.5
581.0313	10.25	581.0413	20.5
581.0625	10.5	581.0738	23.25
581.0988	9	581.105	22.25
581.1338	9	581.1375	21.25
581.1688	10	581.17	22.75
581.1988	10.25	581.2038	25.25
581.2363	8.5	581.2363	22.5
581.265	8.5	581.2763	21
581.2963	10.75	581.3088	24
581.33	12	581.34	20.75
581.3713	13	581.37	24.5
581.4038	10.75	581.4	16
581.4338	9.5	581.4363	23.25
581.4675	11	581.4675	25.75
581.5	8.75	581.5025	24
581.5313	10	581.5413	22
581.5625	11.25	581.5738	24
581.5988	10.25	581.605	23.25
581.6338	8.5	581.6375	20.25
581.6663	12.25	581.67	21.5
581.6988	9	581.7038	21
581.7338	13	581.7388	23.5
581.765	13.25	581.7763	26.75
581.7963	11.75	581.8088	19

581.83	12.5	581.84	20.75
581.8713	8.25	581.87	22.75
581.9038	9.5	581.9025	23
581.9338	9.75	581.9363	16.75
581.9663	14.75	581.9675	19.75
582	13.75	582.0063	18.75
582.0313	11.75	582.0413	25.75
582.0625	11	582.0738	20.75
582.0988	9	582.105	19.75
582.1338	12	582.1375	19.75
582.1663	10.5	582.17	20.25
582.1988	8.25	582.2038	24
582.2338	11.75	582.2388	23.5
582.2675	10.75	582.2763	20.75
582.2963	14	582.3088	23.25
582.3325	12	582.34	22.25
582.3713	9.25	582.37	21.5
582.4038	12	582.4	20.5
582.4338	10.5	582.4363	21
582.4663	8.75	582.47	21.25
582.5	9.25	582.5038	25.75
582.5313	12.5	582.5413	27.75
582.5625	8.25	582.5738	23.75
582.5988	9.75	582.605	17
582.6338	12.25	582.6375	20.5
582.6663	6.75	582.67	22.5
582.6988	8.5	582.7038	23.5
582.7338	9.25	582.7388	21.25
582.765	9.75	582.7763	19.75
582.7963	11.25	582.8088	20
582.83	9.5	582.84	18.5
582.87	12	582.87	18.25
582.9038	10	582.9	21
582.9338	10.25	582.9363	16.75
582.9663	10.5	582.9675	20.75
583	10	583.0038	15.75
583.0313	9.5	583.0413	22.25
583.0625	10.5	583.0738	19.75
583.0988	11	583.105	20
583.1338	10.5	583.1375	16.75
583.1663	10.5	583.17	18.75
583.1988	9.25	583.2063	19
583.2338	9.25	583.2388	18
583.265	8.25	583.2788	18.75
583.2963	9	583.3088	17
583.33	6.25	583.34	16.75
583.37	9.75	583.37	17.75
583.4038	8.75	583.4	21.5
583.4338	7.5	583.4363	19.25
583.4663	7.75	583.4688	17

583.5	9	583.5038	21.25
583.5313	8.25	583.5413	16.75
583.5625	10.75	583.5738	17.75
583.5988	12	583.605	23.25
583.6338	12	583.6375	17
583.6663	9.25	583.6725	19.5
583.6988	8.75	583.705	20.5
583.7338	7.25	583.74	20.5
583.765	9.75	583.78	14.5
583.7963	12	583.8113	20.75
583.83	10	583.8425	21.25
583.87	10	583.8725	17.5
583.9038	10	583.9025	17.5
583.9338	9.5	583.9388	20.75
583.9663	9.25	583.9713	19
584	8.25	584.0063	16.5
584.0313	7.5	584.045	17.5
584.0625	7.5	584.075	16.25
584.0988	10	584.1063	14
584.1363	11.25	584.1388	17.25
584.1663	8.75	584.1738	14.5
584.1988	7	584.205	17
584.2338	9.5	584.2425	14.75
584.265	6.5	584.28	12.5
584.2963	9.75	584.3113	17.25
584.33	7.75	584.3425	11.5
584.37	5.75	584.3725	16.75
584.4038	8.5	584.4025	14.5
584.4338	13.25	584.4388	17.5
584.4663	12.25	584.4713	20
584.5	12	584.5113	18.5
584.5313	7	584.5438	17.5
584.5625	9.5	584.575	17.25
584.5988	7	584.6063	19.5
584.6338	7.75	584.6388	22.25
584.6663	10	584.6725	16
584.7013	8.25	584.7075	14
584.7338	7.5	584.7425	19.5
584.765	8.75	584.78	17.5
584.7963	11.5	584.8113	17.75
584.83	6.75	584.8425	15.25
584.87	9.75	584.8725	17
584.9038	7.25	584.9025	20
584.9338	10.25	584.9388	17.25
584.9663	10.75	584.9713	17.75
585	7	585.0113	21.25
585.0313	6.5	585.0438	14
585.0638	9	585.075	19.75
585.0988	9.25	585.1063	19.75
585.1338	11	585.1388	23

585.1663	7.25	585.1725	17.5
585.2013	6.5	585.2075	20.25
585.2338	9	585.2425	20
585.265	9.25	585.28	18.75
585.2963	9.5	585.3113	19.75
585.33	8.5	585.3425	23.5
585.37	10	585.3725	21.5
585.4038	12.25	585.405	21
585.435	10.25	585.4388	23.75
585.4663	12.75	585.4738	17.5
585.5	10	585.5113	23.25
585.5313	11.75	585.5438	19.75
585.5638	8.25	585.575	20.75
585.5988	10	585.6063	21
585.6338	11	585.6388	20.25
585.6663	11.5	585.6738	23.25
585.7013	10.75	585.7075	18.25
585.7338	10.5	585.7425	16
585.765	11.75	585.78	17
585.7963	12.25	585.8113	19.25
585.83	10	585.8425	19.25
585.87	11.25	585.8725	21.25
585.9038	9	585.905	20.25
585.9338	11.75	585.9388	18.75
585.9663	10.5	585.9738	18.25
586	10.25	586.0113	22
586.0313	11	586.0438	20.75
586.0638	10.5	586.075	24.5
586.0988	11	586.1063	20
586.135	10.75	586.1388	20.5
586.1675	7.75	586.1725	18.25
586.2025	8	586.2075	21.75
586.2363	14.75	586.2425	21.25
586.2675	10.5	586.2813	20
586.2988	10.75	586.3113	19
586.3325	6.75	586.3425	15.75
586.3738	9.25	586.3725	22.75
586.4063	7.75	586.405	20.5
586.4363	11.5	586.44	16.5
586.4688	8.25	586.4738	17.5
586.5013	9.25	586.5113	18.5
586.5325	8.5	586.5438	20.5
586.565	10.25	586.575	18.5
586.6013	12.5	586.6063	15.75
586.635	11.5	586.6388	16
586.6675	10.25	586.6725	15.75
586.7025	13.5	586.7075	13.75
586.7363	14.25	586.7425	16.5
586.7675	10.5	586.78	16.75
586.7988	9.25	586.8113	17.25

586.8325	10
586.8738	11.5
586.9063	12.5
586.9363	11.5
586.9688	14.5
587.0013	9
587.0325	15.75
587.065	10
587.1013	8.5
587.135	9
587.1675	10
587.2025	9.25
587.2363	13.75
587.2675	8.25
587.2988	12
587.3325	11.75
587.3713	10.25
587.4063	10.75
587.4375	11.5
587.4688	15.75
587.5013	11.5
587.5325	10.25
587.565	13
587.6013	10.5
587.635	11.75
587.6675	9
587.7025	13.5
587.7363	8.5
587.7675	12
587.7988	9.25
587.8325	9.75
587.8713	8.75
587.9063	11.25
587.9375	12.25
587.9688	12
588.0013	11.75
588.0325	12.75
588.065	10.25
588.1038	12.25
588.135	10.25
588.1675	13.25
588.2025	11.25
588.2363	8.75
588.2675	11.75
588.3013	10.25
588.3325	10.25
588.3713	11.75
588.4063	12.25
588.4375	14
588.4688	11.75

586.8425	21
586.8725	18
586.905	14.75
586.94	11.25
586.975	19.75
587.0113	17.25
587.045	17.25
587.075	14.25
587.1063	16.25
587.1388	17
587.1725	13.5
587.2075	12.75
587.2425	13.25
587.28	12.25
587.3113	14
587.3425	12.5
587.3725	12
587.405	13
587.44	16.75
587.475	12
587.5113	14.75
587.5438	13.25
587.575	13.25
587.6063	14.5
587.6388	12.25
587.6725	13.25
587.7075	14.5
587.7425	13
587.78	11.25
587.8113	11.75
587.8425	11
587.8725	13
587.905	14
587.9375	16.25
587.975	16.25
588.0113	16.5
588.0438	13.5
588.075	14
588.1063	13.75
588.1413	12.5
588.175	14.75
588.2075	9.75
588.2425	11
588.28	12
588.3113	12
588.345	11
588.3725	11.25
588.405	14.25
588.4375	13.25
588.4763	12.5

588.5013	12.75	588.5113	12
588.5325	14.25	588.5438	12
588.565	16.75	588.575	12
588.6013	15	588.6063	12.5
588.635	18.5	588.6413	10
588.6675	17	588.675	13
588.7025	17.75	588.7075	11.25
588.7363	15.75	588.7425	13
588.7675	14.25	588.78	13
588.8013	14.25	588.8113	14.75
588.8325	14	588.8425	16.75
588.8713	12.5	588.8725	14.5
588.9063	14.5	588.905	16.75
588.9375	13	588.94	16.25
588.9688	13	588.9775	13.25
589.0013	11.5	589.0138	12.75
589.0325	13.75	589.0463	16
589.065	16.5	589.0775	15.25
589.1013	14.5	589.1088	12.75
589.135	12	589.1438	12
589.17	12.5	589.1763	12
589.2025	10	589.2088	12
589.2363	14.75	589.2463	15.25
589.2675	14.5	589.2813	13.25
589.3013	15.5	589.3125	13.25
589.3325	13.25	589.3438	12
589.3713	13.25	589.3738	16.5
589.4063	12	589.4075	14
589.4375	15	589.44	13.25
589.4688	15.75	589.4788	16
589.5013	18	589.5138	9.5
589.5325	13	589.5463	13.5
589.565	13.5	589.5775	14.25
589.6013	15	589.6088	11.25
589.635	16.5	589.6438	12.25
589.67	13.5	589.6763	14.75
589.705	10.25	589.7088	11
589.7363	18.25	589.7475	12.75
589.7675	15.25	589.7813	16.25
589.8013	11	589.8125	16.5
589.8325	10.5	589.8438	14.25
589.8713	9.5	589.875	12.75
589.9063	12	589.9075	14.5
589.9375	13	589.94	14
589.9688	14	589.9788	13.25
590.0013	11.25	590.0138	15
590.0325	11.75	590.0463	15.25
590.065	8.5	590.0775	13.75
590.1013	12.25	590.1088	14.75
590.135	8.75	590.1438	12.75

590.17	8.25	590.1763	10.75
590.2025	11.5	590.2088	11.75
590.2338	11.5	590.2475	11.75
590.2675	11	590.2813	11.25
590.3013	8.25	590.3125	10.25
590.3325	9	590.3438	10.5
590.3713	13.5	590.375	13.25
590.4063	12.5	590.4075	11.25
590.4375	11.5	590.44	13.25
590.4688	7.75	590.4788	14.5
590.5013	8	590.5138	9.75
590.5325	11.5	590.5463	12.75
590.5675	7.5	590.5775	14
590.6013	9.5	590.6088	14.25
590.635	10.75	590.6438	12.75
590.67	11	590.6763	14.5
590.7025	6.5	590.7113	13.75
590.7338	12	590.7475	15.25
590.7675	7.75	590.7813	12.5
590.8013	10.5	590.8125	13.5
590.8325	10	590.8438	12.75
590.8713	9.75	590.8775	12.5
590.9075	9.25	590.91	13.5
590.9388	8.25	590.94	17.5
590.9688	6.75	590.9788	13.75
591.0013	8.25	591.0138	10.75
591.0338	8.25	591.0463	13.75
591.065	7.25	591.0775	12.75
591.1013	8.25	591.1088	15.5
591.135	9	591.1438	17
591.17	9.75	591.1788	12.75
591.2025	7.75	591.21	13
591.2338	5.5	591.2475	13
591.27	8.5	591.2813	18.25
591.3013	7.25	591.3125	16.75
591.3313	7.75	591.3438	16.5
591.3713	7.75	591.375	16.25
591.405	8.25	591.41	17
591.4375	8	591.44	16
591.4688	9.5	591.4788	15.5
591.5013	9	591.5138	16.25
591.5338	6	591.5463	14.5
591.565	5	591.5775	15.5
591.6013	6.5	591.6113	12.5
591.635	6.25	591.6438	12.75
591.67	5.75	591.6763	13.5
591.7025	10.75	591.71	15.75
591.7338	7.5	591.7475	13.5
591.7675	11.5	591.7788	20.25
591.8013	10.75	591.8125	14.25

591.8313	7.25	591.8438	17.75
591.8713	7.25	591.875	15.75
591.905	8	591.91	17
591.9375	9.75	591.94	16.25
591.9688	8.5	591.9788	18.75
592.0013	9.25	592.0138	14.25
592.0338	7.5	592.0463	17
592.065	9.75	592.0775	20
592.1013	9.25	592.1113	15.75
592.135	12	592.1438	16.5
592.17	9.25	592.1763	16.5
592.2025	6.75	592.2125	17
592.235	9.75	592.2475	17.5
592.2675	9.5	592.2788	13.75
592.3013	9.25	592.3125	14.25
592.3313	13.25	592.3438	16.5
592.3713	6.75	592.375	14.25
592.405	11.5	592.41	19
592.4375	11	592.44	12.75
592.4688	10.25	592.4788	15
592.5013	8.75	592.5138	14.25
592.5338	8.25	592.5463	14
592.5675	9.25	592.5775	18.25
592.6013	11.25	592.6113	13
592.6375	7.5	592.6438	13.75
592.67	10.5	592.6763	19.25
592.7025	10	592.7125	19.25
592.7338	9.75	592.7475	15.25
592.7675	10	592.7788	15
592.8013	12.5	592.8125	19.25
592.8313	9	592.8438	20.5
592.8713	10.5	592.875	18.75
592.905	11	592.91	19.25
592.9375	10.75	592.94	16
592.9688	10.75	592.9788	18.75
593.0013	9.75	593.0138	14.75
593.0363	9.5	593.0463	15.5
593.065	12	593.0775	19.75
593.1013	10.25	593.1113	15.75
593.1375	10.5	593.1438	14.5
593.17	8.75	593.1763	12.25
593.2025	9.25	593.2125	13.5
593.2338	12.5	593.2475	19.25
593.27	11.75	593.2788	16
593.3013	8.75	593.3138	17.5
593.3313	10	593.3438	16.75
593.3713	8	593.375	14
593.405	10.5	593.41	14.75
593.4375	8.75	593.44	13.75
593.4688	11.25	593.4788	16.25

593.5013	11.5	593.5138	20.25
593.5338	8.25	593.5463	21.75
593.565	10.25	593.5775	17
593.6013	9.5	593.6113	16.75
593.6388	9.75	593.6438	17.75
593.67	10.25	593.6763	16
593.7025	7.5	593.7125	18
593.7338	10.25	593.7475	15
593.77	9.25	593.7788	15.5
593.8013	8.5	593.8125	19.75
593.8313	10.75	593.845	17.75
593.8713	11.5	593.875	13.75
593.905	11.25	593.91	16.5
593.9375	7.75	593.94	18
593.9688	7.5	593.9763	15.75
594.0013	7.25	594.0138	15.25
594.0338	12	594.0463	12.75
594.065	11	594.0775	15.25
594.1013	8.5	594.1113	17.75
594.1375	11.25	594.145	14.25
594.17	10.25	594.1763	17.75
594.2025	9.75	594.2125	12.5
594.2338	9.5	594.2475	17.25
594.27	10.75	594.2788	16.25
594.3013	8.5	594.3125	15.75
594.3338	13	594.345	17.75
594.3725	11	594.3775	15.5
594.405	11.25	594.41	16.25
594.4375	11.5	594.44	17.75
594.4688	9.75	594.4763	16.25
594.5013	12	594.5138	18.75
594.5338	12.25	594.545	19.5
594.565	12.25	594.5775	15.25
594.6013	12	594.6113	15
594.6375	5.75	594.645	15
594.67	11.5	594.6763	19
594.7025	10.5	594.7125	15.25
594.7338	10	594.7475	17.25
594.77	8.25	594.7788	18
594.8013	12.25	594.8125	17
594.8338	10.75	594.845	19.25
594.8725	10.25	594.875	17.25
594.905	10.75	594.91	11.25
594.9375	10.5	594.9413	16.75
594.9688	14.5	594.9763	13.75
595.0013	12.25	595.0138	19.25
595.0338	13.75	595.045	13.75
595.065	12.5	595.0775	15.75
595.1013	12.5	595.1113	17.25
595.1375	12.25	595.145	15.75

595.17	14.25	595.1763	12.5
595.205	13	595.2125	17.25
595.2338	11.75	595.2475	14
595.27	13.25	595.2788	14.5
595.3013	8.5	595.3125	14.5
595.3338	9.25	595.345	15.75
595.3725	10.75	595.375	16.5
595.405	11.25	595.41	15
595.4375	8.75	595.4413	17
595.4688	7.5	595.4763	18
595.5038	8.75	595.5138	14.75
595.5338	9.25	595.545	14
595.565	6.75	595.58	15.5
595.6038	9.5	595.6113	17.5
595.6375	9.75	595.645	17.75
595.67	10	595.6763	15.25
595.7025	5.75	595.7125	14.75
595.7338	9.75	595.7475	15.5
595.77	7	595.7788	16.5
595.8013	8.25	595.8125	17.25
595.8338	11.25	595.845	14.5
595.87	7.25	595.875	14.75
595.905	11.25	595.91	15.75
595.9375	10.5	595.9413	11.75
595.9688	11.25	595.9763	16.25
596.0013	11.25	596.0138	13.25
596.0338	11	596.045	10.5
596.065	12	596.0813	14.25
596.1038	13.5	596.1113	13.5
596.1375	14.5	596.145	14.25
596.17	13.25	596.1763	17.5
596.2025	11	596.2125	15.75
596.2338	11.5	596.2475	15.5
596.27	11.5	596.2788	16.75
596.3013	12.25	596.31	15
596.3338	13	596.345	12.75
596.37	13.5	596.375	14.75
596.405	11	596.41	15.25
596.4375	13.75	596.4413	13.25
596.4688	15.25	596.4763	11.75
596.5013	11.75	596.5138	15.25
596.5338	13	596.545	12.75
596.565	13	596.58	15.25
596.6038	13.5	596.6113	15
596.6375	14.5	596.645	12
596.6688	12.75	596.6788	13.25
596.7025	11.5	596.7125	12.75
596.7338	16	596.7475	11.75
596.77	12.75	596.7788	11
596.8013	14.75	596.81	12.25

596.8338	14.25	596.845	16.5
596.87	16.25	596.875	15.75
596.905	15.75	596.9113	16.75
596.9375	15	596.9413	12.25
596.9688	13.75	596.9763	17.5
597.0013	14.5	597.0138	15.25
597.0338	11.25	597.045	14.25
597.065	13.75	597.08	14
597.1038	11.5	597.1113	16
597.1375	11.5	597.145	13
597.1688	12.25	597.1788	14.75
597.2025	13.5	597.2125	16.5
597.2338	14.75	597.2475	15.75
597.27	13.5	597.2788	17.25
597.3013	11.75	597.3113	16
597.3363	8.75	597.345	17.25
597.37	12.25	597.375	18
597.405	10.25	597.41	15.75
597.4375	13.25	597.4413	18.5
597.4688	11.25	597.4763	14.75
597.5013	9.5	597.5138	15.25
597.5338	13.5	597.545	15.75
597.565	11.25	597.58	15.5
597.6038	11.25	597.6113	16.75
597.6375	11.5	597.645	19.75
597.67	11.75	597.6788	18.75
597.7025	13.75	597.7125	13.5
597.735	14.5	597.7475	14
597.77	9	597.7813	16
597.8013	11.75	597.8113	15.25
597.8363	14	597.845	14.25
597.87	12	597.875	16
597.905	12	597.91	11.5
597.9375	11.25	597.9413	14
597.9688	12.25	597.9763	13.75
598.0013	12	598.0138	11
598.0338	14	598.045	13.25
598.065	13.5	598.0788	11
598.1013	11	598.1113	9.5
598.1375	12.75	598.145	13.25
598.1688	10	598.1788	11
598.2025	14.5	598.2125	13.75
598.2363	10.5	598.2475	13.5
598.27	10.75	598.2788	17.75
598.3013	14.75	598.3113	15.5
598.3363	10.5	598.345	13.75
598.37	14.25	598.375	15
598.405	14.5	598.41	16
598.4375	20.75	598.4413	16
598.4688	10.75	598.4763	16.25

598.5013	17.25
598.5338	12.25
598.565	12
598.6013	11.25
598.6375	13
598.6688	12
598.7025	15
598.735	12.5
598.77	13.75
598.8013	12.25
598.8363	24.75
598.87	12
598.905	32.75
598.9375	15.25
598.9713	28.5
599.0013	22.5
599.0338	22.75
599.0675	18.25
599.1013	13.5
599.1375	14.75
599.1688	12.5
599.2025	8.5
599.235	14.5
599.2688	7.5
599.3013	13.5
599.3363	11
599.37	14.5
599.405	11.5
599.4375	14.75
599.4713	12.25
599.5013	14.5
599.5363	18.5
599.57	10
599.6025	16.25
599.64	16
599.6713	16.25
599.7038	12.5
599.7363	12
599.77	18.25
599.8025	16.75

598.5138	13
598.545	12.5
598.5788	17
598.6113	14.5
598.645	20
598.6788	20.5
598.7125	16
598.7475	18.5
598.7788	17.5
598.8113	16.25
598.845	14
598.875	19
598.91	17.5
598.9438	12.75
598.9763	11.5
599.0138	14.5
599.0475	11.5
599.0788	13.75
599.1113	15.25
599.145	15.75
599.1788	15.5
599.2125	16
599.245	12
599.2788	14
599.3113	16.5
599.3425	11.75
599.375	11.25
599.41	16.5
599.4413	14.25
599.4763	12.5
599.5138	15
599.5475	13
599.5788	14.5
599.6113	10.5
599.645	13.75
599.6788	13
599.7125	11.25
599.745	13
599.78	11.75
599.8113	11.25
599.8438	13.25
599.875	12.75
599.91	11.75
