# Supplementary material for: Bioinsecticide-Predator Interactions: Azadirachtin Behavioral and Reproductive Impairment of the Coconut Mite Predator Neoseiulus baraki
Source: PLoS One. 2015 Feb 13;10(2):e0118343. doi: 10.1371/journal.pone.0118343 (PMC4334557; doi:10.1371/journal.pone.0118343)
Supplement: S2 Dataset — (PDF) [file pone.0118343.s002.pdf]

Time in each group of activities
(segundos)

| | freezing | middle | burst | | freezing | middle | burst |
|---|---|---|---|---|---|---|---|
| controle | 22.9 | 73.6 | 503.6 | azamax | 125.5 | 179.2 | 295.3 |
| controle | 58.5 | 72.4 | 469.1 | azamax | 125 | 126.5 | 348.5 |
| controle | 16.1 | 42.2 | 541.8 | azamax | 100.9 | 133.5 | 365.6 |
| controle | 4.5 | 20.8 | 574.7 | azamax | 52.6 | 140.3 | 407.1 |
| controle | 7.2 | 22.5 | 570.3 | azamax | 16.9 | 40.6 | 542.5 |
| controle | 10.7 | 35.5 | 553.8 | azamax | 170.8 | 204.7 | 224.5 |
| controle | 8.4 | 45.2 | 546.4 | azamax | 8.2 | 36.4 | 555.5 |
| controle | 2.8 | 14.8 | 582.4 | azamax | 30.7 | 89.5 | 479.8 |
| | | | | | | | |
| media | 16.3875 | 40.875 | 542.7625 | | 78.825 | 118.8375 | 402.35 |
| EPM | 6.439899 | 7.944737 | 13.65735 | | 21.16422 | 21.35084 | 41.50274 |

Frequency in each group of activity
unidade

| | freezing | middle | burst | | freezing | middle | burst |
|---|---|---|---|---|---|---|---|
| controle | 553 | 1660 | 1713 | azamax | 2248 | 3307 | 2535 |
| controle | 818 | 1575 | 1414 | azamax | 1576 | 2610 | 2238 |
| controle | 319 | 970 | 974 | azamax | 1448 | 2618 | 2150 |
| controle | 116 | 521 | 555 | azamax | 1198 | 2758 | 2681 |
| controle | 171 | 534 | 575 | azamax | 344 | 883 | 881 |
| controle | 245 | 787 | 809 | azamax | 2669 | 3846 | 2846 |
| controle | 227 | 1065 | 1154 | azamax | 196 | 845 | 860 |
| controle | 65 | 333 | 336 | azamax | 700 | 1868 | 1846 |
| | | | | | | | |
| media | 314.25 | 930.625 | 941.25 | | 1297.375 | 2341.875 | 2004.625 |
| EPM | 89.15731 | 172.6734 | 165.4308 | | 309.7824 | 380.5227 | 271.2455 |
