# Supplementary material for: Bioinsecticide-Predator Interactions: Azadirachtin Behavioral and Reproductive Impairment of the Coconut Mite Predator Neoseiulus baraki
Source: PLoS One. 2015 Feb 13;10(2):e0118343. doi: 10.1371/journal.pone.0118343 (PMC4334557; doi:10.1371/journal.pone.0118343)
Supplement: S3 Dataset — (PDF) [file pone.0118343.s003.pdf]

treatment	treat code	replicate	walking	mounting	coupling
			time (min)		
untreated coupled	1	1	0.28	0.3	363.25
untreated coupled	1	2	0.03	0.52	239.93
untreated coupled	1	3	0.3	0.1	360.37
untreated coupled	1	4	1	0.18	418.31
untreated coupled	1	5	1.55	0.35	358.28
untreated coupled	1	6	1.67	0.02	238.13
untreated coupled	1	7	0.38	0.52	299.12
untreated coupled	1	8	0.52	0.12	239.86
untreated coupled	1	9	0.65	0.13	479.47
untreated coupled	1	10	0.62	0.23	360.12
untreated coupled	1	11	1.55	1.98	297.24
untreated coupled	1	12	0.98	0.44	419.08
untreated coupled	1	13	0.33	0.27	359.88
untreated coupled	1	14	1.75	1.1	357.65
untreated coupled	1	15	0.98	0.41	418.86
untreated coupled	1	16	0.87	0.16	419.12
untreated coupled	1	17	0.37	0.35	239.53
untreated coupled	1	18	1.62	0.53	358.45
untreated coupled	1	19	1.62	0.53	238.33
untreated coupled	1	20	3.47	0.09	296.86
treated female	2	1	0.98	0.4	293.62
treated female	2	2	0.77	0.09	363.14
treated female	2	3	4.55	0.27	298.18
treated female	2	4	0.52	0.35	297.13
treated female	2	5	0.62	1.96	330.42
treated female	2	6	0.25	0.05	97.7
treated female	2	7	8.92	0.7	65.38
treated female	2	8	0.28	0.22	404.5
treated female	2	9	0.17	0.83	239.58
treated female	2	10	0.57	0.38	323.05
treated female	2	11	0.32	0.28	383.4
treated female	2	12	0.42	0.05	439.53
treated female	2	13	0.5	0.12	431.38
treated female	2	14	0.03	0.08	29.89
treated female	2	15	0.73	0.2	401.07
treated female	2	16	0.87	0.29	221.84
treated female	2	17	0.28	0.32	250.4
treated female	2	18	0.37	0.92	230.71
treated female	2	19	2.37	0.74	326.89
treated female	2	20	0.67	0.27	343.06
treated male	3	1	2.05	0.53	231.42
treated male	3	2	2.92	0.04	249.04
treated male	3	3	2	0.13	310.87
treated male	3	4	1.93	0.18	420.49
treated male	3	5	1.65	0.42	262.76
treated male	3	6	1.65	0.47	158.88

treated male	3	7	1.52	0.25	124.59
treated male	3	8	1.58	0.37	93.05
treated male	3	9	1.2	0.83	38.97
treated male	3	10	2.35	0.46	388.55
treated male	3	11	3.15	0.2	33.43
treated male	3	12	0.92	0.25	111.83
treated male	3	13	7	0.05	113.95
treated male	3	14	0.3	0.47	354.23
treated male	3	15	1.12	0.17	390.71
treated male	3	16	0.92	0.98	267.1
treated male	3	17	1.86	0.36	231.78
treated male	3	18	0.05	0.24	236.71
treated male	3	19	2.03	0.27	230.7
treated male	3	20	1.22	0.72	436.06
treated couple	4	1	0.67	0.85	454.48
treated couple	4	2	0.75	1.1	362.15
treated couple	4	3	0.4	0.34	326.26
treated couple	4	4	0.22	0.33	351.45
treated couple	4	5	1.35	1	519.57
treated couple	4	6	3.07	0.5	16.33
treated couple	4	7	1.67	0.2	434.23
treated couple	4	8	1.28	0.22	418.03
treated couple	4	9	2.35	0.35	443.3
treated couple	4	10	3.3	0.24	327.23
treated couple	4	11	0.67	0.28	409.05
treated couple	4	12	0.32	0.34	239.34
treated couple	4	13	0.38	0.38	76.24
treated couple	4	14	0.57	0.37	385.06
treated couple	4	15	2.93	0.42	270.93
treated couple	4	16	0.3	0.2	45.5
treated couple	4	17	0.88	0.31	483.81
treated couple	4	18	0.3	0.43	421.27
treated couple	4	19	3.03	0.2	353.77
treated couple	4	20	1.35	0.71	345.24
